# Unveiling the diversity of the families *Cyphellaceae* and *Mycenaceae* from Southeast Asia

**DOI:** 10.3897/imafungus.17.182174

**Published:** 2026-07-10

**Authors:** Long Fei Fan, Arooj Naseer, Ammara Khalid, Abdul Nasir Khalid, Yan Deng, Chen Yan Wang, Yuan Wang, Si Xuan Ma

**Affiliations:** 1 College of Plant Protection, Gansu Agricultural University, Lanzhou, China Institute of Botany, University of the Punjab Lahore Pakistan; 2 Institute of Botany, University of the Punjab, Lahore, Pakistan College of Plant Protection, Gansu Agricultural University Lanzhou China

**Keywords:** Diversity, mycenoid fungi, systematics

## Abstract

Nine new species of the families *Cyphellaceae* and *Mycenaceae* are described from Asia, China, and Pakistan on the basis of detailed morphological and molecular investigations. Seven species belong to the genus *Mycena* (*M. albomarginata*, *M. oblongispora*, *M. lavendula*, *M. longnanensis*, *M. minispora*, *M. pakistanica*, and *M. tephroleuca*), and one species each belongs to *Atheniella* and *Phloeomana*. These *Mycena* species belong to three sections. Five *Mycena* species, i.e., *M. lavendula*, *M. longnanensis*, *M. minispora*, *M. pakistanica*, and *M. tephroleuca*, have been assigned to *Mycena sect. Calodontes*, while one species (*M. albomarginata*) belongs to *Mycena* sect. *Mycena* and one (*M. oblongispora*) to *Mycena sect. Fragilipedes*. All of the new species are supported by phylogenetic analyses based on the nuclear internal transcribed spacer (ITS) of nuclear ribosomal DNA and translation elongation factor 1-alpha (*tef1-α*).

## Introduction

The family *Mycenaceae* Overeem consists of small, fragile, white-spored saprotrophic fungi that are widely distributed worldwide across a variety of ecological communities. *Mycena* (Pers.) Roussel (*Mycenaceae*, *Agaricales*, *Basidiomycota*) is a large, widely distributed genus comprising approximately 1,312 species (He et al. 2019; Kalichman et al. 2020; Zhang et al. 2024; Aronsen 2025). It is characterized by fragile, small basidiomata, a thin, convex pileus with a sulcate margin, non-deliquescent lamellae, and a hollow stipe (Persoon 1797). Species of *Mycena* play important roles in ecosystems; they include edible and saprotrophic taxa; form symbiotic associations with *Orchidaceae* seeds (as orchidoid mycorrhizae) that enhance germination (Lee et al. 2017; Liu et al. 2025); function as decomposers (Guerreiro et al. 2023); help promote nutrient cycling, forest metabolism, and natural renewal; include bioluminescent taxa (Cortés-Pérez et al. 2019, 2023; Lu et al. 2024); and represent promising sources of bioactive secondary metabolites (Jaeger and Spiteller 2010).

The genus *Mycena* has been divided into multiple clades based on multilocus phylogenetic analyses using internal transcribed spacer (ITS), nuclear large subunit (nLSU), and small subunit (SSU) markers and into 23 sections according to their morphological features (Na et al. 2022; Cortés-Pérez et al. 2023). The taxa described during this study cluster into three *Mycena* sections: *Mycena sect. Fragilipedes* (Fr.) Quél. (*M. oblongispora*), *Mycena sect. Calodontes* (Fr. ex Berk.) Quél. (*M. lavendula*, *M. longnanensis*, *M. minispora*, *M. pakistanica*, and *M. tephroleuca*) and *Mycena* sect. *Mycena* (Pers.) Roussel (*M. albomarginata*).

*Mycena sect. Calodontes* is characterized by a pinkish, reddish, purplish to brownish, and mostly hygrophanous pileus, interveined lamellae, smooth cheilocystidia and pleurocystidia (when present), and mostly amyloid spores (Fries 1821; Berkeley 1836; Maas Geesteranus 1992a, 1992b; Harder et al. 2010). In contrast, *Mycena sect. Fragilipedes*, the largest section, is distinguished by its grayish-brown to dark brown pileus, along with smooth cystidia and a pileipellis typically covered with excrescences, as well as amyloid basidiospores. On the other hand, *Mycena* sect. *Mycena* is characterized by its relatively large and robust basidiomata, a typically grayish to brownish pileus with a lubricous or viscid surface, and a rigid, often rooting stipe. Microscopically, it is distinguished by having cheilocystidia that are often covered with short or long, simple to branched excrescences, which are characteristic of this section (Maas Geesteranus 1985, 1992a; Aronsen and Læssøe 2016).

The genus *Mycena* has been recognized as highly polyphyletic (Moncalvo et al. 2002; Aronsen and Læssøe 2016). In recent years, several new genera have been proposed to accommodate non-amyloid species formerly placed within *Mycena*, such as *Atheniella* Redhead, Moncalvo, Vilgalys, Desjardin & B.A. Perry, *Phloeomana* Redhead (Redhead 2012, 2013), *Pruinomycena* Kun L. Yang, Jia Y. Lin & Zhu L. Yang (Yang et al. 2025), and amyloid species such as *Chrysomycena* Vizzini, Picillo, Perrone & Dovana (Vizzini et al. 2019; Villarreal et al. 2021).

Recent phylogenetic studies have placed these fungal taxa across several independent evolutionary lineages (the “hydropoid fungi”) within the families *Porotheleaceae* Murrill and *Cyphellaceae* Lotsy (Eberhardt et al. 2018). These families belong to the suborder *Marasmiineae* Aime, Dentinger & Gaya, as circumscribed on a phylogenomic basis by Dentinger et al. (2016), a grouping that corresponds to the *Marasmioid* clade *sensu*Matheny et al. (2006) and Binder et al. (2010).

The family *Cyphellaceae*, typified by *Cyphella* Fr., is a small monophyletic family comprising a heterogeneous assemblage of lignicolous fungi with diverse macromorphology, including agaricoid, cyphelloid, clavarioid/typhuloid, pleurotoid, corticioid, or stereoid basidiomata. Among these, the agaricoid–mycenoid forms are represented by six genera: *Atheniella*, *Hemimycena*, *Mycenella*, *Mycopan*, *Phloeomana*, and *Pleurella*. In the present study, two new species belonging to the genera *Atheniella* and *Phloeomana* are described.

The genus *Atheniella*, typified by *Atheniella adonis* (Bull.) Redhead, Moncalvo, Vilgalys, Desjardin & B.A. Perry, is characterized by its brightly colored pileus, inamyloid tissues, and pileipellis covered with simple to branched excrescences. It is a recently introduced genus that was elevated from *Mycena* sect. *Adonideae* (Fr.) Quél. (Redhead 2012). It shares characteristics with *Mycena*, such as small basidiomata, white lamellae, a hollow stipe, and a saprotrophic habit on rotten wood or plant debris. However, *Atheniella* is differentiated from *Mycena* by a brightly colored pileus (e.g., yellow, orange, pink, or red) and all tissues unreactive in Melzer’s reagent (Redhead 2012).

The genus *Phloeomana* Redhead, typified by *Phloeomana speirea* (Fr.) Redhead, is characterized by its mycenoid basidiomata, non-amyloid basidiospores, and pileipellis hyphae with branched excrescences. Previously, *Phloeomana* was placed in the family *Porotheleaceae* (order *Agaricales*), formally proposed by Murrill (1916), which comprises saprotrophic, mainly wood-decaying fungi that are primarily agarics but also include cyphelloid fungi. Recently, however, the genus has been reassigned to the family *Cyphellaceae* (Eberhardt et al. 2018).

During various field surveys to collect fungal fruiting bodies for different research projects, researchers in China and Pakistan obtained several collections. Some taxa, morphologically similar to *Mycena* and allied species, were separated and analyzed both morphologically and through DNA sequencing (ITS and *tef1-α* regions). Several collections were found to have unique morphological characteristics. Phylogenetic tree reconstruction indicated that these collections are new species, clearly distinguished from closely related known species in *Atheniella*, *Mycena*, and *Phloeomana*, and are presented here as new species.

## Materials and methods

### Sampling and morphological observations

Basidiomata were collected and photographed in the field, and key morphological features—such as size, shape, and color—were noted fresh. Color codes were recorded using Munsell color charts (Munsell Color 1975). Basidiomata were dried with hot air and preserved in zip-lock bags. Micromorphological features were noted using an MX4300H microscope by mounting sections of various tissues in 5% KOH, Phloxine B (C_20_H_2_Br_4_Cl_4_Na_2_O_5_), and Melzer’s reagent. Photographs and measurements were obtained using calibrated Motic Images Plus 2.0 software. The abbreviation (*n*/*m*/*p*) refers to the number of basidiospores measured (*n*), the number of basidiomata (*m*), and the number of collections examined (*p*). Basidiospore dimensions are presented as (*a*) *b*–*c* (*d*), where (*a*) and (*d*) represent the extreme minimum and maximum values, and the range *b*–*c* includes at least 90% of all measured spores. *Q* denotes the length-to-width (*L*/*W*) ratio of individual spores, and av. *Q* represents the average *Q* value across all measured spores. Hyphal measurements are provided as ranges.

### Molecular phylogenetic study

For molecular analysis, genomic DNA was extracted using the cetyltrimethylammonium bromide (CTAB) method (Bruns and Grunert 1995). Polymerase chain reaction (PCR) amplification of the ITS region was carried out using primer pairs ITS1F or ITS5 with ITS4 (White et al. 1990), following the thermal cycling conditions described by Gardes and Bruns (1993). For the *tef1-α* gene region, primer pairs 983F/1567R were used. The PCR products were purified and sequenced, and consensus sequences were assembled. The resulting sequences were queried using BLAST at NCBI (http://www.ncbi.nlm.nih.gov/) to assess sequence homology, and closely related ITS and *tef1-α* sequences were downloaded. A combined ITS + *tef1-α* dataset was constructed to confirm the phylogenetic relationships of the new species. Additional sequences from published literature were incorporated to maximize taxon sampling and improve nucleotide-level comparisons. *Xeromphalina campanella* (Batsch) Kühner & Maire was chosen as the outgroup for rooting the phylogenetic tree (Na et al. 2022). All sequences were aligned separately using the MUSCLE algorithm available through the EMBL-EBI web platform (http://www.ebi.ac.uk/). Details of all sequences included in this study are shown in Table 1. The best-fit evolutionary model for the combined ITS + *tef1-α* dataset was determined using MrModeltest 2.3 (Nylander 2004).

**Table 1.** Specimens included in molecular phylogenetic analyses and their corresponding GenBank accession numbers.

**Species**	**Sample No**.	**Locality**	**GenBank Accession No**.		**References**
			**ITS**	***Tef-1α***	
*Atheniella adonis*	HMJAU 43213	China	MK309771	–	Ge et al. 2021
*A. adonis*	HMJAU 43193	China	MK309770	–	Ge et al. 2021
*A. amabillissima*	AFTOL–ID 1686	USA	DQ490644		Matheny et al. 2006
*A. amabillissima*	BD–2020a	FINLAND	MW540733		Ge et al. 2021
*A. aurantiidisca*	AFTOL-ID 1685	USA	DQ490646	**–**	Ge et al. 2021
*A. aurantiidisca*	PBM1282	USA	–	GU187728	Binder et al. 2010
*A. flavida*	FFAAS0350	China	MW969653		Ge et al. 2021
*A. flavida*	FFAAS0355	China	MW969654		Ge et al. 2021
*A. flavoalba*	CBS:258.53	Netherlands	MH857185	**–**	Ge et al. 2021
*A. flavoalba*	CBS:359.50	Netherlands	MH856659	**–**	Ge et al. 2021
*A. rutila*	FFAAS0354	China	MW969658	**–**	Ge et al. 2021
*A. rutila*	FFAAS0356	China	MW969659		Ge et al. 2021
*A. taoyao*	FFAAS0352	China	MW969656	**–**	Ge et al. 2021
*A. taoyao*	FFAAS0353	China	MW969657	**–**	Ge et al. 2021
*Leucoinocybe auricoma*	HKAS126433	China	OQ025169	**–**	Antonín et al. 2019
*L. auricoma*	AFTOL-ID 1341	USA	DQ490647	**–**	Antonín et al. 2019
*M. “amygdalina”*	HMJAU 43700	China	MT497545	–	Na and Bau 2020
*M. “amygdalina”*	HMJAU 43700R	China	MT497544	–	Na and Bau 2020
*M. acicula*	AH56040	Spain	OQ633209	**–**	Unpublished
*M. acicula*	H6036823	Hungary	MW540677	**–**	Unpublished
*M. aff. pura*	TL8052	Ecuador	FN394623	KF723641	Fan et al. 2024
*M. aff. pura*	TL9433	Ecuador	FN394622	KF723642	Fan et al. 2024
*M. aff. pura*	TL9450	Ecuador	KJ144653	KF723643	Fan et al. 2024
*M. aff. pura*	TL9678	Ecuador	FN394621	KF723644	Fan et al. 2024
*M. algeriensis*	HKAS 134295	China	PV706731	–	Lu et al. 2025
*M. alnetorum*	AH57250	Spain	PP868141	–	Villarreal et al. 2024
*M. alphitophora*	NX0491	China	MH136830	**–**	Na and Bau 2019
*M. alphitophora*	NX0679	China	MH136831	**–**	Na and Bau 2019
*M. brunneoviolacea*	BAP594	Africa	MH414546	–	Fan et al. 2024
*M. cahaya*	ACL134	Malaysia	KF537248	–	Fan et al. 2024
*M. cf. pura* I	CBH039	Denmark	FN394588	KF723634	Fan et al. 2024
*M. cf. pura* II	CBH105	Denmark	FN394581	KF723625	Fan et al. 2024
*M. cf. pura* II	CBH366	Denmark	FN394572	KF723627	Fan et al. 2024
*M. cf. pura* III	CBH019	Denmark	FN394605	KF723629	Fan et al. 2024
*M. cf. pura* III	CBH022	Denmark	FN394574	KF723630	Fan et al. 2024
*M. cf. pura* IV	CBH410	Denmark	FN394595	KF723621	Fan et al. 2024
*M. cf. pura* IV	JV06979	Denmark	FN394585	KF723622	Fan et al. 2024
*M. cf. pura* IX	CBH166	Denmark	FN394607	KF723655	Fan et al. 2024
*M. cf. pura* IX	CBH358	Denmark	FN394608	KF723656	Fan et al. 2024
*M. cf. pura* V	CBH226	Denmark	FN394604	KF723618	Fan et al. 2024
*M. cf. pura* V	TL5614	Denmark	FN394602	KF723620	Fan et al. 2024
*M. cf. pura* VI	BAP132	USA	FN394561	KF723614	Fan et al. 2024
*M. cf. pura* VII	IS10/11/2000	USA	FN394611	–	Fan et al. 2024
*M. cf. pura* VIII	CBH216	Denmark	FN394598	KF723616	Fan et al. 2024
*M. cf. pura* VIII	CBH402	Denmark	FN394599	KF723617	Fan et al. 2024
*M. cf. pura* X	BAP165A	USA	FN394563	KF723652	Fan et al. 2024
*M. cf. pura* XI	CBH187	Sweden	FN394564	KF723632	Fan et al. 2024
*M. cf. pura* XI	CBH386	Denmark	–	KF723633	Fan et al. 2024
*M. clavicularis*	HMJAU43611	China	ON791480	**–**	Wei et al. 2024
*M. clavicularis*	HMJAU43616	China	ON791479	**–**	Unpublished
*M. diosma*	CBH400	Denmark	FN394617	KF723653	Fan et al. 2024
*M. diosma*	LK1191/2000	Germany	FN394619	KF723654	Fan et al. 2024
*M. dura*	10315	Austria	FN394560	KF723648	Fan et al. 2024
*M. epipterygia*	olrim523	Sweden	AY805613	**–**	Unpublished
*M. epipterygia*	JB13	Sweden	GU234008	**–**	Unpublished
*M. filopes*	HMJAU43445	China	MH396634	**–**	Unpublished
*M. filopes*	HMJAU43562	China	MH396635	**–**	Zhang et al. 2024
*M. flos-nivium*	CBS:260.53	Netherlands	MH857186	**–**	Corriol and Jargeat 2023
*M. flos-nivium*	CBS:364.50	Netherlands	MH856661	**–**	Vu et al. 2019
*M. glabra*	FLF449	China	PP949212	PP967101	Fan et al. 2024
*M. glabra*	WBY449	China	PP949213	PP967102	Fan et al. 2024
*M. griseotincta*	HMJAU43805	China	MK309782	**–**	Na and Bau 2019
*M. griseotincta*	HMJAU43800	China	MK309783	**–**	Na and Bau 2019
*M. haematopus*	420526MF0200	China	MH142012	–	Unpublished
*M. haematopus*	420526MF0193	China	MH142010	**–**	Unpublished
*M. heteracantha*	HMJAU 43711	China	MK309786	–	Na and Bau 2019
*M. heteracantha*	HMJAU 43709	China	MK309785	–	Na and Bau 2019
*M. hyalinostipitata*	NX0686	China	MH136828	**–**	Na and Bau 2019
*M. hyalinostipitata*	NX0694	China	MH136829	**–**	Na and Bau 2019
*M. hygrophoroides*	HMJAU43417	China	MK309780	**–**	Na and Bau 2019
*M. hygrophoroides*	HMJAU43421	China	MK309781	**–**	Na and Bau 2019
*M. interrupta*	HMJAU43791	China	MK733300	**–**	Wei et al. 2024
*M. interrupta*	HMJAU43849	China	MK733301	**–**	Wei et al. 2024
*M. laevigata*	HMJAU43604	China	MK733303	**–**	Wei et al. 2024
*M. laevigata*	HMJAU43187	China	MK733302	**–**	Wei et al. 2024
*M. lammiensis*	TUR165927	Finland	FN394552	KF723651	Fan et al. 2024
*M. laurisilvae*	AH56022	Spain	OP382886		Ge et al. 2021
*M. laurisilvae*	AH56029	Spain	OP382887		Ge et al. 2021
*M. laurisilvae*	AH56030	Spain	OP382888		Ge et al. 2021
***M. lavendula***	**FLF1785**	**China**	**PX612289**	**PX684295**	**This study**
***M. lavendula***	**MSX1785**	**China**	**PX612290**	**PX684296**	**This study**
*M. leaiana*	HKAS79900	China	ON791476	**–**	Xiao et al. 2025
*M. leaiana*	HKAS126400	China	OQ025147	**–**	Xiao et al. 2025
***M. longnanensis***	**FLF1804**	**China**	**PX612291**	**PX684297**	**This study**
***M. longnanensis***	**MSX1804**	**China**	**PX612292**	**PX684298**	**This study**
*M. luceata*	ACP2116	Mexico	OR233614	OR233755	Fan et al. 2024
*M. luceata*	ACP2126	Mexico	OR233613	OR233754	Fan et al. 2024
*M. luciferina*	ACP2114	Mexico	OR233612	–	Fan et al. 2024
*M. lucisnieblae*	ACP2140	Mexico	OR233610	OR233752	Fan et al. 2024
*M. lucisnieblae*	ACP2139	Mexico	OR233611	OR233753	Fan et al. 2024
*M. luxmanantlan*	ACP2160	Mexico	OR233603	OR233747	Fan et al. 2024
*M. luxmanantlan*	ACP2159	Mexico	OR233604	OR233748	Fan et al. 2024
*M. maculata*	HMJAU 43111	China	MK309792	–	Xiao et al. 2025
*M. maculata*	HMJAU 43009	China	MK309791	–	Xiao et al. 2025
*M. metata*	HMJAU43625	China	MH396636	**–**	Xiao et al. 2025
*M. metata*	HMJAU43680	China	MH396637	**–**	Unpublished
***M. minispora***	**FLF1223**	**Chin**a	**PX612293**	**–**	**This study**
***M. minispora***	**FLF1244**	**China**	**PX612294**	**PX684299**	**This study**
*M. miscanthi*	HMJAU43573	China	MK309777	**–**	Na and Bau 2019
*M. miscanthi*	HMJAU43582	China	MK309778	**–**	Na and Bau 2019
***M. oblongispora***	**FLF1861**	**China**	**PX612295**	**PX684300**	**This study**
***M. oblongispora***	**MSX1861**	**China**	**PX612296**	**PX684301**	**This study**
***M. pakistanica***	**KB16**	**Pakistan**	**PX612297**	**–**	**This study**
*M. pearsoniana*	CBH068	Germany	FN394614	KF723645	Fan et al. 2024
*M. pearsoniana*	LK880/2002	Germany	FN394613	KF723647	Fan et al. 2024
*M. pelianthina*	CBH015	Denmark	FN394549	KF723649	Fan et al. 2024
*M. pelianthina*	CBH016	Denmark	FN394547	KF723650	Fan et al. 2024
*M. picta*	iNat64022635	USA	OP345850	**–**	Xiao et al. 2025
*M. picta*	TUR194167	Hungary	MW540717	**–**	Xiao et al. 2025
*M. pluteoides*	HMJAU43765	China	MK733306	**–**	Xiao et al. 2025
*M. pluteoides*	HMJAU43771	China	MK733307	**–**	Xiao et al. 2025
*M. polycystidiata*	FFAAS0417	China	ON427731	ON468469	Fan et al. 2024
*M. polycystidiata*	FFAAS0418	China	ON427732	ON468470	Fan et al. 2024
*M. polygramma*	H6039079	Hungary	MW540711	**–**	Unpublished
*M. polygramma*	H6039058	Hungary	MW540703	**–**	Unpublished
***M. polyspora***	**FLF744**	**Chin**a	**PX612298**	**PX684302**	**This study**
***M. polyspora***	**MSX744**	**China**	**PX612299**	**PX684303**	**This study**
*M. purpureofusca*	oka284	Turkey	ON707205	**–**	Kaygusuz 2022
*M. purpureofusca*	oka283	Turkey	ON707204	**–**	Kaygusuz 2022
***M. qilianensis***	**FLF1269**	**China**	**PX612300**	**PX684304**	**This study**
***M. qilianensis***	**FLF1582**	**China**	**PX612301**	**–**	**This study**
*M. rosea*	CBH097	Denmark	FN394556	KF723635	Fan et al. 2024
*M. rosea*	CBH409	Denmark	FN394551	KF723637	Fan et al. 2024
*M. rufobrunnea*	FFAAS0415	China	ON427729	ON468467	Fan et al. 2024
*M. rufobrunnea*	FFAAS0416	China	ON427730	ON468468	Fan et al. 2024
*M. sanguinolenta*	CBHHK098	Norway	MT153147	–	Thoen et al. 2020
*M. sanguinolenta*	ASIS21424	Korea	KF668294	–	Unpublished
*M. seminau*	ACL136	Malaysia	KF537250	–	Fan et al. 2024
*M. seminau*	ACL308	Malaysia	KF537252	–	Fan et al. 2024
*M. semivestipes*	HMJAU43825	China	MK733308	**–**	Wei et al. 2024
*M. semivestipes*	HMJAU43830	China	MK733309	**–**	Wei et al. 2024
*M. shengshanensis*	FFAAS0424	China	ON427739	ON468477	Fan et al. 2024
*M. shengshanensis*	FFAAS0425	China	ON427740	ON468478	Fan et al. 2024
*M. silvae-nigrae*	515	USA	JF908452		Ge et al. 2021
*M. silvae-nigrae*	CC13-12	USA	KF359604		Ge et al. 2021
*M. sinar*	ACL092	Malaysia	KF537247	–	Fan et al. 2024
*M. sinar*	ACL135	Malaysia	KF537249	–	Fan et al. 2024
*M. sinar*	ACL307	Malaysia	KF537251	–	Fan et al. 2024
*M. sophiae*	ACP2157	Mexico	OR233606	OR233749	Fan et al. 2024
*M. sophiae*	ACP2161	Mexico	OR233605	OR233757	Fan et al. 2024
***M. squamulosa***	**FLF1971**	**China**	**PX612302**	**PX684305**	**This study**
***M. squamulosa***	**MSX1971**	**China**	**PX612303**	**PX684306**	**This study**
*M. stipata*	AH57256	Spain	PP868150		Unpublished
*M. strobilinoidea*	NX0647	China	MG654743	**–**	Zhang et al. 2024
*M. strobilinoidea*	NX0648	China	MG654744	**–**	Zhang et al. 2024
*M. stylobates*	JS1511302	China	MH400929	**–**	Sandoval et al. 2025
*M. stylobates*	JS151130	China	MH400928	**–**	Sandoval et al. 2025
***M. subfloridula***	**FLF1713**	**China**	**PX612304**	**PX684307**	**This study**
***M. subfloridula***	**MSX1713**	**China**	**PX612305**	**PX684308**	**This study**
*M. substylobates*	NX0574	China	MH216190	**–**	Na and Bau 2019
*M. substylobates*	NX0571	China	MH216189	**–**	Na and Bau 2019
*M. subulata*	FFAAS0419	China	ON427735	ON468473	Fan et al. 2024
*M. subulata*	FFAAS0423	China	ON427737	ON468475	Fan et al. 2024
*M. tenerrima*	HMJAU43646	China	MK309795	**–**	Na and Bau 2019
*M. tenerrima*	HMJAU43816	China	MK309796	**–**	Na and Bau 2019
***M. tephroleuca***	**FLF1816**	**China**	**PX612306**	**PX684309**	**This study**
***M. tephroleuca***	**MSX1816**	**China**	**PX612307**	**PX684310**	**This study**
*M. yuezhuoi*	FFAAS0344	China	MW581490	MW882249	Fan et al. 2024
*M. yuezhuoi*	FFAAS0347	China	MW581493	MW882252	Fan et al. 2024
*M. zephirus*	HMJAU 43106	China	MT497550	–	Na and Bau 2020
*M. zephirus*	KA13-1265	Korea	KR673722	–	Na and Bau 2020
*P. speirea*	PRM:922296	Czech	LT671445	–	Na and Bau 2020
*P. speirea*	PRM 860810	Czech	LT671446		Na and Bau 2020
*Xeromphalina campanella*	TFB7283A	Sweden	KM024575	–	Na et al. 2022
*Xeromphalina campanella*	TFB14487	Czech	KP835678	–	Na et al. 2022

**Figure 1.**
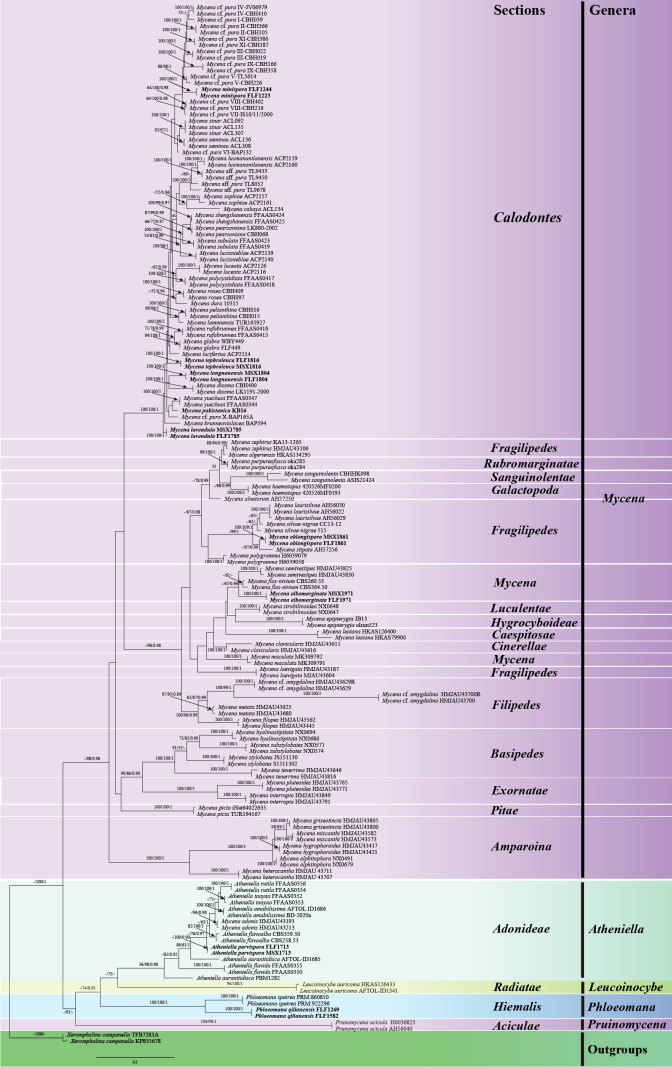
Phylogenetic consensus tree inferred from the ML analysis based on the concatenated ITS and *tef1-α* dataset. The tree is rooted with *Xeromphalina campanella*. Studied genera are indicated in different colors, and sequences generated in this study are highlighted in bold.

Phylogenetic analyses using maximum likelihood (ML), maximum parsimony (MP), and Bayesian inference (BI) were conducted through the CIPRES Science Gateway v. 3.3 (Miller et al. 2012). ML analyses were performed with RAxML-HPC BlackBox 8.2.6, MP analyses with PAUP on XSEDE (v. 4.a165), and BI analyses with MrBayes on XSEDE 3.2.6. All characters were equally weighted, and gaps were treated as missing data. Tree searches employed heuristic methods with tree bisection and reconnection (TBR) branch swapping and 1,000 random sequence additions. For Bayesian analyses, four Markov chains were run in two independent runs, starting from random trees, for 2 million generations. Trees were sampled every 100 generations, and runs were continued until the average standard deviation of split frequencies fell below 0.01. Phylogenetic trees were visualized in FigTree v. 1.4.2 (Rambaut 2012) and further edited in Adobe Illustrator CS6 (Guide 2012). Branches receiving bootstrap support ≥50% in MP (BP) and ML (BS) analyses, and Bayesian posterior probabilities (BPP) ≥0.90, were considered significantly supported. The MP, ML, and BI analyses produced largely congruent topologies; therefore, only the ML tree is presented (Fig. 1), with support values shown in the order BP/BS/BPP. The final concatenated ITS + *tef1-α* alignments were deposited in TreeBase (https://treebase.org/treebase-web/home.html, submission ID XXXX), and the taxonomic novelties were registered in MycoBank (http://www.MycoBank.org).

## Results

### Molecular phylogenetic analyses

The ITS + *tef1-α* dataset involved in the phylogenetic analysis comprised 152 taxa, including 140 taxa of *Mycena*, eight taxa of *Atheniella*, and two taxa of *Phloeomana*. The aligned matrix contained 1,474 characters, including gaps, of which 537 characters were constant, 35 were variable but parsimony-uninformative, and 902 were parsimony-informative. MP analysis yielded 11 equally parsimonious trees (TL = 3,779, CI = 0.439, RI = 0.812, RC = 0.356, HI = 0.561). The best-fit substitution model selected for the ITS + *tef1-α* dataset and applied in the BI analysis was GTR + I + G. BI and ML analyses generated topologies similar to the MP analysis, with an average standard deviation of split frequencies of 0.009845 (BI). The best ML tree, including BP, BS, and BPP support values, is shown in Fig. 1. The resulting phylogeny shows that the 10 newly described species each form distinct, well-supported lineages separated from *Atheniella*, *Mycena*, and *Phloeomana* (Fig. 1).

### Taxonomy

#### 1. Atheniella parvispora

Taxon classificationFungiAgaricalesMycenaceae

S. X. Ma, A. Naseer, A. N. Khalid & L. F. Fan sp. nov.

9FB85940-DD08-53AF-AB1D-D0DD3B2B48C7

860938

Figs 2, 3

##### Etymology.

The specific epithet **“parvispora”** refers to small size of basidiospores.

**Figure 2.**
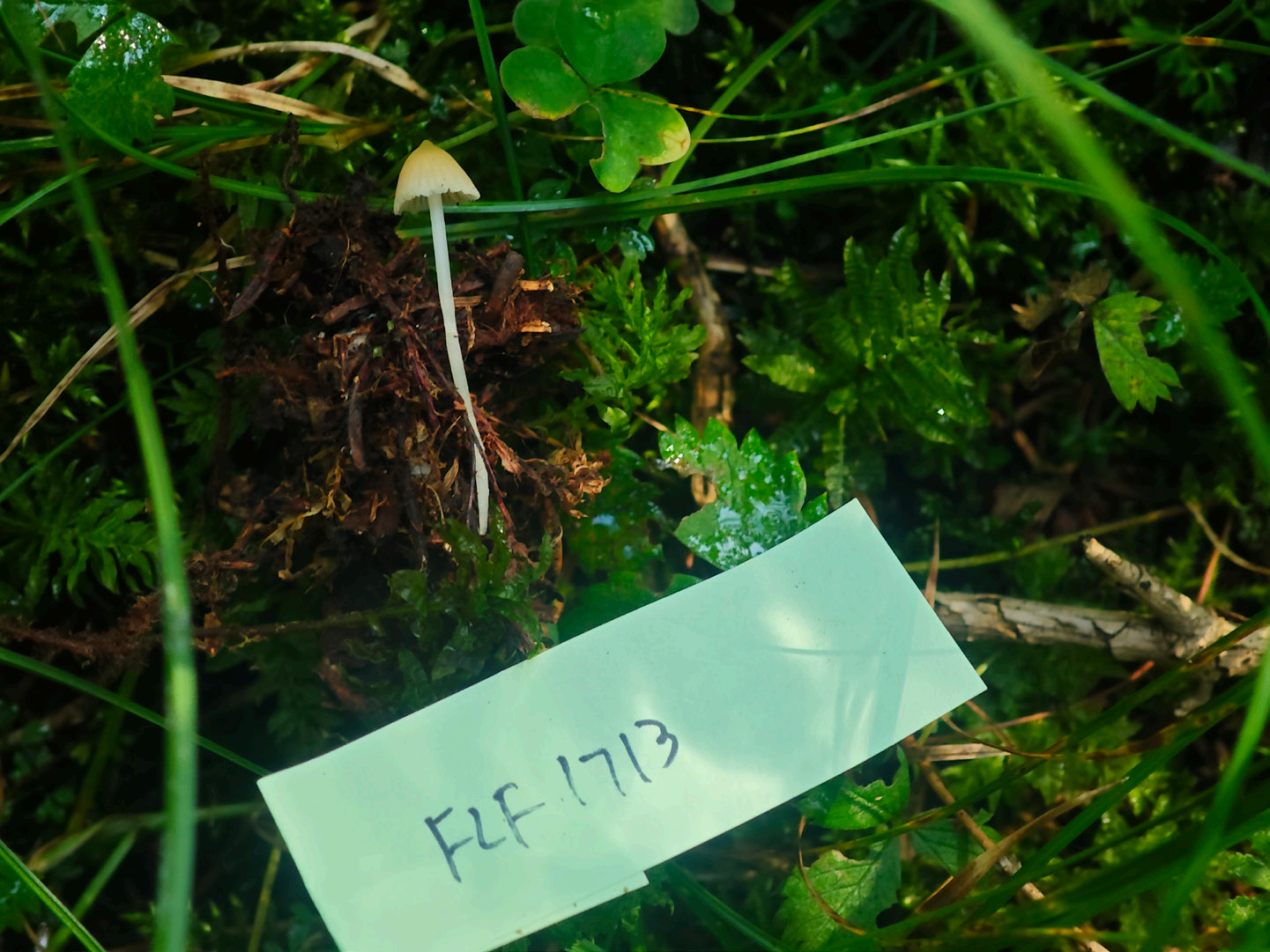
Morphological features of *Atheniella parvispora*, sp. nov., showing the pileus and stipe. Scale bar: 1 cm.

**Figure 3.**
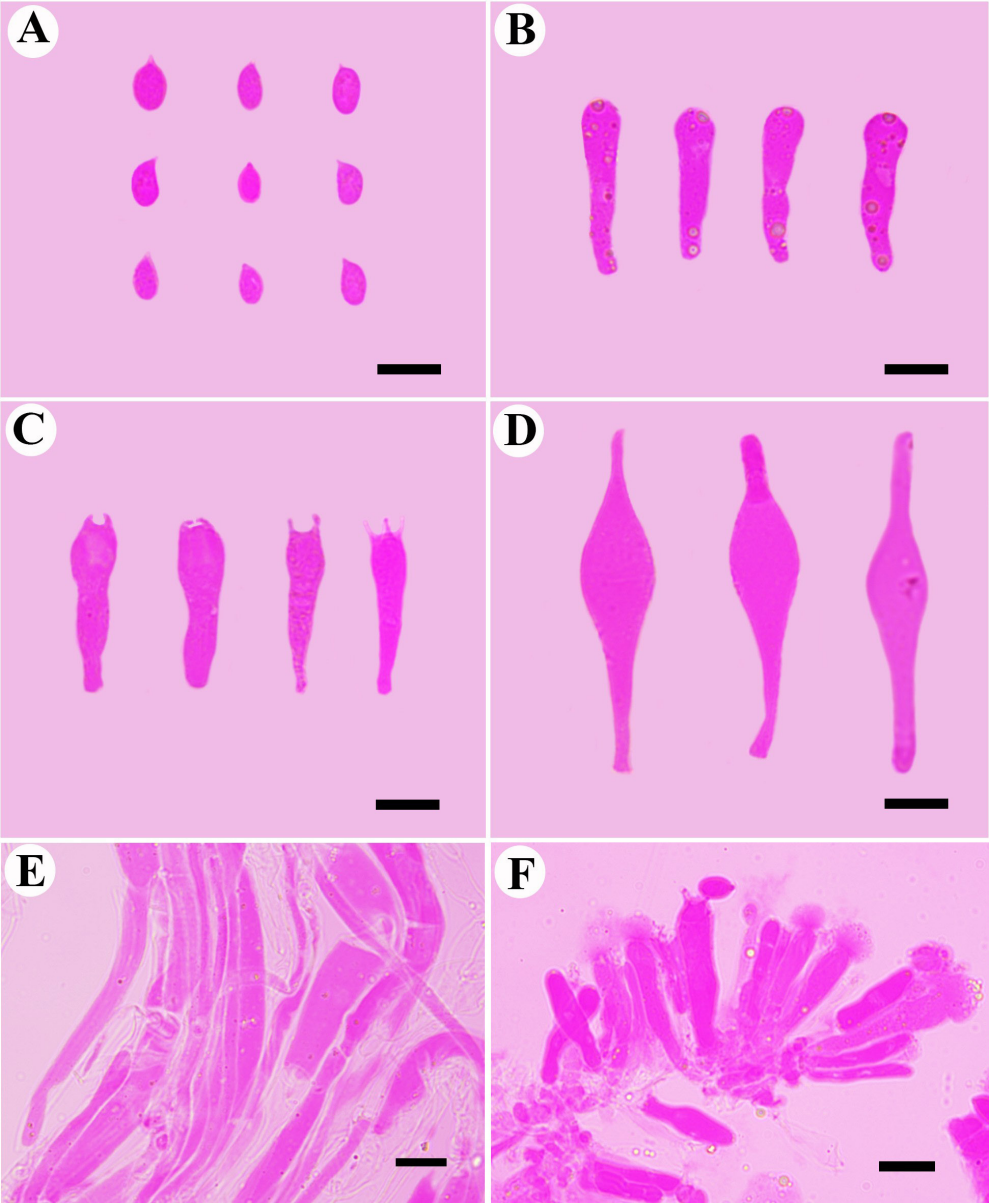
Anatomical characters of *Atheniella parvispora*, sp. nov. **A** Basidiospores, **B** probasidia, **C** basidia, **D** cheilocystidia, **E** hyphae of the pileipellis, **F** hymenium. Scale bar: 10 μm.

##### Diagnosis.

The species is characterized by papillate, umbilicate, orangish yellow pileus with off white margins, lacrymoid, ovoid to subcylindrical, small basidiospores (6.71 × 3.70 µm) and abundant fusiform cheilocystidia.

##### Holotype.

**CHINA**, Gansu Province, Gannan Tibetan Autonomous Prefecture, Lintan County, Lianhuashan National Forest Park, 34°57'N, 103°30'E, on broad-leaved forests land, 2 September 2024, FLF1713 (GenBank ITS = PX612304; *tef1-α* = PX684307)

##### Description.

***Basidiomata*** small-sized. ***Pileus*** 1.0–1.3 cm in diameter, orangish yellow (2.5R 8/4), margins off white (10R 8/1) in circular ring, papillate, umbilicate, dry, smooth at center, fibrillose towards margins, plicate to split, margin decurved to incurved. ***Context*** thin, white. ***Lamellae*** adnate with decurrent tooth, chalky white (10R 8/1), narrow, subdistant, thin, unequal, even. ***Lamellulae***, in two tiers, alternating with lamellae. ***Stipe*** ca. 5.3 cm, chalky white (10R 8/1) to smoky white (2.5YR 8/1), central, equal to slightly flexuous at base, surface scurfy to canescent. ***Hyphal system*** composed of generative clamped hyphae, IKI–, inflated in KOH, thin-walled, occasionally branched, subregular to irregularly arranged, 2.74–12.24 µm in diameter.

***Basidiospores*** 5.32–8.19 × 2.91–4.91 µm, av. *L* × av. *W* = 6.71 × 3.70 µm, *Q* = 1.75–1.81, lacrymoid, ovoid to subcylindrical with an apiculus and oil drop, smooth, hyaline in 5% KOH, IKI–, guttulated, thin-walled. ***Probasidia*** cylindrical to clavate, thin-walled. ***Basidia*** 22.56–26.53 × 5.58–7.32 µm, av. *L* × av. *W* = 24.82 × 6.34 µm, *Q* = 3.91, clavate to narrowly clavate, guttulated, hyaline, thin-walled, bi to tetra-sterigmated. ***Cheilocystidia*** 46.44–55.01 × 8.81–12.30 µm, av. *L* × av. *W* = 51.45 × 9.88 µm, abundant, fusiform, ventricose-rostrate, with an obtuse apex, short- to long-stalked, smooth, hyaline in 5% KOH, thin–walled. ***Pleurocystidia*** not observed. ***Pileipellis*** a cutis of inflated, clavate, hyphae, hyphae 10.53–20.11 µm in diameter, thin-walled, with branched excrescences.

##### Additional specimen (isotype) examined.

**CHINA**, Gansu Province, Gannan Tibetan Autonomous Prefecture, Lintan County, Lianhuashan National Forest Park, 34°57'N, 103°30'E on broad-leaved forests land, 2 September 2024, MSX1713 (GenBank ITS = PX612305; *tef1-α* = PX684308).

##### Notes.

The presence of a brightly colored pileus, an inamyloid hymenophoral trama, and pileipellis covered with simple to branched excrescences suggests that this species belongs to the genus *Atheniella*. It is characterized by small basidiomata, a pileus with white margins to orangish yellow, thin and white context, a chalky white to smoky white hollow stipe with a scurfy to canescent surface, thin-walled and clamped hyphae inflated in KOH, lacrymoid, ovoid to subcylindrical, small basidiospores (6.71 × 3.70 μm) with an apiculus and oil drop, and abundant fusiform cheilocystidia.

In the combined ITS + *tef1-α* phylogram, *Atheniella parvispora* is separated from *M. floridula* (Fr.) Quél. and *A. flavoalba* (Fr.) Redhead, Moncalvo, Vilgalys, Desjardin & B.A. Perry. The latter two species were formerly classified in *Mycena* sect. *Adonideae*. Redhead (2012) proposed that *M. floridula* was a synonym of *A. adonis* (Bull.) Redhead, Moncalvo, Vilgalys, Desjardin & B.A. Perry; however, more recently, *M. floridula sensu* Kühner has been considered a pink-capped form of *M. flavoalba* (Kühner and Maire 1938; Aronsen and Læssøe 2016).

In the phylogeny, the new species forms a sister group to *Atheniella adonis* (previously named *M. floridula*). Although morphologically similar in terms of basidiospores, *A. parvispora* can be differentiated from *A. adonis* by its papillate, umbilicate, and plicate pileus with a circular ring at the margin, as well as its scurfy to canescent stipe. Anatomically, *A. adonis* possesses pseudofusiform or lageniform pleurocystidia with a distended venter. Furthermore, *A. parvispora* has smaller basidiospores (6.71 × 3.70 μm) than the larger ones of *A. adonis* (7–9.5 × 3.5–5 μm). The close relative is *A. flavoalba*, which is the most widely distributed species in the Northern Hemisphere and can be easily differentiated by its narrowly ellipsoid basidiospores and larger caulocystidia (up to 60 μm) (Aronsen and Læssøe 2016; Na and Bau 2019).

#### 2. Mycena albomarginata

Taxon classificationFungiAgaricalesMycenaceae

S. X. Ma, A. Naseer, A. N. Khalid & L. F. Fan sp. nov.

95C0829A-2CEA-542D-BE4C-A8AA8936E3C8

860899

Figs 4, 5

##### Etymology.

The specific epithet “albomarginata” refers to the white margins of pileus.

**Figure 4.**
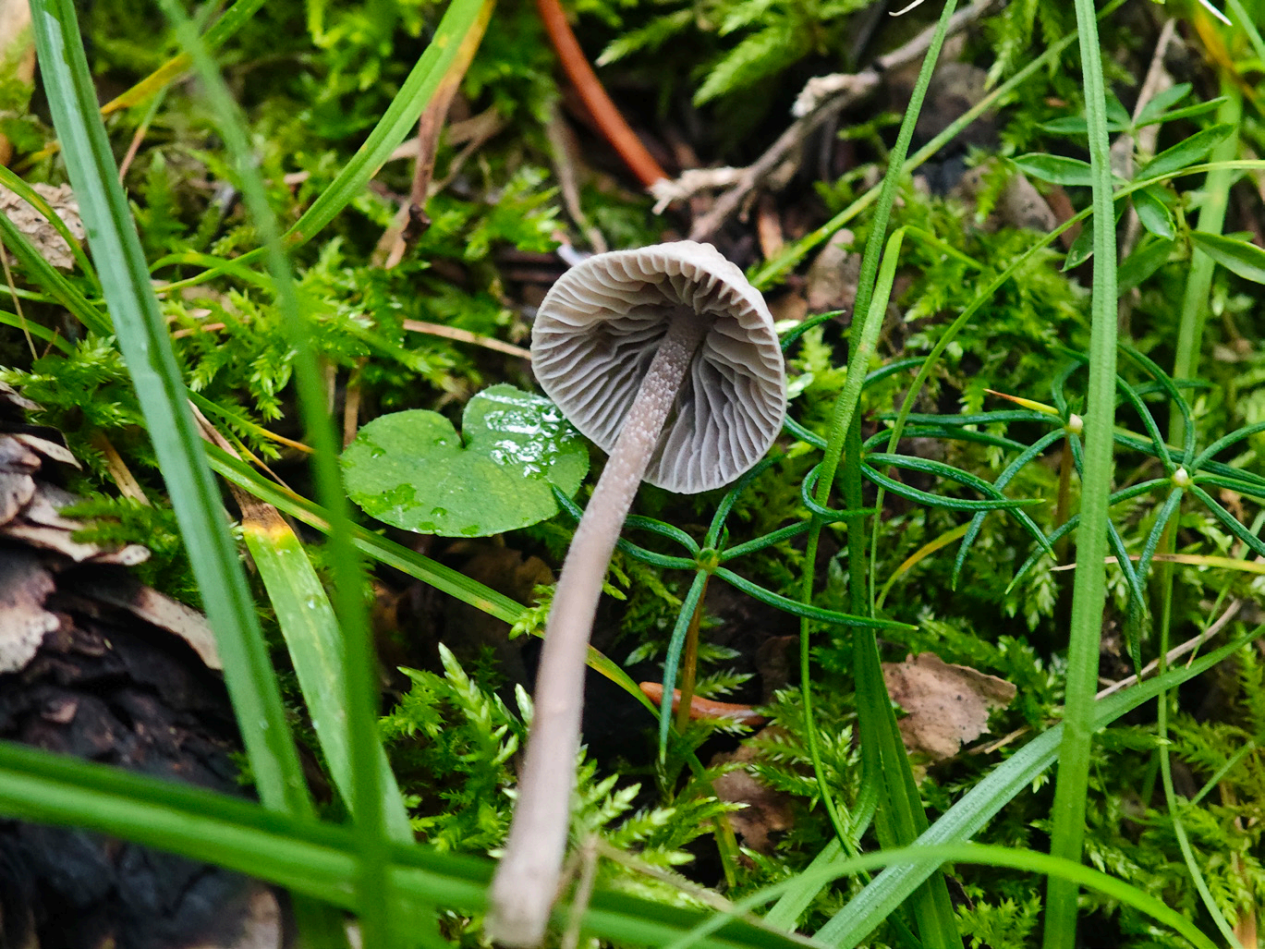
Morphological features of *Mycena albomarginata*, showing the stipe, pileus, and lamellae. Scale bars: 1 cm.

**Figure 5.**
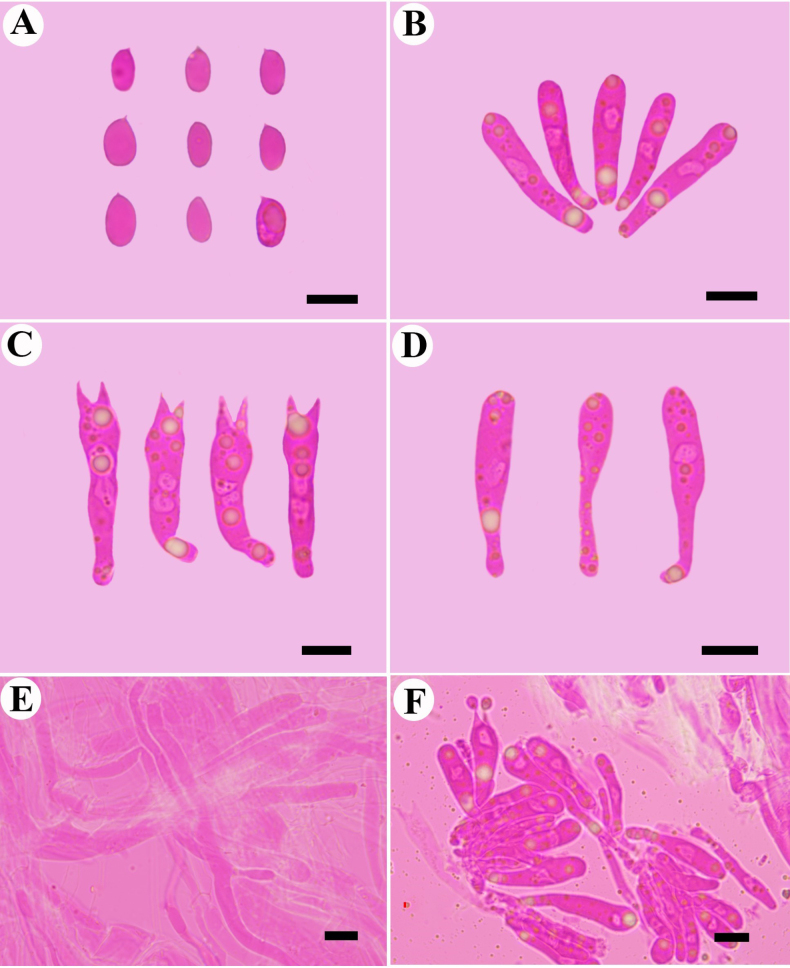
Anatomical characters of *Mycena albomarginata*. **A** Basidiospores, **B** probasidia, **C** basidia, **D** cheilocystidia, **E** hyphae of the pileipellis, **F** hymenium. Scale bar: 10 μm.

##### Diagnosis.

The species is characterized by virgate to sulcate, brownish gray pileus with white margins, brownish gray stipe with pruinose apex, free to adnexed lamellae, narrowly clavate cheilocystidia with slightly long base, larger (9.08 × 5.51 µm), ellipsoid basidiospores, and bi-sterigmated basidia.

##### Holotype.

**CHINA**, Gansu Province, Tianzhu Tibetan Autonomous County, Xidatan Town, 37°28'N, 103°16'E, in mixed coniferous, broadleaf forest, 19^th^ September 2024, FLF1971 (GenBank ITS = PX612302; *tef1-α* = PX684305).

##### Description.

***Basidiomata*** small-sized. ***Pileus*** 1.9–2.2 cm in diameter, pigeon gray (N 9/0) to gray (7.5Y 5/1), brown, margins white (6.9GY 7/1), in circular ring, conical to parabolic, dry, smooth in center, glabrous, virgate to sulcate, margins smooth, even, entire, regular, slightly recurved to straight. ***Context*** thin, gray. ***Lamellae*** regular, free to adnexed, fimbriate, gray (7.5Y 6/1), broad, subdistant, thick, wavy. ***Lamellulae*** of two different size, in two tiers, alternating with lamellae. ***Stipe*** approximately 4.5 cm long, brownish gray (10Y 6/1) with white pruinose apex, upper 3^rd^ part of stipe is white, central, cylindrical to flexuous, only apex floccose, smooth towards base, base white and slightly bulbous. ***Hyphal system*** composed of hyphae without clamp connections, dextronoid, inflated in KOH, thin-walled, branched, septate, regular to subregular arrangement, 4.84–8.29 µm in diameter.

***Basidiospores*** 7.56–10.43 × 5.5–6.90 µm, av. *L* × av. *W* = 9.08 × 5.51 µm, *Q* = 1.54–1.66, broadly ellipsoid with apiculus and oil drop, smooth, hyaline in 5% KOH, amiloid, thin-walled. ***Probasidia*** cylindrical to clavate, thin-walled. ***Basidia*** 24.49–37.44 × 6.18–8.67 µm, av. *L* × av. *W* = 32.30 × 7.39 µm, *Q* = 4.25–4.36, clavate, guttulated, hyaline, thin-walled, two-spored. ***Cheilocystidia*** 27.84–33.63 × 5.28–6.67 µm, av. *L* × av. *W* = 31.84 × 5.99 µm, abundant, narrowly clavate with slightly long base, smooth, hyaline in 5% KOH, thin-walled, in groups. ***Pileipellis*** a cutis; hyphae 11.4–20.1 µm in diam., inflated, thin-walled, predominantly smooth but with scattered excrescences.

##### Additional specimens examined.

**CHINA**, Gansu Province, Tianzhu Tibetan Autonomous County, Xidatan Town, 37°25'N, 102°58'E, in mixed coniferous-broadleaf forests, 29 September 2024, MSX1971 (GenBank ITS = PX612303; *tef1-α* = PX684306).

##### Notes.

*Mycena albomarginata* belongs to *Mycena* sect. *Mycena*. The species is characterized by a virgate to sulcate, brownish-gray pileus with white margins, a brownish-gray stipe with a pruinose apex, free to adnexed gray lamellae, narrowly clavate cheilocystidia with a slightly elongated base, larger, ellipsoid basidiospores (9.08 × 5.51 μm), and 2^-st^erigmated basidia. In the phylogenetic analysis (Fig. 1), *M. albomarginata* forms a sister group with *M. semivestipes* (Peck) A.H. Sm., sharing similar pileus and stipe morphology as well as spore shape. However, *M. albomarginata* is distinguished by a smaller (2.2 cm), brownish-gray pileus with white margins and gray lamellae, whereas *M. semivestipes* possesses a dark brown to nearly black pileus with darker margins and white to pink lamellae. Anatomically, *M. albomarginata* has larger basidiospores (9.08 × 5.51 μm) and larger, 2-spored basidia (24.5–37.4 × 6.2–8.7 μm), whereas *M. semivestipes* has smaller, 4-spored basidia (21–30 × 4.5–5.4 μm) (Bessette et al. 1995).

Another close relative is *M. flos-nivium* Kühner, which can be easily distinguished in the field by the presence of a stipe base densely and conspicuously covered with long, coarse, flexuous, whitish fibrils. Both species share similarities in pileus morphology and coloration, exhibiting very dark sepia brown to dark brown tones that become paler toward the margins. *Mycena albomarginata* has larger basidiospores (9.08 × 5.51 μm) and smaller, smooth, narrowly clavate cheilocystidia (27.8–33.6 × 5.3–6.7 μm) with a slightly elongated base. In contrast, *M. flos-nivium* possesses smaller basidiospores (8–11.5 × 4–5 μm) and larger cheilocystidia (20–80 × 6–12 μm) that are clavate, with some very long-stalked and covered with fairly few, unevenly spaced, usually rather coarse, simple to branched, straight to curved excrescences (Na and Bau 2020).

#### 3. Mycena oblongispora

Taxon classificationFungiAgaricalesMycenaceae

S. X. Ma, A. Naseer, A. N. Khalid & L. F. Fan sp. nov.

78D64CFC-46F8-56D2-B8C1-72101EC001AB

860937

Figs 6, 7

##### Etymology.

The specific epithet “oblongispora” refers to the oblong shape of spores.

**Figure 6.**
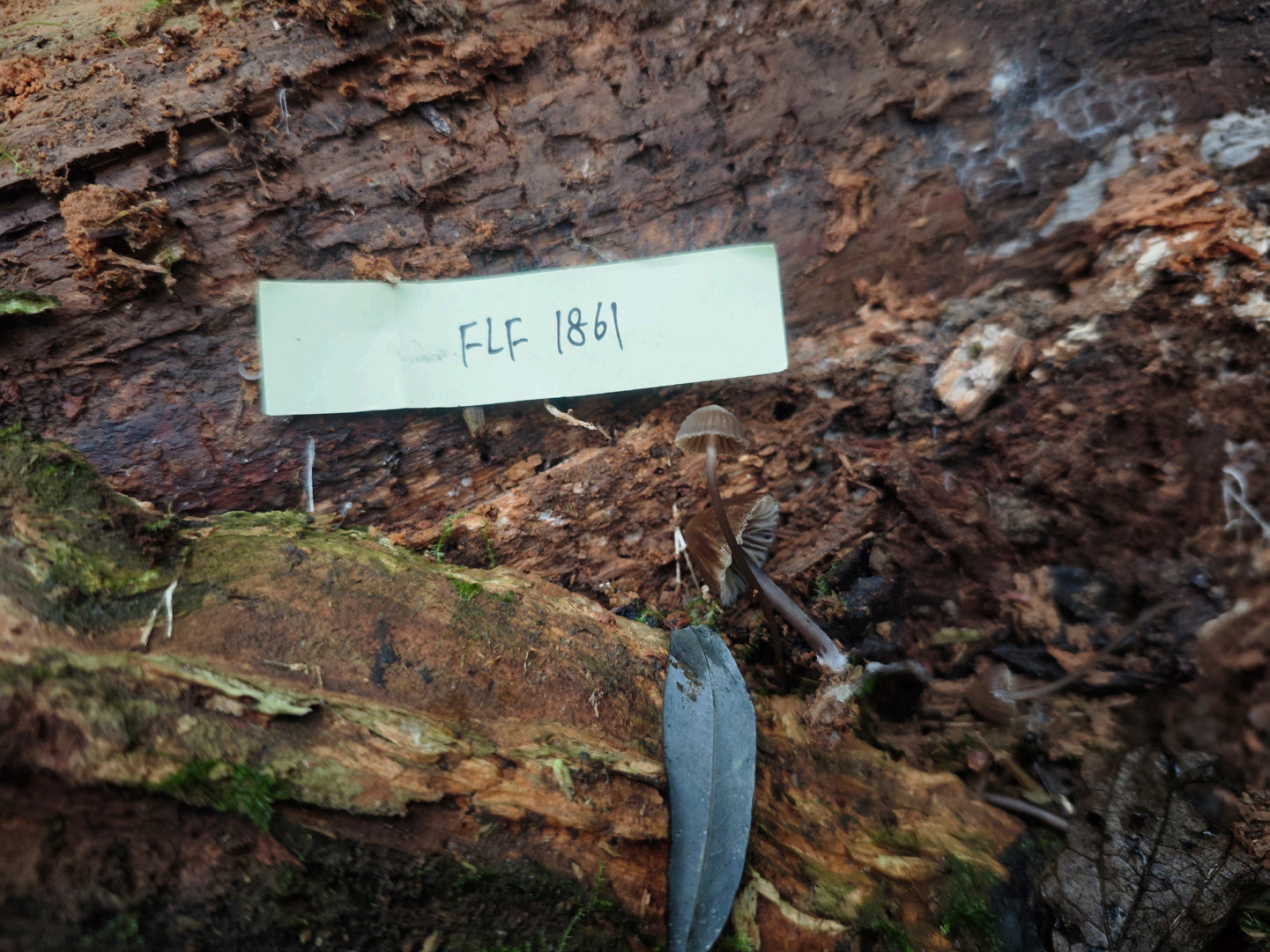
Morphological features of *Mycena oblongispora*, sp. nov., showing the pileus, stipe, and lamellae. Scale bar: 1 cm.

**Figure 7.**
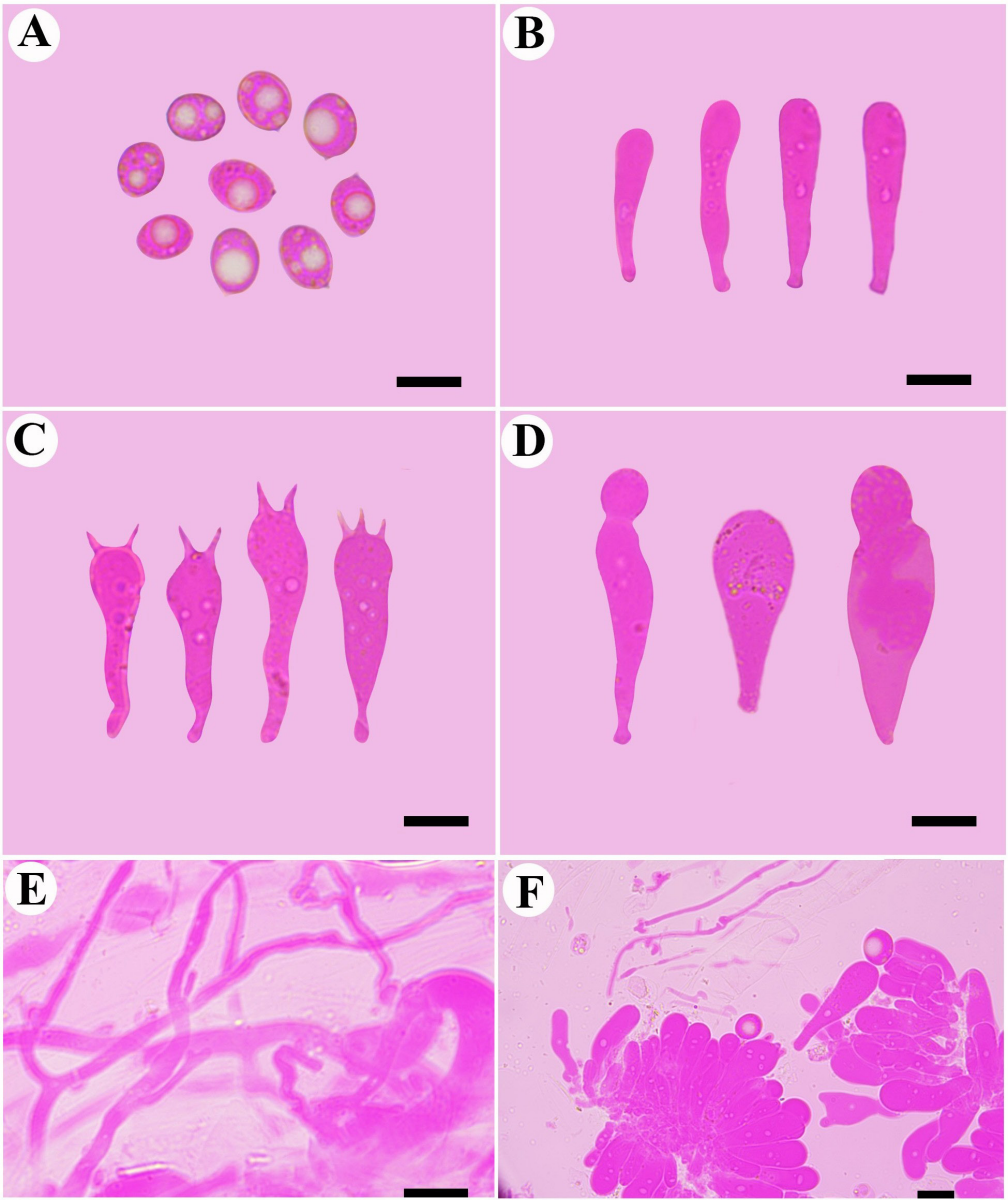
Anatomical characters of *Mycena oblongispora*, sp. nov. **A** Basidiospores, **B** probasidia, **C** basidia, **D** cheilocystidia, **E** hyphae of the pileipellis, **F** hymenium. Scale bar: 10 μm.

##### Diagnosis.

It is characterized by grayish brown pileus having brownish black fibrils, gray, free to remote lamellae, with rhizomorphs at the base stipe, growing on decaying logs, smaller (7.25 × 4.96 µm), subglobose to ellipsoid to oblong basidiospores, smooth, smaller (35.27 × 12.40 µm) cheliocystidia and with scattered excrescences in pileipellis.

##### Holotype.

**CHINA**, Gansu Province, Longnan City, Guan’e gully national forest park, 33°9'N, 104°2'E, grow on decaying logs in mixed coniferous-broadleaf forests, 5 September 2024, FLF1861 (GenBank ITS = PX612295; *tef1-α* = PX684300).

##### Description.

***Basidiomata*** small-sized. ***Pileus*** 1.2–1.9 cm in diameter, with brownish black (10YR 2/2) fibrils on a grayish brown (10YR 4/2) context, parabolic, dry, sulcate to plicate, striated, striation expands towards margins, margins slightly uplifted to straight, even, entire. ***Context*** thin, white to grayish brown (10YR 4/2). ***Lamellae*** free to remote, even, entire, gray (2.5GY 7/1), broad, subdistant, average, unequal to forked. ***Lamellulae*** regular, of two different sizes, alternating with lamellae, in two tiers. ***Stipe*** 2.5–3.9 cm long, central, cylindrical to flexuous, smooth to virgate with maturity, apex gray (2.5GY 7/1), becoming black (10YR 3/2) to dark brown (10YR 2/3) towards the base whitish (10R 8/1) with distinct rhizomorphs. ***Hyphal system*** composed of generative hyphae with clamp connections, dextrinoid, inflated in KOH, thin-walled, branched, regultar to subregularly arranged, 1.94–6.32 µm in diameter.

***Basidiospores*** 5.94–8.94 × 3.38–7.06 µm, av. *L* × av. *W* = 7.25 × 4.96 µm, *Q* = 1.34–1.46, subglobose to oblong ellipsoid or slightly ovoid with an apiculus and oil drop, smooth, hyaline in 5% KOH, amyloid, thin-walled. ***Probasidia*** cylindrical to clavate, elongated and slender at the base, thin-walled. ***Basidia*** 23.98–34.59 × 5.78–9.88 µm, av. *L* × av. *W* = 28.83 × 8.76 µm, *Q* = 2.28, clavate, guttulated, hyaline, thin-walled, two to four spored. ***Cheilocystidia*** 29.60–43.74 × 8.46–14.87 µm, av. *L* × av. *W* = 35.27 × 12.40 µm, abundant, clavate with capitate apex, smooth, hyaline in 5% KOH, thin-walled. ***Pleurocystidia*** not observed. *Hyphae of the pileipellis* 5.87–16.77 µm, inflated, thin-walled, with scattered excrescences.

##### Additional specimen (isotype) examined.

**CHINA**. Gansu Province, Longnan City, Guan’e gully national forest park, 33°93'N, 104°2'E, grow on decaying logs in mixed coniferous-broadleaf forests, 5 September 2024, MSX1861. (GenBank ITS = PX612296; *tef1-α* = PX684301).

##### Notes.

This new species belongs to *Mycena sect. Fragilipedes*. It is characterized by a grayish-brown pileus with brownish-black fibrils, gray, free to remote lamellae, and a stipe base with distinct rhizomorphs. Ecologically, it grows on decaying logs. Microscopically, it is distinguished by its smaller, subglobose to oblong-ellipsoid basidiospores (av. 7.25 × 4.96 μm) and hyphae of the pileipellis with scattered excrescences. *Mycena stipata* Maas Geest. & Schwöbel shares similarities with *M. oblongispora*, as both possess a brownish-beige to blackish-brown pileus. However, *M. oblongispora* can be distinguished by its fibrillose pileus (smooth in *M. stipata*), gray, free to remote lamellae (adnate to adnexed in *M. stipata*), clavate cheilocystidia with a capitate apex (fusiform to lageniform in *M. stipata*), and smaller basidiospores (7.25 × 4.96 μm).

Another close relative, *Mycena silvae-nigrae* Maas Geest. & Schwöbel, shares several similarities, such as a blackish-brown pileus, whitish to grayish lamellae, and the presence of excrescences in the pileipellis. However, *M. oblongispora* has smaller basidiospores (7.25 × 4.96 μm) and smaller cheilocystidia (29.60–43.74 × 8.46–14.87 μm) with a rounded apex, while *M. silvae-nigrae* has larger basidiospores (9.5 × 9 μm) and larger cheilocystidia (20–73 × 7–20 μm) (Osmundson et al. 2013).

#### 4. Mycena lavendula

Taxon classificationFungiAgaricalesMycenaceae

S. X. Ma, A. Naseer, A.N. Khalid & L. F. Fan sp. nov.

8D4BAEF2-AB61-5AED-A66A-9F6F69369DC5

860920

Figs 8, 9

##### Etymology.

The specific epithet “lavendula” refers to the lilac color of basidio­mata.

**Figure 8.**
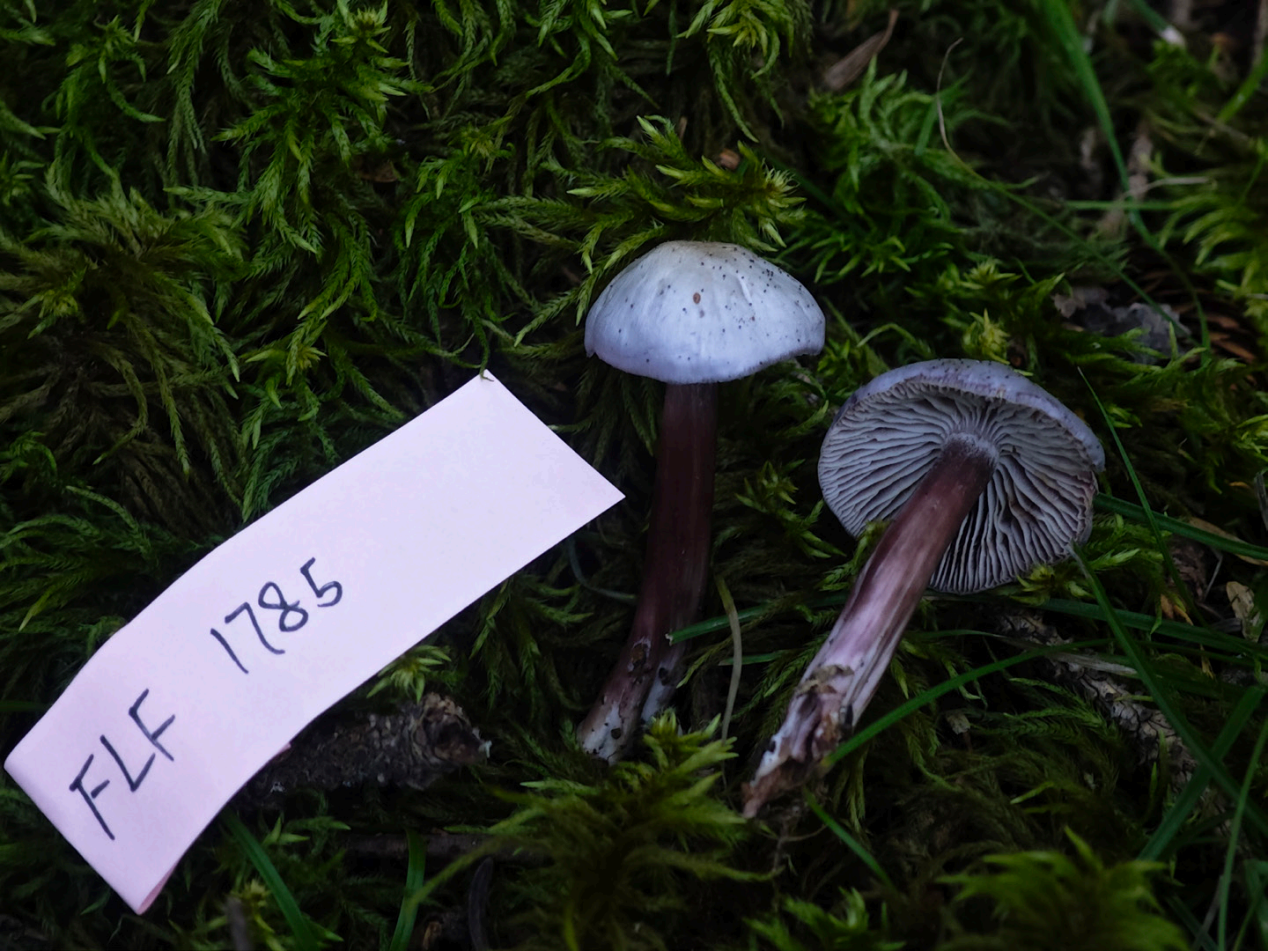
Morphological features of *Mycena lavendula*, sp. nov., showing the pileus, stipe, and lamellae. Scale bar: 1 cm.

**Figure 9.**
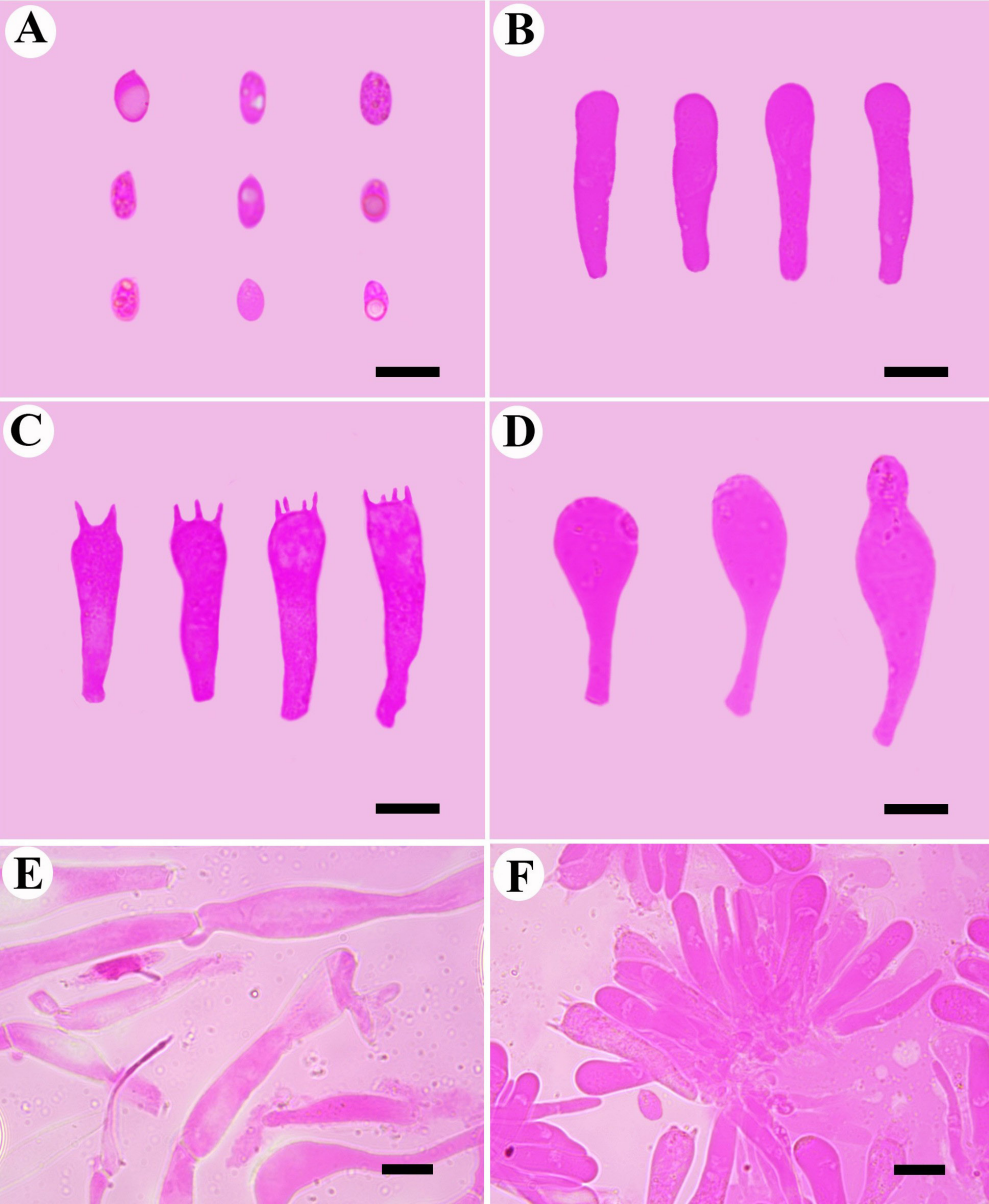
Anatomical characters of *Mycena lavendula*, sp. nov. **A** Basidiospores, **B** probasidia, **C** basidia, **D** cheilocystidia, **E** hyphae of the pileipellis, **F** hymenium. Scale bar: 10 μm.

##### Diagnosis.

*Mycena lavendula* is characterized by a subcylindrical to ellipsoid basidiospores (6.68 × 2.56 µm), polymorphic cheilocystidia with rounded apex and generative hyphae with clamp connections.

##### Holotype.

**CHINA**, Gansu Province, Tibetan Autonomous of Gannan, Taohe National Nature Reserve, 34°40'70"N, 103°53'26"E, in mixed coniferous and broadleaf forests, 3^rd^ September 2024 FLF1785 (GenBank ITS = PX612289; *tef1-α* = PX684295)

##### Description.

***Basidiomata*** medium-sized. ***Pileus*** 2.1–2.4 cm in diameter, lilac (10P 8/2), hemispherical to convex, becoming plano-convex, sticky, glabrous to sericeous, margin incurved, with purple (10P 6/10) fibrills, and smooth, entire. ***Context*** thin, lilac. ***Lamellae*** subdecurrent, lilac (10P 8/2), narrow, crowded, thick, edges even, entire to eroded. ***Lamellulae*** abundant, irregular, of varying lengths, in more than two tiers, alternating with lamellae. ***Stipe*** 3.4–3.8 cm long, reddish purple (2.5RP 2/2) at the apex and darker than the base, lilac (10P 8/2) at the base, central, cylindrical to flexuous, slightly pruinose, longitudinally striate, hollow, context lilac (10P 8/2). ***Hyphal system*** composed of generative hyphae with clamp connections, dextrinoid, inflated in KOH, thin-walled, occasionally branched, regular to subregularly arranged, 4.1–10.8 µm in diameter.

***Basidiospores*** 4.95–8.31 × 2.56–5 µm, av. *L* × av. *W* = 6.68 × 2.56 µm, *Q* = 1.76–1.88, subcylindrical to ellipsoid with an apiculus and oil drop, amyloid, smooth, hyaline in 5% KOH, amyloid, thin-walled. ***Probasidia*** subclavate to clavate, thin-walled. ***Basidia*** 22.36–35.12 × 7.64–8.73 µm, av. *L* × av. *W* = 30.60 × 8.05 µm, *Q* = 3.58–3.79, broadly clavate to slightly pyriform, smooth, hyaline in 5% KOH, thin-walled, four-spored. ***Cheilocystidia*** 28.32–44.76 × 8.56–13.75, av. *L* × av. *W* = 35.99 × 11.81 µm, abundant, polymorphic, clavate, fusiform, pyriform, some with a rounded apex, smooth, hyaline in 5% KOH, thin-walled. ***Pleurocystidia*** not observed. ***Pileipellis*** a cutis of irregular and inflated smooth hyphae, thin-walled, 17.32–23.93 µm in diameter.

##### Additional specimen (paratype) examined.

**CHINA**. Gansu Province, Tibetan Autonomous of Gannan, Taohe National Nature Reserve, 34°40'70"N, 103°53'26"E, mossy ground in mixed forests, 4 September 2024, MSX1785. (GenBank ITS = PX612290; *tef1-α* = PX684296).

##### Notes.

Species of *Mycena sect. Calodontes* are easily recognized by the pinkish, reddish, purplish to brownish pileus and large-sized basidiomata. *Mycena lavendula* is characterized by its lilac pileus and reddish-purple stipe that becomes lilac toward the base, is longitudinally striated, and has a pruinose apex. Microscopically, it is distinguished by small, subcylindrical to ellipsoid basidiospores (av. 6.68 × 2.56 μm) with an oil drop, polymorphic cheilocystidia, and clamped generative hyphae.

*Mycena lavendula* resembles *M. luciferina* Cortés-Pérez, Guzm.-Dáv. & Ram.-Cruz., a recently described bioluminescent species from Mexico, in the shape and size of its basidiospores and cheilocystidia with rounded apices. However, *M. lavendula* can be differentiated from *M. luciferina* by its unique pileus and stipe morphology. *Mycena luciferina* has a reddish-pink pileus and white stipe, whereas *M. lavendula* has a lilac pileus and a reddish-purple stipe with a pruinose apex. Anatomically, *M. lavendula* differs from *M. luciferina* by its larger basidia (22.36–35.12 × 7.64–8.73 μm vs. 15.2–27.2 × 4.5–5.6 μm). Furthermore, *M. luciferina* has larger basidiospores (7.4 × 3.8 μm), whereas *M. lavendula* possesses smaller ones (6.68 × 2.56 μm) (Cortés-Pérez et al. 2023). In the phylogenetic tree, *Mycena lavendula* is separated from *M. oblongispora*, another species described in this study, and forms a sister clade with it. *Mycena oblongispora* can be differentiated from *M. lavendula* by its grayish-brown pileus with brownish-black fibrils, clavate cheilocystidia, and larger basidiospores, whereas *M. lavendula* has a lilac pileus, polymorphic cheilocystidia, and smaller basidiospores (6.68 × 2.56 μm vs. 7.25 × 4.96 μm).

#### 5. Mycena longnanensis

Taxon classificationFungiAgaricalesMycenaceae

S. X. Ma, A. Naseer, A. N. Khalid & L. F. Fan sp. nov.

E31A1660-371D-52C4-9AAB-FDEE389D14B0

860921

Figs 10, 11

##### Etymology.

The specific epithet “longnanensis” refers to type locality Longnan.

**Figure 10.**
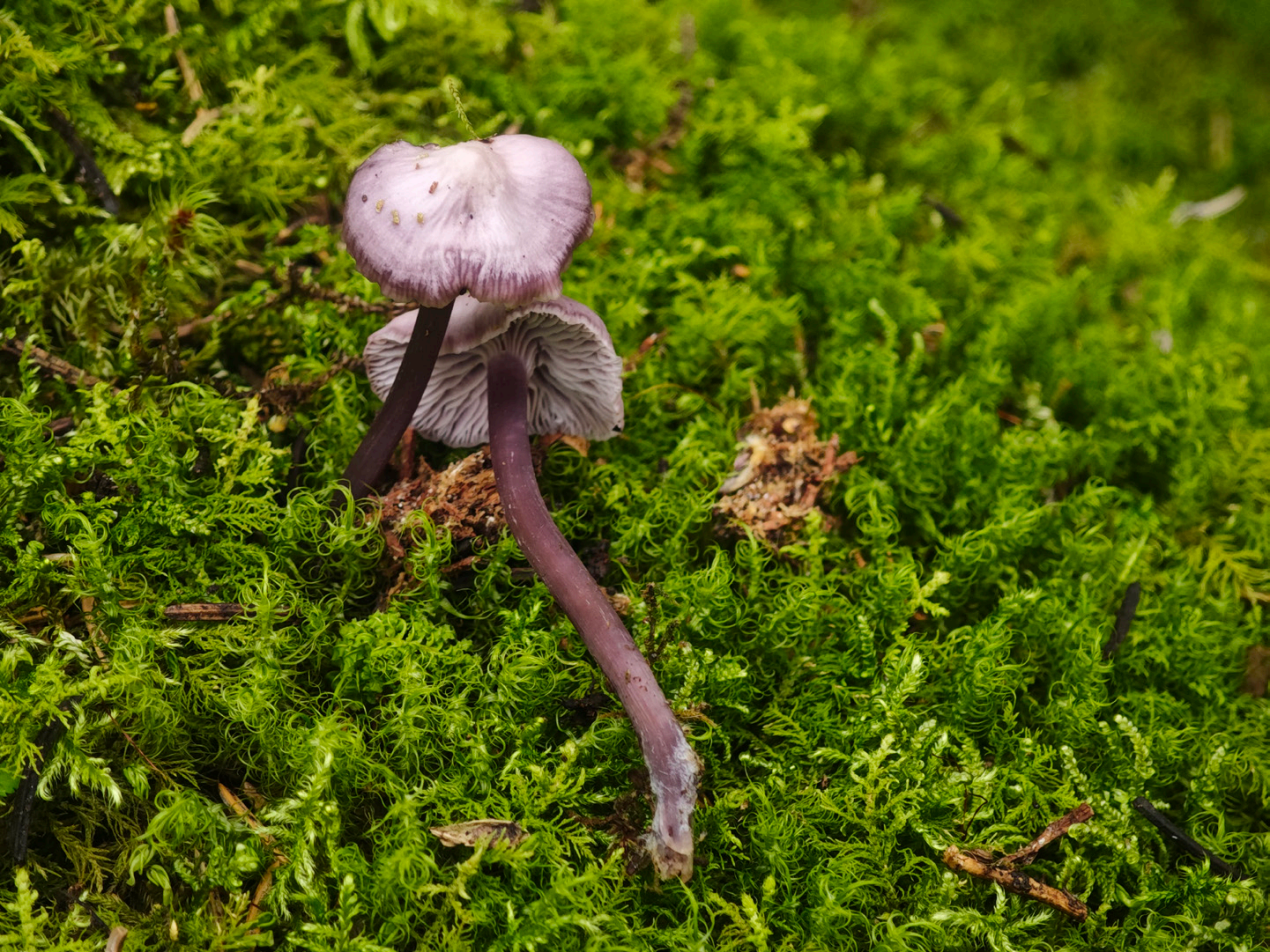
Morphological features of *Mycena longnanensis*, sp. nov., showing the stipe, pileus, and lamellae. Scale bar: 1 cm.

**Figure 11.**
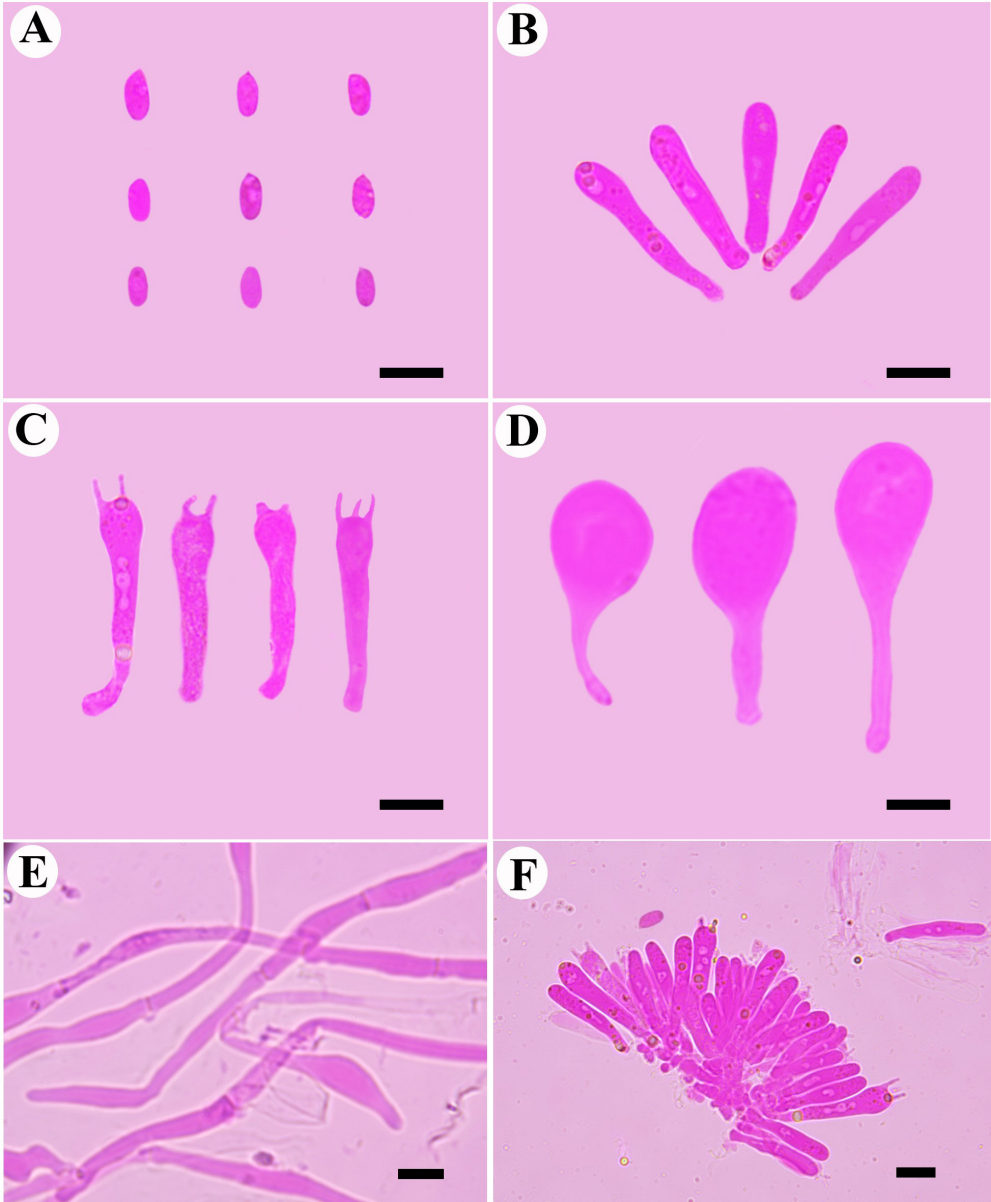
Anatomical characters of *Mycena longnanensis*, sp. nov. **A** Basidiospores, **B** probasidia, **C** basidia, **D** cheilocystidia, **E** hyphae of the pileipellis, **F** hymenium. Scale bar: 10 μm.

##### Diagnosis.

The species is characterized by an umbonate, radially fibrillose, lilac to dark purple pileus, decurrent and lilac lamellae, ellipsoid to subcylindrical basidiospores (6.75 × 3.45 µm), and spheropedunculate, small cheilocystidia (39 × 14.21 µm), with a slightly long tail and a round base.

##### Holotype.

**CHINA**, Gansu Province, Longnan City, Dangchang County, 34°N, 103°8'E, in mixed coniferous-broadleaf forests, 4 September 2024, FLF1804 (GenBank ITS = PX612291; *tef1-α* = PX684297).

##### Description.

***Basidiomata*** medium-sized. ***Pileus*** 2.2–2.4 cm in diameter, grayish lilac (5P 18) to dark purple (7.5P 5/10), campanulate, with a round and prominent lilac (10P 8/2) umbo. Surface radially fibrillose and sticky. ***Margin*** decurved to incurved, sulcate to slightly undulating, dark purple (7.5P 5/10) in a ring, followed by a lilac (10P 8/2) circle. ***Context*** thin, grayish lilac. ***Lamellae*** uncinate, deeply decurrent, lilac (10P 8/2), broad, crowded, thick, even, crenate, forking. ***Lamellulae*** frequent, irregular, in more than two tiers, alternating with lamellae. ***Stipe*** c. 4.3 cm long, reddish purple (2.5RP 2/2) to dark purple brown (10YR 2/3) with lilac (10P 8/2) ting at base, central, flexuous, cylindrical, tapering towards the base, fibrillose, fine appressed longitudinal fibrils, solid.

***Hyphal system*** composed of generative clamped hyphae, dextrinoid, inflated in KOH, thin-walled, occasionally branched, regularly to subregularly arranged, 5.87–14.94 µm in diameter.

***Basidiospores*** 5.73–8.28 × 2.75–4.39 µm, av. *L* × av. *W* = 6.75 × 3.45 µm, *Q* = 1.85–1.96, long-ellipsoid to subcylindrical with an apiculus and oil drop, smooth, hyaline in 5% KOH, amyloid, thin-walled. ***Probasidia*** cylindrical to clavate, thick-walled. ***Basidia*** 22.83–31.92 × 4.68–6.75 µm, av. *L* × av. *W* = 27.88 × 5.92 µm, *Q* = 4.95, narrowly clavate, smooth, hyaline in 5% KOH, thin-walled, two or four-spored. ***Cheilocystidia*** 31.61–47.81 × 10.79–17.70 µm, av. *L* × av. *W* = 39.00 × 14.21 µm, abundant, spheropedunculate with slightly long tail, round apex, smooth, hyaline in 5% KOH, thin-walled. ***Pleurocystidia*** not observed. ***Pileipellis*** a cutis of smooth, thin-walled and clamped hyphae of 12.15–18.21 µm in diameter.

##### Additional specimen (isotype) examined.

**CHINA**. Gansu Province, Longnan City, Dangchang County, 34°N, 103°8'E, in mixed coniferous-broadleaf forests, 4 September 2024, MSX1804. (GenBank ITS = PX612292; *tef1-α* = PX684298).

##### Notes.

The new species is characterized by an umbonate, radially fibrillose, lilac to dark purple pileus, decurrent and lilac lamellae, ellipsoid to subcylindrical basidiospores (6.75 × 3.45 μm), and smaller, spheropedunculate cheilocystidia (39 × 14.21 μm) with a slightly elongated tail and rounded apex. It resembles *M. brunneoviolacea* A.C. Cooper, Desjardin & B.A. Perry in its pileus and stipe morphology. However, the new species is distinguished by a grayish-lilac umbo and a radially fibrillose pileus. Furthermore, *M. brunneoviolacea* possesses ascending to adnate, white to pale gray lamellae, whereas *M. longnanensis* features uncinate, deeply decurrent, lilac lamellae. Anatomically, *M. longnanensis* can be differentiated by its unique, smaller, spheropedunculate cheilocystidia (av. 39.0 × 14.2 μm), whereas *Mycena brunneoviolacea* possesses larger, lageniform cheilocystidia (32–72 × 9.6–18.4 μm) (Cooper et al. 2018). Another similar species is *M. diosma* Krieglst. & Schwöbel, which features basidiomata with a dark violet-brown or reddish-purple, hygrophanous pileus. Furthermore, *M. diosma* is distinguished by its dark brownish-violet to dark violet lamellae and larger cheilocystidia [20–60(–80) × 3.5–20 μm] (Aronsen and Læssøe 2016). Another species in the same clade is *M. cahaya* A.L.C. Chew & Desjardin, which has a brown pileus, elongated basidiospores (av. 7.1 × 3.8 μm), and abundant clavate to ventricose cheilocystidia (Chew et al. 2014). *Mycena longnanensis* resembles *M. glabra* in having long-ellipsoid to subcylindrical basidiospores and bi- or tetrasporic basidia. However, *M. longnanensis* is distinguished by its dark purple pileus, lilac lamellae, and reddish-purple to dark purple-brown stipe, whereas *M. glabra* has white to cream basidiomata, white to pale pink-yellow lamellae, and a white to pale wax-yellow stipe. Anatomically, *M. longnanensis* is distinguished from *M. glabra* by its narrower basidiospores (2.75–4.39 μm vs. 3.7–5.5 μm), significantly smaller basidia (22.83–31.92 × 4.68–6.75 μm vs. 29.0–45.0 × 6.3–12.0 μm), and shorter cheilocystidia (31.61–47.81 × 10.79–17.70 μm vs. 48.0–77.4 × 10.0–16.4 μm) (Fan et al. 2024).

#### 6. Mycena minispora

Taxon classificationFungiAgaricalesMycenaceae

S. X. Ma, A. Naseer, A. N. Khalid & L. F. Fan sp. nov.

CD21CCB0-AA89-520B-BE1C-426C2E20F8E8

860922

Figs 12, 13

##### Etymology.

The specific epithet minispora” refers to the small-sized basidiospores.

**Figure 12.**
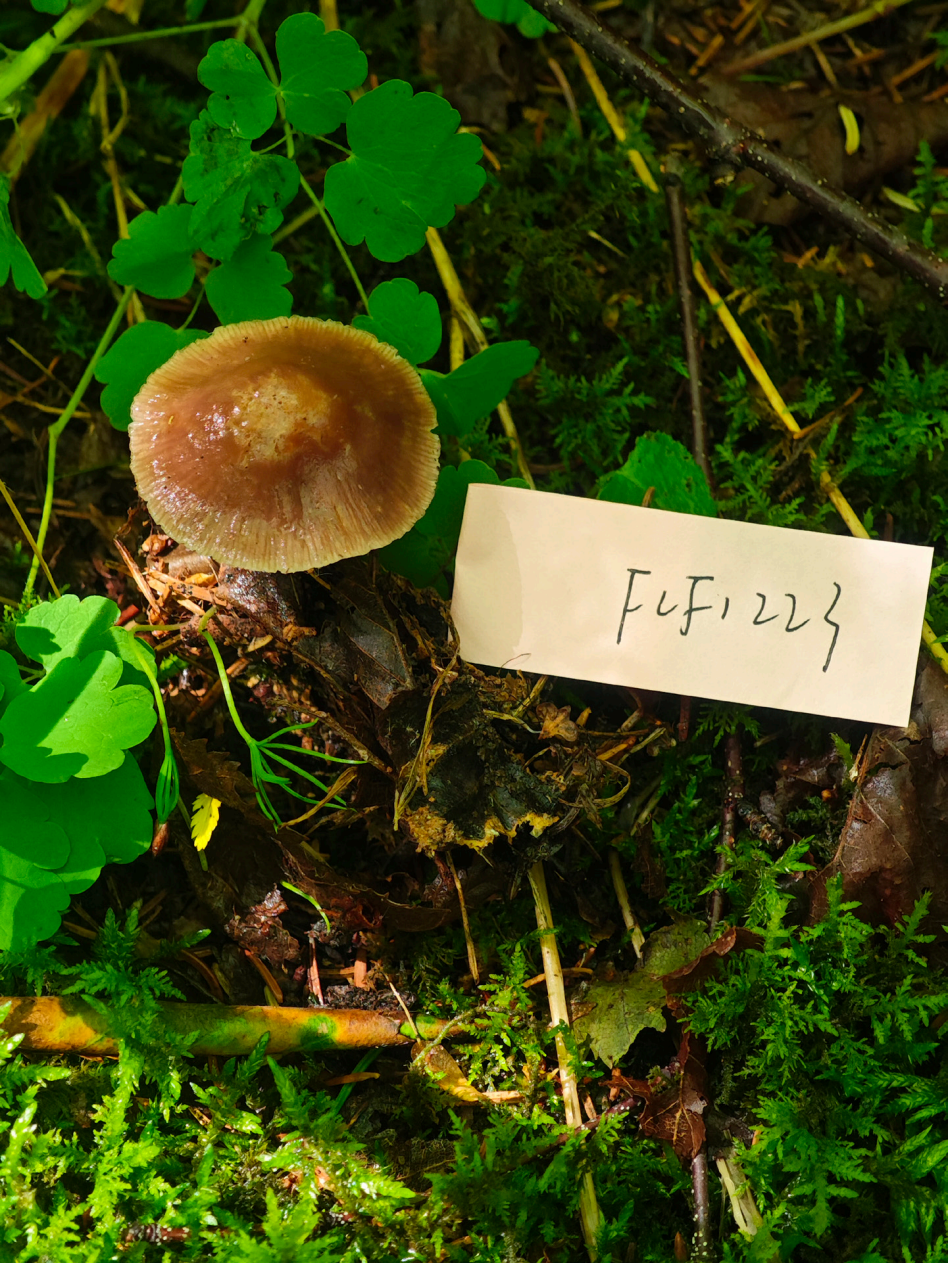
Morphological features of *Mycena minispora*, sp. nov., showing the stipe, pileus, and lamellae. Scale bars: 1 cm.

**Figure 13.**
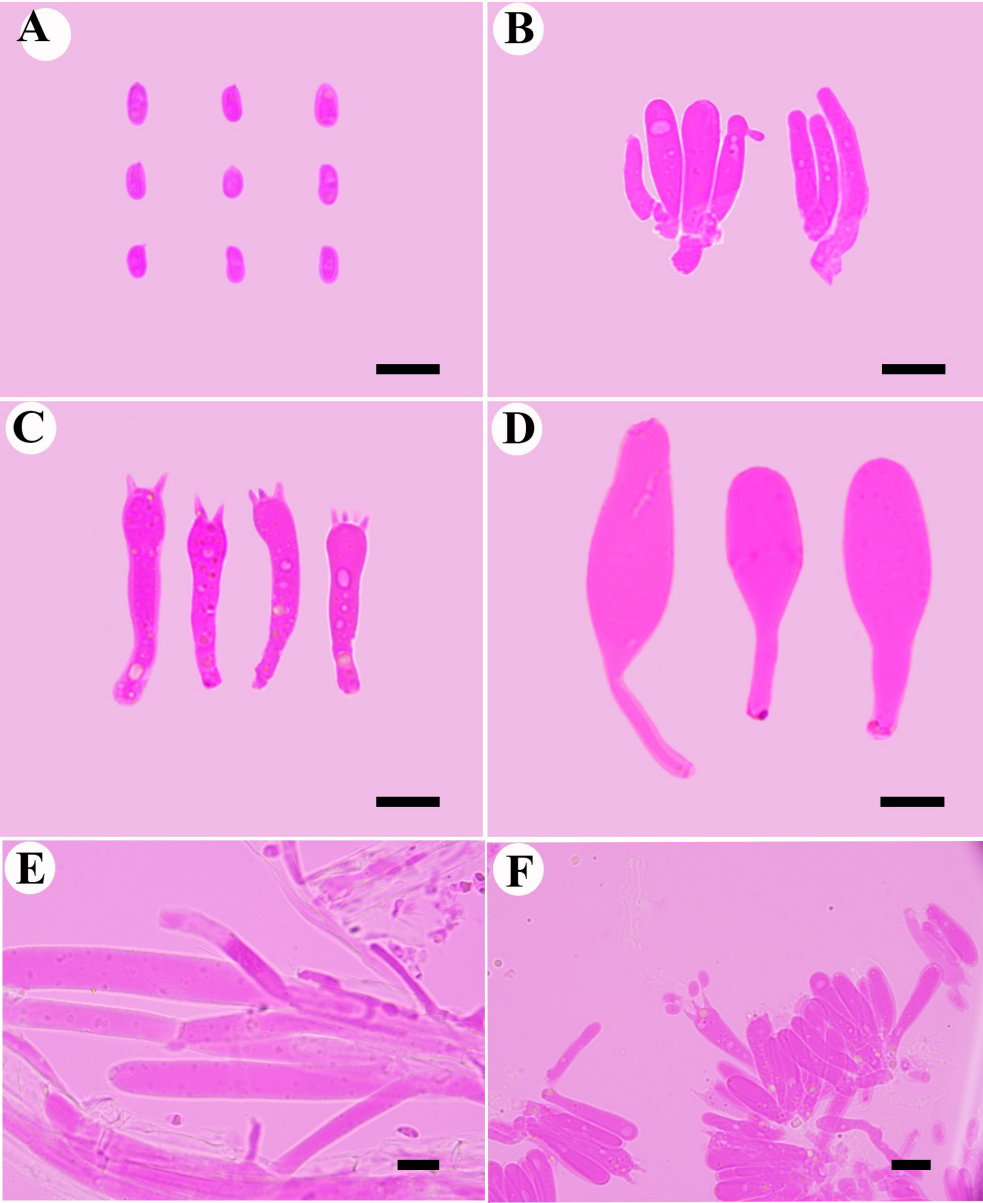
Anatomical characters of *Mycena minispora*, sp. nov. **A** Basidiospores, **B** probasidia, **C** basidia, **D** cheilocystidia, **E** hyphae of the pileipellis, **F** hymenium. Scale bar: 10 μm.

##### Diagnosis.

*Mycena minispora* is characterized by its glutinous, fibrillose golden-brown pileus with a low obtuse umbo, milky-yellow lamellae, smaller (6.04 × 3.13 µm) amyloid basidiospores and fusiform to subclavate cheilocystidia with blunt and rounded apices.

##### Holotype.

**CHINA**, Gansu Province, Gannan Tibetan Autonomous Prefecture, Yeliguan National Forest Park, 34°95'N, 103°60'E, in mixed coniferous, broadleaf forests, 5 July 2024, FLF1223 (GenBank ITS = PX612293).

##### Description.

***Basidiomata*** large-sized. ***Pileus*** 3.5–3.7 cm in diameter, brown (7.5YR 6/8) with a light golden-brown (2.5Y 7/8) umbo and margins; plane to slightly convex with a low, obtuse, non-prominent umbo; surface glutinous to viscid, hygrophanous, and fibrillose; margin straight to very slightly uplifted, plicate. ***Context*** thin, white to gray. ***Lamellae*** sinuate, edges eroded to crenate, milky-yellow (5Y 8/1), broad, close, thick, even, unequal. ***Lamellulae*** frequent, intervenose, irregular, arranged in more than two tiers and alternating with the lamellae. ***Stipe*** approximately 4.7 cm long, eccentric, terete and cylindrical, firm, ribbed or costate, pale yellow (5Y 8/4) with a light green (7.5GY 8/2) tinge at the base; apex slightly pruinose.

***Hyphal system*** composed of generative clamped hyphae, dextrinoid, inflated in KOH, thin-walled, septate, regular to subregularly arranged, 6.28–10.59 µm in diameter.

***Basidiospores*** 4.61–6.92 × 2.28–4.04 µm, av. *L* × av. *W* = 6.04 × 3.13 µm, *Q* = 1.89–1.95, ellipsoid to subcylindrical with a distinct apiculus and oil drop, smooth, pale yellow in 5% KOH, amyloid, thin-walled, guttulated. ***Probasidia*** subclavate to clavate, thin-walled. ***Basidia*** 17.28–26.82 × 4.67–6.46 µm, av. *L* × av. *W* = 23.96 × 5.62 µm, *Q* = 4.21–4.25, narrowly clavate, pale yellow in 5% KOH, thin-walled, four-spored. ***Cheilocystidia*** 35.82–59.93 × 12.86–20.37 µm, av. *L* × av. *W* = 48.76 × 16.18 µm, abundant fusiform to subclavate with a blunt, rounded apex, smooth, pale yellow in 5% KOH, thin-walled. ***Pleurocystidia*** not observed. ***Pileipellis*** a cutis, made up smooth, thin-walled and clamped hyphae of 13.62–21.32 µm in diameter.

##### Additional specimen examined.

**CHINA**. Gansu Province, Gannan Tibetan Autonomous Prefecture, Yeliguan National Forest Park, 34°95'N, 103°50'E, in mixed coniferous-broadleaf forests, 5 July 2024, FLF1244. (GenBank ITS = PX612294; *tef1-α* = PX684299).

##### Notes.

The presence of a hygrophanous, brownish pileus, intervenose lamellae, smooth cheilocystidia, and amyloid basidiospores confirms the placement of *M. minispora* within *Mycena sect. Calodontes*. Phylogenetic analyses based on ITS and *tef1-α* further support this taxonomic position.

*Mycena minispora* is characterized by a brown pileus with a light golden-brown umbo, milky yellow, sinuate lamellae with eroded to crenate edges, and a pale-yellow stipe exhibiting a light green tinge at the base. Microscopically, the species possesses small, ellipsoid to subcylindrical basidiospores (6.04 × 3.13 μm) and fusiform or pyriform cheilocystidia. *Mycena minispora* can be readily distinguished from *M. pura* (Pers.) P. Kumm, which has larger basidiospores (6–9.5 × 4–5.2 μm) and pink to violet lamellae. *Mycena minispora* is separated from *M. luciferina*, which is characterized by a campanulate, reddish-pink to pale pink pileus and white intervenose lamellae. In contrast, *M. minispora* possesses a hygrophanous, brownish pileus, a yellow stipe with a light green base, and smaller basidiospores (6.04 × 3.13 μm vs. 7.4 × 3.8 μm; Cortés-Pérez et al. 2023).

Another morphologically similar species is *M. shengshanensis*, which shares several features with *M. minispora*, including a convex pileus with an obtuse, disc-like umbo, a paler striate margin, golden-brown to brown coloration, sinuate and irregularly serrulate lamellae, and a central, cylindrical stipe. However, *M. minispora* differs by having a larger pileus (3.5–3.7 cm vs. 1.3–2.6 cm) and a pale-yellow stipe with a light green tinge at the base, whereas *M. shengshanensis* possesses a reddish-brown to brownish-gray stipe. Anatomically, *M. minispora* is further distinguished by its smaller basidiospores and basidia (4.61–6.92 × 2.28–4.04 μm vs. 6.10–8.10 × 3.40–4.40 μm for basidiospores; 17.28–26.82 × 4.67–6.46 μm vs. 22–32 × 6–8 μm for basidia; Liu et al. 2022).

#### 7. Mycena pakistanica

Taxon classificationFungiAgaricalesMycenaceae

A. Naseer, A. Khalid, A.N. Khalid, S. X. Ma & L. F. Fan sp. nov.

31266469-4D51-548A-B9C0-BD61FFEA5B60

860923

Figs 14, 15

##### Etymology.

The specific epithet “pakistanica” refers to the country name, Pakistan.

**Figure 14.**
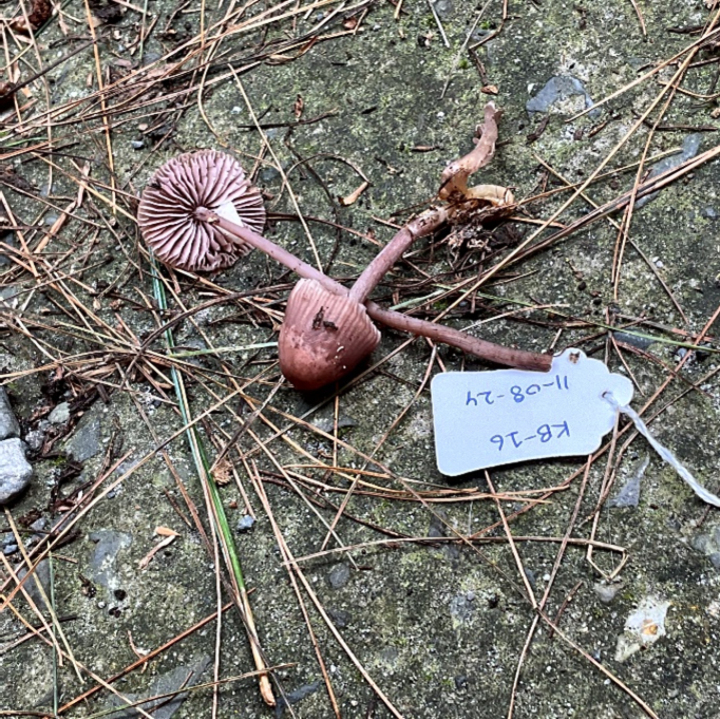
Morphological features of *Mycena pakistanica* sp. nov. showing the stipe, pileus, and lamellae. Scale bar: 0.9 cm.

**Figure 15.**
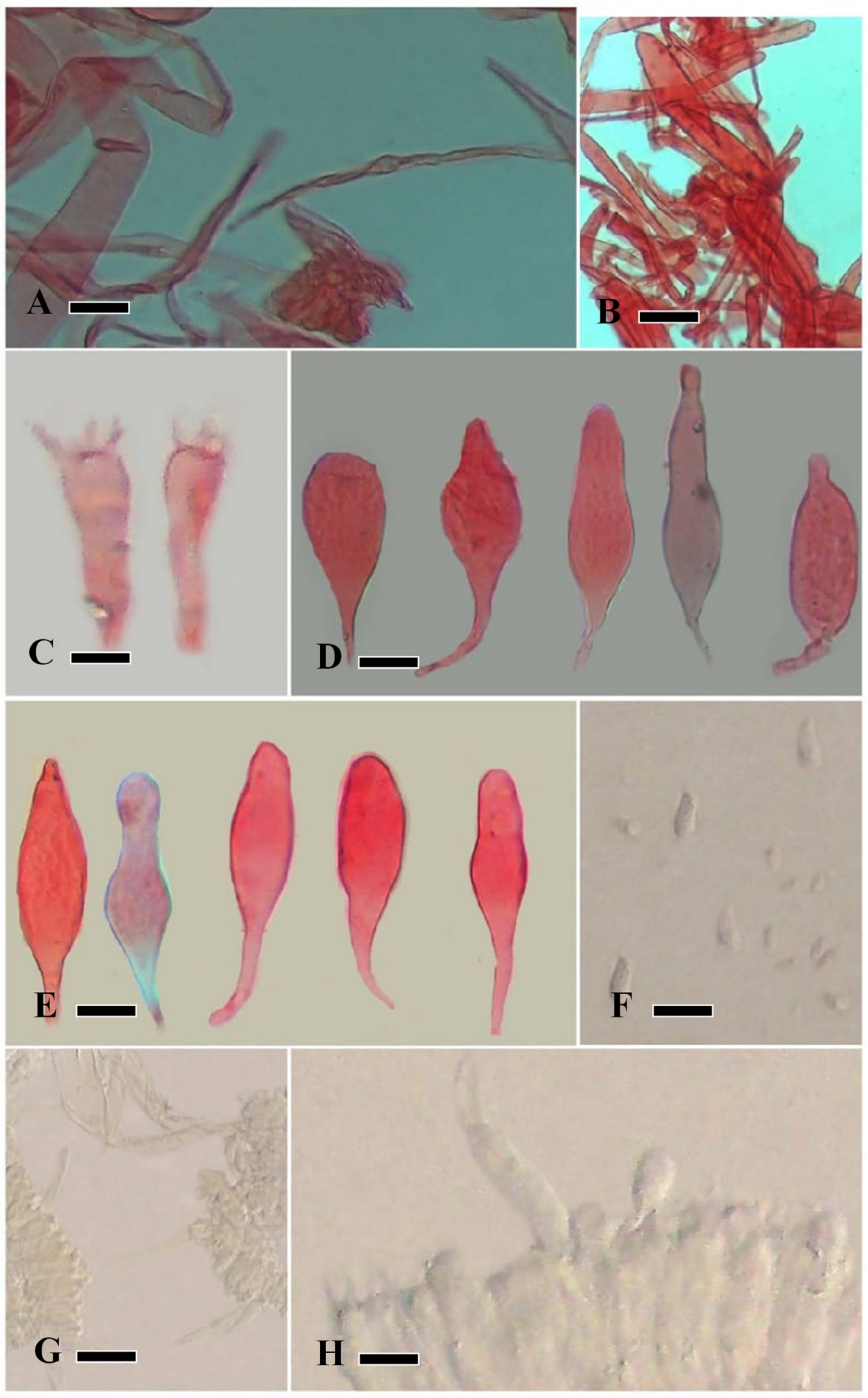
Anatomical characters of *Mycena pakistanica* sp. nov. **A** Hyphae of the pileipellis, **B** hyphae of the stipitipellis, **C** basidia, **D** cheilocystidia, **E** pleurocystidia, **F** basidiospores, **G, H** hymenium in KOH. Scale bars: 25.6 μm (**A**); 29.2 μm (**B**); 17.1 μm (**C, D**); 10 μm (**E**); 4.7 μm (**F**); 15.4 μm (**G**); 15.5 μm (**H**).

##### Diagnosis.

The species is characterized by a lavender pileus with grape wine fibrils, larger (12 cm) stipe, lacrymoid subcylindrical to ellipsoid, smaller (5.9 × 2.6 µm) basidiospores, spathulate pleurocystidia, and fusiform to sublageniform cheilocystidia with both sharp ends.

##### Holotype.

**PAKISTAN**, Khyber Pakhtunkhwa Province, Abbottabad district, Kala Bagh, 34°2'0"N, 73°25'0"E, in mixed coniferous forest, found on wood, 11 August 2024, Arooj Naseer, KB–16 (GenBank ITS = PX612297).

##### Description.

***Basidiomata*** large-sized, solitary, or scattered. ***Pileus*** 2.0–2.9 cm in diameter, lavender (7.5R 5/3) with grape-wine (7.5R 6/2) fibrils; shape conical to parabolic, becoming planate when mature; surface dry, fibrillose, with distinct veil remnants; margin decurved, becoming slightly incurved with age, varying from plicate or undulating when young to sulcate and entire at maturity. ***Context*** thin, lavender. ***Lamellae*** emerginate, light grayish-purple (N 7/0), ventricose, thick, and subdistant, unequal to forked; ***Lamellulae*** frequent, irregular, arranged in one to two tiers alternating with the lamellae. ***Stipe*** 8.0–12.0 × 0.1–0.3 cm, central, equal to slightly flexuous, fragile; surface smooth to fibrillose towards the base, light grayish-purple (N 7/0) at the apex, becoming dark purplish-brown (7.5YR 3/4) towards the base.

***Hyphal system*** composed of generative clamped hyphae, dextrinoid, inflated in KOH. generative hyphae in context brown to reddish brown, thin-walled, occasionally branched, septate, irregularly arranged, 2.2–11.8 µm in diameter. Generative hyphae in lamella hyaline in KOH, thin-walled, occasionally branched, irregularly arranged, 2–8 µm in diameter.

***Basidiospores*** (4.4–)4.9 – 7.3(–7.4) × (2.2–)2.4 – 2.8(–3) µm, av. *L* × av. *W* = 5.9 × 2.6 µm, *Q* = 1.8 – 3.3, av. *Q.* = 2.3, lacrymoid, subcylindrical to ellipsoid, with suprahilar depression, phaseoliform in side view, oblong in frontal view, apiculate, smooth, hyaline to light yellow in 5% KOH, amyloid, thin-walled. ***Basidia*** (18.6–)18.8 – 22.3(–22.9) × (4.5–)4.6 – 5.7(–6) µm, av. *L* × av. *W* = 20.1 × 5.4 µm, clavate to slightly pyriform, smooth, hyaline in 5% KOH, thick-walled, mostly four-spored. ***Cheilocystidia*** (57.8–)58.2 – 78.4(–91) × (11.2–)12.7 – 16.2(–16.4) µm, av. *L* × av. *W* = 71.6 × 13.9 µm, frequently present, mostly clavate to broadly clavate with acute apices, fusiform to sublageniform with both sharp ends, rarely lecythiform, mucronate, smooth, hyaline to light brown in 5% KOH, thick-walled. ***Pleurocystidia*** (49.5–)54.2 – 72(–74) × (9.5–)10 – 17.6(–19.5) µm, av. *L* × av. *W* = 62.3 × 14.3 µm, similar in shape to cheilocystidia but smaller, mostly clavate to spathulate with knob-like outgrowth, narrowly lageniform to mucronate or with cylindrical outgrowth, smooth, hyaline to light brown in 5% KOH, thin to rarely thick-walled. ***Pileipellis*** a cutis, made up of irregular, branched 1.6–7.6 µm, average width 4.02 µm, inflated, hyaline to pale yellow in 5% KOH, septate, thick-walled.

##### Notes.

The presence of a pinkish, reddish, purplish to brownish pileus, large-sized basidiomata, and smooth hymenial cystidia supports the placement of *M. pakistanica* within *Mycena sect. Calodontes*. *Mycena pakistanica* is characterized by a lavender pileus with grape-wine fibrils, a long stipe (up to 12 cm), and small, lacrymoid, subcylindrical to ellipsoid basidiospores (av. 5.9 × 2.6 μm). Additionally, it features spathulate pleurocystidia and fusiform to sublageniform cheilocystidia with acute apices.

*Mycena pakistanica* shares morphological similarities with *Mycena pura*, including light purplish-gray lamellae, a dark purplish-brown, smooth, and equal stipe, and clavate to fusiform cheilocystidia. However, *M. pakistanica* can be distinguished from *M. pura* by its smaller (2.9 cm in diameter), conical to flat, fibrillose pileus with plicate to sulcate margins and emarginate lamellae. Additionally, *M. pakistanica* possesses a longer (12 cm in length) stipe that is fibrillose toward the base, whereas *M. pura* has a relatively shorter stipe (up to 11 cm) that is pruinose to glabrescent. Anatomically, *M. pakistanica* is distinguished by its smaller, lacrymoid, subcylindrical to ellipsoid basidiospores (av. 5.9 × 2.6 μm), whereas *M. pura* possesses larger, narrowly ellipsoid to cylindrical basidiospores (av. 8.4 × 4 μm) (Perry 2002). Moreover, *M. pakistanica* has larger, sublageniform cheilocystidia with both ends sharp and mucronate (up to 91 μm) and spathulate pleurocystidia (up to 74 μm).

*Mycena pakistanica* is also comparable with *M. pelianthina* (Fr.) Quél. (1872). Both share a dark brown pileus, purple lamellae, an equal and fibrillose stipe, thin-walled clavate basidia, and fusiform cheilocystidia. However, *M. pelianthina* differs from *M. pakistanica* mainly in its larger (up to 50 mm), hemispherical to campanulate pileus with a glabrous surface that is slightly lubricous when moist. Furthermore, *M. pelianthina* features adnate to decurrent lamellae and a longer (up to 120 mm), whitish to lilaceous stipe that is striate with dark fibrils. Microscopically, *M. pelianthina* is distinguished by larger pip-shaped basidiospores (av. 7 × 3.8 μm), smaller cheilo- and pleurocystidia (up to 70 μm), and smooth cortical hyphae in the pileipellis (Harder et al. 2010, 2012).

#### 8. Mycena tephroleuca

Taxon classificationFungiAgaricalesMycenaceae

S. X. Ma, A. Naseer, A. N. Khalid & L. F. Fan sp. nov.

8899FD5B-D59C-5D5C-885F-3F42F483F6A6

860925

Figs 16, 17

##### Etymology.

The specific epithet **“tephroleuca”** refers to color of the basidiomata.

**Figure 16.**
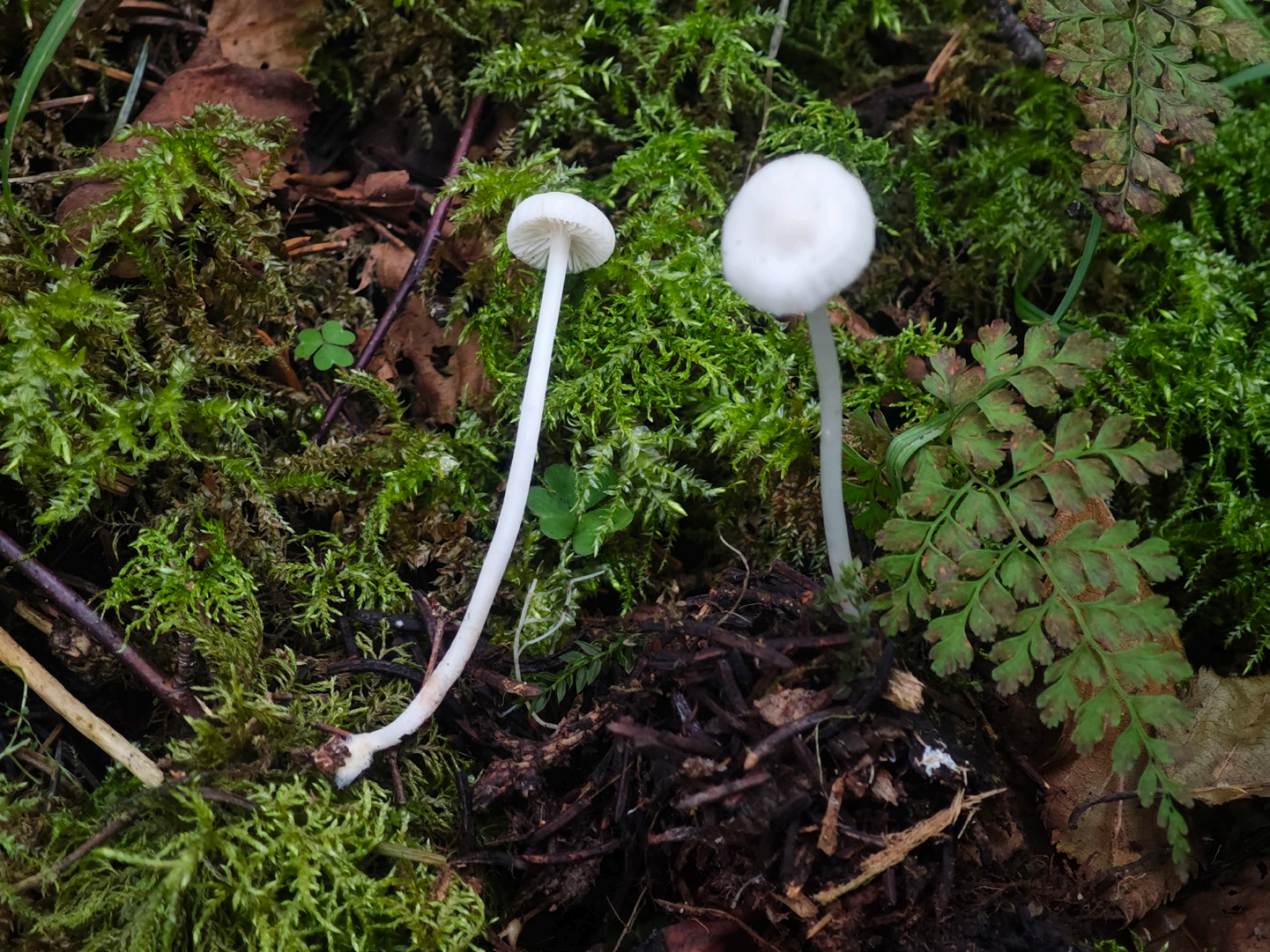
Morphological features of *Mycena tephroleuca* sp. nov., showing the pileus, stipe, and lamellae. Scale bar: 1 cm.

**Figure 17.**
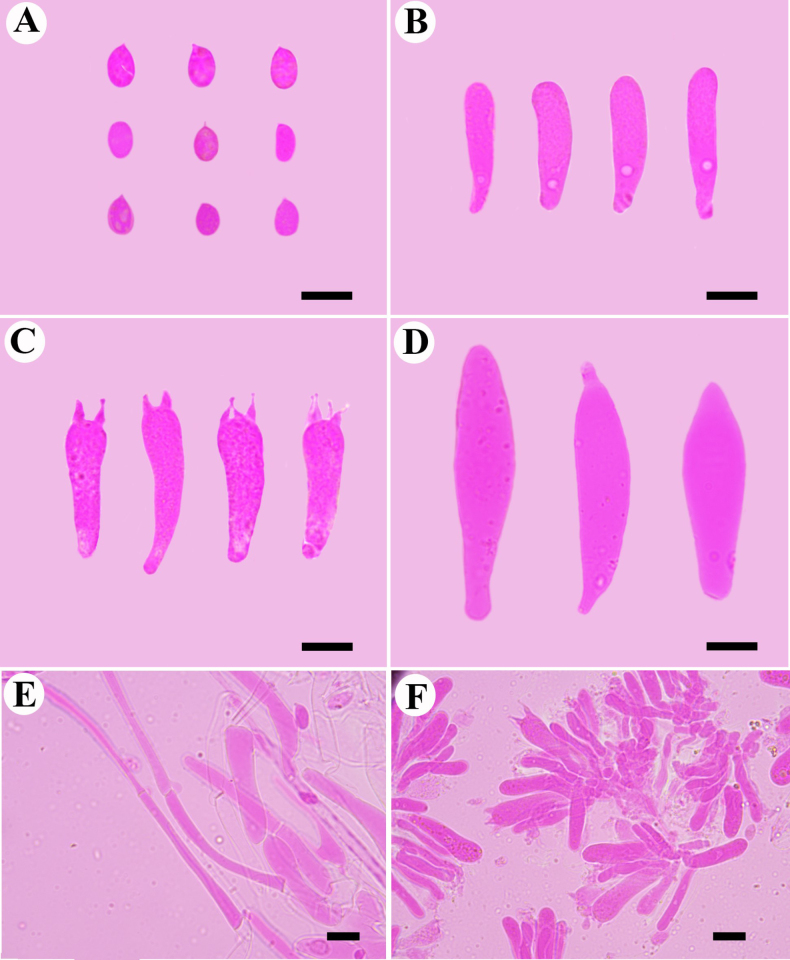
Anatomical characters of *Mycena tephroleuca* sp. nov. **A** Basidiospores, **B** probasidia, **C** basidia, **D** cheilocystidia, **E** hyphae of the stipitipellis, **F** hymenium. Scale bar: 10 μm.

##### Diagnosis.

The species is characterized by white to grayish-white pileus, adnate lamellae with a decurrent tooth, a white stipe with brown tinge at base, thin-walled hyphae and clamped, and the presence of clavate to narrowly fusiform or utriform cheilocystidia with cylindrical outgrowths.

##### Holotype.

**CHINA**, Gansu Province, Gannan Tibetan Autonomous Prefecture, Diebu County, Lazikou Forest Protection Station, 33°85'N, 104°55'E, in broad-leaved forests, 4^th^ September 2024, FLF1816 (GenBank ITS = PX612306; *Tef1-α* = PX684309).

##### Description.

***Basidiomata*** small-sized. ***Pileus*** 0.7–1.5 cm in diameter, chalky white (10R 8/1) to chalky white (10R 8/1), pulvinate, slightly sticky, glabrous in center, marginally fibrillose, even, entire to plicate, margins incurved to straight. ***Context*** thin, white. ***Lamellae*** adnate with decurrent tooth, fimbriate, chalky white (10R 8/1), broad, subdistant, thick, unequal, wavy. ***Lamellulae*** are of two different sizes, in two tiers, alternating with lamellae. ***Stipe*** 3.8–4.3 cm long, chalky white (10R 8/1) to smoky white (2.5YR 8/1), flexuous, equal, surface smooth, with some brown tinge at base, white rhizomorphs, central.

***Hyphal system*** composed of generative hyphae with clamp connections, dextrinoid, inflated in KOH, thin-walled, regular to subregularly arranged, 1.26–9.20 µm in diameter.

***Basidiospores*** 7–8.77 × 3.8–5.9 µm, av. *L* × av. *W* = 7.73 × 4.79 µm, *Q* = 1.61–1.73, oblong to elliptical, with an apiculus and oil drop, smooth, hyaline in 5% KOH, amyloid, thin-walled. ***Basidia*** 24.97–32.57 × 7.38–9.16 µm, av. *L* × av. *W* = 29.45 × 8.19 µm, clavate, guttulated, hyaline, thin-walled, two to four-spored. ***Cheilocystidia*** 48.22–54.80 × 13.04–18.80 µm, av. *L* × av. *W* = 14.26 × 14.7 µm, clavate, narrowly fusiform, utriform, mucronate or with a cylindrical outgrowth, smooth, hyaline in 5% KOH, thin-walled. ***Pleurocystidia*** not observed. ***Pileipellis*** a cutis made up clamped hyphae of 10.08–24.14 µm in diameter, smooth, thin-walled.

##### Additional specimen (paratype) examined.

**CHINA**. Gansu Province, Gannan Tibetan Autonomous Prefecture, Lazikou Forest Protection Station, Laolong Gully, 33°85'N, 104°55'E, in broad-leaved forests, 4 September 2024, MSX1816. (GenBank ITS = PX612307; *tef1-α* = PX684310).

##### Notes.

The species is characterized by small-sized basidiomata, a chalky-white to grayish-white pileus, adnate lamellae with a decurrent tooth, a chalky-white to smoky-white stipe with some brown tinge at the base, a pileipellis as a cutis made up of clamped hyphae 10.08–24.14 μm in diameter, smooth, thin-walled hyphae, and abundant, smooth, clavate to narrowly fusiform or utriform cheilocystidia with cylindrical outgrowths.

*Mycena tephroleuca* shows similarity to *M. cahaya* in the size of its basidiospores and two- or four-spored basidia. However, *M. tephroleuca* possesses a pulvinate, marginally fibrillose pileus with entire to plicate margins and a brown tinge at the stipe base with white rhizomorphs, whereas *M. cahaya* possesses a parabolic to convex or campanulate, uneven to slightly wrinkled pileus and a yellowish-gray to beige, glabrous stipe. Anatomically, *M. tephroleuca* is distinguished by spherical to phaseoliform basidiospores and broader basidia, whereas *M. cahaya* has elongate to cylindrical basidiospores and narrower basidia (7.38–9.16 μm vs. 4.0–6.4 μm) (Chew et al. 2014).

*Mycena tephroleuca* the novel species described in this study, forms a sister group to *M. glabra* B.Y. Wang, T.F. Ma & L.F. Fan in the phylogenetic analysis, sharing similarities in its long ellipsoid basidiospores and comparable basidial size and shape. Morphologically, *M. tephroleuca* possesses a chalky-white to grayish-white, pulvinate pileus that is glabrous at the center and fibrillose at the margin, with fimbriate lamellae, whereas *M. glabra* features a white to cream pileus with vertical stripes or grooves and subdecurrent lamellae. In *M. tephroleuca*, the stipe is flexuous, chalky-white to smoky-white with brown tinges at the base, and covered with white rhizomorphs, whereas *M. glabra* has a smooth, white to cream stipe that is pale wax-yellow at the base. Anatomically, *M. tephroleuca* has broader cheilocystidia (13.04–18.80 μm), whereas *M. glabra* has narrower cheilocystidia (10.0–16.4 μm).

*Mycena rufobrunnea* shares morphological affinities with *Mycena tephroleuca*, particularly in small-sized basidiomata with a relatively thin, white pileus context, basidiospore dimensions, and the absence of pleurocystidia. However, *M. rufobrunnea* is readily distinguished by its vividly colored stipe, ranging from grayish-magenta to dark purple, in contrast to the entirely chalky-white to smoky-white stipe of *M. tephroleuca*. Anatomically, *M. tephroleuca* possesses smaller basidiospores (7.73 × 4.79 μm) and larger cheilocystidia (48.22–54.80 × 13.04–18.80 μm) that are more morphologically diverse, including mucronate forms and cylindrical outgrowths. In contrast, *M. rufobrunnea* has larger basidiospores (8.3 × 4.4 μm) and smaller, utriform to clavate cheilocystidia (23–44 × 7–17 μm) (Wei et al. 2024). This species also shares similarities with another taxon described in this study, *Mycena lavendula*, which is characterized by a lilac pileus and reddish-purple stipe with a lilac base, longitudinal striations, and a pruinose apex. Anatomically, *M. lavendula* has subcylindrical to ellipsoid basidiospores (6.68 × 2.56 μm), polymorphic cheilocystidia with rounded apices, and clamped generative hyphae.

#### 9. Phloeomana qilianensis

Taxon classificationFungiAgaricalesPorotheleaceae

S. X. Ma, A. Naseer, A. N. Khalid & L. F. Fan sp. nov.

D767F70A-4551-5F74-9344-561B1D007542

860924

Figs 18, 19

##### Etymology.

The specific epithet “*qilianensis*” refers to the type locality, Qilian.

**Figure 18.**
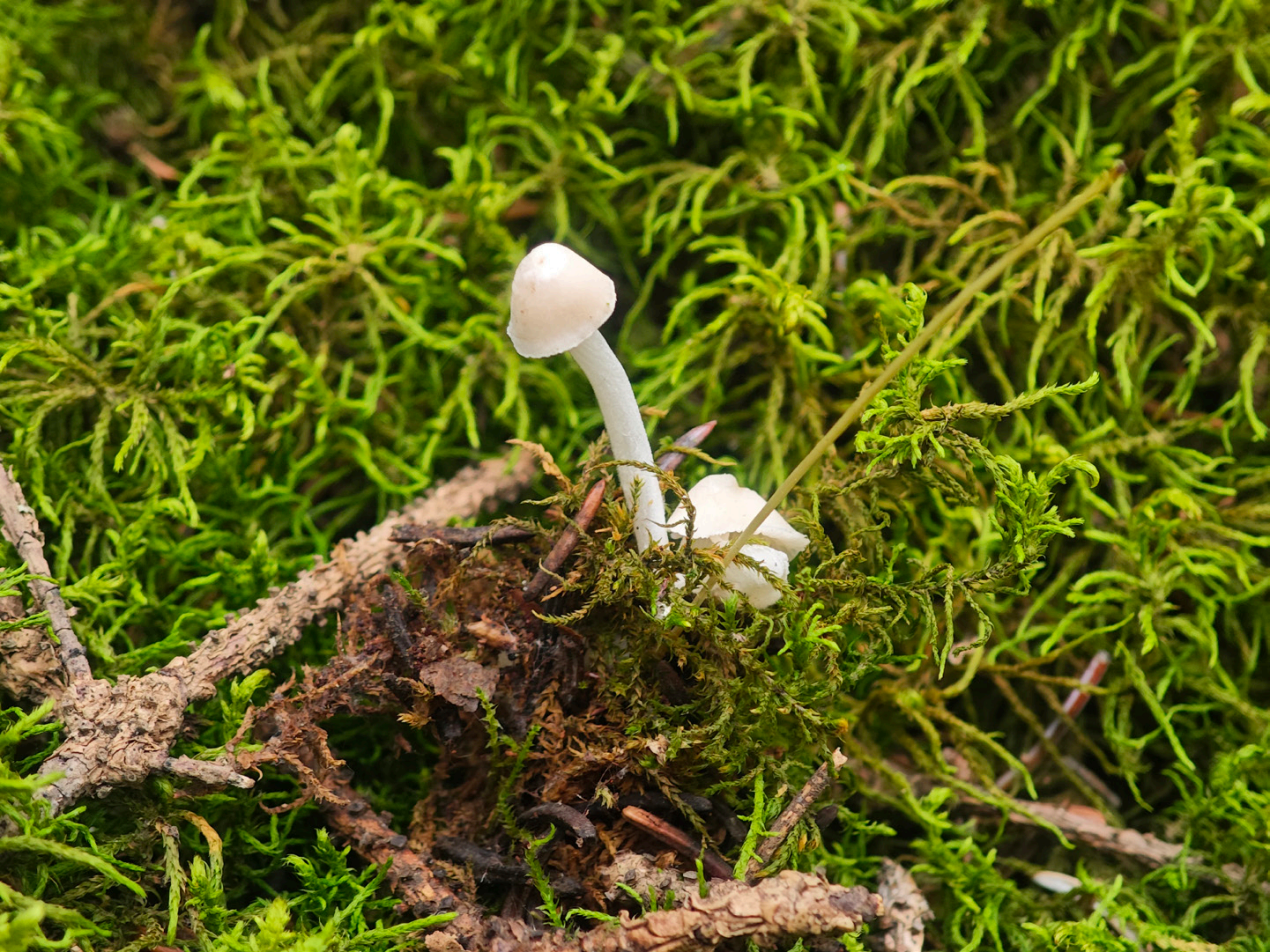
Morphological features of *Phloeomana qilianensis* sp. nov., showing the stipe and lamellae. Scale bar: 1 cm.

**Figure 19.**
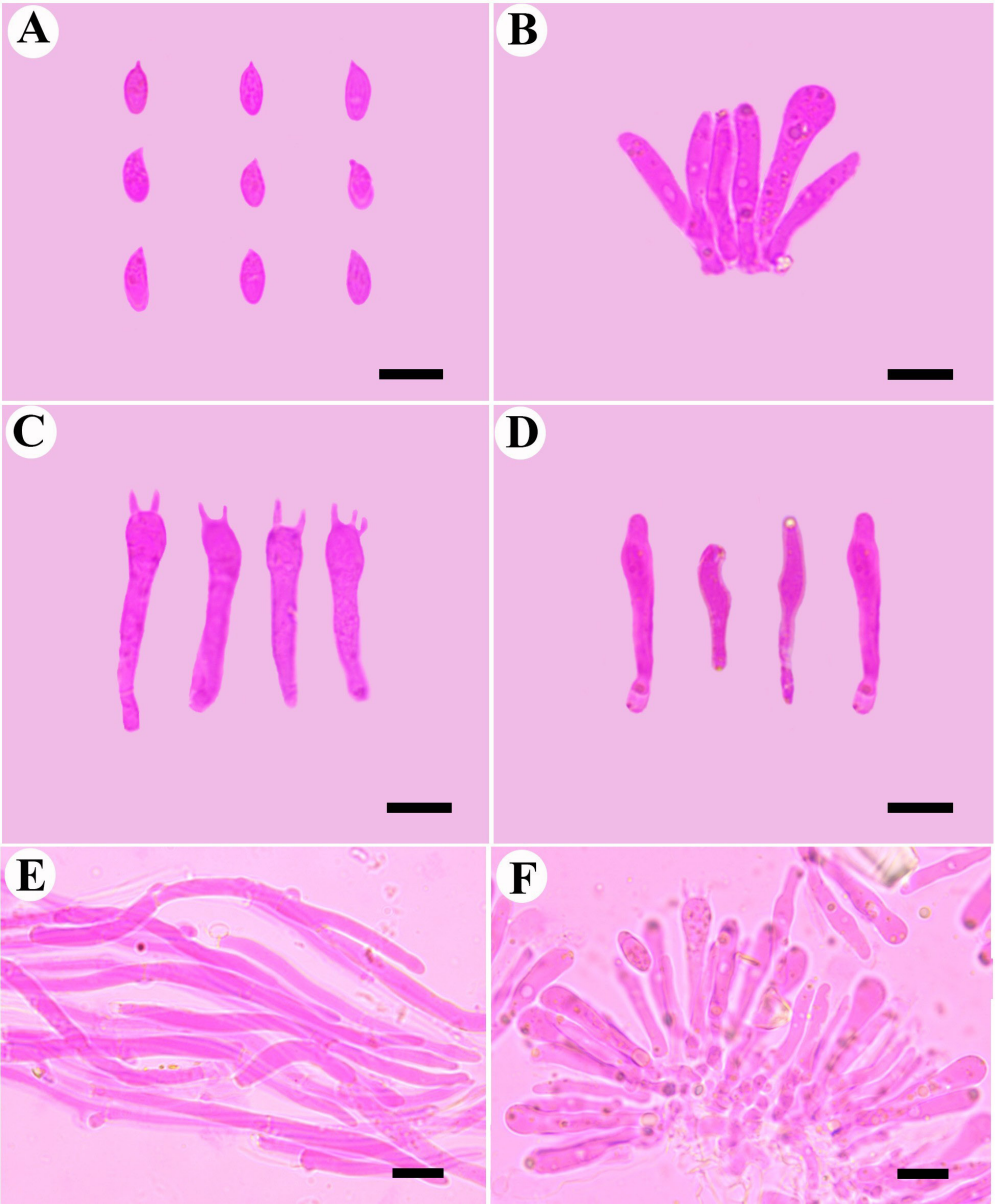
Anatomical characters of *Phloeomana qilianensis* sp. nov. **A** Basidiospores, **B** probasidia, **C** basidia, **D** cheilocystidia, **E** hyphae of the stipitipellis, **F** hymenium. Scale bar: 10 μm.

##### Diagnosis.

*Phloeomana qilianensis* is characterized by small (up to 1 cm) basidiomata, an off-white to pale yellow pileus, fimbriate lamellae, a furfuraceous stipe, lacrymoid basidiospores (7.39 × 3.99 µm), and abundant, fusiform to ventricose-rostrate cheilocystidia with an obtuse apex and tapered base.

##### Holotype.

**CHINA**, Gansu Province, Zhangye City, Qilian Mountains National Nature Reserve, Qinglong Resource Conservation Station, 38°68'N, 99°92'E, in mixed coniferous-broadleaf forests, 3 August 2024, FLF1582. (GenBank ITS = PX612301).

##### Description.

***Basidiomata*** small, in cluster. ***Pileus*** 0.4–1.0 cm in diameter, off-white (10R 8/1) when young, becoming pale yellow (2.5R 8/4) with age, campanulate to parabolic, dry, sometimes viscid, smooth at the center, fibrillose towards margins, plicate to split, margin incurved to slightly uplifted. ***Context*** thin, white. ***Lamellae*** regular, fimbriate, off-white (10R 8/1), narrow, subdistant, thick, unequal, even. ***Lamellulae*** of two lengths, arranged in two tiers, alternating with the lamellae. ***Stipe*** central, flexuous, cylindrical to slightly bulbous towards base 2.1–3.4 cm, smoky-white (2.5YR 8/1) with a white pruinose cpating, surface scurfy, branny, furfuraceous.

***Hyphal system*** composed of generative hyphae with clamp connections, IKI–, inflated in KOH, thin-walled, occasionally branched, septate, regular to subregularly arranged, 3.31–3.94 µm in diameter.

***Basidiospores*** 6–8.5 × 3.43–4.72 µm, av. *L* × av. *W* = 7.39 × 3.99 µm, *Q* = 1.72–1.85, lacrymoid, oblong ellipsoid to subcylindrical with an apiculus and oil drop, smooth, hyaline in 5% KOH, IKI–, guttulated, thin-walled. ***Probasidia*** cylindrical to clavate, thin-walled. ***Basidia*** 21.01–33.21 × 5.21–6.56 cm, av. *L* × av. *W* = 28.16 × 5.92 µm, *Q* = 4.75, narrowly clavate, guttulated, hyaline, thin–walled, two or four–spored. ***Cheilocystidia*** 24.65–28.56 × 3.13–3.87 µm, av. *L* × av. *W* = 27.04 × 3.37 µm, abundant, fusiform, ventricose-rostrate, with obtuse apex, base tapered, with short to long stalk, smooth, hyaline in 5% KOH, thin-walled. ***Pleurocystidia*** not observed. ***Pileipellis*** a cutis of smooth, thin-walled hyphae of 3.64–11.55 µm in diameter, with clavate terminal cells.

##### Additional specimen examined.

**CHINA**. Gansu Province, Qilian Mountains National Nature Reserve, Tianzhu Tibetan Autonomous County, Dongdatan, Xichakou Village, 37°32'N, 102°7'23"E, in mixed coniferous-broadleaf forests, 28 July 2024, FLF1269. (GenBank ITS = PX612300; *tef1-α* = PX684304).

##### Notes.

The presence of horizontal to arcuate lamellae with straight or concave edges, smooth cheilocystidia, and inamyloid spores confirms that the new species belongs to the genus *Phloeomana* Redhead. The new species is characterized by small, clustered basidiomata, an off-white to pale yellow pileus, fimbriate lamellae, a scurfy, furfuraceous stipe, lacrymoid to oblong-ellipsoid or subcylindrical basidiospores, and abundant, fusiform, ventricose-rostrate cheilocystidia with an obtuse apex and a tapered base.

*Phloeomana qilianensis* resembles *P. speirea* (Fr.) Redhead in having small basidiomata with furfuraceous surfaces and ellipsoid basidiospores. However, *P. qilianensis* can be distinguished from *P. speirea* by its off-white to pale yellow pileus and smoky-white stipe covered with white dust, whereas *P. speirea* displays a pale gray-brown to pale yellowish-brown pileus and a stipe that is densely but minutely puberulous throughout. Both species share similar cheilocystidia in terms of abundance and general fusiform shape, but they differ in apex morphology—*P. qilianensis* has ventricose-rostrate cheilocystidia with tapered bases and obtuse apices, whereas *P. speirea* features utriform or somewhat irregularly shaped cheilocystidia, usually simple but sometimes furcated, lobed, or bearing a few excrescences, and often curved to somewhat flexuous.

## Conclusion

According to preliminary molecular and morphological studies on the specimens collected from China and Pakistan, the species diversity of mycenoid taxa in Asia is underestimated, and many additional new species remain to be described. From Pakistan, only eight species of *Mycena* have been described based on morpho-anatomical characters. With further fieldwork and the availability of more collections, additional new taxa will undoubtedly be revealed.

Furthermore, the phylogenetic framework of *Mycena* s.l. should be continuously refined and improved based on specimens and sequences from a broader geographic range, particularly through the inclusion of an increasing number of type materials. In recent years, some new genera have been proposed to accommodate some non-amyloid species formerly placed within *Mycena*, e.g., *Atheniella* Redhead, Moncalvo, Vilgalys, Desjardin & B.A. Perry, *Phloeomana* Redhead (Redhead 2012, 2013), *Leucoinocybe* Singer ex Antonín, Borov., Holec & Kolařík (Antonín et al. 2019), and *Pruinomycena* Kun L. Yang, Jia Y. Lin & Zhu L. Yang (Yang et al. 2025).

However, several unresolved clades still persist, and the phylogenetic relationships within the genus *Mycena* remain unclear (Yang et al. 2025). At present, the ITS region remains the most effective DNA barcode for identifying mycenoid fungi. Nevertheless, additional specimens accompanied by multigene data, such as SSU, LSU, *rpb1*, and *rpb2*, are needed to further investigate species diversity and the phylogenetic relationships within *Mycena* s.l. Such data will help clarify the framework of *Mycena* s.l., especially as more sequences from type materials become available.

Mycologists should devote greater attention to material collected from specific habitats. Northwest China contains the largest tracts of virgin forest in the country, which serve as essential refuges for a wide variety of unusual macrofungi.

## Supplementary Material

XML Treatment for Atheniella parvispora

XML Treatment for Mycena albomarginata

XML Treatment for Mycena oblongispora

XML Treatment for Mycena lavendula

XML Treatment for Mycena longnanensis

XML Treatment for Mycena minispora

XML Treatment for Mycena pakistanica

XML Treatment for Mycena tephroleuca

XML Treatment for Phloeomana qilianensis

## Additional information

### Artificial Intelligence (AI) use

The authors accept full responsibility for the content of the manuscript, including the disclosure of any use of AI.

No AI tools were used in the preparation of this manuscript.

### Adherence to national and international regulations

All the fungal strains used in this study have been legally obtained, respecting the Convention on Biological Diversity (Rio Convention).

### Author ORCIDs

L.F. Fan https://orcid.org/0000-0001-9784-758X

A. Naseer https://orcid.org/0000-0002-4458-9043

A. Khalid https://orcid.org/0009-0001-7656-0392

A.N. Khalid https://orcid.org/0000-0002-5635-8031

### Data availability

The sequences generated in this study are deposited in NCBI GenBank (see Table 1).

## Supplementary materials

Supplementary material 1 Alignment for Fig. 1 Data type pdf Explanation note Alignment of ITS and tef1-α used to generate the phylogeny in Fig. 1. https://binary.pensoft.net/file/1713015This dataset is made available under the Open Database License (http://opendatacommons.org/licenses/odbl/1.0/). The Open Database License (ODbL) is a license agreement intended to allow users to freely share, modify, and use this Dataset while maintaining this same freedom for others, provided that the original source and author(s) are credited. Long Fei Fan, Arooj Naseer, Ammara Khalid, Abdul Nasir Khalid, Yan Deng, Chen Yan Wang, Yuan Wang, Si Xuan Ma

Supplementary material 2 Model Test Output 1 Data type pdf Explanation note The result of best-fit evolution model test. https://binary.pensoft.net/file/1713016This dataset is made available under the Open Database License (http://opendatacommons.org/licenses/odbl/1.0/). The Open Database License (ODbL) is a license agreement intended to allow users to freely share, modify, and use this Dataset while maintaining this same freedom for others, provided that the original source and author(s) are credited. Long Fei Fan, Arooj Naseer, Ammara Khalid, Abdul Nasir Khalid, Yan Deng, Chen Yan Wang, Yuan Wang, Si Xuan Ma
