# Supplementary material for: Unveiling the diversity of the families Cyphellaceae and Mycenaceae from Southeast Asia
Source: IMA Fungus. 2026 Jul 10;17:e182174. doi: 10.3897/imafungus.17.182174 (PMC13379713; doi:10.3897/imafungus.17.182174)
Supplement: Supplementary material 1 — Alignment for Fig. 1 [file imafungus-17-e182174-s001.pdf]

152 1474
Mycena_aff_pura_TL8052
ACTTGGTG-TTGA-GCTGGCC-CTA-----GTC--GGGTA-TGTGCTCGCATCAT--ATTA-TTT-A-TCT---ATCTCT
-TGTGCACCTTTTG-TAGTCTTTGAAGTG---
----TTCGCAGTC----------------------AATGCGG---------TAT--------------TGGGT---CTTG
GGC-----------TT-----GCCCTGTTCCCTG--
---------------TTAG------CTTCA---------AGG-----------------------GCTATG-------TT
TA----------------------------TATA-CACTA--GTTTGAAGTCAC-AGAATGTA---TCTTATTGA------
--C-TC---T-CAAGTCAGT--------AAATC-T-ATACAACTTTCAGCAACGGATCTCTTGGCTCTCCCATCGATGAA
GAACGCAGCGAAATGCGATAAGTAATGTGAATTGCAGAATTCAGTGAATCATCGAATCTTTGAACGCACCTTGCGCCCTT
TGGTATTCCGAAGGGCATGCCTGTTTGAGTGTY-ATTATATTC-TCAA---------CCTTGCGA-----AG--------
---CTTGCTTTG--------CTAGGC---TTGGAT-GTGA-GG---G-C-TTTGCTGGC-TTCCA---TTGAGTT-GGAT
GGTCTGCTCCCTTCAAATTTATTAGTGGGA-TCCT--TTGTGGA-----------TGG-TCACTTGG-TGTGATAA-TTA
TCTACGCCGC-CTGACTGTGAAGCAAAACTTGTGGG-GA-CCTGCTTATAACTG------TCTC-TTTG--GGGA-----
---CAAT--CT------TTGACTATTTGACCT-GCCTTCACTCTCGGTGTGCGCCAGCTTATTGTTGCGGTCAACAAGAT
GGACACGACCAAGGTTCG---GTGTGCTTGAA-GTC----------------CTAACGAATAAT------GTTGAT--GA
CACCTATTCACAGTGGAGTGAGGACCGTTTCAACGAAATCGTCAAGGAGACGTCCACTTTCATAAAGAAGGTCGGCTACA
ACCCCAAAACTATTTCTTTCGTCCCTATCTCTGGCTGGCACGG-TGACAACAT--GCTTGAGGAGTCCACGAAGTATGAA
ATTCAGACTTGTATTTCTACCGAATCCAACT-TATGTTTTGCG----------------CAGCATGCCCTGG-TTCAAGG
GCTGGACCAAGGAGAC---GAAAGGTGGT--GTCGTCAAGGGCAAGACTCTTCTT---GAAGCTATCG------------
-ATGCTATTGAGCCCCCGGTCCGGCCTGTGGAC-
Mycena_aff_pura_TL9678
ACTTGGTG-TTGA-GCTGGCC-CTA-----GTT-TGGGCA-TGTGCTCGCATCAT--ATTA-TTT-A-TCT---ATCTCT
-TGTGCACCTTTTG-TAGTCTTTGAAGTG---
----GTCGCAGTC----------------------AATGCGG---------TAT--------------TGGGT---CTTG
GGC-----------TT-----GCCCTTCTCCCTG--
---------------TTAC------CTTCA---------AGG-----------------------GCTATG-------TT
TA----------------------------TACA-CACTA--G-TTGAAGTCAC-AGAATGTA---TCTTATTGA------
--C-CT---T-TGAGTCAGT--------AAATC-T-ATACAACTTTCAGCAACGGATCTCTTGGCTCTCCCATCGATGAA
GAACGCAGCGAAATGCGATAAGTAATGTGAATTGCAGAATTCAGTGAATCATCGAATCTTTGAACGCACCTTGCGCCCTT
TGGTATTCCGAAGGGCATGCCTGTTTGAGTGTC-ATTATATTC-TCAA---------CCTTGCAG-----AG--------
---CTTGCTTTG--------CTAGGC---TTGGAT-GTGA-GG---G-CTTTTGCTGGC-TTCCA---TTGAGTT-GGAT
GGTCTGCTCCCTTCAAATTTATTAGTGGGA-TCCT--TTGTGGA-----------TGG-TCACTTGG-TGTGATAA-TTA
TCTACGCCGC-TTGACTGTGAAGCAAGACTTGTGGG-AA-CCTGCTTATAACTG------TCTC-TTCG--GGGA-----
---CTAT--CT------TTGACTATTTGACCT--------------GTGTGCGCCAGCTTATTGTTGCGGTCAATAAGAT
GGACACYACCAAGGTTCG---GTGTACTTGAA-GTC----------------CTAACGAATAAT------TTCGAT--GA
CACCTATTCACAGTGGAGCGAGGACCGTTTCAACGAAATCGTCAAGGAGACGTCCACTTTCATAAAGAAGGTTGGCTACA
ACCCCAAAACTATTTCTTTCGTCCCTATCTCTGGCTGGCACGG-TGACAACAT--GCTTGAGGAGTCGACGAAGTATGAA
GTTCAGATTTGTATT-----CGAATCCAGCT-TATGTTTTGCG----------------CAGCATGCCTTGG-TTCAAGG
GCTGGACCAAGGAGAC---GAAAGGTGGT--GTCGTYAAGGGCAAGACTCTTCTT---GAAGCTATYG------------
-ATGCCATTGAGCCCCCGATCCGGCCTGTGGAC-
Mycena_aff_pura_TL9433
ACTTGGTG-TTGA-GCTGGCC-CTA-----GTC--GGGCA-TGTGCTTGCATCAT--ATTA-TTT-A-TCT---ATCTCT
-TGTGCATCTTTTG-TAGTCTTTGAAGCG---

----TTCGCAGTT----------------------GATGCGG---------TAT--------------TGGGT---CTTG
GGG-----------TTT--A-GCCCTGTTCCCTG--
---------------TTAG------CTTCA---------AGG-----------------------GCTATG-------TT
TAT---------------------------TACA-CACTA--G-TTGAAGTCAC-AGAATGTA---TCTTATTGA-----
--C-TT---T-TGGGTCAGT--------AAATC-C-ATACAACTTTCAGCAACGGATCTCTTGGCTCTCCCATCGATGAA
GAACGCAGCGAAATGCGATAAGTAATGTGAATTGCAGAATTCAGTGAATCATCGAATCTTTGAACGCACCTTGCGCCCTT
TGGTATTCCGAAGGGCATGCCTGTTTGAGTGTC-ATTATATTC-TCAA---------CCTTGTGA-----AG--------
---CTTGCTTCA--------CTAGGC---TTGGATGGTGA-GG---G-C-TTTGCTGGC-TTCCA---TTGAGTT-GGAT
GGTCTGCTCCCTTTAAATTTATTAGTGGGA-TCCT--TTGTGGA-----------TGG-TCACTTGG-TGTGATAA-TTA
TCTACGCCGC-CTGAC--TGAAGCAAGACTTGTGGG-AA-CCTGCTTATAACCG------TCTT-CTCG--GAGA-----
---CTAT--CT------TTGACTATTTGACCT-GCCTTCACTCTCGGTGTGCGCCAGCTTATTGTTGCGGTCAACAAGAT
GGACACGACCAAGGTTCG---GTGTACTTGAA-GTC----------------CTAACGAATAAT------TTTCAT--GA
CACCTATTCACAGTGGAGTGAGGATCGTTTCAACGAAATCGTMAAGGAGACGTCCACTTTCATAAAGAAGGTCGGCTACA
ACCCCAAAGCTATTTCTTTCGTCCCTATCTCTGGCTGGCACGG-TGACAACAT--GCTTGAGGAGTCCACGAAGTATGAA
GTTCAGATTTGTATTTCTACCGAATCCAACT-TATGTTTTGCG----------------CAGCATGCCCTGG-TTCAAGG
GCTGGACCAAGGAGAC---GAAAAGTGGT--GTCGTCAAGGGCAAGACTCTTCTT---GAAGCTATCG------------
-ATGCTATTGAGCCCCCGGTCCGGCCTACGGAC-
Mycena_aff_pura_TL9450
ACTTGGTG-TTGA-GCTGGCC-CTA-----GTC--GGGCA-TGTGCTTGCATCAT--ATTA-TTT-A-TCT---ATCTCT
-TGTGCATCTTTTG-TAGTCTTTGAAGCG--
----TTCGCAGTT----------------------GATGCGG---------TAT--------------TGGGT---CTTG
GGG-----------TTT--A-GCCCTGTTCCCTG--
---------------TTAG------CTTCA---------AGG-----------------------GCTATG-------TT
TAT---------------------------TACA-CACTA--G-TTGAAGTCAC-AGAATGTA---TCTTATTGA-----
--C-TT---T-TGGGTCAGT--------AAATC-C-ATACAACTTTCAGCAACGGATCTCTTGGCTCTCCCATCGATGAA
GAACGCAGCGAAATGCGATAAGTAATGTGAATTGCAGAATTCAGTGAATCATCGAATCTTTGAACGCACCTTGCGCCCTT
TGGTATTCCGAAGGGCATGCCTGTTTGAGTGTC-ATTATATTC-TCAA---------CCTTGTGA-----AG--------
---CTTGCTTCA--------CTAGGC---TTGGATGGTGA-GG---G-C-TTTGCTGGC-TTCCA---TTGAGTT-GGAT
GGTCTGCTCCCTTTAAATTTATTAGTGGGA-TCCT--TTGTGGA-----------TGG-TCACTTGG-TGTGATAA-TTA
TCTACGCCGC-CTGAC--TGAAGCAAGACTTGTGGG-AA-CCTGCTTATAACCG------TCTT-CTCG--GAGA-----
---CTAT--CT------TTGACTATTTGACCT-GCCTTCACTCTCGGTGTGCGCCAGCTTATTGTTGCGGTCAACAAGAT
GGACACGACCAAGGTTCG---GTGTACTTGAA-GTC----------------CTAACGAATAAT------TTTCAT--GA
CACCTATTCACAGTGGAGTGAGGATCGTTTCAACGAAATCGTCAAGGAGACGTCCACTTTCATAAAGAAGGTCGGCTACA
ACCCCAAAGCTATTTCTTTCGTCCCTATCTCTGGCTGGCACGG-TGACAACAT--GCTTGAGGAGTCCACGAAGTATGAA
GTTCAGATTTGTATTTCTACCGAATCCAACT-TATGTTTTGCG----------------CAGCATGCCCTGG-TTCAAGG
GCTGGACCAAGGAGAC---GAAAAGTGGT--GTCGTCAAGGGCAAGACTCTTCTT---GAAGCTATCG------------
-ATGCTATTGAGCCCCCGGTCCGGCCTACGGAC-
Mycena_luxmanantlanensis_ACP2160
ACTTGGTG-TTGA-GCTGGCC-CTA-----GTG--GGGCA-TGTGCTTGCATCAT--ATTA-TTT-A-TCT---ATCTCT
-TGTGCATCTTTTG-TAGTCTTTGAAGCG--
----TTCGCAGTTG---------------------AATGCGG---------TAT--------------TGGGT---CTTG
GGC-----------TT-----GCCCTGTTCCCTG--
---------------TTAG------CTTCA---------AGG-----------------------GCTATG-------TT
TA----------------------------TACA-CACTA--G-TTGAAGTCAC-AGAATGTA---TCTTATTGAC----

-TC-TT---T-TGAGTCGGT--------AAATC-C-ATACAACTTTCAGCAACGGATCTCTTGGCTCTCCCATCGATGAA
GAACGCAGCGAAATGCGATAANNNNNNNNATTGCAGAATTCAGTGAATCATCGAATCTTTGAACGCACCTTGCGCCCTT
TGGTATTCCGAAGGGCATGCCTGTTTGAGTGTCAATTATATTC-TCAA---------CCTTGTGA-----AG--------
---CTTGCTTCG--------CTAGGC---TTGGAT-GTGA-GG---G-C-TTTGCTGGC-TTCCA---TTGAGTT-GGAT
GGTCTGCTCCCTTCAAATTTATTAGTGGGA-TCCT--TTGTGGA-----------TGG-TCACTTGG-TGTGATAA-TTA
TCTACGCCGC-CTGAC--TGAAGCAAGACTTGTGGG-AA-CCTGCTTATAACCG------TCTT-CTTG--GAGA-----
---CTAT--CT-----TTTGACTATTTGACCT-GCCTTCACTCTCGGTGTGCGCCAGCTTATTGTTGCGGTCAACAAGAT
GGACACGGCCAAGGTTCG---GTGTACTTGAA-GTC----------------CTAACGAATGAT------TTTCAT--GA
CA-CTATTCACAGTGGAGTGAGGACCGTTTCAACGAAATCGTCAAGGAGACGTCCACTTTCATAAAGAAGGTCGGCTACA
ACCCCAAAGCGATTTCTTTCGTCCCTATCTCCGGCTGGCACGG-TGACAACAT--GCTTGAGGAGTCCACGAAGTATGAA
GTTCAGATTTGCATTTCTACCGAATCCAACT-GATGTTTTGCG----------------CAGCATGCCGTGG-TTCAAGG
GCTGGACTAAGGAGAC---GAAAGGCGGT--GTCGTCAAGGGCAAGACTCTTCTT---GAAGCTATCG------------
-ATGCTATTGAGCCCCCGGTCCGGCCTGTGGAC-
Mycena_luxmanantlanensis_ACP2159
ACTTGGTG-TTGA-GCTGGCC-CTA-----GTG--GGGCA-TGTGCTTGCATCAT--ATTA-TTT-A-TCT---ATCTCT
-TGTGCATCTTTTG-TAGTCTTTGAAGCG---
----TTCGCAGTTG---------------------AATGCGG---------TAT--------------TGGGT---CTTG
GGC-----------TT-----GCCCTGTTCCCTG--
---------------TTAG------CTTCA---------AGG-----------------------GCTATG-------TT
TA----------------------------TACA-CACTA--G-TTGAAGTCAC-AGAATGTA---TCTTATTGAC----
-TC-TT---T-TGAGTCGGT--------AAATC-C-ATACAACTTTCAGCAACGGATCTCTTGGCTCTCCCATCGATGAA
GAACGCAGCGAAATGCGATAANNNNNNNAATTGCAGAATTCAGTGAATCATCGAATCTTTGAACGCACCTTGCGCCCTT
TGGTATTCCGAAGGGCATGCCTGTTTGAGTGTCAATTATATTC-TCAA---------CCTTGTGA-----AG--------
---CTTGCTTCG--------CTAGGC---TTGGAT-GTGA-GG---G-C-TTTGCTGGC-TTCCA---TTGAGTT-GGAT
GGTCTGCTCCCTTCAAATTTATTAGTGGGA-TCCT--TTGTGGA-----------TGG-TCACTTGG-TGTGATAA-TTA
TCTACGCCGC-CTGAC--TGAAGCAAGACTTGTGGG-AA-CCTGCTTATAACCG------TCTT-CTTG--GAGA-----
---CTAT--CT-----TTTGACTATTTGACCT-GCCTTCACTCTCGGTGTGCGCCAGCTTATTGTTGCGGTCAACAAGAT
GGACACGGCCAAGGTTCG---GTGTACTTGAA-GTC----------------CTAACGAATGAT------TTTCAT--GA
CA-CTATTCACAGTGGAGTGAGGACCGTTTCAACGAAATCGTCAAGGAGACGTCCACTTTCATAAAGAAGGTCGGCTACA
ACCCCAAAGCGATTTCTTTCGTCCCTATCTCCGGCTGGCACGG-TGACAACAT--GCTTGAGGAGTCCACGAAGTATGAA
GTTCAGATTTGCATTTCTACCGAATCCAACT-GATGTTTTGCG----------------CAGCATGCCGTGG-TTCAAGG
GCTGGACTAAGGAGAC---GAAAGGCGGT--GTCGTCAAGGGCAAGACTCTTCTT---GAAGCTATCG------------
-ATGCTATTGAGCCCCCGGTCCGGCCTGTGGAC-
Mycena_dura_10315
ACTTGGTG-TTGA-GCTGGCC-CTA------TT--GGGCA-TGTGCTTGCATCAT--ATTA-TTT-A-TCT---ATCTCT
-TGTGCACCTTTTG-TAGTCTTTGAAGCG---
---TTTCGCAGTC----------------------AATGCGG---------TTT--------------TGGGA---CCTG
GGC-----------TTTGCT-GTCCTTTTCCCTG--
---------------TTTG------CTTCA---------AAG-----------------------GCTATG-----TTTT
TA----------------------------TACA-CACTA----TTAAAGTCAC-AGAATGTC---TCTTATTGA-----
--C-TT---T-CGAGTCAGT--------AAATC-T-ATACAACTTTCAGCAACGGATCTCTTGGCTCTCCCATCGATGAA
GAACGCAGCGAAATGCGATAAGTAATGTGAATTGCAGAATTCAGTGAATCATCGAATCTTTGAACGCACCTTGCGCCCTT
TGGTATTCCGAAGGGCATGCCTGTTTGAGTGTC-ATTAAATTC-TCAA---------CCTTGCAA-----GC--------
----TTGC-TTG--------CGAGGC---TTGGAT-GTGA-GG---G-TATTTGCTGGC-TTCC----TTCATTT-GGAT

GGTCTGCTCCCTTTAAATTTATTAGTGGAA-TCCT--TTGTGGA-----------TGG-TCACTTGG-TGTGATAA-TTA
TCTACGCCGC-CTGACTATGAAACAAGACTTGTGGG-AA-CCCGCTTATAACCG------TCTC-TCGA--G--------
-ACTTAT--C-------TTGACAATTTGACCT-GCCTTCACTCTTGGTGTGCGCCAGCTTATCGTTGCGGTCAACAAGAT
GGACACGACCAAGGTTCG---GTGCATTATAA-GCC----------------CCAACGAATAAT-------CTGAT--CA
CACCTGTTCACAGTGGAGTGAGGACCGTTTCAACGAAATCATCAAGGAGACATCCACTTTCATTAAGAAGGTCGGCTACA
ACCCCAAAGCTATTTCTTTCGTCCCTATTTCTGGCTGGCACGG-TGACAACAT--GCTTGAGGAGTCCACGAAGTATGAA
GTTCAGATTTGTATTTTTACCGA-CCCAACT-TATG-TTTGCG----------------CAGCATGCCCTGG-TTCAAGG
GCTGGACCAAGGAGAC---CAAAGCCGGT--GTCGTCAAGGGCAAGACTCTTCTT---GAAGCCATCG------------
-ATGCTATTGAGCCCCCGGTTCGGCCTGTGGAC-
Mycena_rosea_CBH097
ACTTGGTG-TTGA-GCTGGCC-CTA------TT--GGGCA-TGTGCTCGCATCAT--ATTATTTT-A-TCT---ATCTCT
-TGTGCACCTTTTG-TAGTCTTTGAAGTG---
----TTCGCAGTC----------------------AATGCGG---------TTT--------------TGGGT---CTTG
GGC-----------TTTGCT-GCCCTTTTCCCTG--
---------------TTTG------CTTCA---------AAG-----------------------GCTATG-----TTTT
TA----------------------------TATA-CACTA----TTAAAGTCAC-AGAATGTC---TCTTATTGA-----
--C-TT---C-CGAGTCGGT--------AAATC-T-ATACAACTTTCAGCAACGGATCTCTTGGCTCTCCCATCGATGAA
GAACGCAGCGAAATGCGATAAGTAATGTGAATTGCAGAATTCAGTGAATCATCGAATCTTTGAACGCACCTTGCGCCCTT
TGGTATTCCGAAGGGCATGCCTGTTTGAGTGTC-ATTAAATTC-TCAA---------CCTTGCAA-----GC--------
--TTTTGC-TTG--------TGAGGC---TTGGAT-GTGA-GG---G-TTTTTGCTGGC-TTCC----TTCATTT-GGAT
GGTCTGCTCCCTTTAAATTTATTAGTGGGA-TCCT--TTGTGGA-----------TGG-TCACTTGG-TGTGATAA-TTA
TCTACGCCGC-CTGACTCTGATACAAGACTTGTGGG-AA-CCCGCTTATAACCG------TCTC-TTCA--GAGAGACA-
-ACTTAT--A-------TTGACAATTTGACCT-GCCTTCACTCTCGGTGTGCGCCAGCTTATCGTTGCGGTCAACAAGAT
GGACACGACCAAGGTTCG---GTACATTATAA-ACC----------------CTAACGAATAAT------TTTGAT--GA
CACCTGTTTACAGTGGAGTGAGGACCGTTTCAACGAAATCGTCAAGGAGACGTCGACTTTCATTAAGAAGGTCGGCTACA
ACCCCAAATCTATTTGTTTCGTCCCTATTTCTGGCTGGCACGG-TGACAACAT--GCTTGAGGAGTCTACGAAGTATGAA
GTTCAGA-TTGTATTTCTACCGAACCCAACT-TATG-TTTGCG----------------CAGCATGCCCTGG-TTCAAGG
GCTGGACCAAGGAGAC---CAAAGGTGGT--GTCGTCAAGGGCAAGACTCTGCTT---GAAGCTATCG------------
-ATGCTATTGAGTCCCCGACCCGGCCTACGGAC-
Mycena_rosea_CBH409
ACTTGGTG-TTGA-GCTGGCC-CTA------TT--GGGCA-TGTGCTCGCATCAT--ATTATTTT-A-TCT---ATCTCT
-TGTGCACCTTTTG-TAGTCTTTGAAGTG---
----TTCGCAGTC----------------------AATGCGG---------TTT--------------TGGGT---CTTG
GGC-----------TTTGCT-GCCCTTTTCCCTG--
---------------TTTG------CTTCA---------AAG-----------------------GCTATG-----TTTT
TA----------------------------TACA-CACTA----TTAAAGTCAC-AGAATGTC---TCTTATTGA-----
--C-TT---C-CGAGTCAGT--------AAATC-T-ATACAACTTTCAGCAACGGATCTCTTGGCTCTCCCATCGATGAA
GAACGCAGCGAAATGCGATAAGTAATGTGAATTGCAGAATTCAGTGAATCATCGAATCTTTGAACGCACCTTGCGCCCTT
TGGTATTCCGAAGGGCATGCCTGTTTGAGTGTC-ATTAAATTC-TCAA---------CCTTGCAA-----GC--------
--TTTTGC-TTG--------TGAGGC---TTGGAT-GTGA-GG---G-TTTTTGCTGGC-TTCC----TTCATTT-GGAT
GGTCTGCTCCCTTTAAATTTATTAGTGGGA-TCCT--TTGTGGA-----------TGG-TCACTTGG-TGTGATAA-TTA
TCTACGCCGC-CTGACTCTGATACAAGACTTGTGGG-AA-CCCGCTTATAACCG------TCTC-TTCA--GAGAGACA-
-ACTTAT--A-------TTGACAATTTGACCT-GCCTTCACTCTCGGTGTGCGCCWRCTTATCGTTGCGGTCAACAAGAT
GGACACGACCAAGGTTCG---GTACATTATAA-ACC----------------CTAACGAATAAT------TTTGAT--GA

CACCTGTTTACAGTGGAGTGAGGACCGTTTCAACGAAATCGTCAAGGAGACGTCGACTTTCATTAAGAAGGTCGGCTACA
ACCCCAAATCTATTTGTTTCGTCCCTATTTCTGGCTGGCACGG-TGACAACAT--GCTTGAGGAGTCTACGAAGTATGAA
GTTCAGA-TTGTATTTCTACCGAACCCAACT-TATG-TTTGCG----------------CAGCATGCCCTGG-TTCAAGG
GCTGGACCAAGGAGAC---CAAAGGTGGT--GTCGTCAAGGGCAAGACTCTGCTT---GAAGCTATCG------------
-ATGCTATTGAGTCCCCGACCCGGCCTACGGAC-
Mycena_seminau_ACL136
ACTTGGTG-TTGA-GCTGGCC-CTA------TT--GGGCA-TGTGCTTGCATCAT--ATTA-TTT-A-TCT---ATCTCT
-TGTGCACCTTTTG-TAGTCTTTGAAGTG---
----TTCGCAGTC----------------------GATGCGG---------TTT--------------TGGGG---CTTG
GGC-----------TC---T-GCCCTCTTCCCTG--
---------------TTTG------CTTCA---------AAG-----------------------GCTATGT----TTTT
TA----------------------------CACA-CACTA----TTGAAGTTAC-AGAATGTC---TCTTATTGA-----
--C-TT---T-CGAGTCAGT--------AAATC-T-ATACAACTTTCAGCAACGGATCTCTTGGCTCTCCCATCGATGAA
GAACGCAGCGAAATGCGATAAGTAATGTGAATTGCAGAATTCAGTGAATCATCGAATCTTTGAACGCACCTTGCGCCCTT
TGGTATTCCGAAGGGCATGCCTGTTTGAGTGTC-ATTAAATTC-TCAA---------CCTCGCAA-----GC--------
----TTGCTTTG--------CGAGGC---TTGGAT-GTGA-GG---G-TTTTTGCTGGC-TTCC----TTCAGTT-GGAT
GGTCTGCTCCCTTGAAATTCATTAGTGGGA-TCCT--TTGTGGA-----------TGG-TCACTTGG-TGTGATAA-TTA
TCTACGCCGC-CTGACTCTGAAACAAGACTTGTGGG-AA-CCTGCCTATAACCG------TCTC-TTTC--AAGAGACAA
-GCTTAT--C-------TTGACAATTTGACCT--

Mycena_seminau_ACL308
ACTTGGTG-TTGA-GCTGGCC-CTA------TT--GGGCA-TGTGCTTGCATCAT--ATTA-TTT-A-TCT---ATCTCT
-TGTGCACCTTTTG-TAGTCTTTGAAGTG---
----TTCGCAGTC----------------------RATGCGG---------TTT--------------TGGGG---CTTG
GGC-----------TC---T-GCCCTYTTCCCTG--
---------------TTTG------CTTCA---------AAG-----------------------GCTATG------TTT
TA----------------------------CACA-CACTA----TTGAAGTTAC-AGAATGTC---TCTTATTGA-----
--C-TT---T-CGAGTCAGT--------AAATC-T-ATACAACTTTCAGCAACGGATCTCTTGGCTCTCCCATCGATGAA
GAACGCAGCGAAATGCGATAAGTAATGTGAATTGCAGAATTCAGTGAATCATCGAATCTTTGAACGCACCTTGCGCCCTT
TGGTATTCCGAAGGGCATGCCTGTTTGAGTGTC-ATTAAATTC-TCAA---------CCTCGYAA-----GC--------
----TTGCTTTG--------CGAGGC---TTGGAT-GTGA-GG---G-TTTTTGCTGGC-TTCC----TTCAGTT-GGAT
GGTCTGCTCCCTTGAAATTCATTAGTGGGA-TCCT--TTGTGGA-----------TGG-TCACTTGG-TGTGATAA-TTA
TCTACGCCGC-CTGACTCTGAAACAAGACTTGTGGG-AA-CCTGCTTATAACTG------TCTC-TTTC--AAGAGACAA
-GCTTAT--C-------TTGACAATTTGACCT--

Mycena_sinar_ACL092

ACTTGGTG-TTGA-GCTGGCC-CTA------TT--GGGCA-TGTGCTCGCATCAT--ATTA-TTT-A-TCT---ATCTCT
-TGTGCACCTTTTG-TAGTCTTTGAAGTG---
----TTCGCAGTC----------------------AATGCGG---------TTT--------------TGGGG---CTTG
GGC-----------TCTTTT-GCTCTCTTCCCTG--
---------------TTTG------CTTCA---------AAG-----------------------GCTATG-----TTTT
TA----------------------------TACA-CACTA----TTGAAGTTAC-AGAATGTCC--TCTTATTGA-----
--C-TT---T-CGAGTCAGT--------AAATC-T-ATACAACTTTCAGCAACGGATCTCTTGGCTCTCCCATCGATGAA
GAACGCAGCGAAATGCGATAAGTAATGTGAATTGCAGAATTCAGTGAATCATCGAATCTTTGAACGCACCTTGCGCCCTT
TGGTATTCCGAAGGGCATGCCTGTTTGAGTGTC-ATTAAATTC-TCAA---------CCTTGCAA-----GC--------
----TTGC-TTG--------CGAGGC---TTGGAT-GTGA-GG---G-TTTTTGCTGGC-TTCC----TTCAGTT-GGAT
GGTCTGCTCCCTTCAAATTTATTAGTGGGA-TCCT--TTGTGGA-----------TGG-TCACTTGG-TGTGATAA-TTA
TCTACGCCGC-CTGACTCTGACACAAGACTTGTGGG-AA-CCTGCTTATAACCG------TCTC-TTC---GAGAGACAA
-GCTTAC--CT------TTGACAATTTGACCT--

Mycena_sinar_ACL135

ACTTGGTG-TTGA-GCTGGCC-CTA------TT--GGGCA-TGTGCTCGCATCAT--ATTA-TTT-A-TCT---ATCTCT
-TGTGCACCTTTTG-TAGTCTTTGAAGTG---
----TTCGCAGTC----------------------AATGCGG---------TTT--------------TGGGG---CTTG
GGC-----------TCTTTT-GCTCTCTTCCCTG--
---------------TTTG------CTTCA---------AAG-----------------------GCTATG-----TTTT
TA----------------------------TACA-CACTA----TTGAAGTTAC-AGAATGTCC--TCTTATTGA-----
--C-TT---T-CGAGTCAGT--------AAATC-T-ATACAACTTTCAGCAACGGATCTCTTGGCTCTCCCATCGATGAA
GAACGCAGCGAAATGCGATAAGTAATGTGAATTGCAGAATTCAGTGAATCATCGAATCTTTGAACGCACCTTGCGCCCTT
TGGTATTCCGAAGGGCATGCCTGTTTGAGTGTC-ATTAAATTC-TCAA---------CCTTGCAA-----GC--------
----TTGC-TTG--------CGAGGC---TTGGAT-GTGA-GG---G-TTTTTGCTGGC-TTCC----TTCAGTT-GGAT
GGTCTGCTCCCTTCAAATTTATTAGTGGGA-TCCT--TTGTGGA-----------TGG-TCACTTGG-TGTGATAA-TTA
TCTACGCCGC-CTGACTCTGACACAAGACTTGTGGG-AA-CCTGCTTATAACCG------TCTC-TTC---GAGAGACAA
-GCTTAC--CT------TTGACAATTTGACCT--

Mycena_sinar_var_tangkaisinar_ACL307

ACTTGGTG-TTGA-GCTGGCC-CTA------TT--GGGCA-TGTGCTCGCATCAT--ATTA-TTT-A-TCT---ATCTCT
-TGTGCACCTTTTG-TAGTCTTTGAAGTG---

----TTCGCAGTCA---------------------AATGCGG---------TTT--------------TGGGG---CTTG
GGC-----------TCTTTT-GCTCTCTTCCCTG--
---------------TTTG------CTTCA---------AAG-----------------------GCTATG-----TTTT
TA----------------------------TACA-CACTA----TTGAAGTTAC-AGAATGTCC--TCTTATTGA-----
--C-TT---T-CGAGTCAGT--------AAATC-T-ATACAACTTTCAGCAACGGATCTCTTGGCTCTCCCATCGATGAA
GAACGCAGCGAAATGCGATAAGTAATGTGAATTGCAGAATTCAGTGAATCATCGAATCTTTGAACGCACCTTGCGCCCTT
TGGTATTCCGAAGGGCATGCCTGTTTGAGTGTC-ATTAAATTC-TCAA---------CCTTGCAA-----GC--------
----TTGC-TTG--------CGAGGC---TTGGAT-GTGA-GG---G-TTTTTGCTGGC-TTCC----TTCAGTT-GGAT
GGTCTGCTCCCTTCAAATTTATTAGTGGGA-TCCT--TTGTGGA-----------TGG-TCACTTGG-TGTGATAA-TTA
TCTACGCCGC-CTGACTCTGACACAAGACTTGTGGG-AA-CCTGCTTATAACCG------TCTC-TTC---GAGAGACAA
-GCTTAC--CT------TTGACAATTTGACCT--

Mycena_rufobrunnea_FFAAS0415
ACTTGGTG-TTGA-GCTGGCC-CTG------TT--GGGCA-TGTGCTTGCATCAT--ATTA-TTT-A-TCT---ATCTCT
-TGTGCACCTTTTG-TAGTCTTTGAAGCG---
----TTCGCAGTC----------------------AATGCGG---------TTT--------------TGGGG---CTTG
GGC-----------TT---T-GCCCTCTTCCCTG--
---------------TTTG------CTTCA---------AAG-----------------------GCTATG-----TTTT
TA----------------------------TACA-CACTA----TTGAAGTTAC-AGAATGTC---TCTTATTGA-----
--C-TT---T-TGAGTCAGT--------AAATC-T-ATACAACTTTCAGCAACGGATCTCTTGGCTCTCCCATCGATGAA
GAACGCAGCGAAATGCGATAAGTAATGTGAATTGCAGAATTCAGTGAATCATCGAATCTTTGAACGCACCTTGCGCCCTT
TGGTATTCCGAAGGGCATGCCTGTTTGAGTGTC-ATTAAATTC-TCAA---------CCTTGCAA-----GC--------
----TTGC-TTG--------CAAGGC---TTGGAT-GTGA-GG---G-TTTTTGCTGGC-TTCC----TTCAGTTGGGAT
GGTCTGCTCCCTTTAAATTCATTAGTGGGA-TCCT--TTGTGGA-----------TGG-TCACTTGG-TGTGATAG-TTA
TCTACGCCCC-CTGACTCTGAAACAAAACTTATGGG-AA-CCTGCTTATAACCG------TCTC-TTG---AAGAGACAA
-GCTTAT--C-------TTGACAATTTGACCT-GCCTTCACTCTCGGTGTGCGTCAGCTCATCGTTGCGGTGAACAAGAT
GGACACGACCAAGGTTCG---GTGCATTATAA-GCC---------------ACTAACGGATGAT------TTTGAT--GA
CACCTATTCACAGTGGAGCGAGGACCGTTTCAACGAAATCATCAAGGAGACGTCCACCTTCATTAAGAAGGTCGGCTACA
ACCCCAAATCTATTTCTTTCGTCCCTATTTCTGGCTGGCACGG-TGACAACAT--GCTTGAGGAGTCCACGAAGTATGAA
GTTTAGATTTTTATTTCTACTGAACCCAACT-TATG-TTTGCG----------------CAGCATGCCCTGG-TTCAAGG
GCTGGACCAAGGAGAC---CAAAGCTGGT--GTCGTCAAGGGCAAGACTCTTCTC---GAAGCTATCG------------
-ATGCTATCGAGCCCCCGATTCGGCCCAATGAC-
Mycena_rufobrunnea_FFAAS0416
ACTTGGTG-TTGA-GCTGGCC-CTG------TT--GGGCA-TGTGCTTGCATCAT--ATTA-TTT-A-TCT---ATCTCT
-TGTGCACCTTTTG-TAGTCTTTGAAGCG---
----TTCGCAGTC----------------------AATGCGG---------TTT--------------TGGGG---CTTG
GGC-----------TT---T-GCCCTCTTCCCTG--
---------------TTTG------CTTCA---------AAG-----------------------GCTATG-----TTTT
TA----------------------------TACA-CACTA----TTGAAGTTAC-AGAATGTC---TCTTATTGA-----

--C-TT---T-TGAGTCAGT--------AAATC-T-ATACAACTTTCAGCAACGGATCTCTTGGCTCTCCCATCGATGAA
GAACGCAGCGAAATGCGATAAGTAATGTGAATTGCAGAATTCAGTGAATCATCGAATCTTTGAACGCACCTTGCGCCCTT
TGGTATTCCGAAGGGCATGCCTGTTTGAGTGTC-ATTAAATTC-TCAA---------CCTTGCAA-----GC--------
----TTGC-TTG--------CAAGGC---TTGGAT-GTGA-GG---G-TTTTTGCTGGC-TTCC----TTCAGTTGGGAT
GGTCTGCTCCCTTTAAATTCATTAGTGGGA-TCCT--TTGTGGA-----------TGG-TCACTTGG-TGTGATAG-TTA
TCTACGCCCC-CTGACTCTGAAACAAAACTTATGGG-AA-CCTGCTTATAACCG------TCTC-TTG---AAGAGACAA
-GCTTAT--C-------TTGACAATTTGACCT-GCCTTCACTCTCGGTGTGCGTCAGCTCATCGTTGCGGTGAACAAGAT
GGACACGACCAAGGTTCG---GTGCATTATAA-GCC---------------ACTAACGGATGAT------TTTGAT--GA
CACCTATTCACAGTGGAGCGAGGACCGTTTCAACGAAATCATCAAGGAGACGTCCACCTTCATTAAGAAGGTCGGCTACA
ACCCCAAATCTATTTCTTTCGTCCCTATTTCTGGCTGGCACGG-TGACAACAT--GCTTGAGGAGTCCACGAAGTATGAA
GTTTAGATTTTTATTTCTACTGAACCCAACT-TATG-TTTGCG----------------CAGCATGCCCTGG-TTCAAGG
GCTGGACCAAGGAGAC---CAAAGCTGGT--GTCGTCAAGGGCAAGACTCTTCTC---GAAGCTATCG------------
-ATGCTATCGAGCCCCCGATTCGGCCCAATGAC-
Mycena_cf_pura_VI_BAP132
ACTTGGTG-TTGA-GCTGGCC-CTA------TT--GGGCA-TGTGCTCGCATCAT--ATTA-TTT-A-TCT---ATCTCT
-TGTGCACCTTTTG-TAGTCTTTGGAGTG---
----TTCGCAGTC----------------------AATGCGG---------TTT--------------TGGGG---CTTG
GGC-----------TT---T-GCCCTTTTCCCTG--
---------------TTTG------CTTCA---------AAG-----------------------GCTATG-----TTTT
TA----------------------------TACA-CACTA----TTGAAGTTAC-AGAATGTC---TCTTATTGA------
--C-TT---T-CGAGTCAGT--------AAATC-T-ATACAACTTTCAGCAACGGATCTCTTGGCTCTCCCATCGATGAA
GAACGCAGCGAAATGCGATAAGTAATGTGAATTGCAGAATTCAGTGAATCATCGAATCTTTGAACGCACCTTGCGCCCTT
TGGTATTCCGAAGGGCATGCCTGTTTGAGTGTC-ATTAAATTC-TCAA---------CCTTGCAA-----GC--------
----TTGC-TTG--------CGAGGC---TTGGAT-GTGA-GG---G-TTTTTGCTGGC-TTCCT---TTCAGTT-GGAT
GGTCTGCTCCCTTTAAATTCATTAGTGGGA-TCCT--TTGTGGA-----------TGG-TCACTTGG-TGTGATAA-TTA
TCTACGCCGC-CTGACTCTGAAACAAGACTTGTGGG-AA-CCTGCTTATAACCG------TCTC-TTTC--AAGAGACAA
-GCTTAT--C-------TTGACAATTTGACCT---------------TGTGCGCCAGCTTATCGTTGCGGTCAACAAGAT
GGACACGACCAAGGTTCG---GTGCATTATAA-GCC----------------CCAACGAATAAT------TTTGAT--GA
CACCTATTCACAGTGGAGCGAAGACCGTTTCAACGAAATCGTTAAGGAGACGTCCACTTTCATTAAGAAGGTCGGCTACA
ACCCCAAATCTATTTCTTTCGTCCCTATCTCTGGCTGGCACGG-TGACAACAT--GCTTGAGGAGTCCACGAAGTATGAA
GTTCAGATTTGTATTTCTGCCGAACCTAACT-TACG-TTTGCG----------------CAGCATGCCCTGG-TTCAAGG
GCTGGACCAAGGAGAC---CAAAGGTGGT--GTCGTCAAGGGCAAGACTCTTCTT---GAAGCTATCG------------
-ATGCTATCGAGCCCCCGGTCCGGCCTGTGGAC-
Mycena_glabra_FLF449
ACTTGGTG-TTGA-GCTGGCC-CTT------TT--GGGCA-TGTGCTTGCATCAT--ATTA-TTT-A-TCT---ATCTCT
-TGTGCACCTTTTG-TAGTCTTTGAAGTG---
----TTCGCAGTC----------------------AATGCGG---------TTT--------------TGGGG---CTTG
GGC-----------TT---T-GCCCTCTTCCCTG--
---------------TTTG------CTTCA---------AAG-----------------------GCTATG-----TTTT
TA----------------------------TACA-CACTA----TCAAAGTTAC-AGAATGTC---TCTTATTGA------
--C-TT---T-TGGGTCAGT--------AAATCTT-ATACAACTTTCAGCAACGGATCTCTTGGCTCTCCCATCGATGAA
GAACGCAGCGAAATGCGATAAGTAATGTGAATTGCAGAATTCAGTGAATCATCGAATCTTTGAACGCACCTTGCGCCCTT
TGGTATTCCGAAGGGCATGCCTGTTTGAGTGTC-ATTAAATTC-TCAA---------CCTCGCAA-----GC--------
----TTGCTTTGC-------CGAGGC---TTGGAT-GTGA-GG---G-TTTTTGCTGGC-TTCC----TTCAGTT-GGAT

GGTCTGCTCCCTTTAAATTCATTAGTGGGA-TCCT--TTGTGGA-----------TGG-TCACTTGG-TGTGATAA-TTA
TCTACGCCGC-CTGACTCTGAAACAAGACTTATGGG-AA-CCTGCTTATAACTG------TCTC-TTC---AAGAGACAA
-GCTTAT--C-------TTGACAATTTGACCT-GCCTTCACTCTCGGTGTGCGCCAGCTCATCGTTGCGGTGAACAAGAT
GGACACGACCAAGGTTCG---GTGCATTATAA-GCC---------------ACTAACGAATAA--------TTGAT--GA
CACCTATTCATAGTGGAGTGAGGACCGCTTCAACGAAATCATCAAGGAGACGTCCACCTTCATTAAGAAGGTCGGCTACA
ACCCCAAATCGATTTCTTTCGTCCCTATTTCTGGCTGGCACGG-TGACAACAT--GCTTGAGGAGTCCACGAAGTATGAA
GTTCAGATTTGTATTTCTACTGAACCCAACT-TATG-CTTGCG----------------CAGCATGCCCTGG-TTCAAGG
GCTGGACCAAGGAGAC---CAAAGCTGGT--GTCGTCAAGGGCAAGACTCTTCTC---GAAGCTATCG------------
-ATGCTATTGAGCCCCCGGTCCGGCCTCATGAC-

Mycena_glabra_WBY449

ACTTGGTG-TTGA-GCTGGCC-CTT------TT--GGGCA-TGTGCTTGCATCAT--ATTA-TTT-A-TCT---ATCTCT
-TGTGCACCTTTTG-TAGTCTTTGAAGTG--
----TTCGCAGTC----------------------AATGCGG---------TTT--------------TGGGG---CTTG
GGC-----------TT---T-GCCCTCTTCCCTG--
---------------TTTG------CTTCA---------AAG-----------------------GCTATG-----TTTT
TA----------------------------TACA-CACTA----TCAAAGTTAC-AGAATGTC---TCTTATTGA-----
--C-TT---T-TGGGTCAGT--------AAATCTT-ATACAACTTTCAGCAACGGATCTCTTGGCTCTCCCATCGATGAA
GAACGCAGCGAAATGCGATAAGTAATGTGAATTGCAGAATTCAGTGAATCATCGAATCTTTGAACGCACCTTGCGCCCTT
TGGTATTCCGAAGGGCATGCCTGTTTGAGTGTC-ATTAAATTC-TCAA---------CCTCGCAA-----GC--------
----TTGCTTTGC-------CGAGGC---TTGGAT-GTGA-GG---G-TTTTTGCTGGC-TTCC----TTCAGTT-GGAT
GGTCTGCTCCCTTTAAATTCATTAGTGGGA-TCCT--TTGTGGA-----------TGG-TCACTTGG-TGTGATAA-TTA
TCTACGCCGC-CTGACTCTGAAACAAGACTTATGGG-AA-CCTGCTTATAACTG------TCTC-TTC---AAGAGACAA
-GCTTAT--C-------TTGACAATTTGACCT-GCCTTCACTCTCGGTGTGCGCCAGCTCATCGTTGCGGTGAACAAGAT
GGACACGACCAAGGTTCG---GTGCATTATAA-GCC---------------ACTAACGAATAA--------TTGAT--GA
CACCTATTCATAGTGGAGTGAGGACCGCTTCAACGAAATCATCAAGGAGACGTCCACCTTCATTAAGAAGGTCGGCTACA
ACCCCAAATCGATTTCTTTCGTCCCTATTTCTGGCTGGCACGG-TGACAACAT--GCTTGAGGAGTCCACGAAGTATGAA
GTTCAGATTTGTATTTCTACTGAACCCAACT-TATG-CTTGCG----------------CAGCATGCCCTGG-TTCAAGG
GCTGGACCAAGGAGAC---CAAAGCTGGT--GTCGTCAAGGGCAAGACTCTTCTC---GAAGCTATCG------------
-ATGCTATTGAGCCCCCGGTCCGGCCTCATGAC-

Mycena_lammiensis_TUR165927

ACTTGGTG-TTGA-GCTGGCC-CTA-----GTC--GGGCA-TGTGCTTGCATCAT--ATTA-TTT-A-CCT---ATCTCT
-TGTGCACCTTTTG-TAGTCTTTGAAGTG--
----TTCGCAGTCA---------------------AATGCGG---------TTT--------------TGGGG---ATTG
GGC-----------TTTGTT-GCGCTTTCCCCTG--
---------------TTTG------CTTCA---------AAG-----------------------GCTATG-----TTTT
TA----------------------------TACA-CACTA----TTAAAGTCAC-AGAATGTC---TCTTATTGA-----
--C-TT---T-TGAGTCAGT--------AAATC-T-ATACAACTTTCAGCAACGGATCTCTTGGCTCTCCCATCGATGAA
GAACGCAGCGAAATGCGATAAGTAATGTGAATTGCAGAATTCAGTGAATCATCGAATCTTTGAACGCACCTTGCGCCCTT
TGGTATTCCGAAGGGCATGCCTGTTTGAGTGTC-ATTMAATTC-TCAA---------CCTTGCAA-----GT--------
--T-TTGC-TTG--------CGAGGC---TTGGAT-GTGA-GG---G-TTTTTGCTGGC-TTCCA---TTCAGTT-GGAT
GGTCTGCTCCCTTTAAATTCATTAGTGGGA-TCCT--TTGTGGA-----------TGG-TCACTTGG-TGTGATAA-TTA
TCTACGCCGC-CTGACTCTGAAACAAGACTTGTGGG-AA-CCTGCTTATAACCG------TCTC-TTTT--G-GAGACA-
-GCCTAC--A-------TTGACAATTTGACCT-GCCTTCACCCTCGGTGTACGCCAGCTTATCGTTGCGGTCAACAAGAT
GGACACGACCAAGGTTCG---GTGCATTATAA-GCCC--------------CCTATCGAATAAT------TTTGAT--AA

CA-CTATTCACAGTGGAGTGAGGACCGTTTCAACGAAATCATCAAGGAGACGTCCACTTTCATTAAGAAGGTCGGCTTCA
ACCCCAAAAGTATTTCTTTCGTCCCTATTTCTGGCTGGCACGG-TGACAACAT--GCTTGAGGAGTCCACGAAGTATGAA
GTTCAGATTTGTATTTCT------CCCAACT-TATG-TTTGCG----------------CAGCATGCCCTGG-TTCAAGG
GCTGGACCAAGGAGAC---CAAAGCTGGT--GTCATCAAGGGCAAGACTCTTCTT---GAAGCTATCG------------
-ATGCTATTGAGCCCCCGATCCGGCCTCTGGAC-
Mycena_pelianthina_CBH015
ACTTGGTG-TTGA-GCTGGCC-CTA-----GTC--GGGCA-TGTGCTTGCATCAC--ATTA-TTT-A-CCT---ATCTCT
-TGTGCACCTTTTG-TAGTCTTTGAAGTG---
----TTCGCAGTCA---------------------AATGCGG---------TTT--------------TGGGG---ATTG
GGC-----------TTTGTT-GTGCTTTCCCCTG--
---------------TTTG------CTTCA---------AAG-----------------------GCTATG-----TTTT
TA----------------------------TACA-CACTA----TTAAAGTCAC-AGAATGTC---TCTTATTGA-----
--C-TT---T-CGAGTCAGT--------AAATC-T-ATACAACTTTCAGCAACGGATCTCTTGGCTCTCCCATCGATGAA
GAACGCAGCGAAATGCGATAAGTAATGTGAATTGCAGAATTCAGTGAATCATCGAATCTTTGAACGCACCTTGCGCCCTT
TGGTATTCCGAAGGGCATGCCTGTTTGAGTGTC-ATTAAATTC-TCAA---------CCTTGCAA-----GT--------
--TTTTGC-TTG--------TGAGGC---TTGGAT-GTGA-GG---G-TTTTTGCTGGC-TTCCA---TTCAGTT-GGAT
GGTCTGCTCCCTTTAAATTCATTAGTGGGA-TCCT--TTGTGGA-----------TGG-TCACTTGG-TGTGATAA-TTA
TCTACGCCGC-CTGAACCTGAAACAAGACTTGTGGG-AA-CCTGCTTATAACCG------TCTC-TTTC--G-GAGACA-
-GCCTAC--A-------TTGACAATTTGACCT-GCCTTCACCCTCGGTGTGCGCCAGCTTATCGTTGCGGTCAACAAGAT
GGACACGACCAAGGTTCG---GTGCATTATAA-GGCC--------------CCTATCGAATAAT------TTTGGT--AA
CACCTATTCACAGTGGAGTGAGGACCGTTTCAACGAAATCATCAAGGAGACGTCCACTTTCATTAAGAAGGTCGGCTACA
ACCCTAAATCTATCTGTTTCGTCCCTATTTCTGGCTGGCACGG-TGACAACAT--GCTTGAGGAGTCCACGAAGTATGAA
GTTCAGATTTGTATTTCT------CACAACT-TATG-TTTGCG----------------CAGCATGCCCTGG-TTCAAGG
GCTGGACCAAGGAGAC---CAAAGGTGGT--GTCGTTAAGGGCAAGACTCTTCTT---GAAGCTATCG------------
-ATGCTATCGAGGCCCCGGTCCGGCCTAAGGAC-
Mycena_pelianthina_CBH016
ACTTGGTG-TTGA-GCTGGCC-CTA-----GTC--GGGCA-TGTGCTTGCATCAC--ATTA-TTT-A-CCT---ATCTCT
-TGTGCACCTTTTG-TAGTCTTTGAAGTG---
----TTCGCAGTCA---------------------AATGCGG---------TTT--------------TGGGG---ATTG
GGC-----------TTTGTT-GCGCTTTCCCCTG--
---------------TTTG------CTTCA---------AAG-----------------------GCTATG-----TTTT
TA----------------------------TACA-CACTA----TTAAAGTCAC-AGAATGTC---TCTTATTGA-----
--C-TT---T-CGAGTCAGT--------AAATC-T-ATACAACTTTCAGCAACGGATCTCTTGGCTCTCCCATCGATGAA
GAACGCAGCGAAATGCGATAAGTAATGTGAATTGCAGAATTCAGTGAATCATCGAATCTTTGAACGCACCTTGCGCCCTT
TGGTATTCCGAAGGGCATGCCTGTTTGAGTGTC-ATTAAATTC-TCAA---------CCTTGCAA-----GT--------
--TTTTGC-TTG--------TGAGGC---TTGGAT-GTGA-GG---G-TTTTTGCTGGC-TTCCA---TTCAGTT-GGAT
GGTCTGCTCCCTTTAAATTCATTAGTGGGA-TCCT--TTGTGGA-----------TGG-TCACTTGG-TGTGATAA-TTA
TCTACGCCGC-CTGAACCTGAAACAAGACTTGTGGG-AA-CCTGCTTATAACCG------TCTC-TTTC--G-GAGACA-
-GCCTAC--A-------TTGACAATTTGACCT-GCCTTCACCCTCGGTGTGCGCCAGCTTATCGTTGCGGTCAACAAGAT
GGACACGACCAAGGTTCG---GTGCATTATAA-GCCC--------------CCTATCGAATAAT------TTTGGT--AA
CACCTATTCACAGTGGAGTGAGGACCGTTTCAACGAAATCATCAAGGAGACGTCCACTTTCATTAAGAAGGTCGGCTACA
ACCCTAAATCTATCTGTTTCGTCCCTATTTCTGGCTGGCACGG-TGACAACAT--GCTTGAGGAGTCCACGAAGTATGAA
GTTCAGATTTGTATTTCT------CACAACT-TATG-TTAGCG----------------CAGCATGCCCTGG-TTCAAGG
GCTGGACCAAGGAGAC---CAAAGGTGGT--GTCGTTAAGGGCAAGACTCTTCTT---GAAGCTATCG------------

-ATGCTATCGAGGCCCCGGTCCGGCCTAAGGAC-

Mycena_polycystidiata_FFAAS0417

ACTTGGTG-TTGA-GCTGGCC-TTA------TT--GGGCA-TGTGCTCGCATCAT--ATTA-TTT-A-CCT---ATCTCT
-TGTGCACCTTTTG-TAGTCTTTGAAGTG---
----TTCGCAGTC----------------------AATGCGG---------TTT--------------TGGGT---CTTG
GGC-----------TTTGCT-GCCCTTTTCCCTG--
---------------TTTG------CTTCA---------AAG-----------------------GCTATG-----TTTT
TA----------------------------TACA-CACTA--T-TTGAAGTTAC-AGAATGTC---TCTTATTGA-----
--C-CT---T-CGAGTCAGT--------AAATC-T-ATACAACTTTCAGCAACGGATCTCTTGGCTCTCCCATCGATGAA
GAACGCAGCGAAATGCGATAAGTAATGTGAATTGCAGAATTCAGTGAATCATCGAATCTTTGAACGCACCTTGCGCCCTT
TGGTATTCCGAAGGGCATGCCTGTTTGAGTGTC-ATTAAATTC-TCAA---------CCTTGCAA-----GA-TTTT---
----TTTC-TTG--------TGAGGC---TTGGAT-GTGA-GG---G-TTTTTGCTGGC-TTCC----TTTATCA-GGAT
GGTCTGCTCCCTTTAAATTCATTAGTGGGA-TCCT--TTGTGGA-----------TGG-TCACTTGG-TGTGATAA-TTA
TCTACGCCGC-CTGACTTTGAAACAAGACTTGTGGG-AA-CCTGCTCATAACCG------TCTC-TTCA--G--AGACA-
-ATCTAT--A-------TTGACAATTTGACCT-GCCTTCACTCTCGGTGTGCGCCAGCTCATCGTTGCGGTCAACAAGAT
GGACACGACCAAGGTTCG---GTGCATTATAA-GCC----------------CTAACGAATCGT------TTTGAT--GA
CACGTATTCACAGTGGAGTGAGGACCGTTTCAACGAAATCGTTAAGGAGACGTCCACTTTCATTAAGAAGGTCGGCTACA
ACCCCAAATCTATTTCTTTCGTCCCGATTTCTGGCTGGCACGG-TGACAACAT--GCTTGAGGAGTCCACCAAGTATGAA
ATTCAGATTTGTATTTCTAYCGAACCCAGCT-TATG-TTTGCG----------------CAGCATGCCCTGG-TTCAAGG
GCTGGACCAAGGAGAC---CAAAGGTGGT--GTCGTCAAGGGCAAGACTCTCCTC---GAAGCTATCG------------
-ATGCTATTGAGCCCCCGGTCCGGCCTGTGGAC-

Mycena_polycystidiata_FFAAS0418

TCTTGGTG-TTGA-GCTGGCC-TTA------TT--GGGCA-TGTGCTCGCATCAT--ATTA-TTT-A-CCT---ATCTCT
-TGTGCACCTTTTG-TAGTCTTTGAAGTG---
----TTCGCAGTC----------------------AATGCGG---------TTT--------------TGGGT---CTTG
GGC-----------TTTGCT-GCCCTTTTCCCTG--
---------------TTTG------CTTCA---------AAG-----------------------GCTATG-----TTTT
TA----------------------------TACA-CACTA--T-TTGAAGTTAC-AGAATGTC---TCTTATTGA-----
--C-CT---T-CGAGTCAGT--------AAATC-T-ATACAACTTTCAGCAACGGATCTCTTGGCTCTCCCATCGATGAA
GAACGCAGCGAAATGCGATAAGTAATGTGAATTGCAGAATTCAGTGAATCATCGAATCTTTGAACGCACCTTGCGCCCTT
TGGTATTCCGAAGGGCATGCCTGTTTGAGTGTC-ATTAAATTC-TCAA---------CCTTGCAA-----GA-TTTT---
----TTTC-TTG--------TGAGGC---TTGGAT-GTGA-GG---G-TTTTTGCTGGC-TTCC----TTTATCA-GGAT
GGTCTGCTCCCTTTAAATTCATTAGTGGGA-TCCT--TTGTGGA-----------TGG-TCACTTGG-TGTGATAA-TTA
TCTACGCCGC-CTGACTTTGAAACAAGACTTGTGGG-AA-CCTGCTCATAACCG------TCTC-TTCA--G--AGACA-
-ATCTAT--A-------TTGACAATTTGACCT-GCCTTCACTCTCGGTGTGCGCCAGCTCATCGTTGCGGTCAACAAGAT
GGACACGACCAAGGTTCG---GTGCATTATAA-GCC----------------CTAACGAATCGT------TTTGAT--GA
CACGTATTCACAGTGGAGTGAGGACCGTTTCAACGAAATCGTTAAGGAGACGTCCACTTTCATTAAGAAGGTCGGCTACA
ACCCCAAATCTATTTCTTTCGTCCCGATTTCTGGCTGGCACGG-TGACAACAT--GCTTGAGGAGTCCACCAAGTATGAA
ATTCAGATTTGTATTTCTAYCGAACCCAGCT-TATG-TTTGCG----------------CAGCATGCCCTGG-TTCAAGG
GCTGGACCAAGGAGAC---CAAAGGTGGT--GTCGTCAAGGGCAAGACTCTCCTC---GAAGCTATCG------------
-ATGCTATTGAGCCCCCGGTCCGGCCTGTGGAC-

Mycena_yuezhuoi_FFAAS0344

CATTGGTG-TTGA-GCTGGCC-CTA------TT--GGGCA-TGTGCTCGCATCAT--ATTA-TTT-A-TCT---ATCTCT
-TGTGCACCTTTTG-TAGTCTTTGTAGCA---

----CTCGCAGTC----------------------AATGCGG---------TTT--------------TGGGG---CTTG
GGC-----------TT---T-GCCCTTTCCCTG---
---------------TTTG------CTTCA---------AAG-----------------------GCTATG------TTT
TA----------------------------TACA-CACTA----TTGAAGTTAC-AGAATGTC---TCTTATTGA-----
--C-TC---T-CGAGTCAGT--------AAATC-T-ATACAACTTTCAGCAACGGATCTCTTGGCTCTCCCATCGATGAA
GAACGCAGCGAAATGCGATAAGTAATGTGAATTGCAGAATTCAGTGAATCATCGAATCTTTGAACGCACCTTGCGCCCTT
TGGTATTCCGAAGGGCATGCCTGTTTGAGTGTC-ATTAAATTC-TCAA---------CCTTGCAA-----GC--------
----TTGC-TTG--------CGAGGC---TTGGAT-GTGA-GG---G-TTTTTGCTGGC-TTCC----TTTAGTT-GGAT
GGTCTGCTCCCTTTAAATTCATTAGTGGGA-TCCT--TTGTGGA-----------TGG-TCACTTGG-TGTGATAA-TTA
TCTACGCCGT-CTGACTCTGAAACAAGACTTGTGGG-GA-CCTGCTTATAACTG------TCTC-TTCG--GAGACA---
-----AT--C-------TTGACAATTTGACCT-GCCTTCACTCTCGGTGTGCGCCAGCTTATCGTTGCGGTCAACAAGAT
GGACACGACCAAGGTTCG---GTGCATTACAA-CCC----------------CTAACGAATGAT------TTTGTT--GA
CACCTATTCACAGTGGAGTGAGGACCGTTTCAATGAAATCGTCAAGGAGACGTCGACTTTCATTAAGAAGGTTGGCTACA
ACCCCAAATCTATTTGTTTCGTCCCTATTTCTGGCTGGCACGG-TGACAACAT--GCTTGAGGAGTCCACGAAGTATGAA
GTTGAGA-TTGCATTTCTACCGAAACCAACT-TATG-TTTGCG----------------CAGCATGCCCTGG-TTCAAGG
GCTGGACCAAGGAGAC---CAAAGCTGGT--GTCGTCAAGGGCAAGACTCTTCTT---GAAGCTATCG------------
-ATGCTATTGAGCCCCCGGTCCGGCCTCATGAC-
Mycena_yuezhuoi_FFAAS0347
C-TTGGTG-TTGA-GCTGGCC-CTA------TT--GGGCA-TGTGCTCGCATCAT--ATTA-TTT-A-TCT---ATCTCT
-TGTGCACCTTTTG-TAGTCTTTGTAGCA---
----CTCGCAGTC----------------------AATGCGG---------TTT--------------TGGGG---CTTG
GGC-----------TT---T-GCCCTTTCCCTG---
---------------TTTG------CTTCA---------AAG-----------------------GCTATG------TTT
TA----------------------------TACA-CACTA----TTGAAGTTAC-AGAATGTC---TCTTATTGA-----
--C-TC---T-CGAGTCAGT--------AAATC-T-ATACAACTTTCAGCAACGGATCTCTTGGCTCTCCCATCGATGAA
GAACGCAGCGAAATGCGATAAGTAATGTGAATTGCAGAATTCAGTGAATCATCGAATCTTTGAACGCACCTTGCGCCCTT
TGGTATTCCGAAGGGCATGCCTGTTTGAGTGTC-ATTAAATTC-TCAA---------CCTTGCAA-----GC--------
----TTGC-TTG--------CGAGGC---TTGGAT-GTGA-GG---G-TTTTTGCTGGC-TTCC----TTTAGTT-GGAT
GGTCTGCTCCCTTTAAATTCATTAGTGGGA-TCCT--TTGTGGA-----------TGG-TCACTTGG-TGTGATAA-TTA
TCTACGCCGT-CTGACTCTGAAACAAGACTTGTGGG-GA-CCTGCTTATAACTG------TCTT-TTCG--GAGACA---
-----AT--C-------TTGACAATTTGACCT-GCCTTCACTCTCGGTGTGCGCCAGCTTATCGTTGCGGTCAACAAGAT
GGACACGACCAAGGTTCG---GTGCATTACAA-GCC----------------CTAACGAATGAT------TTT-TT--GA
CACCTATTCACAGTGGAGTGAGGACCGTTTCAATGAAATCGTCAAGGAGACGTCGACTTTCATTAAGAAGGTTGGCTACA
ACCCCAAATCTATTTGTTTCGTCCCTATTTCTGGCTGGCACGG-TGACAACAT--GCTTGAGGAGTCCACGAAGTATGAA
GTTGAGA-TTGCATTTCTACCGAAACCAACT-TATG-TTTGCG----------------CAGCATGCCCTGG-TTCAAGG
GCTGGACCAAGGAGAC---CAAAGCTGGT--GTCGTCAAGGGCAAGACTCTTCTT---GAAGCTATCG------------
-ATGCTATTGAGCCCCCGGTCCGGCCTCATGAC-
KB16M._pakistanica
ACTTGGTG-TTGA-GCTGGCC-CTA------TT--GGGCA-TGTGCTCGCATCAT--ATTG-TTT-A-TCT---ATCTCT
-TGTGCACCTTTTG-TAGTCTTTGAAGCA---
----CTCGCAGTC----------------------AATGCGG---------TTT--------------TGGGG---CTTG
GGC-----------TTTGCT-GCCCTTTTCCCTG--
---------------TTTG------CTTCA---------AAG-----------------------GCTATG------TTT
TA----------------------------TACA-CACTA----TTAAAGTTAC-AGAATGTC---TCTTATTGA-----

--C-CT---T-CGAGTCAGT--------AAATC-T-ATACAACTTTCAGCAACGGATCTCTTGGCTCTCCCATCGATGAA
GAACGCAGCGAAATGCGATAAGTAATGTGAATTGCAGAATTCAGTGAATCATCGAATCTTTGAACGCACCTTGCGCCCTT
TGGTATTCCGAAGGGCATGCCTGTTTGAGTGTC-ATTAAATTC-TCAA---------CCTTGCAA-----GC--------
----TTGC-TTG--------CGAGGC---TTGGAT-GTGA-GG---G-TCTTTGCTGGC-TTCC----TTTAGTTGGGAT
GGTCTGCTCCCTTTAAATTCATTAGTGGGA-TCCT--TTGTGGA-----------TGG-TCACTTGG-TGTGATAA-TTA
TCTACGCCGC-CTGACTCTGAAACAAGACTTGTGGG-AA-CCCGCTTATAACCG------CCTC-TTCG--GAGACA---
-----AT--C-------TTGACAATTTGACCT--

FLF1785

ACTTGGTG-TTGA-GCTGGCC-CTA------TT--GGGCA-TGTGCTCGCATCAT--ATTA-TTT-A-TCT---ATCTCT
-TGTGCACCTTTTG-TAGTCTTTGAAGCG---
----TTCGCAGTC----------------------AATGCGG---------TTT--------------TGGGG---CTTG
GGC-----------TTTGCT-GCCCTTTTCCCTG--
---------------TCTG------CTTCA---------AAG-----------------------GCTATG-----TTTT
TA----------------------------TACA-CACTA----TTGAAGTTAC-AGAATGTC---TCTTATTGA-----
--C-TT---T-CGAGTCAGT--------AAATC-T-ATACAACTTTCAGCAACGGATCTCTTGGCTCTCCCATCGATGAA
GAACGCAGCGAAATGCGATAAGTAATGTGAATTGCAGAATTCAGTGAATCATCGAATCTTTGAACGCACCTTGCGCCCTT
TGGTATTCCGAAGGGCATGCCTGTTTGAGTGTC-ATTAAATTC-TCAA---------CCTTGCAA-----GC--------
--TTTTGC-TTG--------CGAGGC---TTGGAT-GTGA-GG---G--CTTTGCTGGC-TTCC----TTCAGTG-GGAT
GGTCTGCTCCCTTTAAATTCATTAGTGGGA-TCCT--TTGTGGA-----------TGG-TCACTTGG-TGTGATAA-TTA
TCTACGCCGC-CTGACTATGAAACAAGACTTGTGGG-AA-CCTGCTTATAACCG------TCTC-TTCA--GAGACA---
-GCTTAT--C-------TTGACAATTTGACCT-GCCTTCACTCTCGGTGTGCGCCAGCTTATCGTTGCGGTCAACAAGAT
GGACACGACCAAGGTTCG---GTGCATTATAA-TCC----------------CTAACGAATAAT------ATTGAT--GA
CACCTATTCACAGTGGAGTGAGGACCGTTTCAACGAAATCATTAAGGAGACGTCCACTTTCATTAAGAAGGTCGGCTACA
ACCCCAAACATATATCTTTCGTCCCTATTTCTGGCTGGCACGG-TGACAACAT--GCTTGAGGAGTCCACGAAGTATGAA
GTTCAGATTTGTATCTCTACCGAACCCAACT-TACG-TTTGCG----------------CAGCATGCCCTGG-TTCAAGG
GCTGGACCAAGGAGAC---CAAAGCTGGT--GTCGTCAAGGGCAAGACTCTTCTT---GAAGCTATCG------------
-ATGCTATCGAGCCCCCGGTCCGGCCTAGTGAC-

MSX1785

ACTTGGTG-TTGA-GCTGGCC-CTA------TT--GGGCA-TGTGCTCGCATCAT--ATTA-TTT-A-TCT---ATCTCT
-TGTGCACCTTTTG-TAGTCTTTGAAGCG---
----TTCGCAGTC----------------------AATGCGG---------TTT--------------TGGGG---CTTG
GGC-----------TTTGCT-GCCCTTTTCCCTG--
---------------TCTG------CTTCA---------AAG-----------------------GCTATG-----TTTT
TA----------------------------TACA-CACTA----TTGAAGTTAC-AGAATGTC---TCTTATTGA-----
--C-TT---T-CGAGTCAGT--------AAATC-T-ATACAACTTTCAGCAACGGATCTCTTGGCTCTCCCATCGATGAA
GAACGCAGCGAAATGCGATAAGTAATGTGAATTGCAGAATTCAGTGAATCATCGAATCTTTGAACGCACCTTGCGCCCTT
TGGTATTCCGAAGGGCATGCCTGTTTGAGTGTC-ATTAAATTC-TCAA---------CCTTGCAA-----GC--------
--TTTTGC-TTG--------CGAGGC---TTGGAT-GTGA-GG---G--CTTTGCTGGC-TTCC----TTCAGTG-GGAT

GGTCTGCTCCCTTTAAATTCATTAGTGGGA-TCCT--TTGTGGA-----------TGG-TCACTTGG-TGTGATAA-TTA
TCTACGCCGC-CTGACTATGAAACAAGACTTGTGGG-AA-CCTGCTTATAACCG------TCTC-TTCA--GAGACA---
-GCTTAT--C-------TTGACAATTTGACCT-GCCTTCACTCTCGGTGTGCGCCAGCTTATCGTTGCGGTCAACAAGAT
GGACACGACCAAGGTTCG---GTGCATTATAA-TCC----------------CTAACGAATAAT------ATTGAT--GA
CACCTATTCACAGTGGAGTGAGGACCGTTTCAACGAAATCATTAAGGAGACGTCCACTTTCATTAAGAAGGTCGGCTACA
ACCCCAAACATATATCTTTCGTCCCTATTTCTGGCTGGCACGG-TGACAACAT--GCTTGAGGAGTCCACGAAGTATGAA
GTTCAGATTTGTATCTCTACCGAACCCAACT-TACG-TTTGCG----------------CAGCATGCCCTGG-TTCAAGG
GCTGGACCAAGGAGAC---CAAAGCTGGT--GTCGTCAAGGGCAAGACTCTTCTT---GAAGCTATCG------------
-ATGCTATCGAGCCCCCGGTCCGGCCTAGTGAC-

Mycena_luciferina_ACP2114

ACTTGGTG-TTGA-GCTGG-C-CTA------TT--GGGCA-TGTGCTCGCATCAT--ATTA-TTT-A-TCT---ATCTCT
-TGTGCACCTTTTG-TAGTCTTTGAAGTG---
----TTCGCAGTC----------------------AATGCGG---------TTT--------------TGGGG---TTTG
GGC-----------TTTGCT-GTCCCTTGCCCTG---
---------------TTTG------CTTCA---------AAG-----------------------GCTATGT----TTTA
CA----------------------------TACA-CACTA----TTGAAGTTAC-AGAATGTC---TCTTATTGA-----
--C-TT---T-CGAGTCAGT--------AAATC-T-ATACAACTTTCAGCAACGGATCTCTTGGCTCTCCCATCGATGAA
GAACGCAGCGAAATGCGATAAGTAATGTGAATTGCAGAATTCAGTGAATCATCGAATCTTTGAACGCACCTTGCGCCCTT
TGGTATTCCGAAGGGCATGCCTGTTTGAGTGTC-ATTAAATTC-TCAA---------CCTTGCAA-----GC-TTTTTTT
AAAATTGCTTTG--------CGGGGC---TTGGAT-GTGA-GG---GCTTTTTGCTGGC-TTCC----TTCAGTT-GGAT
GGTCTGCTCCCTTTAAATTCATTAGTGGGA-TCCT--TTGTGGA-----------TGG-TCACTTGG-TGTGATAA-TTA
TCTACGCCGC-CTGACTCTGAAACAAGACTTGTGGG-GA-CCTGCTTATAACCG------TCTC-TTCA--AAGAGACA-
-GCTTAT--C-------TTGACAATTTGACCT--

Mycena_cf_pura_I_CBH039

ACTTGGTG-TTGA-GCTGGCC-CTA------TT--GGGCA-TGTGCTCGCACCAT--ATTA-TTT-A-TCT---ATCTCT
-TGTGCACCTTTTG-TAGTCTTTGAAGTG---
----TTCGCAGTC----------------------AATGCGG----------TT--------------TGGGG---CTTG
GGC-----------TTTTGA-AGTCCTTTCCCTG---
---------------TTTG------CTTCA---------AAG-----------------------GCTATG------TTT
TA----------------------------TACA-CACTA--T-TTGAAGTTAC-AGAATGTC---TCTTATTGA-----
--C-CT---T-AGAGTCAGT--------AAATC-A-ATACAACTTTCAGCAACGGATCTCTTGGCTCTCCCATCGATGAA
GAACGCAGCGAAATGCGATAAGTAATGTGAATTGCAGAATTCAGTGAATCATCGAATCTTTGAACGCACCTTGCGCCCTT
TGGTATTCCGAAGGGCATGCCTGTTTGAGTGTC-ATTAAATTC-TCAA---------CCTTGCAA-----GC---TT---
--TTTTGC-TAG--------TGAGGC---TTGGAT-GTGA-GG---G-CTTTTGCTGGC-TTCCA---TTCAGTT-GGAT
GGTCTGCTCCCTTTAAATTCATTAGTGGAT-TCCT--TTGTGGA-----------TGG-TCACTTGG-TGTGATAA-TTA
TCTACGCCGC-CTGACTCTGATACAAGACTTGTGGG-GA-CCTGCTTATAACCG------TCTC-TTTC--AAGAGACA-
-ATCTCT--A-------TTGACAATTTGACCT-GCCTTCACTCTCGGTGTGCGCCAGCTTATCGTTGCGGTCAACAAGAT
GGACACGACCAAGGTTCG---GTGCATTATCA-CCC----------------CTAACGAATAAT------TTCGAT--GA

CATCGATTCACAGTGGAGCGAGGACCGTTTCAACGAAATCGTCAAGGAGACGTCCACTTTCATTAAGAAGGTCGGCTACA
ACCCCAAAAATATTTGTTTCGTCCCTATCTCTGGCTGGCACGG-TGACAACAT--GCTTGAGGAGTCCACGAAGTATGAA
GTTCAGA-TTGTATTTCTACCGAACCTAACT-TGAG-TTTGCG----------------CAGCATGCCCTGG-TTCAAGG
GCTGGACCAAGGAGAC---CAAAAGTGGT--GTCGTCAAGGGCAAGACTCTTCTT---GAAGCTATCG------------
-ATGCTATCGAGCCCCCGGTACGGCCTATGGAC-
Mycena_cf_pura_IV_CBH410
ACTTGGTG-TTGA-GCTGGCC-CTA------TT--GGGCA-TGTGCTCGCACCAT--ATTA-TTT-A-TCT---ATCTCT
-TGTGCACCTTTTG-TAGTCTTTGAAGTG---
----TTCGCAGTC----------------------AATGCGG----------TT--------------TGGGG---TTTG
GGC-----------TTTTGA-AGTCCTTTCCCTG--
---------------TTTG------CTTCA---------AAG-----------------------GCTATG------TTT
TA----------------------------TACA-CACTA--T-TTGAAGTTAC-AGAATGTC---TCCTATTGA-----
--C-CT---T-AGAGTCAGT--------AAATC-A-ATACAACTTTCAGCAACGGATCTCTTGGCTCTCCCATCGATGAA
GAACGCAGCGAAATGCGATAAGTAATGTGAATTGCAGAATTCAGTGAATCATCGAATCTTTGAACGCACCTTGCGCCCTT
TGGTATTCCGAAGGGCATGCCTGTTTGAGTGTC-ATTAAATTC-TCAA---------CCTTGCAA-----GC---TT---
--TTTTGC-TAG--------TGAGGC---TTGGAT-GTGA-GG---G-CTTTTGCTGGC-TTCCA---TTCAGTT-GGAT
GGTCTGCTCCCTTTAAATTCATTAGTGGAT-TCCT--TTGTGGA-----------TGG-TCACTTGG-TGTGATAA-TTA
TCTACGCCGC-CTGACTCTGATACAAGACTTGTGGG-GA-CCTGCTCATAACCG------TCTC-TTC---AAGAGACA-
-ATCTTT--A-------TTGACAATTTGACCT-GCCTTCACTCTCGGTGTGCGCCAGCTTATCGTTGCGGTCAACAAGAT
GGACACGACCAAGGTTCG---GTGCATTATCA-GCC----------------CTAACGAATAAT------TTTGAT--GA
CACCGATTCACAGTGGAGTGAGGACCGTTTCAACGAAATCGTCAAGGAGACGTCCACTTTCATTAAGAAGGTCGGCTACA
ACCCCAAATCTATTTCTTTCGTCCCTATCTCTGGCTGGCACGG-TGACAACAT--GCTTGAGGAGTCCACGAAGTATGAA
GTTCAGATTTGTATTTCTACCGAATCTAACT-TATG-TTTG------------------CAGCATGCCCTGG-TTCAAGG
GCTGGACCAAGGAGAC---CAAAGCTGGT--GTCGTCAAGGGCAAGACTCTTCTT---GAAGCTATCG------------
-ATGCCATCGAGCCCCCGCTCCGGCCTGTGGAC-
Mycena_cf_pura_IV_JV06979
ACTTGGTG-TTGA-GCTGGCC-CTA------TT--GGGCA-TGTGCTCGCACCAT--ATTA-TTT-A-TCT---ATCTCT
-TGTGCACCTTTTG-TAGTCTTTGAAGTG---
----TTCGCAGTC----------------------AATGCGG----------TT--------------TGGGG---TTTG
GGC-----------TTTTGA-AGTCCTTTCCCTG--
---------------TTTG------CTTCA---------AAG-----------------------GCTATG------TTT
TA----------------------------TACA-CACTA--T-TTGAAGTTAC-AGAATGTC---TCCTATTGA-----
--C-CT---T-AGAGTCAGT--------AAATC-A-ATACAACTTTCAGCAACGGATCTCTTGGCTCTCCCATCGATGAA
GAACGCAGCGAAATGCGATAAGTAATGTGAATTGCAGAATTCAGTGAATCATCGAATCTTTGAACGCACCTTGCGCCCTT
TGGTATTCCGAAGGGCATGCCTGTTTGAGTGTC-ATTAAATTC-TCAA---------CCTTGCAA-----GC---TT---
--TTTTGC-TAG--------TGAGGC---TTGGAT-GTGA-GG---G-CTTTTGCTGGC-TTCCA---TTCAGTT-GGAT
GGTCTGCTCCCTTTAAATTCATTAGTGGAT-TCCT--TTGTGGA-----------TGG-TCACTTGG-TGTGATAA-TTA
TCTACGCCGC-CTGACTCTGATACAAGACTTGTGGG-GA-CCTGCTCATAACCG------TCTC-TTC---AAGAGACA-
-ATCTTT--A-------TTGACAATTTGACCT-GCCTTCACTCTCGGTGTGCGCCAGCTTATCGTTGCGGTCAACAAGAT
GGACACGACCAAGGTTCG---GTGCATTATCA-GCC----------------CTAACGAATAAT------TTTGAT--GA
CACCGATTCACAGTGGAGTGAGGACCGTTTCAACGAAATCGTCAAGGAGACGTCCACTTTCATTAAGAAGGTCGGCTACA
ACCCCAAATCTATTTCTTTCGTCCCTATCTCTGGCTGGCACGG-TGACAACAT--GCTTGAGGAGTCCACGAAGTATGAA
GTTCAGATTTGTATTTCTACCGAATCTAACT-TATG-TTTG------------------CAGCATGCCCTGG-TTCAAGG
GCTGGACCAAGGAGAC---CAAAGCTGGT--GTCGTCAAGGGCAAGACTCTTCTT---GAAGCTATCG------------

-ATGCCATCGAGCCCCCGCTCCGGCCTGTGGAC-
Mycena_cf_pura_II_CBH105
ACTTGGTG-TTGA-GCTGGCC-CTA------TT--GGGCA-TGTGCTCGCATCAT--ATTA-TTT-A-TCT---ATCTCT
-TGTGCACCTTTTG-TAGTCTTTGAAGTG--
----TTCGCAGTC----------------------AATGCGG----------TT--------------TGGGG---TTTG
GGC-----------TTTTGC-AGTCCTTTCCCTG--
---------------TTTG------CTTCA---------AAG-----------------------GCTATG------TTT
TA----------------------------TACA-CACTA--T-TTGAAGTTAC-AGAATGTC---TCTTATTGA-----
--C-CT---T-TGAGTCAGT--------AAATC-A-ATACAACTTTCAGCAACGGATCTCTTGGCTCTCCCATCGATGAA
GAACGCAGCGAAATGCGATAAGTAATGTGAATTGCAGAATTCAGTGAATCATCGAATCTTTGAACGCACCTTGCGCCCTT
TGGTATTCCGAAGGGCATGCCTGTTTGAGTGTC-ATTAAATTC-TCAA---------CCTTGCAA-----GC---TT---
--TTTTGC-TAG--------TGAGGC---TTGGAT-GTGA-GG---G-CTTTTGCTGGC-TTCC----TTCATT--GGAT
GGTCTGCTCCCTTTAAATTCATTAGTGGAT-TCCT--TTGTGGA-----------TGG-TCACTTGG-TGTGATAA-TTA
TCTACGCCGC-CTGACTCTGACGCAAGACTTGTGGG-GA-CCTGCTTATAACCG------TCTC-TTTC--AAGAGACA-
-ATCTAC--A-------TTGACTATTTGACCT-GCCTTCACTCTCGGTGTGCGCCAGCTYATCGTTGCGGTCAACAAGAT
GGACACGACCAAGGTTCG---GTGCATTGTCA-GCC---------------STAACGAATAAT------TTTGAT--GA
CACCGATTCACAGTGGAGTGAGGACCGTTTCAACGAAATCGTCAAGGAGACGTCCACCTTYATTAAGAAGGTCGGCTACA
ACCCCAAAACTATTTGTTTCGTCCCTATCTCTGGCTGGCACGG-TGACAACAT--GCTTGAGGAGTCSGTCAAGTATGAA
GTTCAAATTTGTATTTCTACCGAACCTAACT-TATG-TTTGCG----------------CAGCATGCCCTGG-TTCAAGG
GCTGGACCAAGGAGAC---CAAAGGTGGT--GTCGTCAAGGGCAAGACTCTTCTT---GAAGCTATCG------------
-ATGCTATCGAGCCCCCGGTCCGGCCTAGCGAC-
Mycena_cf_pura_II_CBH366
ACTTGGTG-TTGA-GCTGGCC-CTA------TT--GGGCA-TGTGCTCGCATCAT--ATTA-TTT-A-TCT---ATCTCT
-TGTGCACCYTTTG-TAGTCTTTGAAGTG--
----TTCGCAGTC----------------------AATGCGG----------TT--------------TGGGG---TTTG
GGC-----------TTTTGC-AGTCCTTTCCCTG--
---------------TTTG------CTTCA---------AAG-----------------------GCTATG------TTT
TA----------------------------TACA-CACTA--T-TTGAAGTTAC-AGAATGTC---TCTTATTGA-----
--C-CT---T-TGAGTCAGT--------AAATC-A-ATACAACTTTCAGCAACGGATCTCTTGGCTCTCCCATCGATGAA
GAACGCAGCGAAATGCGATAAGTAATGTGAATTGCAGAATTCAGTGAATCATCGAATCTTTGAACGCACCTTGCGCCCTT
TGGTATTCCGAAGGGCATGCCTGTTTGAGTGTC-ATTAAATTC-TCAA---------CCTTGCAA-----GC---TT---
--TTTTGC-TAG--------TGAGGC---TTGGAT-GTGA-GG---G-CTTTTGCTGGC-TTCC----TTCATT--GGAT
GGTCTGCTCCCTTTAAATTCATTAGTGGAT-TCCT--TTGTGGA-----------TGG-TCACTTGG-TGTGATAA-TTA
TCTACGCCGC-CTGACTCTGACACAAGACTTGTGGG-GA-CCTGCTTATAACCG------TCTC-TTTC--AAGAGACA-
-ATCTAC--A-------TTGACTATTTGACCT-GCCTTCACTCTCGGTGTGCGCCAGCTCATCGTTGCGGTCAACAAGAT
GGACACGACCAAGGTTCG---GTGCATTGTCA-GCC---------------GTAACGAATAAT------TTTGAT--GA
CACCGATTCACAGTGGAGTGAGGACCGTTTCAACGAAATCGTCAAGGAGACGTCCACCTTCATTAAGAAGGTCGGCTACA
ACCCCAAAACTATTTGTTTCGTCCCTATCTCTGGCTGGCACGG-TGACAACAT--GCTTGAGGAGTCGGTCAAGTATGAA
GTTCAAATTTGTATTTCTACCGAACCTAACC-TATG-TTTGCG----------------CAGCATGCCCTGG-TTCAAGG
GCTGGACCAAGGAGAC---CAAAGGTGGT--GTCGTCAAGGGCAAGACTCTTCTT---GAAGCTATCG------------
-ATGCTATCGAGCCCCCGGTCCGGCCTAGCGAC-
Mycena_cf_pura_XI_CBH187
ACTTGGTG-TTGA-GCTGGCC-CTA------TT--GGGCA-TGTGCTCGCATCAT--ATTA-TTT-A-TCT---ATCTCT
-TGTGCACCTTTTG-TAGTCTTTGAAGCG--

----TTCGCAGTC----------------------AATGCGG----------TT--------------TGGGG---TTTG
GGC-----------TTTTGC-AGTCCTTTCCCTG--
---------------TTTG------CTTCA---------AAG-----------------------GCTATG------TTT
TA----------------------------TACA-CACTA--T-TTGAAGTTAC-AGAATGTC---TCTTATTGA-----
--C-CT---T--GAGTCAGT--------AAATC-A-ATACAACTTTCAGCAACGGATCTCTTGGCTCTCCCATCGATGAA
GAACGCAGCGAAATGCGATAAGTAATGTGAATTGCAGAATTCAGTGAATCATCGAATCTTTGAACGCACCTTGCGCCCTT
TGGTATTCCGAAGGGCATGCCTGTTTGAGTGTC-ATTAAATTC-TCAA---------CCTTGCAA-----GC---TT---
--TTTTGC-TAG--------TGAGGC---TTGGAT-GTGA-GG---G-C--TTGCTGGC-TTCC----TTCAGTT-GGAT
GGTCTGCTCCCTTTAAATTCATTAGTGGAT-TCCT--TTGTGGA-----------TGG-TCACTTGG-TGTGATAA-TTA
TCTACGCCGC-CTGACTCTGATACAAGACTTGTGGG-GA-CCTGCTCATAACCG------TCTC-TTC-----GAGACA-
-ATCTAT--A-------TTGACAATTTGACCT-GCCTTCACTCTCGGTGTGCGCCAGCTTATCGTTGCGGTCAACAAGAT
GGACACGACCAAGGTTCG---GTGCATTATCA-GCC----------------CTGACGAATAAT------GTTGAT--GA
CATCTATTCGCAGTGGAGTGAGGACCGTTTCAACGAAATCGTCAAGGAGACGTCCACCTTCATTAAGAAGGTCGGCTACA
ACCCCAAAACTATTTCTTTCGTCCCTATCTCTGGCTGGCACGG-TGACAATAT--GCTTGAGGAGTCCACGAAGTATGTA
GTTCAGATTTGTATTTCTACCGACCCTAACT-TGTG-TTTGTG----------------CAGCATGCCCTGG-TTCAAGG
GCTGGACCAAGGAGAC---CAAAGGTGGT--GTCGTCAAGGGCAAGACTCTTCTT---GAAGCTATCG------------
-ATGCTATCGAGGCCCCGACCCGGCCTACGGAC-
Mycena_cf_pura_XI_CBH386
ACTTGGTG-TTGA-GCTGGCC-CTA------TT--GGGCA-TGTGCTCGCATCAT--ATTA-TTT-A-TCT---ATCTCT
-TGTGCACCTTTTG-TAGTCTTTGAAGCG--
----TTCGCAGTC----------------------AATGCGG----------TT--------------TGGGG---CTTG
GGC-----------TTTTGC-AGTCCTTTCCCTG--
---------------TTTG------CTTCA---------AAG-----------------------GCTATG------TTT
TA----------------------------TACA-CACTA--T-TTGAAGTTAC-AGAATGTC---TCTTATTGA-----
--C-CT---T--GAGTCAGT--------AAATC-A-ATACAACTTTCAGCAACGGATCTCTTGGCTCTCCCATCGATGAA
GAACGCAGCGAAATGCGATAAGTAATGTGAATTGCAGAATTCAGTGAATCATCGAATCTTTGAACGCACCTTGCGCCCTT
TGGTATTCCGAAGGGCATGCCTGTTTGAGTGTC-ATTAAATTC-TCAA---------CCTTGCAA-----GC---TT---
--TTTTGC-TAG--------TGAGGC---TTGGAT-GTGA-GG---G-C--TTGCTGGC-TTCC----TTCAGTT-GGAT
GGTCTGCTCCCTTTAAATTCATTAGTGGAT-TCCT--TTGTGGA-----------TGG-TCACTTGG-TGTGATAA-TTA
TCTACGCCGC-CTGACTCTGATACAAGACTTGTGGG-GA-CCTGCTCATAACCG------TCTC-TTC-----GAGACA-
-ATCTAT--A-------TTGACAATTTGACCT-GCCTTCACTCTCGGTGTGCGCCAGCTTATCGTTGCGGTCAACAAGAT
GGACACGACCAAGGTTCG---GTGCATTATCA-GCC----------------CTGACGAATAAT------GTTGAT--GA
CATCTATTCGCAGTGGAGTGAGGACCGTTTCAACGAAATCGTCAAGGAGACGTCCACCTTCATTAAGAAGGTCGGCTACA
ACCCCAAAACTATTTCTTTCGTCCCTATCTCTGGCTGGCACGG-TGACAATAT--GCTTGAGGAGTCCACGAAGTATGTA
GTTCAGATTTGTATTTCTACCGACCCTAACT-TGTG-TTTGTG----------------CAGCATGCCCTGG-TTCAAGG
GCTGGACCAAGGAGAC---CAAAGGTGGT--GTCGTCAAGGGCAAGACTCTTCTT---GAAGCTATCG------------
-ATGCTATCGAGGCCCCGACCCGGCCTACGGAC-
Mycena_cf_pura_III_CBH019
ACTTGGTG-TTGA-GCTGGCC-CTA------GC--GGGCA-TGTGCTCGCATCAT--ATTA-TTT-A-TCT---ATCTCT
-TGTGCACCTTTTG-TAGTCTTTGAAGTT--
----TTCGCAGTC----------------------AATGCGG----------TT--------------TGGGG---CTTG
GGC-----------TTTTGC-AGTCCTTTCCCTG--
---------------TTTG------CTTCA---------AAG-----------------------GCTATG------TTT
TA----------------------------TACA-CACTA----TTGAAGTTAC-AGAATGTC---TCTTATTGA-----

--C-CT---T-TGAGTCAGT--------AAATC-A-ATACAACTTTCAGCAACGGATCTCTTGGCTCTCCCATCGATGAA
GAACGCAGCGAAATGCGATAAGTAATGTGAATTGCAGAATTCAGTGAATCATCGAATCTTTGAACGCACCTTGCGCCCTT
TGGTATTCCGAAGGGCATGCCTGTTTGAGTGTC-ATTAAATTC-TCAA---------CCTTGCAA-----GC---TT---
--TTTTGC-TAG--------TGAGGC---TTGGAT-GTGA-GG---G-TTTTTGCTGGC-TTCC----TTCAGTT-GGAT
GGTCTGCTCCCTTTAAATTCATTAGTGGAT-TCCT--TTGTGGA-----------TGG-TCACTTGG-TGTGATAA-TTA
TCTACGCCGC-CTGACTCTGATACAAGACTTGTGGG-GA-CCTGCTTATAACCG------TCTC-TTC---AAGAGACA-
-ATCTATACA-------TTGACAATTTGACCT-GCCTTCACTCTCGGTGTGCGCCAGCTTATCGTTGCGGTCAACAAGAT
GGACACGACCAAGGTTCG---GTACATTATCA-GCT----------------CCAACGAACAAT------TTTGAT--GA
CACCTATTCACAGTGGAGCGAGGACCGTTTCAACGAAATCGTCAAGGAGACGTCGACTTTTATTAAGAAGGTCGGCTACA
ACCCCAAATGTATTTCTTTCGTCCCTATCTCTGGCTGGCACGG-TGACAACAT--GCTCGAGGAGTCCACGAAGTATGAA
GTCCATATTTGTATTTYT-CCGAACCTAACT-TATG-TTTGCG----------------CAGCATGCCCTGG-TTCAAGG
GCTGGACCAAGGAGAC---CAAAGGTGGT--GTCGTCAAGGGCAAGACTCTTCTT---GAAGCTATCG------------
-ATGCTATCGAGCCCCCGGTCCGGCCTATGGAC-
Mycena_cf_pura_III_CBH022
ACTTGGTG-TTGA-GCTGGCC-CTA------GC--GGGCA-TGTGCTCGCATCAT--ATTA-TTT-A-TCT---ATCTCT
-TGTGCACCTTTTG-TAGTCTTTGAAGTT--
----TTCGCAGTC----------------------AATGCGG----------TT--------------TGGGG---CTTG
GGC-----------TTTTGC-AGTCCTTTCCCTG---
---------------TTTG------CTTCA---------AAG----------------------GCTATG------TTT
TA----------------------------TACA-CACTA----TTGAAGTTAC-AGAATGTC---TCTTATTGA-----
--C-CT---T-TGAGTCAGT--------AAATC-A-ATACAACTTTCAGCAACGGATCTCTTGGCTCTCCCATCGATGAA
GAACGCAGCGAAATGCGATAAGTAATGTGAATTGCAGAATTCAGTGAATCATCGAATCTTTGAACGCACCTTGCGCCCTT
TGGTATTCCGAAGGGCATGCCTGTTTGAGTGTC-ATTAAATTC-TCAA---------CCTTGCAA-----GC---TT---
--TTTTGC-TAG--------TGAGGC---TTGGAT-GTGA-GG---G-TTTTTGCTGGC-TTCC----TTCAGTT-GGAT
GGTCTGCTCCCTTTAAATTCATTAGTGGAT-TCCT--TTGTGGA-----------TGG-TCACTTGG-TGTGATAA-TTA
TCTACGCCGC-CTGACTCTGATACAAGACTTGTGGG-GA-CCTGCTTATAACCG------TCTC-TTC---AAGAGACA-
-ATCTATACA-------TTGACAATTTGACCT-GCCTTCACTCTCGGTGTGCGCCAGCTTATCGTTGCGGTCAACAAGAT
GGACACGACCAAGGTTCG---GTACATTATCA-GCT----------------CCAACGAACAAT------TTTGAT--GA
CACCTATTCACAGTGGAGCGAGGACCGTTTCAACGAAATCGTCAAGGAGACGTCGACTTTTATTAAGAAGGTCGGCTACA
ACCCCAAATGTATTTCTTTCGTCCCTATCTCTGGCTGGCACGG-TGACAACAT--GCTCGAGGAGTCCACGAAGTATGAA
GTCCATATTTGTATTTYT-CCGAACCTAACT-TATG-TTTGCG----------------CAGCATGCCCTGG-TTCAAGG
GCTGGACCAAGGAGAC---CAAAGGTGGT--GTCGTCAAGGGCAAGACTCTTCTT---GAAGCTATCG------------
-ATGCTATCGAGCCCCCGGTCCGGCCTATGGAC-
Mycena_cf_pura_V_CBH226
ACTTGGTG-TTGA-GCTGGCC-CTA------TT--GGGCA-TGTGCTCGCATCAT--ATTA-TTT-A-TCT---ATCTCT
-TGTGCACCTTTTG-TAGTCTTTGAAGTGT---
---TTTCGCAGTCA---------------------AATGCGG----------TT--------------TGGGG---CTTG
GGC-----------TTTTGC-GGTCCTTTCCCTG---
---------------TTTG------CTTCA---------AAG----------------------GCTATG------TTT
TA----------------------------TACA-CACTA----TTGAAGTTAC-AGAATGTC---TCTTATTGA-----
--C-CT---T-TGAGTCAGT--------AAATC-A-ATACAACTTTCAGCAACGGATCTCTTGGCTCTCCCATCGATGAA
GAACGCAGCGAAATGCGATAAGTAATGTGAATTGCAGAATTCAGTGAATCATCGAATCTTTGAACGCACCTTGCGCCCTT
TGGTATTCCGAAGGGCATGCCTGTTTGAGTGTC-ATTAAATTC-TCAA---------CCTTGCAA-----GC---TT---
--TTTTGC-TAG--------CGAGGC---TTGGAT-GTGA-GG---G-CTTTTGCTGGC-TTCC----TTCATTT-GGAT

GGTCTGCTCCCTTTAAATTCATTAGTGGAT-TCCT--TTGTGGA-----------TGG-TCACTTGG-TGTGATAA-TTA
TCTACGCCGC-CTGACTCTGAAACAAGACTTGTGGG-GA-CCTGCTCATAACCG------TCTC-TTC---AAGAGACA-
-ATCTTT--A-------TTGACAATTTGACCT-GCCTTCACTCTCGGTGTGCGCCAGCTTATCGTTGCGGTCAACAAGAT
GGACACGACCAAGGTTCG---GTGCATTATAA-GCC---------------CTAACGAATGAT------TTTGAT--GA
CACCTATTCACAGTGGAGCGAGGACCGTTTCAACGAAATCGTCAAGGAGACATCCACCTTCATTAAGAAGGTCGGGTACA
ACCCCAAATCTATTTCTTTCGTCCCTATCTCTGGCTGGCACGG-TGACAACAT--GCTTGAGGAGTCCACGAAGTATGAA
GTTCAGATTTGTATATCTACCGAACCTAACT-TATG-TTTGCG----------------CAGCATGCCCTGG-TTCAAGG
GCTGGACCAAGGAAAC---CAAAGGTGGT--GTCGTCAAGGCCAAAGAGCTTCTT---GAAGCTATCG------------
-ATGCTATCGAGCCCCCGGTCCGGCCTGTGGAC-

Mycena_cf_pura_V_TL5614

ACTTGGTG-TTGA-GCTGGCC-CTA------TT--GGGCA-TGTGCTCGCATCAT--ATTA-TTT-A-TCT---ATCTCT
-TGTGCACCTTTTG-TAGTCTTTGAAGTG--
----TTCGCAGTC----------------------AATGCGG----------TT--------------TGGGG---CTTG
GGC-----------TTTGC--GGTCCTTTCCCTG---
---------------TTTG------CTTCA---------AAG-----------------------GCTATG------TTT
TA-----------------------------TACA-CACTA----TTGAAGTTAC-AGAATGTC---TCTTATTGA-----
--C-CT---T-TGAGTCAGT--------AAATC-A-ATACAACTTTCAGCAACGGATCTCTTGGCTCTCCCATCGATGAA
GAACGCAGCGAAATGCGATAAGTAATGTGAATTGCAGAATTCAGTGAATCATCGAATCTTTGAACGCACCTTGCGCCCTT
TGGTATTCCGAAGGGCATGCCTGTTTGAGTGTC-ATTAAATTC-TCAA---------CCTTGCAA-----GC---TT---
--TTTTGC-TAG--------TGAGGC---TTGGAT-GTGA-GG---G-CTTTTGCTGGC-TTCC----TTCATTT-GGAT
GGTCTGCTCCCTTTAAATTCATTAGTGGAT-TCCT--TTGTGGA-----------TGG-TCACTTGG-TGTGATAA-TTA
TCTACGCCGC-CTGACTCTGAAACAAGACTTGTGGG-GA-CCTGCTCATAACCG------TCTC-TTC---AAGAGACA-
-ATCTAT--A-------TTGACAATTTGACCT-GCCTTCACTCTCGGTGTGCGCCAGCTTATCGTTGCGGTCAACAAGAT
GGACACGACCAAGGTTCG---GTGCATTATAA-GCC---------------CTAACGAATGAT------TTTGAT--GA
CACCTATTCACAGTGGAGCGAGGACCGTTTCAACGAAATCGTCAAGGAGACATCCACCTTCATTAAGAAGGTCGGCTACA
ACCCCAAATCTATTTCTTTCGTCCCTATCTCTGGCTGGCACGG-TGACAACAT--GCTTGAGGAGTCCACGAAGTATGAA
GTTCAGATTTGTATATCTACCGAACCTAACT-TATG-TTTGCG----------------CAGCATGCCCTGG-TTCAAGG
GCTGGACCAAGGAAAC---CAAAGGTGGT--GTCGTCAAGGGCAAGACTCTTCTT---GAAGCTATCG------------
-ATGCTATCGAGCCCCCGGTCCGGCCTGTGGAC-

Mycena_cf_pura_VIII_CBH216

ACTTGGTG-TTGA-GCTGGCT-CTA------TT--GGGCA-TGTGCTCGCGTCAT--ATTA-TTT-A-TCT---ATCTCT
-TGTGCACCTTTTG-TAGTCTTTGAAGTG--
----TTCGCAGTC----------------------AATGCGG----------TT--------------TGGGG---CTTG
GGC-----------TTTGC--GGTCCTTTCCCTG---
---------------TTTG------CTTCA---------AAG-----------------------GCTATG------TTT
TA-----------------------------TACA-CACTA----TTGAAGTTAC-AGAATGTC---TCTTATTGA-----
--C-CT---T-TGAGTCAGT--------AAATC-A-ATACAACTTTCAGCAACGGATCTCTTGGCTCTCCCATCGATGAA
GAACGCAGCGAAATGCGATAAGTAATGTGAATTGCAGAATTCAGTGAATCATCGAATCTTTGAACGCACCTTGCGCCCTT
TGGTATTCCGAAGGGCATGCCTGTTTGAGTGTC-ATTAAATTC-TCAA---------CCTTGCAA-----GC---TT---
--TTTTGC-TAG--------CGAGGC---TTGGAT-GTGA-GG---G-CTTTTGCTGGC-TTCC----TTTA-TT-GGAT
GGTCTGCTCCCTTTAAATTCATTAGTGGGA-TCCT--TTGTGGA-----------TGG-TCACTTGG-TGTGATAA-TTA
TCTACGCCGC-CTGACTCTGAAACAAGACTTGTGGG-GA-CCTGCTCATAACCG------TCTC-TTC---AAGGGACA-
-ATCTAT--A-------TTGACAATTTGACCT-GCCTTCACTCTCGGTGTGCGCCAGCTTATCGTTGCCGTCAACAAGAT
GGACACGACCAAGGTTCG---GTGCATTATAA-GCC---------------CTAATGAATAAT------TTTGAT--GA

CACCTATTCACAGTGGAGCGAGGACCGTTTCAACGAAATCGTTAAGGAGACGTCCACTTTCATTAAGAAGGTCGGCTACA
ACCCCAAATCTATTTCTTTCGTCCCTATCTCTGRCTGGCACGG-TGACAACAT--GCTTGAGGAGTCCACGAAGTATGAA
GTTCAGATTTGTATTTCTGCCGAACCTAACT-TATG-TTTGCGC--------------ACAGCATGCCCTGG-TTCAAGG
GCTGGACCAAGGAGAC---CAAAGGTGGT--GTCGTCAAGGGCAAGACTCTTCTT---GAAGCTATCG------------
-ATGCTATCGAGCCCCCGGTCCGGCCTGTGGAC-
Mycena_cf_pura_VIII_CBH402
ACTTGGTG-TTGA-GCTGGCT-CTA------TT--GGGCA-TGTGCTCGCGTCAT--ATTA-TTT-A-TCT---ATCTCT
-TGTGCACCTTTTG-TAGTCTTTGAAGTG---
----TTCGCAGTC----------------------AATGCGG----------TT--------------TGGGG---CTTG
GGC-----------TTTGC--GGTCCTTTCCCTG--
---------------TTTG------CTTCA---------AAG-----------------------GCTATG------TTT
TA----------------------------TACA-CACTA----TTGAAGTTAC-AGAATGTC---TCTTATTGA-----
--C-CT---T-TGAGTCAGT--------AAATC-A-ATACAACTTTCAGCAACGGATCTCTTGGCTCTCCCATCGATGAA
GAACGCAGCGAAATGCGATAAGTAATGTGAATTGCAGAATTCAGTGAATCATCGAATCTTTGAACGCACCTTGCGCCCTT
TGGTATTCCGAAGGGCATGCCTGTTTGAGTGTC-ATTAAATTC-TCAA---------CCTTGCAA-----GC---TT---
--TTTTGC-TAG--------CGAGGC---TTGGAT-GTGA-GG---G-CTTTTGCTGGC-TTCC----TTTR-TT-GGAT
GGTCTGCTCCCTTTAAATTCATTAGTGGGA-TCCT--TTGTGGA-----------TGG-TCACTTGG-TGTGATAA-TTA
TCTACGCCGC-CTGACTCTGAAACAAGACTTGTGGG-GA-CCTGCTCATAACCG------TCTC-TTC---AAGGGACA-
-ATCTAT--A-------TTGACAATTTGACCT-GCCTTCACTCTCGGTGTGCGCCAGCTTATCGTTGCCGTCAACAAGAT
GGACACGACCAAGGTTCG---GTGCATTATAA-GCC----------------CTAATGAATAAT------TTTGAT--GA
CACCTATTCACAGTGGAGCGAGGACCGTTTCAACGAAATCGTTAAGGAGACGTCCACTTTCATTAAGAAGGTCGGCTACA
ACCCCAAATCTATTTCTTTCGTCCCTATCTCTGGCTGGCACGG-TGACAACAT--GCTTGAGGAGTCCACGAAGTATGAA
GTTCAGATTTGTATTTCTGCCGAACCTAACT-TATG-TTTGCGC--------------ACAGCATGCCCTGG-TTCAAGG
GCTGGACCAAGGAGAC---CAAAGGTGGT--GTCGTCAAGGGCAAGACTCTTCTT---GAAGCTATCG------------
-ATGCTATCGAGCCCCCGGTCCGGCCTGTGGAC-
Mycena_cf_pura_VII_IS10_11_2000
------------------------A------TT--GGGCA-TGTGCTCGCGTCAT--ATTA-TTT-A-TCT---ATCTCT
-TGTGCACCTTTTG-TAGTCTTTGAAGTG---
----TTCGCAGTC----------------------AATGCGG----------TT--------------TGGGG---CTTG
GGC-----------TTTGC--GGTCCTTTCCCTG--
---------------TTTG------CTTCA---------AAG-----------------------GCTATG------TTT
TA----------------------------TACA-CACTA----TTGAAGTTAC-AGAATGTC---TCTTATTGA-----
--C-CT---T-TGAGTCAGT--------AAATC-A-ATACAACTTTCAGCAACGGATCTCTTGGCTCTCCCATCGATGAA
GAACGCAGCGAAATGCGATAAGTAATGTGAATTGCAGAATTCAGTGAATCATCGAATCTTTGAACGCACCTTGCGCCCTT
TGGTATTCCGAAGGGCATGCCTGTTTGAGTGTC-ATTAAATTC-TCAA---------CCTTGCAA-----GC---TT---
--TTTTGC-TGG--------CGAGGC---TTGGAT-GTGA-GG---G-CTTTTGCTGGC-TTCC----TTCA-TT-GGAT
GGTCTGCTCCCTTTAAATTCATTAGTGGGA-TCCT--TTGTGGA-----------TGG-TCACTTGG-TGTGATAA-TTA
TCTACGCCGC-CTGACTCTGAAACAAGACTTGTGGG-GA-CCTGCTCATAACCG------TCTC-TTC---AAGGGACA-
-ATCTAT--A-------TTGACAA--

Mycena_cf_pura_IX_CBH166

ACTTGGTG-TTGA-GCTGGCC-CTA------TT-GGGGCA-TGTGCTCGCATCAT--ATTA-TTT-A-TCT---ATCTCT
-TGTGCACCTTTTG-TAGTCTTTGAAGTG---
----CTCGCAGTC----------------------AATGCGG---------TTT--------------TGGGA---CTTG
GGC-----------TTTTT--GCCCTTGTCCCTG--
---------------TTCG------CTTCA---------AAG-----------------------GCTATG-----TTTT
TA----------------------------TACA-CACTA--T-TTGAAGTCAC-AGAATGTC---TCTTATTGA-----
--C-CT---T-TGAGTCAGT--------AAATC-A-ATACAACTTTCAGCAACGGATCTCTTGGCTCTCCCATCGATGAA
GAACGCAGCGAAATGCGATAAGTAATGTGAATTGCAGAATTCAGTGAATCATCGAATCTTTGAACGCACCTTGCGCCCTT
TGGTATTCCGAAGGGCATGCCTGTTTGAGTGTC-ATTAAATTC-TCAA---------CCTTGCAA-----GC--TTT---
--TTTTGC-TAG--------TGAGGC---TTGGAT-GTGA-GG---G-TTTTTGCTGGC-TTCC----TTCAGTT-GGAT
GGTCTGCTCCCTTTAAATTCATTAGTGGAT-TCCT--TTGTGGA-----------TGG-TCACTTGG-TGTGATAA-TTA
TCTACGCCGC-CTGACTCTGATACAAGACTTGTGGG-GA-CCTGCTTATAACCG------TCTC-TTC---AAGAGACA-
-ATCTATATA-------TTGACAATTTGACCT-GCGTTCACTCTCGGTGTGCGCCAGCTTATCGTTGCGGTCAACAAGAT
GGACACGACCAAGGTTCG---GTGCATTATAA-GCC------------------AACGAATGAT------TTTGAT--GA
CACCTAT--ACAGTGGAGCGAGGACCGTTACAACGAAATCGTCAAGGAGACGTCCACTTTCATTAAGAAGGTCGGCTACA
ACCCCAAAGCGATTTCTTTCGTCCCTATTTCTGGCTGGCACGG-CGACAACAT--GCTTGAGGAGTCTACCAAGTATGAA
AGTTCAGATTGTGTTTCTGTCGAAGCCAACT-CATG-TTTGCG---------------CAGCATGCCCTGG-TTCAAGG
GCTGGACCAAGGAGAC---CAAAGGTGGT--GTCATCAAGGGCAAGACTCTTCTT---GAAGCTATCG------------
-ATGCTATCGAACCCCCGGTTCGGCCTGTGGAC-

Mycena_cf_pura_IX_CBH358

ACTTGGTG-TTGA-GCTGGCC-CTA------TT-GGGGCA-TGTGCTCGCATCAT--ATTA-TTT-A-TCT---ATCTCT
-TGTGCACCTTTTG-TAGTCTTTGAAGTG---
----CTCGCAGTC----------------------AATGCGG---------TTT--------------TGGGA---CTTG
GGC-----------TTTTT--GCCCTTGTCCCTG--
---------------TTCG------CTTCA---------AAG-----------------------GCTATG------TTT
TA----------------------------TACA-CACTA--T-TTGAAGTCAC-AGAATGTC---TCTTATTGA-----
--C-CT---T-TGAGTCAGT--------AAATC-A-ATACAACTTTCAGCAACGGATCTCTTGGCTCTCCCATCGATGAA
GAACGCAGCGAAATGCGATAAGTAATGTGAATTGCAGAATTCAGTGAATCATCGAATCTTTGAACGCACCTTGCGCCCTT
TGGTATTCCGAAGGGCATGCCTGTTTGAGTGTC-ATTAAATTC-TCAA---------CCTTGCAA-----GC--TTT---
--TTTTGC-TAG--------TGAGGC---TTGGAT-GTGA-GG---G-TTTTTGCTGGC-TTCC----TTCAGTT-GGAT
GGTCTGCTCCCTTTAAATTCATTAGTGGAT-TCCT--TTGTGGA-----------TGG-TCACTTGG-TGTGATAA-TTA
TCTACGCCGC-CTGACTCTGATACAAGACTTGTGGG-GA-CCTGCTTATAACCG------TCTC-TTC---AAGAGACA-
-ATCTATATA-------TTGACAATTTGACCT-GCGTTCACTCTCGGTGTGCGCCAGCTTATCGTTGCGGTCAACAAGAT
GGACACGACCAAGGTTCG---GTGCATTATAA-GCC------------------AACGAATGAT------TTTGAT--GA
CACCTAT--ACAGTGGAGCGAGGACCGTTACAACGAAATCGTCAAGGAGACGTCCACTTTCATTAAGAAGGTCGGCTACA
ACCCCAAAGCGATTTCTTTCGTCCCTATTTCTGGCTGGCACGG-CGACAACAT--GCTTGAGGAGTCTACCAAGTATGAA
AGTTCAGATTGTGTTTCTGTCGAAGCCAACT-CATG-TTTGCG---------------CAGCATGCCCTGG-TTCAAGG
GCTGGACCAAGGAGAC---CAAAGGTGGT--GTCATCAAGGGCAAGACTCTTCTT---GAAGCTATCG------------
-ATGCTATCGAACCCCCGGTTCGGCCTGTGGAC-

FLF1223

ACCTGGTG-TTGA-GCTGGCC-CTA------TC--GGGCA-TGTGCTCGCATCAT--ATTA-TTT-A-TCT---ATCTCT
-TGTGCACCTTTTG-TAGTCTTTGAAGTG---

----TTCGCAGTCA---------------------AATGCGG----------TT--------------TGGGG---CTTG
GGC-----------TTCGC--GGTCCTTTCCCTGT---
---------------TTTG------CTTCA---------AAG-----------------------GCTATG------TTT
TA----------------------------TACA-CACTA--T-TTGAAGTTAC-AGAATGTC---TCTTATTGA-----
--C-CT---T-TGAGTCAGT--------AAATC-A-ATACAACTTTCAGCAACGGATCTCTTGGCTCTCCCATCGATGAA
GAACGCAGCGAAATGCGATAAGTAATGTGAATTGCAGAATTCAGTGAATCATCGAATCTTTGAACGCACCTTGCGCCCTT
TGGTATTCCGAAGGGCATGCCTGTTTGAGTGTC-ATTAAATTC-TCAA---------CCTTGCCA-----GC--------
--TTTTGC-TAG--------TGAGGC---TTGGAT-GTGA-GG---G-C-TTTGCTGGC-TTCC----TTCAGTT-GGAT
GGTCTGCTCCCTTAAAATTCATTAGTGGGA-TCCT--TTGTGGA-----------TGG-TCACTTGG-TGTGATAA-TTA
TCTACGCCGC-CTGACTCTGAAACAAGACTTGTGGG-GA-CCTGCTCATAACCG------TCTC-TTC---ATGAGACA-
-ATTTTT--A-------TTGACAATTTGACCT--

FLF1244
ACCTGGTG-TTGA-GCTGGCC-CTA------TC--GGGCA-TGTGCTCGCATCAT--ATTA-TTT-A-TCT---ATCTCT
-TGTGCACCTTTTG-TAGTCTTTGAAGTG---
----TTCGCAGTCA---------------------AATGCGG----------TT--------------TGGGG---CTTG
GGC-----------TTCGC--GGTCCTTTCCCTGT---
---------------TTTG------CTTCA---------AAG-----------------------GCTATG------TTT
TA----------------------------TACA-CACTA--T-TTGAAGTTAC-AGAATGTC---TCTTATTGA-----
--C-CT---T-TGAGTCAGT--------AAATC-A-ATACAACTTTCAGCAACGGATCTCTTGGCTCTCCCATCGATGAA
GAACGCAGCGAAATGCGATAAGTAATGTGAATTGCAGAATTCAGTGAATCATCGAATCTTTGAACGCACCTTGCGCCCTT
TGGTATTCCGAAGGGCATGCCTGTTTGAGTGTC-ATTAAATTC-TCAA---------CCTTGCCA-----GC--------
--TTTTGC-TAG--------TGAGGC---TTGGAT-GTGA-GG---G-C-TTTGCTGGC-TTCC----TTCAGTT-GGAT
GGTCTGCTCCCTTAAAATTCATTAGTGGGA-TCCT--TTGTGGA-----------TGG-TCACTTGG-TGTGATAA-TTA
TCTACGCCGC-TTGACTCTGAAACAAGACTTGTGGG-GA-CCTGCTCATAACCG------TCTC-TTC---ATGAGACA-
-ATTTTT--A-------TTGACAATTTGACCT-GCCTTCACTCTCGGGGTGCGCCAGCTCATCGTTGCGGTCAACAAGAT
GGACACGACCAAGGTTCC---GTGCATTATCA-GCC----------------CTAACGAATAGC------TTTGGT--GA
CACCGATTCACAGTGGAGCGAGGACCGTTTCAACGAAATCATCAAGGAGACGTCGACTTTCATTAAGAAGGTCGGCTACA
ACCCCAAATCTATTGCTTTCGTCCCCATCTCTGGCTGGCACGG-TGACAACAT--GCTCGAGGAGTCCCCGAAGTATGAA
GTTC--ATTTGTATTTCCACCGAACCTAACT-TATG-TTTGCG----------------CAGCATGCCCTGG--ACAAGG
GCTGGACCAAGGAGAC---CAAAGCTGGT--GTCATCAAGGG-AAGACTCTTCTT---GAAGCTATCG------------
-ATGGTATCGAGCCCCCGGTCCGG-CTACGGAC-
Mycena_cf_pura_X_BAP165A
ACTTGGTG-TTGA-GCTGGCC-CTA------TT-GGGGCA-TGTGCTCGCATCAT--ATTA-TTT-T-ACT---ATCTCT
-TGTGCACCTTTTG-TAGTCTTTGAAGTG---
----TTCGCAGTC----------------------AATGCGG---------TTT--------------TGGGG---CTTG
GGC-----------TTTGCT-GTCCTTTTCCCTG--
---------------TTTG------CTTCA---------AAG-----------------------GCTATG-----TTTT
TA----------------------------TACA-CACTA----TTGAAGTTAC-AGAATGTC---TCTTATTGA-----

--C-TT---TTTGAGTCAGT--------AAATC-T-ATACAACTTTCAGCAACGGATCTCTTGGCTCTCCCATCGATGAA
GAACGCAGCGAAATGCGATAAGTAATGTGAATTGCAGAATTCAGTGAATCATCGAATCTTTGAACGCACCTTGCGCCCTT
TGGTATTCCGAAGGGCATGCCTGTTTGAGTGTC-ATTAAATTC-TCAA---------CCTTGTAA-----GC-TTTT---
----TTGC-TTG--------CGAGGC---TTGGAT-GTGA-GG---GCTTTTTGCTGGC-TTCCTTCCTTCAGTT-GGAT
GGTCTGCTCCCTTTAAATTCATTAGTGGGA-TCCT--TTGTGGA-----------CGG-TCACTTGG-TGTGATAA-TTA
TCTACGCCGC-CTGACTTTGAAACAAGACTTGTGGG-AA-CCTGCTTATAACCG------TCTC-TTTG--GAGACAAT-
---------CT------TTGACTATTTGACCT--
----------AAGGTTCG---GTGCATTAAAA-GCC----------------ATAACGAATAAR------TTTGAT--GA
CACCTATTCACAGTGGAGTGAGGACCGTTTCAACGAAATCGTCAAGGAGACGTCGACTTTCATTAGGAAGGTCGGCTACA
ACCCCAAATGCATTACTTTCGTCCCTATTTCTGGCTGGCACGG-TGACAACAT--GCTTGAGGAGTCCACGAAGTATGAA
GTTCAGATTTGCATTTCTACCGAACCCAACT-TATG-TTTGCT----------------CAGCATGCCCTGG-TTCAAGG
GCTGGACCAAGGAGAC---CAAAGCTGGT--GTCGTCAAGGGCAAGACTCTTCTT---GAAGCTATCG------------
-ATGCTATTGAGCCCCCCGTCCGGCCTAGTGAC-

FLF1804

CTTTGGTG-TTGA-GCTGGCC-CTA------TT--GGGCA-TGTGCTTGCATCAT--ATTA-TTT-A-TCT---ATCACT
-TGTGCACCTTTTG-TAGTCTTTGAAGTG---
----TTCGCAGTC----------------------AATGCGG---------TTT--------------TGGGG---CTTG
GGC-----------TTTGCT-GCCCTTTCCCCTG--
---------------TTTG------CTTCA---------AAG----------------------GCTATG-----TTTT
TA----------------------------TACA-CACTA----TTAAAGTTAC-AGAATGTC---TCTTATTGA-----
--C-TT---T-TGAGTCAGTG-------AAATC-T-ATACAACTTTCAGCAACGGATCTCTTGGCTCTCCCATCGATGAA
GAACGCAGCGAAATGCGATAAGTAATGTGAATTGCAGAATTCAGTGAATCATCGAATCTTTGAACGCACCTTGCGCCCTT
TGGTATTCCGAAGGGCATGCCTGTTTGAGTGTC-ATTAAATTC-TCAA---------CCTTGAAA-----GC--------
--TTTTGC-TTT--------CGAGGC---TTGGAT-GTGA-GG---G-TTTTTGCTGGC-TTCC----TTCAGTT-GGAT
GGTCTGCTCCCTTTAAATTCATTAGTGGAT-TCCT--TTGTGGA-----------CGG-TCACTTGG-TGTGATAA-TTA
TCTACGCCGC-CTGACTCTGAAACAAGACTTATGGG-AC-CCTGCTTATAACTG------TCTC-TTCA--GAGACAAT-
---------TT-----CTTGACAATTTGACCT-GCCTTCACTCTCGGTGTGCGCCAGCTTATCGTTGCGGTCAACAAGAT
GGACACGACCAAGGTT-G---GTGCATTATAA-GCC----------------TTAACGAATTATT---TTTTTTAT--GA
CACCTATTCACAGTGGAGTGAGGACCGTTTCAACGAAATCATCAAGGAGACGTCCAGTTTCATTAAGAAGGTCGGCTTCA
ACCCCAAATCCGTTTGTTTCGTCCCTATATCTGGCTGGCACGG-TGACAACAT--GCTTGAGGAGTCCACGAAGTATGAA
GTTCAGATTTGTATTTTTACCGAACCCAACT-TATG-TTCGCG----------------CAGCATGCCCTGG-TTCAAGG
GCTGGACCAAGGAGTC---CAAAACTGGT--GTCGTCAAGGGCAAGACTCTTCTT---GAAGCTATCG------------
-ATGCTATGGAACCCCCGACACGTCCTACGGAC-

MSX1804

CTTTGGTG-TTGA-GCTGGCC-CTA------TT--GGGCA-TGTGCTTGCATCAT--ATTA-TTT-A-TCT---ATCACT
-TGTGCACCTTTTG-TAGTCTTTGAAGTG---
----TTCGCAGTC----------------------AATGCGG---------TTT--------------TGGGG---CTTG
GGC-----------TTTGCT-GCCCTTTCCCCTG--
---------------TTTG------CTTCA---------AAG----------------------GCTATG-----TTTT
TA----------------------------TACA-CACTA----TTAAAGTTAC-AGAATGTC---TCTTATTGA-----
--C-TT---T-TGAGTCAGTG-------AAATC-T-ATACAACTTTCAGCAACGGATCTCTTGGCTCTCCCATCGATGAA
GAACGCAGCGAAATGCGATAAGTAATGTGAATTGCAGAATTCAGTGAATCATCGAATCTTTGAACGCACCTTGCGCCCTT
TGGTATTCCGAAGGGCATGCCTGTTTGAGTGTC-ATTAAATTC-TCAA---------CCTTGAAA-----GC--------
--TTTTGC-TTT--------CGAGGC---TTGGAT-GTGA-GG---G-TTTTTGCTGGC-TTCC----TTCAGTT-GGAT

GGTCTGCTCCCTTTAAATTCATTAGTGGAT-TCCT--TTGTGGA-----------CGG-TCACTTGG-TGTGATAA-TTA
TCTACGCCGC-CTGACTCTGAAACAAGACTTATGGG-AC-CCTGCTTATAACTG------TCTC-TTCA--GAGACAAT-
---------TT-----CTTGACAATTTGACCT-GCCTTCACTCTCGGTGTGCGCCAGCTTATCGTTGCGGTCAACAAGAT
GGACACGACCAAGGTT-G---GTGCATTATAA-GCC----------------TTAACGAATTATT---TTTTTTAT--GA
CACCTATTCACAGTGGAGTGAGGACCGTTTCAACGAAATCATCAAGGAGACGTCCAGTTTCATTAAGAAGGTCGGCTTCA
ACCCCAAATCCGTTTGTTTCGTCCCTATATCTGGCTGGCACGG-TGACAACAT--GCTTGAGGAGTCCACGAAGTATGAA
GTTCAGATTTGTATTTTTACCGAACCCAACT-TATG-TTCGCG----------------CAGCATGCCCTGG-TTCAAGG
GCTGGACCAAGGAGTC---CAAAACTGGT--GTCGTCAAGGGCAAGACTCTTCTT---GAAGCTATCG------------
-ATGCTATGGAACCCCCGACACGTCCTACGGAC-

FLF1816

ACTTGGTG-TTGA-GCTGGCC-CTA------TT-GGGGCA-TGTGCTCGCATCAT--ATTA-TTT-A-TCT---ATCTCT
-TGTGCACCTTTTG-TAGTCTTTGAAGTGG--
---CTTCGCAGTC----------------------AATGCGG---------TTT--------------TGGGG---CTTG
GGTG--------C-TTTGCT-GCCCTTGTCCCTGT--
---------------TCAC------TTTCA---------AAG-----------------------GCTATG-----TTTT
TA----------------------------TACA-CACTA--T-CAAAAGTTAC-AGAATGTC---TCTTATTGA-----
--C-CT---TTCGAGTCAGT--------AAATC-T-ATACAACTTTCAGCAACGGATCTCTTGGCTCTCCCATCGATGAA
GAACGCAGCGAAATGCGATAAGTAATGTGAATTGCAGAATTCAGTGAATCATCGAATCTTTGAACGCACCTTGCGCCCTT
TGGTATTCCGAAGGGCATGCCTGTTTGAGTGTC-ATTAAATTC-TCAA---------CCTTGCAA-----GC-TTTT---
--TTTTGC-TTG--------TGAGGC---TTGGAT-GTGA-GG---G-TTTTTGCTGGC-TTCCT---TTAAATT-GGAT
GGTCTGCTCCCTTTAAATTCATTAGTGGGA-TCCT--TTGTGGA-----------TGG-TCACTTGG-TGTGATAA-TTA
TCTACGCCGC-CTGACTCTGAAACAAGACTTGTGGG-AA-CCTGCTTATAACCG------TCTCTTTTG--GAGACTAA-
---------CT------TTGACAATTTGACCT-GCCTTCACTCTCGGTGTGCGCCAGCTTATCGTTGCGGTCAACAAGAT
GGACACAACCAAGGTTCG---GTGCATTATAA-TATAA-----------GCCTTAACGAATAAT------TTTGAT--GA
CACGTGTTCACAGTGGAGTGAGGACCGTTTCAACGAAATCATCAAGGAGACGTCCACTTTCATTAAGAAGGTCGGCTACA
ACCCCAAATCTATTTCTTTCGTCCCCATTTCTGGCTGGCACGG-TGACAACAT--GCTTGAGGAGTCCACGAAGTATAAA
G-----ATTTGTATTTCTACCGAACCCAACT-TATG-TTTGCG----------------CAGCATGCCCTGG-TTCAAGG
GCTGGACCAAGGAGATCGGCAAAGGTGGG--GTCATCAAGGGCAAGACTCTTCTT---GAAGCTATCG------------
-ATGCTATCGAGCCCCCGGTCCGGCCTAGCGAC-

MSX1816

ACTTGGTG-TTGA-GCTGGCC-CTA------TT-GGGGCA-TGTGCTCGCATCAT--ATTA-TTT-A-TCT---ATCTCT
-TGTGCACCTTTTG-TAGTCTTTGAAGTGG--
---CTTCGCAGTC----------------------AATGCGG---------TTT--------------TGGGG---CTTG
GGTG--------C-TTTGCT-GCCCTTGTCCCTGT--
---------------TCAC------TTTCA---------AAG-----------------------GCTATG-----TTTT
TA----------------------------TACA-CACTA--T-CAAAAGTTAC-AGAATGTC---TCTTATTGA-----
--C-CT---TTCGAGTCAGT--------AAATC-T-ATACAACTTTCAGCAACGGATCTCTTGGCTCTCCCATCGATGAA
GAACGCAGCGAAATGCGATAAGTAATGTGAATTGCAGAATTCAGTGAATCATCGAATCTTTGAACGCACCTTGCGCCCTT
TGGTATTCCGAAGGGCATGCCTGTTTGAGTGTC-ATTAAATTC-TCAA---------CCTTGCAA-----GC-TTTT---
--TTTTGC-TTG--------TGAGGC---TTGGAT-GTGA-GG---G-TTTTTGCTGGC-TTCCT---TTAAATT-GGAT
GGTCTGCTCCCTTTAAATTCATTAGTGGGA-TCCT--TTGTGGA-----------TGG-TCACTTGG-TGTGATAA-TTA
TCTACGCCGC-CTGACTCTGAAACAAGACTTGTGGG-AA-CCTGCTTATAACCG------TCTCTTTTG--GAGACTAA-
---------CT------TTGACAATTTGACCT-GCCTTCACTCTCGGTGTGCGCCAGCTTATCGTTGCGGTCAACAAGAT
GGACACAACCAAGGTTCG---GTGCATTATAA-TATAA-----------GCCTTAACGAATAAT------TTTGAT--GA

CACGTGTTCACAGTGGAGTGAGGACCGTTTCAACGAAATCATCAAGGAGACGTCCACTTTCATTAAGAAGGTCGGCTACA
ACCCCAAATCTATTTCTTTCGTCCCCATTTCTGGCTGGCACGG-TGACAACAT--GCTTGAGGAGTCCACGAAGTATAAA
G-----ATTTGTATTTCTACCGAACCCAACT-TATG-TTTGCG----------------CAGCATGCCCTGG-TTCAAGG
GCTGGACCAAGGAGATCGGCAAAGGTGGG--GTCATCAAGGGCAAGACTCTTCTT---GAAGCTATCG------------
-ATGCTATCGAGCCCCCGGTCCGGCCTAGCGAC-

Mycena_lucisnieblae_ACP2140
ACTTGGTG-CTGA-GCTGGCC-CTA-----GTC--GGGCA-TGTGCTTGCATCAT--ATTA-TTT-A-TCT---ACCACC
-TGTGCACCTTTTG-TAGTCCTTGAAGTG---
----TTCGCAGTC----------------------AATGCGGT-------TTTT--------------TGGGT---CTTG
GGC-----------TTTGCT-GCCCTTTATCCCTG---
---------------TTTG------CTTCA---------AGG-----------------------ACTATG-----TTTT
CA----------------------------CACA-CACTA--T-TTGAAGTTAC-AGAATGTC---TCTTATTGA-----
--C-TTG----CGAGTCAGT--------AAATC-T-ATACAACTTTCAGCAACGGATCTCTTGGCTCTCCCATCGATGAA
GAACGCAGCGAAATGCGATAAGTAATGTGAATTGCAGAATTCAGTGAATCATCGAATCTTTGAACGCACCTTGCGCCCTT
TGGTATTCCGAAGGGCATGCCTGTTTGAGTGTC-ATTAAATTC-TCAA---------CCTTGCAA-----AT--------
---TTTGT-TTG--------CGAGGC---TTGGAT-GTGA-GG---G-TTTTTGCTGGC-TTCC----TTTG----GGAT
GGTCTGCTCCCTTGAAATTCATTAGTGGGA-TCCT--TTGTGGA-----------TGG-TCACTTGG-TGTGATAA-TTA
TCTACGCCGC-CTGACTCTGATGCAAGACTTGTGGG-AA-CCTGCTTATAACCAGCCGTCTCTT-TTTG--GAGAGACA-
---CTAT--CT------TTGACAATTTGACCT-GCCTTCACTCTCGGTGTCCGCCAGCTTATCGTCGCGGTCAACAAGAT
GGACACGACCAAGGTTCG----TGCACTATAA-GCC----------------CAAACGAATAAT------TTTGAT--GA
CACCTATTCACAGTGGAGTGAGGACCGTTTCAACGAAATCGTCAAGGAGACGTCCGGTTTCATTAAGAAGGTCGGCTACA
ACCCCAAAACTATTTCTTTTGTCCCTATCTCTGGCTGGCACGG-TGACAACAT--GCTTGAGGAGTCTACGAAGTATGAA
GTTCGGATTTGTATCTCTG---

Mycena_lucisnieblae_ACP2139
ACTTGGTG-CTGA-GCTGGCC-CTA-----GTC--GGGCA-TGTGCTTGCATCAT--ATTA-TTT-A-TCT---ACCACC
-TGTGCACCTTTTG-TAGTCCTTGAAGTG---
----TTCGCAGTC----------------------AATGCGGT-------TTTT--------------TGGGT---CTTG
GGC-----------TTTGCT-GCCCTTTATCCCTG---
---------------TTTG------CTTCA---------AGG-----------------------ACTATG-----TTTT
CA----------------------------CACA-CACTA--T-TTGAAGTTAC-AGAATGTC---TCTTATTGA-----
--C-TTG----CGAGTCAGT--------AAATC-T-ATACAACTTTCAGCAACGGATCTCTTGGCTCTCCCATCGATGAA
GAACGCAGCGAAATGCGATAAGTAATGTGAATTGCAGAATTCAGTGAATCATCGAATCTTTGAACGCACCTTGCGCCCTT
TGGTATTCCGAAGGGCATGCCTGTTTGAGTGTC-ATTAAATTC-TCAA---------CCTTGCAA-----AT--------
---TTTGT-TTG--------CGAGGC---TTGGAT-GTGA-GG---G-TTTTTGCTGGC-TTCC----TTTG----GGAT
GGTCTGCTCCCTTGAAATTCATTAGTGGGA-TCCT--TTGTGGA-----------TGG-TCACTTGG-TGTGATAA-TTA
TCTACGCCGC-CTGACTCTGATGCAAGACTTGTGGG-AA-CCTGCTTATAACCAGCCGTCTCTT-TTTG--GAGAGACA-
---CTAT--CT------TTGACAATTTGACCT-GCCTTCACTCTCGGTGTCCGCCAGCTTATCGTCGCGGTCAACAAGAT
GGACACGACCAAGGTTCG----TGCACTATAA-GCC----------------CAAACGAATAAT------TTTGAT--GA
CACCTATTCACAGTGGAGTGAGGACCGTTTCAACGAAATCGTCAAGGAGACGTCCGGTTTCATTAAGAAGGTCGGCTACA
ACCCCAAAACTATTTCTTTTGTCCCTATCTCTGGCTGGCACGG-TGACAACAT--GCTTGAGGAGTCTACGAAGTATGAA
GTTCGGATTTGTATCTCTGCCGA-CCCAACT-TATG-TTTGCG----------------CAGCATGCCCTGG-TTCAAGG
GCTGGACCAAGGAGGT---GAAAGGTGGT--GTCGTCAAGGGCAAGACTCTTCTC---GAAGCCATCG------------

-ATGCTATCGAGCCCCCGGTCCGGCCTGTGGAC-
Mycena_pearsoniana_CBH068
ACTTGGTG-TTGA-GCTGGCC-CTA-----GTC--GGGCA-TGTGCTTGCATCAT--ATTA-TTT-A-TCT---ATCTCT
-TGTGCACCTTTTG-TAGTCTTTGAAGCA---
----TTCGCAGTC----------------------AATGCGG---------TTT--------------TGGGT---CTTG
GGC-----------TTCGCT-GCTCTTTTCCCTGT--
---------------TTTG------CTTCA---------AAG-----------------------GCTATG-----TTTT
CA----------------------------TACA-CACTA--T-TTGAAGTTAC-AGAATGTC---TCTTATTGA-----
--C-TC---T-CGAGTCAGT--------AAATC-T-ATACAACTTTCAGCAACGGATCTCTTGGCTCTCCCATCGATGAA
GAACGCAGCGAAATGCGATAAGTAATGTGAATTGCAGAATTCAGTGAATCATCGAATCTTTGAACGCACCTTGCGCCCTT
TGGTATTCCGAAGGGCATGCCTGTTTGAGTGTC-ATTAAATTC-TCAA---------CCTTGCAA-----AT--------
---TTTGT-TTG--------TGAGGC---TTGGAT-GTGA-GG---G--TTTTGCTGGC-TTCC-----TTTGTT-GGAT
GGTCTGCTCCCTTTAAATTCATTAGTGGGA-TCCT--TTGTGGA-----------TGG-TCGCTTGG-TGTGATAA-TTA
TCTACGCCGC-TTGACTCTGACGCAAGACTTGTGGG-AA-CCTGCTTATAACCG------TCTC-TTCA--GAGA-----
---CTAT-CTT------TTGACAATTTGACCT-GCTTTCACTCTCGGTGTCCGCCAGCTCATCGTCGCGGTCAACAAGAT
GGACACGACCAAGGTTTG---GTGCACTATAA-GCG----------------CTAACGAATAAT------TTCGAT--GA
CACCTATTCACAGTGGAGTGAGGACCGTTTCAACGAAATCGTCAAGGAGACGTCCACCTTCATTAAGAAGGTCGGCTACA
ACCCCAAAAATATTTGTTTTGTCCCTATCTCTGGCTGGCACGG-TGACAACAT--GCTTGAGGAGTCCACGAAGTATGGA
G-----ATTTGTGTTTCTGCCGAATCCGACT-TATG-TTTGCG----------------CAGCATGCCCTGG-TTCAAGG
GCTGGACCAAGGAGAC---CAAAGGTGGT--GTCGTCAAGGGCAAGACTCTTCTT---GAAGCTATCG------------
-ATGCTATTGAGCCCCCGGTCCGGCCTGTGGAC-
Mycena_pearsoniana_LK880_2002
ACTTGGTG-TTGA-GCTGGCC-CTA-----GTC--GGGCA-TGTGCTTGCATCAT--ATTA-TTT-A-TCT---ATCTCT
-TGTGCACCTTTTG-TAGTCTTTGAAGCA---
----TTCGCAGTC----------------------AATGCGG---------TTT--------------TGGGT---CTTG
GGC-----------TTCGCT-GCTCTTTTCCCTGT--
---------------TTTG------CTTCA---------AAG-----------------------GCTATG-----TTTT
CA----------------------------TACA-CACTA--T-TTGAAGTTAC-AGAATGTC---TCTTATTGA-----
--C-TC---T-CGAGTCAGT--------AAATC-T-ATACAACTTTCAGCAACGGATCTCTTGGCTCTCCCATCGATGAA
GAACGCAGCGAAATGCGATAAGTAATGTGAATTGCAGAATTCAGTGAATCATCGAATCTTTGAACGCACCTTGCGCCCTT
TGGTATTCCGAAGGGCATGCCTGTTTGAGTGTC-ATTAAATTC-TCAA---------CCTTGCAA-----AT--------
---TTTGT-TTG--------TGAGGC---TTGGAT-GTGA-GG---G--TTTTGCTGGC-TTCC-----TTTGTT-GGAT
GGTCTGCTCCCTTTAAATTCATTAGTGGGA-TCCT--TTGTGGA-----------TGG-TCGCTTGG-TGTGATAA-TTA
TCTACGCCGC-TTGACTCTGACGCAAGACTTGTGGG-AA-CCTGCTTATAACCG------TCTC-TTCA--GAGA-----
---CTAT-CTT------TTGACAATTTGACCT-----------TCGGTGTCCGCCAGCTCATCGTCGCGGTCAACAAGAT
GGACACGACCAAGGTTTG---GTGCACTATAA-GCG----------------CTAACGAATAAT------TTCGAT--GA
CACCTATTCACAGTGGAGTGAGGACCGTTTCAACGAAATCGTCAAGGAGACGTCCACCTTCATTAAGAAGGTCGGCTACA
ACCCCAAAAATATTTGTTTTGTCCCTATCTCTGGCTGGCACGG-TGACAACAT--GCTTGAGGAGTCCACGAAGTATGGA
G-----ATTTGTGTTTCTGCCGAATCCGACT-TATG-TTTGCG----------------CAGCATGCCCTGG-TTCAAGG
GCTGGACCAAGGAGAC---CAAAGGTGGT--GTCGTCAAGGGCAAGACTCTTCTT---GAAGCTATCG------------
-ATGCTATTGAGCCCCCGGTCCGGCCTGTGGAC-
Mycena_shengshanensis_FFAAS0424
ACTTGGTG-TTGA-GCTGGCC-CTA-----GTC--GGGCA-TGTGCTTGCATCAT--ATTA-TTT-A-TCT---ATCTCT
-TGTGCACCTTTTG-TAGTCTTTGAAGCA---

----TTCGCAGTC----------------------AATGCGG---------TTT--------------TGGGT---CTTG
GGC-----------TTTGCT-GCTCTTTTCCCTGT---
---------------TTTG------CTTCA---------AAG-----------------------GCTATG-----TTTT
CA----------------------------TACA-CACTA--T-TTGAAGTTAC-AGAATGTC---TCTTATTGA-----
--C-TC---T-CGAGTCAGT--------AAATC-T-ATACAACTTTCAGCAACGGATCTCTTGGCTCTCCCATCGATGAA
GAACGCAGCGAAATGCGATAAGTAATGTGAATTGCAGAATTCAGTGAATCATCGAATCTTTGAACGCACCTTGCGCCCTT
TGGTATTCCGAAGGGCATGCCTGTTTGAGTGTC-ATTAAATTC-TCAA---------CCTTGCAG-----AC--------
---TTTGT-TTG--------CGAGGC---TTGGAT-GTGA-GG---G--TTTTGCTGGC-TTCC-----TTTGTT-GGAT
GGTCTGCTCCCTTTAAATTCATTAGTGGGA-TCCT--TTGTGGA-----------TGG-TCGCTTGG-TGTGATAA-TTA
TCTACGCCGC-TTGACTCTGACGCAAGACTTGTGGG-AA-CCTGCTTATAACCG------TCTC-TTCG--GAGA-----
---CTAT-CTT------TTGACAATTTGACCT-GCTTTCACTCTCGGTGTCCGCCAGCTCATCGTCGCGGTCAACAAGAT
GGACACGACCAAGGTTTG---GTGCACTATAA-GCG----------------CTAACGAATAAT------TTCGAT--GA
CACCTATTCACAGTGGAGTGAGGACCGTTTCAACGAAATCGTCAAGGAGACGTCCACCTTCATTAAGAAGGTCGGCTACA
ACCCCAAAAATATTTGTTTTGTCCCTATCTCTGGCTGGCACGG-TGACAACAT--GCTTGAGGAGTCCACGAAGTATGGA
G-----ATTTGTGTTTCTGCCGAATCCGACT-TATG-TTTGCG----------------CAGCATGCCCTGG-TTCAAGG
GCTGGACCAAGGAGAC---CAAAGGTGGT--GTCGTCAAGGGCAAGACTCTTCTT---GAAGCTATCG------------
-ATGCTATTGAGCCCCCGGTCCGGCCTGTGGAC-

Mycena_shengshanensis_FFAAS0425

ACTTGGTG-TTGA-GCTGGCC-CTC-----GTC--GGGCA-TGTGCTTGCATCAT--ATTA-TTT-A-TCT---ATCTCT
-TGTGCACCTTTTG-TAGTCTTTGAAGCA---
----TTCGCAGTC----------------------AATGCGG---------TTT--------------TGGGT---CTTG
GGC-----------TTTGCT-GCTCTTTTCCCTGT---
---------------TTTG------CTTCA---------AAG-----------------------GCTATG-----TTTT
CA----------------------------TACA-CACTA--T-TTGAAGTTAC-AGAATGTC---TCTTATTGA-----
--C-TC---T-CGAGTCAGT--------AAATC-T-ATACAACTTTCAGCAACGGATCTCTTGGCTCTCCCATCGATGAA
GAACGCAGCGAAATGCGATAAGTAATGTGAATTGCAGAATTCAGTGAATCATCGAATCTTTGAACGCACCTTGCGCCCTT
TGGTATTCCGAAGGGCATGCCTGTTTGAGTGTC-ATTAAATTC-TCAA---------CCTTGCAG-----AC--------
---TTTGT-TTG--------CGAGGC---TTGGAT-GTGA-GG---G--TTTTGCTGGC-TTCC-----TTTGTT-GGAT
GGTCTGCTCCCTTTAAATTCATTAGTGGGA-TCCT--TTGTGGA-----------TGG-TCGCTTGG-TGTGATAA-TTA
TCTACGCCGC-TTGACTCTGACGCAAGACTTGTGGG-AA-CCTGCTTATAACCG------TCTC-TTCG--GAGA-----
---CTAT-CTT------TTGACAATTTGACCT-GCTTTCACTCTCGGTGTCCGCCAGCTCATCGTCGCGGTCAACAAGAT
GGACACGACCAAGGTTTG---GTGCACTATAA-GCG----------------CTAACGAATAAT------TTCGAT--GA
CACCTATTCACAGTGGAGTGAGGACCGTTTCAACGAAATCGTCAAGGAGACGTCCACCTTCATTAAGAAGGTCGGCTACA
ACCCCAAAAATATTTGTTTTGTCCCTATCTCTGGCTGGCACGG-TGACAACAT--GCTTGAGGAGTCCACGAAGTATGGA
G-----ATTTGTGTTTCTGCCGAATCCGACT-TATG-TTTGCG----------------CAGCATGCCCTGG-TTCAAGG
GCTGGACCAAGGAGAC---CAAAGGTGGT--GTCGTCAAGGGCAAGACTCTTCTT---GAAGCTATCG------------
-ATGCTATTGAGCCCCCGGTCCGGCCTGTGGAC-

Mycena_subulata_FFAAS0419

ACTTGGTG-CTGA-GCTGGCC-CTA-----CTC--GGGCA-TGTGCTCGTACCAT--ATTA-TTT-A-TCT---ATCTCT
-TGTGCACCTTTTG-TAGTCTTTGAAGCG---
----TTCGCAGTC----------------------AATGCGG---------TTT--------------TGGGT---CTTG
GGC-----------TTTGCT-GCCCTTTTCCCTG--
---------------TTTG------CTTCA---------AAG-----------------------GCTATG-----TTTT
CA----------------------------TACA-CACTA--T-TTGAAGTTAC-AGAATGTC---TCTTATTGA-----

--C-TCT-TT-TGAGTCGGT--------AAATC-A-ATACAACTTTCAGCAACGGATCTCTTGGCTCTCCCATCGATGAA
GAACGCAGCGAAATGCGATAAGTAATGTGAATTGCAGAATTCAGTGAATCATCGAATCTTTGAACGCACCTTGCGCCCTT
TGGTATTCCGAAGGGCATGCCTGTTTGAGTGTC-ATTAAATTC-TCAA---------CCTTGCAA-----GC--------
---TTTGC-TTG--------TGAGGC---TTGGAT-GTGA-GG---G-CTTTTGCTGGC-TTCCTT--TTTTTTT-GGAT
GGTCTGCTCCCTTTAAATTCATTAGTGGGA-TCCT--TTGTGGA-----------TGG-TCACTTGG-TGTGATAA-TTA
TCTACGCCGC-CTGACTCTGACGCAAGACTTGTGGG-AA-CCCGCTTATAACCG------TCTCTTTCA--GAGA-----
---CTAT-CTT------TTGACAATTTGACCT-GCCTTCACTCTCGGTGTCCGCCAGCTTATCGTCGCGGTCAACAAGAT
GGACACGACCAAGG-TCG---GTGCACTATAT-ACGC--------------TCAAACGAATAAT------TTTGAT--GA
CACCTATTCACAGTGGAGCGAGGACCGTTTCAACGAAATTGTCAAGGAGACGTCCACTTTCATCAAGAAGGTTGGCTACA
ACCCCAAAACTATTTCCTTTGTCCCTATCTCTGGCTGGCACGG-TGACAACAT--GCTCGAGGAGTCCACGAAGTATGAA
GTTCAGATTTGTATTTCTGCCGAATCCAACT-TATG-TTTGCG----------------CAGCATGCCCTGG-TTCAAGG
GCTGGACTAAGGAGAC---CAAAGGTGGT--GTCGTCAAGGGCAAGACTCTTCTT---GAAGCTATCG------------
-ATGCTATTGAGCCCCCGGTCCGGCCTGTGGAC-
Mycena_subulata_FFAAS0423
ACTTGGTG-CTGA-GCTGGCC-CTA-----CTC--GGGCA-TGTGCTCGTACCAT--ATTA-TTT-A-TCT---ATCTCT
-TGTGCACCTTTTG-TAGTCTTTGAAGCG--
----TTCGCAGTC----------------------AATGCGG---------TTT--------------TGGGT---CTTG
GGC-----------TTTGCT-GCCCTTTTCCCTG--
---------------TTTG------CTTCA---------AAG-----------------------GCTATG-----TTTT
CA----------------------------TACA-CACTA--T-TTGAAGTTAC-AGAATGTC---TCTTATTGA-----
--C-TCT-TT-TGAGTCGGT--------AAATC-A-ATACAACTTTCAGCAACGGATCTCTTGGCTCTCCCATCGATGAA
GAACGCAGCGAAATGCGATAAGTAATGTGAATTGCAGAATTCAGTGAATCATCGAATCTTTGAACGCACCTTGCGCCCTT
TGGTATTCCGAAGGGCATGCCTGTTTGAGTGTC-ATTAAATTC-TCAA---------CCTTGCAA-----GC--------
---TTTGC-TTG--------TGAGGC---TTGGAT-GTGA-GG---G-CTTTTGCTGGC-TTCCTT--TTTTTTT-GGAT
GGTCTGCTCCCTTTAAATTCATTAGTGGGA-TCCT--TTGTGGA-----------TGG-TCACTTGG-TGTGATAA-TTA
TCTACGCCGC-CTGACTCTGACGCAAGACTTGTGGG-AA-CCCGCTTATAACCG------TCTCTTTCA--GAGA-----
---CTAT-CTT------TTGACAATTTGACCT-GCCTTCACTCTCGGTGTCCGCCAGCTTATCGTCGCGGTCAACAAGAT
GGACACGACCAAGG-TCG---GTGCACTATAT-ACGC--------------TCAAACGAATAAT-------TTGAT--GA
CACCTATTCACAGTGGAGCGAGGACCGTTTCAACGAAATTGTCAAGGAGACGTCCACTTTCATCAAGAAGGTTGGCTACA
ACCCCAAAACTATTTCCTTTGTCCCTATCTCTGGCTGGCACGG-TGACAACAT--GCTCGAGGAGTCCACGAAGTATGAA
GTTCAGATTTGTATTTCTGCCGAATCCAACT-TATG-TTTGCG----------------CAGCATGCCCTGG-TTCAAGG
GCTGGACTAAGGAGAC---CAAAGGTGGT--GTCGTCAAGGGCAAGACTCTTCTT---GAAGCTATCG------------
-ATGCTATTGAGCCCCCGGTCCGGCCTGTGGAC-
Mycena_sophiae_ACP2157

--TCATCGAATCTTTGAACGCACCTTGCGCCCTT
TGGTATTCCGAAGGGCATGCCTGTTTGAGTGTC-ATTAAATTC-TCAA---------TCTCGCAG-----AC--------
--TTTTGTTTTG--------TGAGAC---TTGGAT-GTGA-GG---G--CTTTGCTGGC-TTCCA---TTCAGTT-GGAT

GGTCTGCTCCCTTTAAATTCATTAGTGGGA-TCCT--TTGTGGA-----------TGG-TCACTTGG-TGTGATAA-TTA
TCTACGCCGC-CTGACTCTGAAGCAAGACTTGCGGG-AA-CCTGCTTATAACCG------TCTC-TCGA--GA-------
---CTAT--CT------TTGACCATTTGACCT-GCGTTCACTCTCGGTGTGCGCCAGCTTATCGTTGCGGTCAACAAGAT
GGACACAACCAAGGTTCG---GCGTAATTCAA-GTC----------------CTGACGAATACT------TTTGGT--GA
CACTCGTTGACAGTGGAGCGAGGACCGTTTCAACGAAATCGTCAAGGAGACGTCCACCTTCATTAAGAAGGTCGGCTACA
ACCCCAAAACTATTTCCTTCGTCCCTATCTCTGGCTGGCACGG-TGACAACAT--GCTTGAGGAGTCCACGAAGTC---G
GTTCAGATTTGTATTTCTACTGAATCCAGCT-TATATTTTGTG-----------------CAGCATGCCCTGG-TTCAAGG
GCTGGACCAAGGAGGT---GAAAGGTGGT--GTCGTCAAGGGCAAGACTCTTCTT---GACGCTATCG------------
-ATG------------------------------
Mycena_sophiae_ACP2161
ACTTGGTG-TTGA-GCTGGCC-CTA-----CTCTCGGGCA-TGTGCTCGCATCAT--ATTA-TTT-ATCCT---ATCTCT
-TGTGCACCTTTTG-TAGTCTTTGAAGCG---
---CTTCGCAGTC----------------------AATGCGG---------TTT--------------TGGGT---CTTG
GGC-----------TTTGTT-GCTCTTTTCCCTG--
---------------TTTG------CTTCA---------AAG-----------------------GCTATG-------TT
TA----------------------------TACA-CCCTG--T-TTGAAGTTAC-AGAATGTC---TCTTATTGA-----
--C-TT---T-CGAGTCAGT--------AAATC-T-ATACAACTTTCAGCAACGGATCTCTTGGCTCTCCCATCGATGAA
GAACGCAGCGAAATGCGATAAGTAATGTGAATTGCAGAATTCAGTGAATCATCGAATCTTTGAACGCACCTTGCGCCCTT
TGGTATTCCGAAGGGCATGCCTGTTTGAGTGTC-ATTAAATTC-TCAA---------TCTCGCAG-----AC--------
--TTTTGTTTTG--------TGAGAC---TTGGAT-GTGA-GG---G--CTTTGCTGGC-TTCCA---TTCAGTT-GGAT
GGTCTGCTCCCTTTAAATTCATTAGTGGGA-TCCT--TTGTGGA-----------TGG-TCACTTGG-TGTGATAA-TTA
TCTACGCCGC-CTGACTCTGAAGCAAGACTTGCGGG-AA-CCTGCTTATAACCG------TCTC-TCGA--GA-------
---CTAT--CT------TTGACCATTTGACCT-GCGTTCACTCTCGGTGTGCGCCAGCTTATCGTTGCGGTCAACAAGAT
GGACACAACCAAGGTTCG---GCGTAATTCAAGGTC----------------CTGACGAATACT------TTTGGT--GA
CACTCGTTGACAGTGGAGCGAGGACCGTTTCAACGAAACCGTCAAGGAGACGTCCACCTTCATTAAGAAGGTCGGCTACA
ACCCCAAAACTATTTCCTTCGTCCCTATCTCTGGCTGGCACGG-TGACAACAT--GCTTGAGGAGTCCACGAAGTC---G
GTTCAGATTTGTATTCCTACTGAATCCAGCT-TCTATTTTGTG-----------------CACCATGCCCTGG-TTCAAGG
GCTGGACCAAGGAGGT---GAAACGTGGT--GTCGTCAAGGGCAAGACTCTTCTC---GAAGCCCCCG------------
-ATGCTATCGAGCCCCCGGTCCGGCCTGTGGAC-
Mycena_diosma_CBH400
ACTTGGTG-TTGA-GCTGGCC-CTA------TT-GGGGAA-TGTGCTCGCATCAT--ATTA-TTT-A-CCT---ATCTCT
-TGTGCACCTTTTG-TAGTCTTTGAAGTG---
---CTTCGCAGTCA---------------------AATGCGG---------TTT--------------TTGGG---TCTG
GGT-------------GGCC-TGCCCTTTCCCTG--
---------------TTTG------CTTCA---------AAG-----------------------GCTATG------TTT
TA----------------------------TACA-CACTA--T-TTGAAGTTAC-AGAATGTC---TCTTATTGA-----
--C-TT---T-TGGGTCAGT--------AAATC-T-ATACAACTTTCAGCAACGGATCTCTTGGCTCTCCCATCGATGAA
GAACGCAGCGAAATGCGATAAGTAATGTGAATTGCAGAATTCAGTGAATCATCGAATCTTTGAACGCACCTTGCGCCCTT
TGGTATTCCGAAGGGCATGCCTGTTTGAGTGTC-ATTAAATTCTTCAA----------CCTTGCGA-----GC-TTTT---
--TTTGGCTTTG--------TGAGGC---TTGGAT-GTGA-GG---G-TTTTTGCTGGC-TTCC----TTCAGTT-GGAT
GGTCTGCTCCCTTTAAATTCATTAGTGGGA-TCCT--TTGTGGA-----------TGG-TCACTTGG-TGTGATAA-TTA
TCTACGCCGC-CTGACTCTGATACAAGACTTGTGGG-AA-CCTGCTTATAACCG------TCTC-TTCA--GAGACAATA
-A---------------TTGACTATTTGACCT-GCCTTCACTCTCGGTGTGCGCCAGCTCATCGTTGCGGTCAACAAGAT
GGACACGACCAAGGTTCG---GTGCATTACAT-GGC----------------CTGACGAACCAT------TTTGAC--GA

CACCTGTTCACAGTGGAGTGAGGACCGTTTCAACGAAATCGTGAAGGAGACGTCCGGCTTCATCAAGAAGGTCGGCTACA
ACCCCAAATCTATTTCCTTCGTCCCTATTTCTGGTTGGCACGG-TGACAACAT--GTTGGAGGAGTCCACGAAGTATGAA
GTTCAGATTTGTATTTCTATCGAACCCAACT-TACG-TCTGCG----------------CAGCATGCCGTGG-TTCAAGG
GCTGGGTCAAAGAGAC---CAAAGCCGGT--GTCGTCAAGGGCAAGACTCTTCTT---GAAGCTATCG------------
-ATGCTATTGAGGCCCCTACCCGGCCTACCGAC-
Mycena_diosma_LK1191_2000
ACTTGGTG-TTGA-GCTGGCC-CTA------TT-GGGGAA-TGTGCTCGCATCAT--ATTA-TTT-A-CCT---ATCTCT
-TGTGCACCTTTTG-TAGTCTTTGAAGTG---
---CTTCGCAGTCA---------------------AATGCGG---------TTT--------------TTGGG---TCTG
GGT-------------GGCC-TGCCCTTTCCCTG---
---------------TTTG------CTTCA---------AAG-----------------------GCTATG------TTT
TA----------------------------TACA-CACTA--T-TTGAAGTTAC-AGAATGTC---TCTTATTGA-----
--C-TT---T-TGGGTCAGT--------AAATC-T-ATACAACTTTCAGCAACGGATCTCTTGGCTCTCCCATCGATGAA
GAACGCAGCGAAATGCGATAAGTAATGTGAATTGCAGAATTCAGTGAATCATCGAATCTTTGAACGCACCTTGCGCCCTT
TGGTATTCCGAAGGGCATGCCTGTTTGAGTGTC-ATTAAATTCTTCAA---------CCTTGCGA-----GC-TTTT---
--TTTGGCTTTG--------TGAGGC---TTGGAT-GTGA-GG---G-TTTTTGCTGGC-TTCC----TTCAGTT-GGAT
GGTCTGCTCCCTTTAAATTCATTAGTGGGA-TCCT--TTGTGGA-----------TGG-TCACTTGG-TGTGATAA-TTA
TCTACGCCGC-CTGACTCTGATACAAGACTTGTGGG-AA-CCTGCTTATAACCG------TCTC-TTCA--GAGACAATA
-A---------------TTGACTATTTGACCT-GCCTTCACTCTCGGTGTGCGCCAGCTCATCGTTGCGGTCAACAAGAT
GGACACGACCAAGGTTCG---GTGCATTACAT-GGC----------------CTGACGAACCAT------TTTGAC--GA
CACCTGTTCACAGTGGAGTGAGGACCGTTTCAACGAAATCGTGAAGGAGACGTCCGGCTTCATCAAGAAGGTCGGCTACA
ACCCCAAATCTATTTCCTTCGTCCCTATTTCTGGTTGGCACGG-TGACAACAT--GTTGGAGGAGTCCACGAAGTATGAA
GTTCAGATTTGTATTTCTATCGAACCCAACT-TACG-TCTGCG----------------CAGCATGCCGTGG-TTCAAGG
GCTGGGTCAAAGAGAC---CAAAGCCGGT--GTCGTCAAGGGCAAGACTCTTCTT---GAAGCTATCG------------
-ATGCTATTGAGGCCCCTACCCGGCCTACCGAC-
Mycena_brunneoviolacea_BAP594
ACTTGGTG-TTGA-GCTGGCT-CTA------TT-GGGGCA-TGTGCTTGCATCAT--ATTA-TTT-A-TCT---ATCTCT
-TGTGCACCTTTTG-TAGTCTTTGAAGCG---
----CTCGCAGTC----------------------AATGCGG----------TC--------------TGGGG---TTTG
GGC-----------TTTGTT-GCCCTTT-CCCTG--
---------------TTTG------CTTCA---------AAG-----------------------GCTATG-----TTTT
TA----------------------------TACA-CGCTA--T-TACAAGTTAC-AGAATGTC---TCTTATTGA-----
--C-TT---T-CGAGTCAGT--------AAATC-T-ATACAACTTTCAGCAACGGATCTCTTGGCTCTCCCATCGATGAA
GAACGCAGCGAAATGCGATAAGTAATGTGAATTGCAGAATTCAGTGAATCATCGAATCTTTGAACGCACCTTGCGCCCTT
TGGTATTCCGAAGGGCATGCCTGTTTGAGTGTC-ATTAAATTC-TCAA---------CCTTACAA-----AG--------
---CTTGCTTTG--------TAAGGC---TTGGAT-GTGA-GG---G-TCTTTGCTGGC-TTCC----TTCAGTTTGGAT
GGTCTGCTCCCTTTAAATTCATTAGTGGGA-TCCT--TTGTGGA-----------TGG-TCACTTGG-TGTGATAA-TTA
TCTACGCCGC-CTGACTCTGAAACAAGACTTGTGGG-AA-CCTGCTCATAACCG------TCTCTTTCA--GAGACAAT-
--CTAAC----------TTGACAATTTGACCT--

Mycena_luceata_ACP2116
ACTTGGTG-TTGA-GCTGGCC-CTG------TT--GGGCATTGTGCTCGCACTAT--ATTA-TTT-A-TCT---ATCTCT
-TGTGCACCTTTTG-TAGTCTTTGAAGTG---
----CTCGCAGTC----------------------AATGCGG---------TTT--------------TGGGA---CTTG
GGC-----------CTTGTT-GCTCTTTTCCTTGC--
---------------TTCG------CTTCA---------AAG-----------------------TCTATG-------TT
TA----------------------------TACA-CACTA----TTAGAGTTAC-AGAATGTC---TCTTATTGA-----
--C-TTT-CT-CAAGTCAGT--------AAAATCT-ATACAACTTTCAGCAACGGATCTCTTGGCTCTCCCATCGATGAA
GAACGCAGCGAAATGCGATAAGTAATGTGAATTGCAGAATTCAGTGAATCATCGAATCTTTGAACGCACCTTGCGCCCTT
TGGTATTCCGAAGGGCATGCCTGTTTGAGTGTC-ATTAAATTC-TCAA---------CCTTGTGG-----GC-TTTT---
----TTGC-TTG--------CAAGGC---TTGGAT-GTGA-GG---GCTTCTTGCTGGC-TTCCC---TTTATCTGGGAT
GGTCTGCTCCCTTCAAATTCATTAGTGGGA-TCCT--TTGTGGA------------------------------------
----
---------------------------------GCCTTCACTCTCGGTGTGCGCCAGCTTATCGTTGCGGTCAACAAGAT
GGACACGACCAAGGTTCG---GTGCATTATAA-GCT----------------CCAATGAATAAG------TTTGAT--GA
CATGTATTCACAGTGGAGTGAGGACCGTTTCAACGAAATCGTCAAGGAGACGTCCACTTTCATTAAGAAGGTCGGCTACA
ACCCCAAATGTATTTCCTTCGTCCCGATTTCTGGCTGGCACGG-TGACAACAT--GCTTGAGGAGTCCACAAAGTATGAA
GTTCAGACTTGTATATCAGCCGAACCCAACT-TATA-TTTATG----------------CAGCATGCCCTGG-TTCAAGG
GCTGGACCAAGGAGAC---CAAAGGTGGT--GTCGTCAAGGGCAAGACCCTTCTT---GAAGCTATCG------------
-ATG------------------------------

Mycena_luceata_ACP2126
ACTTGGTG-TTGA-GCTGGCC-CTG------TT--GGGCATTGTGCTCGCACTAT--ATTA-TTT-A-TCT---ATCTCT
-TGTGCACCTTTTG-TAGTCTTTGAAGTG---
----CTCGCAGTC----------------------AATGCGG---------TTT--------------TGGGA---CTTG
GGC-----------CTTGTT-GCTCTTTTCCTTGC--
---------------TTCG------CTTCA---------AAG-----------------------TCTATG-------TT
TA----------------------------TACA-CACTA----TTAGAGTTAC-AGAATGTC---TCTTATTGA-----
--C-TTT-CT-CAAGTCAGT--------AAAATCT-ATACAACTTTCAGCAACGGATCTCTTGGCTCTCCCATCGATGAA
GAACGCAGCGAAATGCGATAAGTAATGTGAATTGCAGAATTCAGTGAATCATCGAATCTTTGAACGCACCTTGCGCCCTT
TGGTATTCCGAAGGGCATGCCTGTTTGAGTGTC-ATTAAATTC-TCAA---------CCTTGTGG-----GC-TTTT---
----TTGC-TTG--------CAAGGC---TTGGAT-GTGA-GG---GCTTCTTGCTGGC-TTCCC---TTTATCTGGGAT
GGTCTGCTCCCTTCAAATTCATTAGTGGGA-TCCT--TTGTGGA-----------TGGTTCACTTGG-TGTGATAA-TTA
TCTACGCCGC-TTGACTCTGAAGCAAGACTTGTGGG-GA-CCCGCTCATAACCG------TCTC-TCCG--GAGA-----
---CTAT--CT------TTGACCATTTGACCT-GCCTTCACTCTCGGTGTGCGCCAGCTTATCGTTGCGGTCAACAAGAT
GGACACGACCAAGGTTCG---GTGCATTATAA-GCT----------------CCAATGAATAAG------TTTGAT--GA
CATGTATTCACAGTGGAGTGAGGACCGTTTCAACGAAATCGTCAAGGAGACGTCCACTTTCATTAAGAAGGTCGGCTACA
ACCCCAAATGTATTTCCTTCGTCCCGATTTCTGGCTGGCACGG-TGACAACAT--GCTTGAGGAGTCCACAAAGTATGAA
GTTCAGACTTGTATATCAGCCGAACCCAACT-TATA-TTTATG----------------CAGCATGCCCTGG-TTCAAGG
GCTGGACCAAGGAGGC---CAAAGGTGGT--GTCGTCAAGGGCAAGACCC-------------------------------
----

Mycena_cahaya_ACL134
ACTTGGTG-TTGA-GCTGGCT--------------ATGCA-AGTGCTTGCATCAT--ATTA-TTT-A-TCT---ATCTCT
-TGTGCACCTTTTG-TAGTCTTTGAAAGT---

---GTTCGCAGTCA---------------------AATGCGGT-------TTTT--------------TGGGC---CTTG
GGC-----------TTTGCT-GTCCTTTGCCCTG--
---------------TTTG------CTTTG---------AAG-----------------------GCTATG------TTT
TA----------------------------TACA-CACTG--TTTTGAAGTCGT-AGAATGTC---TGTTATTGA-----
--C-TT---T-TGAGTCAGT--------AAATC-T-ATACAACTTTCAGCAACGGATCTCTTGGCTCTCCCATCGATGAA
GAACGCAGCGAAATGCGATAAGTAATGTGAATTGCAGAATTCAGTGAATCATCGAATCTTTGAACGCACCTTGCGCCCTT
TGGTATTCCGAAGGGCATGCCTGTTTGAGTGTC-ATTAAATTC-TCAA---------TCTTGCAG-----AC--------
---TTTGT-CTG--------TGAGGC---TTGGAT-GTGA-GG---G--CTTTGCTGGC-TTTCT-----------TTAA
AGTCTGCTCCCTTTAAATTCATTAGTGGGATTCCT--TTGTGGA-----------TGG-TCACTTGG-TGTGATAA-TTA
TCTACGCCGT-CTGACTCTGAAACAAGACTTGTGGGAAA-TCTGCTTATAACCACCA---TCTT----------------
--------TTT------TTGACAATTTGACCT--

Mycena_amygdalina_MT497544
GATCGGTT-CTGATGCTGGCC-CTT-----CAC-CGGGCA-TGTGCTCGCTCCGA--ATCTATTT-A-TCT----TCTCT
-TGTGCACCTCTTG-TAGTCTTTGAAAAACTCGA--
-ACCTCTCCCATC----------------------AATGCGG---------ACT--------------GGGGG---GCTG
GGT----------CCCTTCC-CCCCCTTCCCCTGCTT---
--------------GCTTT------CTTTC---------ACG-----------------------GCTATG-----TTTT
CA----------------------------TATA-CACTA--T---AAAGTTAC-ATAATGTC---TTTTAAAGAT-TGT
CGC-TCGCGC-GCAATCTTT--------AAACC-T-ATACAACTTTCACTCTCCCATCTCTTGGCTCTCCCATCAATGAA
GAACGCAACTGAATGCTATAAATAATGTGAATTGCACAATTCTTTGAATCATCCAATCTTTGAACGCACCTTGCACCCTT
TGGTATTCCGAAGGGCATGCCTGTTTGAGTGTC-ATTAAATTC-TCAA---------CCTTGCTC-----GC--------
---TTTGC-GAG--------TGGGGC---TTGGAT-GTGA-GG---G---CTTGCTGGG-TTCC----TTCCCT--GGAT
GGTCTGCTCCCTTTAAATGCATTAGTGGGA-TCTC--TTGTGGA-----------CCG-TTACTTGG-TGTGATAA-TTA
TCTATGCCTCGTCTACTTTGAAGC-AAAATTATGGG-AA-CCTGCTTATAACCG------TCTCGCAAG---GGACAATA
------T--CT------CTGACATTTTGACCT--

Mycena_amygdalina_HMJAU43629
GATCGGTT-CTGATGCTGGCC-CTT-----CAC-CGGGCA-TGTGCTCGCTCCGA--ATCTATTT-A-TCT----TCTCT
-TGTGCACCTCTTG-TAGTCTTTGAAAAACTCGA--
-ACCTCTCCCATC----------------------AATGCGG---------ACT--------------GGGGG---GCTG
GGT----------CCCTTCC-CCCCCTTCCCCTGCTT---
--------------GCTTT------CTTTC---------ACG-----------------------GCTATG-----TTTT
CA----------------------------TATA-CACTA--T---AAAGTTAC-ATAATGTC---TTTTAAAGAT-TGT

CGC-TCGCGC-GCAATCTTT--------AAACC-T-ATACAACTTTCACTCTCCCATCTCTTGGCTCTCCCATCAATGAA
GAACGCAACTGAATGCTATAAATAATGTGAATTGCACAATTCTTTGAATCATCCAATCTTTGAACGCACCTTGCACCCTT
TGGTATTCCGAAGGGCATGCCTGTTTGAGTGTC-ATTAAATTC-TCAA---------CCTTGCTC-----GC--------
---TTTGC-GAG--------TGGGGC---TTGGAT-GTGA-GG---G---CTTGCTGGG-TTCC----TTCCCT--GGAT
GGTCTGCTCCCTTTAAATGCATTAGTGGGA-TCTC--TTGTGGA-----------CCG-TTACTTGG-TGTGATAA-TTA
TCTATGCCTCGTCTACTTTGAAGC-AAAATTATGGG-AA-CCTGCTTATAACCG------TCTCGCAAG---GGACAATA
------T--CT------CTGACATTTTGACCT--

Mycena_zephirus_KR673722
ATTG-GTA-CTGATGCTGGCT-CTT-----CAC-TGAGCA-TGTGCTCGT--TCC--ATCTATTT-A-TCT----TCTCT
-TGTGCACATTTTG-TAGTCTTGAATT---------------------------------GA----AACC----------
---TCTCGCAGTC----------------------AATGCGGT---------TT--------------GGGAG---ATT-
---GTTAACCC---------------TTCTCCTG-CT---
------------TC------------TTCA----------AG-----------------------GCTATG-----TTTT
CA----------------------------TATA-CACTA----T-ACAGTTAC-AGAATGTC---TTTTAACGA-CTTG
CGC--TTGTC-GCAGTCATT--------AAACC-T-ATACAACTTTCAGCAACGGATCTCTTGGCTCTCCTATCGATGAA
GAACGCAGCGAAATGCGATAAGTAATGTGAATTGCAGAATTCAGTGAATCATCGAATCTTTGAACGCACCTTGCGCCCTT
TGGTATTCCGAAGGGCATGCCTGTTTGAGTGTC-ATTAAATTA-TCAA---------CCTTGCTC-----GC-TTTT---
--ACCGGC-TTGAG------TGAGGT---TTGGAC-GTGA-GG---G--CTTTGCTGGC-TTCC----TTCAGT--GGAC
GGTCTGCTCCCTTTAAATGCATTAGTGGGA-TCTC--TTGTGGA-----------CCG-TCACTTGG-TGTGATAA-TTA
TCTATGCCAT-TTGACTTTGAAGC-AAACTTATGGG-AA-CCTGCTTATAACCG------TCTCT---------------
----

Mycena_zephirus_HMJAU43106
ATTG-GTA-CTGATGCTGGCT-CTT-----CAT-TGAGCA-TGTGCTCGT--TCC--ATCTATTT-A-TCT----TCTCT
-TGTGCACATTTTG-TAGTCTTGAATT---------------------------------GA----AACC----------
---TCTCGCAGTC----------------------AATGCGGT---------TT--------------GGGAG---ATT-
---GTTAACCC---------------TTCTCCTG-CT---
------------TC------------TTCA----------AG-----------------------GCTATG-----TTTT
CA----------------------------TATA-CACTA----T-AAAGTTAC-AGAATGTC---TTTTAACGA-CTTG
CGC--TTGTC-GCAGTCATT--------AAACC-T-ATACAACTTTCAGCAACGGATCTCTTGGCTCTCCTATCGATGAA
GAACGCAGCGAAATGCGATAAGTAATGTGAATTGCAGAATTCAGTGAATCATCGAATCTTTGAACGCACCTTGCGCCCTT
TGGTATTCCGAAGGGCATGCCTGTTTGAGTGTC-ATTAAATTA-TCAA---------CCTTGCTC-----GC-TTTT---
--ACCGGC-TTGAG------TGAGGT---TTGGAC-GTGA-GG---G--CTTTGCTGGC-TTCC----TTCAGT--GGAC

GGTCTGCTCCCTTTAAATGCATTAGTGGGA-TCTC--TTGTGGA-----------CCG-TCACTTGG-TGTGATAA-TTA
TCTATGCCAT-TTGACTTTGAAGC-AAACTTATGGG-AA-CCTGCTTATAACCG------TCTCTTCGG---GGACAAT-
---------CT-----ATTGACATTTTGACCT--

Mycena_algeriensis_HKAS_134295
ATTG-GTA-CTGATGCTGGCT-CTT-----CAC-TGAGCA-TGTGCTCGT--TCC--ATCTATTT-A-TCT----TCTCT
-TGTGCACATCTTG-TAGTCTTGAATT--------------------------------GA----AACC----------
---TCTCGCAGTC----------------------AATGCGGT---------TT--------------GGGAG---ATT-
---GTTAACCC---------------TTCTCCTG-CT---
------------TC------------TTCA----------AG-----------------------GCTATG-----TTTT
CA----------------------------TATA-CACTA----T-AAAGTTAC-AGAATGTC---TTTTAACGA-CTTG
TGC--TTGTC-GCAGTCATT--------AAACC-T-ATACAACTTTCAGCAACGGATCTCTTGGCTCTCCTATCGATGAA
GAACGCAGCGAAATGCGATAAGTAATGTGAATTGCAGAATTCAGTGAATCATCGAATCTTTGAACGCACCTTGCGCCCTT
TGGTATTCCGAAGGGCATGCCTGTTTGAGTGTC-ATTAAATTA-TCAA---------CCTTGCTC-----GC--------
---------TTGAG------TGAGGC---TTGGAT-GTGA-GG---G--CTTTGCTGGC-TTCC----TTCAGT--GGAC
GGTCTGCTCCCTTTAAATGCATTAGTGGGA-TCTC--TTGTGGA-----------CCG-TCACTTGG-TGTGATAA-TTA
TCTATGCCAT-TTGACTTTGAAGC-AAACTTATGGG-AA-CCTGCTTATAACCG------TCTCTTCGG---GGACAAC-
---------CT------TTGACATTTTGACCT--

Mycena_purpureofusca_oka283
ATTG-GTA-CTGATGCTGGCT-CTT-----CAC-AGAGCA-TGTGCTCG---TCC--ATCTATTT-A-TCT----TCTCT
-TGTGCACATCTTG-TAGTCTTGAA-T---------------------------------GA----AACC----------
---TCTCGCAGTC----------------------AATGCGGT---------TT--------------GGGAG----TT-
---GTTAACCC---------------TTCTCCTG-CT---
------------TC------------TTCA----------AG-----------------------GCTATG-----TTTT
CA----------------------------TATA-CACTA----T-AAAGTTAC-AGAATGTC---TTTTAAAGG-CTTG
CGC--TTGTC-GCAGTCATT--------AAACC-T-ATACAACTTTCAGCAACGGATCTCTTGGCTCTCCTATCGATGAA
GAACGCAGCGAAATGCGATAAGTAATGTGAATTGCAGAATTCAGTGAATCATCGAATCTTTGAACGCACCTTGCGCCCTT
TGGTATTCCGAAGGGCATGCCTGTTTGAGTGTC-ATTAAATTA-TCAA---------CCTTGCTC-----GC-TTTT---
--ACCGGC-TTGAG------TTAGGC---TTGGAT-GTGA-GG---G--C-TTGCTGGC-TTCC----TTCAGT--GGAT
GGTCTGCTCCCTTTAAATGCATTAGTGGGA-TCTC--TTGTGGA-----------CCG-TCACTTGGTTGTGATAA-TTA
TCTACGCCAT-TTGACTTTGAAGC-AAACTTATGGG-AA-CCTGCTTATAACCG------TCCTTTCGA---GGACAAT-
---------CT------TTGACATTTTGACCT--

Mycena_purpureofusca_oka284
ATTG-GTA-CTGATGCTGGCT-CTT-----CAC-AGAGCA-TGTGCTCG---TCC--ATCTATTT-A-TCT----TCTCT
-TGTGCACATCTTG-TAGTCTTGAA-T---------------------------------GA----AACC----------
---TCTCGCAGTC----------------------AATGCGGT---------TT--------------GGGAG----TT-
---GTTAACCC---------------TTCTCCTG-CT---
------------TC------------TTCA----------AG-----------------------GCTATG-----TTTT
CA----------------------------TATA-CACTA----T-AAAGTTAC-AGAATGTC---TTTTAAAGG-CTTG
CGC--TTGTC-GCAGTCATT--------AAACC-T-ATACAACTTTCAGCAACGGATCTCTTGGCTCTCCTATCGATGAA
GAACGCAGCGAAATGCGATAAGTAATGTGAATTGCAGAATTCAGTGAATCATCGAATCTTTGAACGCACCTTGCGCCCTT
TGGTATTCCGAAGGGCATGCCTGTTTGAGTGTC-ATTAAATTA-TCAA---------CCTTGCTC-----GC-TTTT---
--ACCGGC-TTGAG------TTAGGC---TTGGAT-GTGA-GG---G--C-TTGCTGGC-TTCC----TTCAGT--GGAT
GGTCTGCTCCCTTTAAATGCATTAGTGGGA-TCTC--TTGTGGA-----------CCG-TCACTTGGTTGTGATAA-TTA
TCTACGCCAT-TTGACTTTGAAGC-AAACTTATGGG-AA-CCTGCTTATAACCG------TCCTTTCGA---GGACAAT-
---------CT------TTGACATTTTGACCT--

Mycena_haematopus_420526MF0193
ATTG-GGA-CTGATGCTGGCT-CTT-----CAC-TGAGCA-TGTGCTCGT--CTC--ATCTATTT-A-TCT----TCTCT
-TGTGCACATCTTG-TGGTCTTGAATT---------------------------------GA----AACC----------
---TTTCGCATT-----------------------CGTGCGGT---------TT--------------GGGAG---ATT-
---GTTAAACC---------------TTCTCCTG-CT---
------------TC-----------ATTCA----------AG-----------------------GTCATG-----TTTT
CA----------------------------TATA-CACTA----T-AAAGTTAC-AGAATGTC---TTTTAACGA--TTG
CGC--TTGTC-GTAGTCATT--------AAACC-T-ATACAACTTTCAGCAACGGATCTCTTGGCTCTCCTATCGATGAA
GAACGCAGCGAAATGCGATAAGTAATGTGAATTGCAGAATTCAGTGAATCATCGAATCTTTGAACGCACCTTGCGCCCTT
TGGTATTCCGAAGGGCATGCCTGTTTGAGTGTC-ATTAAATTA-TCAA---------CCTTGCTC-----GC-TTTT---
--ACTAGC-TTGAG------TTAGGC---TTGGAT-GTGA-GG---G--C-TTGCTGGC-TTCC----TTCAGT--GGAT
GGTCTGCTCCCTTTAAATACATTAGTGGGA-TCTC--TTGTGGA-----------CCG-TCACTTGG-TGTGATAA-TTA
TCTATGCCTT-GAGACTTTGAAGC-AAACTAATGGG-AA-TCCGCTTATAACCG------TCTTC-------GGACAAT-
---------TA------ATGACTATTTGACCT--

Mycena_haematopus_420526MF0200

ATTG-GGA-CTGATGCTGGCT-CTT-----CAC-TGAGCA-TGTGCTCGT--CTC--ATCTATTT-A-TCT----TCTCT
-TGTGCACATCTTG-TGGTCTTGAATT---------------------------------GA----AACC----------
---TTTCGCATT-----------------------CGTGCGGT---------TT--------------GGGAG---ATT-
---GTTAAACC---------------TTCTCCTG-CT---
------------TC-----------ATTCA----------AG-----------------------GTCATG-----TTTT
CA----------------------------TATA-CACTA----T-AAAGTTAC-AGAATGTC---TTTTAACGA--TTG
CGC--TTGTC-GTAGTCATT--------AAACC-T-ATACAACTTTCAGCAACGGATCTCTTGGCTCTCCTATCGATGAA
GAACGCAGCGAAATGCGATAAGTAATGTGAATTGCAGAATTCAGTGAATCATCGAATCTTTGAACGCACCTTGCGCCCTT
TGGTATTCCGAAGGGCATGCCTGTTTGAGTGTC-ATTAAATTA-TCAA---------CCTTGCTC-----GC-TTTT---
--ACTAGC-TTGAG------TTAGGC---TTGGAT-GTGA-GG---G--C-TTGCTGGC-TTCC----TTCAGT--GGAT
GGTCTGCTCCCTTTAAATACATTAGTGGGA-TCTC--TTGTGGA-----------CCG-TCACTTGG-TGTGATAA-TTA
TCTATGCCTT-GAGACTTTGAAGC-AAACTAATGGG-AA-TCCGCTTATAACCG------TCTTC-------GGACAAT-
---------TA------ATGACTATTTGACCT--

Mycena_alnetorum_AH57250

ATTG-GTA-CTGATGCTGGCT-CTT-----AAC-TGAGCA-TGTGCTCG---TCC--ATCTATTT-A-TCT----TCTCT
-TGTGCACATTTTG-TAGTCTTGAACG---------------------------------------TACC----------
---TCTCGCAGTCA---------------------AATGCGGT---------TT--------------GGGAG---CTTG
GGCGCAAGCCC---------------TTCTCCTG-CT---
------------------------TCTTCA----------AG-----------------------GCTATG-----TTTT
CA----------------------------TATA-CACTA----TTAAAGTTCCAAGAATGTC---TTTTAACGA--TTG
CGC--TTGTC-GTAGTCATT--------AAACC-T-ATACAACTTTCAGCAACGGATCTCTTGGCTCTCCTATCGATGAA
GAACGCAGCGAAATGCGATAAGTAATGTGAATTGCAGAATTCAGTGAATCATCGAATCTTTGAACGCACCTTGCGCCCTT
TGGTATTCCGAAGGGCATGCCTGTTTGAGTGTC-ATTAAATTA-TCAA---------CCTTGCTC-----GC-TTTT---
--ACCGGC-TTGAG------TTAGGC---TTGGAT-GTGA-GG---G--C-TTGCTGGC-TTCC----TTCAGT--GGAC
GGTCTGCTCCCTTCAAATGCATTAGTGGGA-TCTC--TTGTGGA-----------CCG-TCACTTGG-TGTGATAA-TTA
TCTATGCCAT-TTGACTTTGAAGCAAAACTTATGGG-AA-CCCGCTCATAACCG------TCTTC-------GGACAAC-
---------TT------TTGACATTTCGA---

Mycena_polygramma_H6039058

ATTG-GAA-CTGATGCTGGCT-TCT-----TAC-GGAGCA-TGTGCTCG---TCC--ATCTATTTAA-CCT----TCTCT
-TGTGCACATTTTG-TAGTCTTGAATC---------------------------------AAAGTGAACC----------

---CTTCGCAGC-----------------------AATGCGGT---------TT--------------GGGGG---AATG
GGCGCAAGCCC---------------TTCTCCTG-CT---
------------TGCTTTC-----TTTTCA----------AG-----------------------GCTATG-----TTTT
CA----------------------------TATA-CACTA----T-AAAGTTTC-AGAATGTC---TTTTAACGA--TTG
CGC--TTGTC-GTAGTCATT--------AAACC-T-ATACAACTTTCAGCAACGGATCTCTTGGCTCTCCCATCGATGAA
GAACGCAGCGAAATGCGATAAGTAATGTGAATTGCAGAATTCAGTGAATCATCGAATCTTTGAACGCACCTTGCGCCCTT
TGGTATTCCGAAGGGCATGCCTGTTTGAGTGTC-ATTAAATTC-TCAA---------CCTCGTTC-----GC-TTTT---
--ACTAGC-TTGAG------CGAGGC---TTGGAC-GTGA-GG---G--C-TTGCTGGC-TTCC----TTCAGT--GGAT
GGTCTGCTCCCTTTAAAAGCATTAGTGGGA-TCTC--TTGTGGA-----------CCG-TCACTTGG-TGTGATAA-TTA
TCTATGCCAG-TTGACTTTGAAGC-AAACTTATGGG-AA-CCTGCTTATAACCG------TCTCGCAAG---GGACAAC-
---------AATCTTAATTGACA-TTTGACCT--

Mycena_polygramma_H6039079
-------------------CT-TCT-----TAC-GGGGCA-TGTGCTCG---TCC--ATCTATTTAA-CCT----TCTCT
-TGTGCACATTTTG-TAGTCTTGAATT---------------------------------AAAGTGAACC----------
---CTTCGCAGC-----------------------AATGCGGT---------TT--------------GGGGG---AATG
GGCGCAAGCCC---------------TTCTCCTG-CT---
------------TGCTTTC-----TTTTCA----------AG-----------------------GCTATG-----TTTT
CA----------------------------TATA-CACTA----TNAAAGTTTC-AGAATGTC---TTTTAACGA--TTG
CGC--TTGTC-GTAGTCATT--------AAACC-T-ATACAACTTTCAGCAACGGATCTCTTGGCTCTCCCATCGATGAA
GAACGCAGCGAAATGCGATAAGTAATGTGAATTGCAGAATTCAGTGAATCATCGAATCTTTGAACGCACCTTGCGCCCTT
TGGTATTCCGAAGGGCATGCCTGTTTGAGTGTC-ATTAAATTC-TCAA---------CCTCGTTC-----GC-TTTT---
--ACTAGC-TTGAG------CGAGGC---TTGGAC-GTGA-GG---G--C-TTGCTGGC-TTCC----TTCAGT--GGAT
GGTCTGCTCCCTTTAAAAGCATTAGTGGGA-TCTC--TTGTGGA-----------CCG-TCACTTGG-TGTGATAA-TTA
TCTATGCCAG-TTGACCTTGAAGC-AAACTTATGGG-AA-CCTGCTTATAACCG------TCTCGCAAG---GGAC----
----

FLF1971
TTGG--TA-CTGATGCTGGCT-CTT-----CGC-TGAGCA-TGTGCTCGT---CC--ATCTATTT-A-TCT----TCTCT
-TGTGCACCTTTTG-TAGTCTTCGAAG----------------------------------AAGCTAACC----------
---TCTCGCAGC-----------------------AATGCGGT---------TT--------------GGGAG--GCATG
GGCGTCAGCCC---------------TTCTCCTG-CT---
------------TGCTTTC-------TTCG----------AG-----------------------GCTATG-----TTTT
CA----------------------------TATAACACTA----TAAAAGTTAC-AGAATGTC---TTTTAAAGA--TTG

CGC--TAGCC-GCAGTCATT--------AAACC-T-ATACAACTTTCAGCAACGGATCTCTTGGCTCTCCCATCGATGAA
GAACGCAGCGAAATGCGATAAGTAATGTGAATTGCAGAATTCAGTGAATCATCGAATCTTTGAACGCACCTTGCGCCCTT
TGGTATTCCGAAGGGCATGCCTGTTTGAGTGTC-ATTAAATTTATCAA---------CCTTGCTT-----GC-TTTT---
--ACTAG--CTGAG------TTAGGC---TTGGAT-GTGG-GGTTTT--TTTTGCTGGC-TTCC----TTCAGT--GGAT
GGTCTGCTCCCCTTAAATGCATTAGTGGGA-TCTC--TTGTGGA-----------CCG-TCACTTGG-TGTGATAA-TTA
TCTATACCAC-TTGGCCTCGAAGCAAATCTTATGGG-AA-CCCGCTCATAACCG------TCTGCTCGT--CGGACAAC-
---------CT------TTGACATTTTGACCT-GCCTTCACCCTCGGTGTGCGTCAGCTCATCGTTGCCGTCAACAAGAT
GGACACGACCAAGGTCGG----TTTTTTTTTC-CCCCCCAATATTTTTCCCAAAATTGAACCCCCCCCCTTTTTCT----
-----------AAGGGAAGGAGGACCGTTTCAACGAAATTATCAAGGAAAAGGCCCCCTTCATCAAAAAGGGTGGGTACA
ACCCCAAAACTATTGGCTTCCTCCCCATCTCGGGCTGGACGG-GGACAAAT--GGTGGAGGAAGCCAATAAATATGGG
TTTTTTGGTTGGTCCCCCGGTAT--------------TTTACTGGAC------ATTGGACCGGATGGCCTGG-TTTAAGG
GTTGGAACAAAGAGAA---CAAGGGCGGT--GGCCTCCAGGGCAAAACCTTCTC-----AAGGTATG-------------
-AAGCCATCGAACCCCGGGCGTTCTTCAGACCA-
MSX1971
TTGG--TA-CTGATGCTGGCT-CTT-----CGC-TGAGCA-TGTGCTCGT---CC--ATCTATTT-A-TCT----TCTCT
-TGTGCACCTTTTG-TAGTCTTCGAAG----------------------------------AAGCTAACC----------
---TCTCGCAGC-----------------------AATGCGGT---------TT--------------GGGAG--GCATG
GGCGTCAGCCC---------------TTCTCCTG-CT---
------------TGCTTTC-------TTCG----------AG-----------------------GCTATG-----TTTT
CA----------------------------TATAACACTA----TAAAAGTTAC-AGAATGTC---TTTTAAAGA--TTG
CGC--TAGCC-GCAGTCATT--------AAACC-T-ATACAACTTTCAGCAACGGATCTCTTGGCTCTCCCATCGATGAA
GAACGCAGCGAAATGCGATAAGTAATGTGAATTGCAGAATTCAGTGAATCATCGAATCTTTGAACGCACCTTGCGCCCTT
TGGTATTCCGAAGGGCATGCCTGTTTGAGTGTC-ATTAAATTTATCAA---------CCTTGCTT-----GC-TTTT---
--ACTAG--CTGAG------TTAGGC---TTGGAT-GTGG-GGTTTT--TTTTGCTGGC-TTCC----TTCAGT--GGAT
GGTCTGCTCCCCTTAAATGCATTAGTGGGA-TCTC--TTGTGGA-----------CCG-TCACTTGG-TGTGATAA-TTA
TCTATACCAC-TTGGCCTCGAAGCAAATCTTATGGG-AA-CCCGCTCATAACCG------TCTGCTCGT--CGGACAAC-
---------CT------TTGACATTTTGACCT-GCCTTCACCCTCGGTGTGCGTCAGCTCATCGTTGCCGTCAACAAGAT
GGACACGACCAAGGTCGG----TTTTTTTTTC-CCCCCCAATATTTTTCCCAAAATTGAACCCCCCCCCTTTTTCT----
-----------AAGGGAAGGAGGACCGTTTCAACGAAATTATCAAGGAAAAGGCCCCCTTCATCAAAAAGGGTGGGTACA
ACCCCAAAACTATTGGCTTCCTCCCCATCTCGGGCTGGACGG-GGACAAAT--GGTGGAGGAAGCCAATAAATATGGG
TTTTTTGGTTGGTCCCCCGGTAT--------------TTTACTGGAC------ATTGGACCGGATGGCCTGG-TTTAAGG
GTTGGAACAAAGAGAA---CAAGGGCGGT--GGCCTCCAGGGCAAAACCTTCTC-----AAGGTATG-------------
-AAGCCATCGAACCCCGGGCGTTCTTCAGACCA-
Mycena_strobilinoidea_NX0647
TTTG-GTT-CTGATGCTGGCA-TCCGGTAACAC-GGTGCA-AGTGCTCGT--CCA--TCCTATTT-A-TCT----TCTCT
-TGTGCACCTCTTG-TAGTCTTTGAAAG--------------------------------GAAGTGAACC----------
---TCTCGCAGT-----------------------GATGCGGT---------TA--------------GGGGG---AATG
GGCGCGAGTCC---------------TTCTCCTG-CT---
------------TGCTTTT-----CGTTCA---------AAG-----------------------GCTATG-----TTTT
CA----------------------------TATA-CACTA----T-AAAGTTAC-AGAATGTC---TTTTAAAGA--TTG
TGC--TAGTC-GCAGTCATT--------AAACC-T-ATACAACTTTCAGCAACGGATCTCTTGGCTCTCCCATCGATGAA
GAACGCAGCGAAATGCGATAAGTAATGTGAATTGCAGAATTCAGTGAATCATCGAATCTTTGAACGCACCTTGCGCCCTT
TGGTATTCCGAAGGGCATGCCTGTTTGAGTGTC-ATTAAATTA-TCAA---------CCTTGCTC-----GC-TTTT---
--ACCGGT-TTGAG------TTAGGC---TTGGAT-GTGG-GG---G-TTTTTGCTGGC-TTCC----TTCAGT--GGAT

GGTCTGCTCCCCTTAAATGCATTAGCGGGA-TCTC--TTGTGGA-----------CCG-TCACTTGG-TGTGATAA-TTA
TCTATGCCACGTTGACTTTGAAGCAAAACTTATGGG-AA-TCCGCTCATAACCG------TCCACTCGT---GGACAAC-
---------TC------TTGACATTTTGACCT--

Mycena_strobilinoidea_NX0648
TTTG-GTT-CTGATGCTGGCA-TCCGGTAACAC-GGTGCA-AGTGCTCGT--CCA--TCCTATTT-A-TCT----TCTCT
-TGTGCACCTCTTG-TAGTCTTTGAAAG--------------------------------GAAGTGAACC----------
---TCTCGCAGT-----------------------GATGCGGT---------TA--------------GGGGG---AATG
GGCGCGAGTCC---------------TTCTCCTG-CT---
------------TGCTTTT-----CGTTCA---------AAG-----------------------GCTATG-----TTTT
CA----------------------------TATA-CACTA----T-AAAGTTAC-AGAATGTC---TTTTAAAGA--TTG
TGC--TAGTC-GCAGTCATT--------AAACC-T-ATACAACTTTCAGCAACGGATCTCTTGGCTCTCCCATCGATGAA
GAACGCAGCGAAATGCGATAAGTAATGTGAATTGCAGAATTCAGTGAATCATCGAATCTTTGAACGCACCTTGCGCCCTT
TGGTATTCCGAAGGGCATGCCTGTTTGAGTGTC-ATTAAATTA-TCAA---------CCTTGCTC-----GC-TTTT---
--ACCGGT-TTGAG------TTAGGC---TTGGAT-GTGG-GG---G-TTTTTGCTGGC-TTCC----TTCAGT--GGAT
GGTCTGCTCCCCTTAAATGCATTAGCGGGA-TCTC--TTGTGGA-----------CCG-TCACTTGG-TGTGATAA-TTA
TCTATGCCACGTTGACTTTGAAGCAAAACTTATGGG-AA-TCCGCTCATAACCG------TCCACTCGT---GGACAAC-
---------TC------TTGACATTTTGACCT--

Mycena_flos_nivium_CBS_364_50

-------------------------------------TGCGGT---------TT--------------GGGGG---AATG
GGTGCAAGCCC---------------TTCTCCTGCCT---
------------TGCTTTC-------TTCA----------AG-----------------------GCTATG-----TTTT
CA----------------------------TATAACACTA----T-AAAGTTAC-AGAATGTC---TTTTTAGGA--TTG
TGC--TAGTC-GCAGTCATT--------AAACC-T-ATACAACTTTCAGCAACGGATCTCTTGGCTCTCCCATCGATGAA
GAACGCAGCGAAATGCGATAAGTAATGTGAATTGCAGAATTCAGTGAATCATCGAATCTTTGAACGCACCTTGCGCCCTT
TGGTATTCCGAAGGGCATGCCTGTTTGAGTGTC-ATTAAATTC-TCAA---------CCTTGCTT-----GC-TTTT---
--ACTAG--CTGAG------CTTGGC---TTGGAC-GTGG-GG---G--TTTTGCTGGC-TTCC----TTCAGT--GGAT
GGTCTGCTCCCCTTAAATGCATTAGTGGGA-TCTC--TTGTGGA-----------CCG-TCACTTGG-TGTGATAA-TTA
TCTATGCCACGTTGACTTTGAAGCAAAACTTATGGG-AA-CCCGCTCATAACCG------TCTGCTCGT--CAGACAAC-
------T--TT------TTGACATTTTGACCT--

Mycena_flos_nivium_CBS_260_53
TTGGTTCA-CTGATGCTGGCT-CTT-----TAC-TGAGTAATGTGCTCGT--GCC--ATCTATTT-A-TCT----TCTCT
-TGTGCACCTTTTG-TAGTCTTTGAAG---------------------------------AAAGCGAACC----------
--TCTTCGCAGC-----------------------AATGCGGT---------TT--------------GGGGG---AATG
GGCGCAAGCCC---------------TTCTCCTGCCT---
------------TGCTTTC-------TTCA----------AG-----------------------GCTATG-----TTTT
CA----------------------------TATAACACTA----T-AAAGTTAC-AGAATGTC---TTTTAAAGA--TTG
TGC--TAGTC-GCAGTCATT--------AAACC-T-ATACAACTTTCAGCAACGGATCTCTTGGCTCTCCCATCGATGAA
GAACGCAGCGAAATGCGATAAGTAATGTGAATTGCAGAATTCAGTGAATCATCGAATCTTTGAACGCACCTTGCGCCCTT
TGGTATTCCGAAGGGCATGCCTGTTTGAGTGTC-ATTAAATTC-TCAA---------CCTTGCTC-----GC-TTTT---
--ACTAG--CTGAG------CTTGGC---TTGGAT-GTGG-GG---G--TTTTGCTGGC-TTCC----TTCAGT--GGAT
GGTCTGCTCCCCTTAAATGCATTAGTGGGA-TCTC--TTGTGGA-----------CCG-TCACTTGG-TGTGATAA-TTA
TCTATGCCACGTTGACTTTGAAGCAAAACTTATGGG-AA-CCCGCTCATAACCG------TCTGCTCGT--CAGACAAC-
------T--TT------TTGACATTTTGACCT--

Mycena_semivestipes_HMJAU43825
TTGG--TA-CTGATGCTGGCT-CTT-----TAC-TGAGCA-TGTGCTCGT---CC--ATCTATTT-A-TCT----TCTCT
CTGTGCACCTTTTG-TAGTCTTTGAAG---
-------------------------------------CAAGGT---------TT--------------GGAGGTTCTGTG
GGTGAAAGCCC---------------TCCCTCTG-CTGTT--
-----------ATGCTTCT-------TCAA----------GG-----------------------ACTATG-----TTTT
CA----------------------------TATA-CACTA----TTAAAGTTAC-AGAATGTC---TTTTAACGA--TTG
TGC--TAGTC-GCAGTCATT--------AAACC-T-ATACAACTTTCAGCAACGGATCTCTTGGCTCTCCCATCGATGAA
GAACGCAGCGAAATGCGATAAGTAATGTGAATTGCAGAATTCAGTGAATCATCGAATCTTTGAACGCACCTTGCGCCCTT
TGGTATTCCGAAGGGCATGCCTGTTTGAGTGTC-ATTAAATTC-TCAA---------CCTTGCTT-----GC-TTTT---
--ACTAG--CTGAG------CTCGGC---TTGGAC-GTGG-GGT--T--TTTTGCTGGC-TTCC----TTCAGT--GGAT
GGTCTGCTACCCTTAAATGCATTAGTGGGA-TCTC--TTGTGGA-----------CCG-TCACTTGG-TGTGATAA-TTA
TCTATGCCAC-TTGACTATGAAGCAAAACTTATGGG-AA-CCCGCTCATAACCG------TCTGCTCGT--CGGACAAC-
---------TT------TTGACATTTTGACCT--

Mycena_semivestipes_HMJAU43830

TTGG--CA-CTGATGCTGGCT-CTT-----TAC-TGAGCA-TGTGCTCGT---CC--ATCTATTT-A-TCT----TCTCT

CTGTGCACCTTTTG-TAGTCTTTGAAG---

-------------------------------------CAAGGT---------TT--------------GGAGGTTCTGTG

GGCGAAAGCCC---------------TCCCTCTG-CTGTT--

-----------ATGCTTCT-------TCAA----------GG-----------------------ACTATG-----TTTT

CA----------------------------TATA-CACTA----TTAAAGTTAC-AGAATGTC---TTTTAACGA--TTG

TGC--TAGTC-GCAGTCATT--------AAACC-T-ATACAACTTTCAGCAACGGATCTCTTGGCTCTCCCATCGATGAA

GAACGCAGCGAAATGCGATAAGTAATGTGAATTGCAGAATTCAGTGAATCATCGAATCTTTGAACGCACCTTGCGCCCTT

TGGTATTCCGAAGGGCATGCCTGTTTGAGTGTC-ATTAAATTC-TCAA---------CCTTGCTT-----GC-TTTT---

--ACTAG--CTGAG------CTCGGC---TTGGAC-GTGG-GGT--T--TTTTGCTGGC-TTCC----TTCAGT--GGAT

GGTCTGCTACCCTTAAATGCATTAGTGGGA-TCTC--TTGTGGA-----------CCG-TCACTTGG-TGTGATAA-TTA

TCTATGCCAC-TTGACTATGAAGCAAAACTTGTGGG-AA-CCCGCTCATAACCG------TCTGCTTGT--CGGACAAC-

---------TT------TTGACATTTTGACCT--

Mycena_clavicularis_HMJAU43616

TTGGGTTA-CTGATGCTGGCT-CTT-----TAC-TGAGCA-TGTGCTCGTTCCCC--TTCTATTT-A-TCT----TCTCT

-TGTGCACCTTTTG-TAGTCTTTGAAG---------------------------------AAAGTGAACC----------

-TTTTTCGCAGC-----------------------AATGCGGT---------TT--------------GGGGG-------

CATGGTAAACC---------------TGCCTCTG-CT---

------------TGCTTTC-------TTCA----------AG-----------------------GCTATG-----TTTT

CA----------------------------TATA-CACTA----T-AAAGTTAC-AGAATGTC---TTTTAACGA--TTG

TGC--TAGTC-GCAGTCATT--------AAACC-TAATACAACTTTCAACAACGGATCTCTTGGCTCTCCCATCGATGAA

GAACGCAGCGAAATGCGATAAGTAATGTGAATTGCAGAATTCAGTGAATCATCGAATCTTTGAACGCACCTTGCGCCCTT

TGGTATTCCGAAGGGCATGCCTGTTTGAGTGTC-ATTAAATTA-TCAA---------CCTTGCTT-----GC-TTTT---

--ACTAGC-TCG--------CTTGGC---TTGGAT-GTGA-GG---G--CTTTGCTGGC-TTCC----TTCAGT--GGAT

GGTCTGCTCCCTTTAAATGCATTAGTGGGA-TCTC--TTGTGGA-----------CCG-TCACTTGG-TGTGATAA-TTA

TCTATGCCAT-ATGACTTTGAAGCAAAACTTATGGG-AA-CCCGCTCATAACCG------TCCGCAA-----GGACAAC-

---------TC------TTGACATT---

Mycena_clavicularis_HMJAU43611

TTGGGTTA-CTGATGCTGGCT-CTT-----TAC-TGAGCA-TGTGCTCGTTCCCC--TTCTATTT-A-TCT----TCTCT

-TGTGCACCTTTTG-TAGTCTTTGAAG---------------------------------AAAGTGAACC----------

-TTTTTCCCAAC-----------------------CATTGGGT---------TT--------------GGGGG-------
GATGGGTAACC---------------TGCCTCCG-CT---
------------TGGTTTC-------CTCC----------AG-----------------------GGTATG-----TTTT
CC----------------------------TATA-CCCTA----T--AAGTTTC-CGAATGGC---TTTTTACCA--TTG
GGC--TAGTC-GCAGTCATT--------AAACC-TTATACAACTTTCAACAACGGATCTCTTGGCTCTCCCATCGATGAA
GAACGCAGCGAAATGCGATAAGTAATGTGAATTGCAGAATTCAGTGAATCATCGAATCTTTGAACGCACCTTGCGCCCTT
TGGTATTCCGAAGGGCATGCCTGTTTGAGTGTC-ATTAAATTA-TCAA---------CCTTGCTT-----GC-TTTT---
--ACTAAC-TCC--------CTTGGC---TTGGAT-GTGA-GG---G--CTTTGCTGGC-TTCC----TTCAGT--GGAT
GGTCTGCTCCCTTTAAATGCATTAGTGGGA-TCTC--TTGTGGA-----------CCG-TCACTTGG-TGTGATAA-TTA
TCTATGCCAT-AAGAATTTGAAGCAAAACTTATGGG-AA-CCCGCTCATAACCG------TCCGCAA-----GGACCAC-
---------TC------TTGACATT---

Mycena_filopes_HMJAU43445
AATTGGTT-CTGATGCTGGCC-TCC-----AG---GGGCA-TGTGCTCGTTCCGA--ATCTATTT-A-TCT----TCTCT
-TGTGCACCTCTTG-TAGCCCTTGCAATAAA-----A--------------------GTTAAAGTGAACC----------
---TCTCGCAGC-----------------------GATGCGGT---------TT--------------GGGGG---ACT-
----TTGAGCCGCAGCAATG-CCTCATTCCCCTGCCT---
------------TGCTTTC-------TCTTCTGTTCAAGGTG-----------------------TCTATG-----TTTT
CA----------------------------TATA-CACTA----TTCAAGTTAC-AGAATGTC---TATTAAAGA--TTG
-----CGCAA-GCAGTCATT--------AAACC-T-ATACAACTTTCAGCAACGGATCTCTTGGCTCTCCCATCGATGAA
GAACGCAGCGAAATGCGATAAGTAATGTGAATTGCAGAATTCAGTGAATCATCGAATCTTTGAACGCACCTTGCGCCCTT
TGGTATTCCGAAGGGCATGCCTGTTTGAGTGTC-ATTAAATTC-TCAA---------CCTTGCTT-----GC-TTTA---
--ACCGGC-TTGAG------TGAGGC---TTGGAT-GTGA-GG---G--CTTTGCTGGC-TTCC----TTCAGT--GGAT
GGTCTGCTCCCTTTAAATGCATTAGTGGGA-TCTC--TTGTGGA-----------CCG-TCACTTGG-TGTGATAA-TTA
TCTATGCCTCGTCGACTTTGAAGC-AAACTTATGGG-AA-CCTGCTTATAACCG------TCCTTTCAG--GACACTAA-
------C--TT----AATTGACCATTTGACCT--

Mycena_filopes_HMJAU43562
AATTGGTT-CTGATGCTGGCC-TCC-----AG---GGGCA-TGTGCTCGTTCCGA--ATCTATTT-A-TCT----TCTCT
-TGTGCACCTCTTG-TAGCCCTTGCAATAAA-----A--------------------GTTAAAGTGAACC----------
---TCTCGCAGC-----------------------GATGCGGT---------TT--------------GGGGG---ACT-
----TTGAGCCGCAGCAATG-CCTCATTCCCCTGCCT---
------------TGCTTTC-------TCTTCTGTTCAAGGTG-----------------------TCTATG-----TTTT
CA----------------------------TATA-CACTA----TTCAAGTTAC-AGAATGTC---TATTAAAGA--TTG

-----CGCAA-GCAGTCATT--------AAACC-T-ATACAACTTTCAGCAACGGATCTCTTGGCTCTCCCATCGATGAA
GAACGCAGCGAAATGCGATAAGTAATGTGAATTGCAGAATTCAGTGAATCATCGAATCTTTGAACGCACCTTGCGCCCTT
TGGTATTCCGAAGGGCATGCCTGTTTGAGTGTC-ATTAAATTC-TCAA---------CCTTGCTT-----GC-TTTA---
--ACCGGC-TCGAG------TGAGGC---TTGGAT-GTGA-GG---G--CTTTGCTGGC-TTCC----TTCAGT--GGAT
GGTCTGCTCCCTTTAAATGCATTAGTGGGA-TCTC--TTGTGGA-----------CCG-TCACTTGG-TGTGATAA-TTA
TCTATGCCTCGTCGACTTTGAAGC-AAACTTATGGG-AA-CCTGCTTATAACCG------TCCTTTCAG--GACACTAA-
------C--TT----AATTGACCATTTGACCT--

Mycena_metata_HMJAU43625
AATCGGTT-CTGATGCTGGCC-CTT-----CAC-CGGGCA-TGTGCTCGCTCCGA--ATCTATTT-A-TCT----TCTCT
-TGTGCACCTCTTG-TAGTCTTTGAAA----------------------------------AAGTGAACC----------
---TCTCGCAGC-----------------------AATGCGGT---------CT--------------GGGGG---ACTG
G--GTCAAACC---------------TTCCCCTG-CT---
------------TGCTTTC-------TTCT----------TG-----------------------GCTATG-----TTTT
CA----------------------------TATA-CACTA----T-AAAGTTAC-AGAATGTC---TTTTAACGA--TTG
TCGCTCGCGC-GCAGTCATT--------AAACC-T-ATACAACTTTCAGCAACGGATCTCTTGGCTCTCCCATCGATGAA
GAACGCAGCGAAATGCGATAAGTAATGTGAATTGCAGAATTCAGTGAATCATCGAATCTTTGAACGCACCTTGCGCCCTT
TGGTATTCCGAAGGGCATGCCTGTTTGAGTGTC-ATTAAATTC-TCAA---------CCTTGCTC-----GC-CTTT---
--ACCGGC-TTGAG------TGAGGC---TTGGAC-GTGA-GG---G--CTTTGCTGGC-TTCC----TTAAGT--GGAT
GGTCTGCTCCCTTTAAATGCATTAGTGGGA-TCTC--TTGTGGA-----------CCG-TCACTTGG-TGTGATAA-TTA
TCTACGCCTCGTCGACTTTGAAGC-AAACTTATGGG-AA-CCTGCTTATAACCG------TCTCGCAAG---AGACAAC-
------T--TT------CTGACATTTTGACCT--

Mycena_metata_HMJAU43680
AATCGGTT-CTGATGCTGGCC-CTT-----CAC-CGGGCA-TGTGCTCGCTCCGA--ATCTATTT-A-TCT----TCTCT
-TGTGCACCTCTTG-TAGTCTTTGAAA----------------------------------AAGTGAACC----------
---TCTCGCAGC-----------------------AATGCGGT---------CT--------------GGGGG---ACTG
G--GTCAAACC---------------TTCCCCTG-CT---
------------TGCTTTC-------TTCT----------TG-----------------------GCTATG-----TTTT
CA----------------------------TATA-CACTA----TACAAGTTAC-AGAATGTC---TTTTAACGA--TTG
TCGCTCGCGC-GCAGTCATT--------AAACC-T-ATACAACTTTCAGCAACGGATCTCTTGGCTCTCCCATCGATGAA
GAACGCAGCGAAATGCGATAAGTAATGTGAATTGCAGAATTCAGTGAATCATCGAATCTTTGAACGCACCTTGCGCCCTT
TGGTATTCCGAAGGGCATGCCTGTTTGAGTGTC-ATTAAATTC-TCAA---------CCTTGCTC-----GC-TTTT---
--ACCGGC-TTGAG------TGAGGC---TTGGAC-GTGA-GG---G--CTTTGCTGGC-TTCC----TTAAGTG-GGAT

GGTCTGCTCCCTTTAAATGCATTAGTGGGA-TCTC--TTGTGGA-----------CCG-TCACTTGG-TGTGATAA-TTA
TCTACGCCTCGTCGACTTTGAAGC-AAACTTATGGG-AA-CCTGCTTATAACCG------TCTCGCGAG---GGACAAC-
------T--TT------CTGACATTTTGACCT--

Mycena_maculata_MK309791
TTGG-TTT-CTGATGCTGGCC-TTT-----TAC-CAGGCA-TGTGCTCGAT-TCC--TTCTATTT-A-TCT----TCTCT
-TGTGCACCTTTTG-TAGTCTTTTGAA--------------------------------TGAGCCAACC----------
----TTCGCTGAA----------------------AATGCGGT---------TT--------------GGGGG---ATTG
AGCGTAACAGCAC-------------TTCCCCTG-CGGTTCACTTCGCAGCAATGCGGTTTGGGGGTCTGAGCCTTTATT
GGCACT-----ATCCCTGC-------TTCA---------AGG-----------------------ACTATG-----TTTT
CA----------------------------TACA-CACTA----TAAAAGTTATCGGAATGTC---TTTTAACGA--TTG
TGC-CTAGTC-GCAGTCATT--------AAACC-T-ATACAACTTTCAGCAACGGATCTCTTGGCTCTCCCATCGATGAA
GAACGCAGCGAAATGCGATAAGTAATGTGAATTGCAGAATTCAGTGAATCATCGAATCTTTGAACGCACCTTGCGCCCTT
TGGTATTCCGAAGGGCATGCCTGTTTGAGTGTC-ATCAAATTA-TCAA---------CCTTGCTT-----GC-TTTT---
--ACTGGC-TCG--------CTCGGC---TTGGAT-GTGA-GG---G----CTGCTGGC-TTCC----TTCAGT--GGAT
GGTCTGCTCCCTTTAAATGCATTAGTGGGA-TCTC--TTGTGGA-----------CCG-TCACTTGG-TGTGATAA-TTA
TCTATGCCAG-TTGACTTTGAAGC-AAACTTATGGG-AA-CCTGCTTATAACCG------TCCTTTG-----GGACAAC-
----T------------TTAACATTTTGACCT--

Mycena_maculata_MK309792
TTGG-TTT-CTGATGCTGGCC-TTT-----TAC-TGGGCA-TGTGCTCGAT-TCC--TTCTATTT-A-TCT----TCTCT
-TGTGCACCTTTTG-TAGTCTTTTGAA--------------------------------TGAGCCAACC----------
----TTCGCTGAA----------------------AATGCGGT---------TT--------------GGGGG---ATTG
AGCGTAACAGCAC-------------TTCCCCTG-CGGTTCACTTCGCAGCAATGCGGTTTGGGGATCTGAGCCTTTATT
GGCACT-----ATCCCTGC-------TTCA---------AGG-----------------------ACTATG-----TTTT
CA----------------------------TACA-CACTA----TAAAAGTTATCGGAATGTC---TTTTAACGA--TTG
TGC-CTAGTC-GCAGTCATT--------AAACC-T-ATACAACTTTCAGCAACGGATCTCTTGGCTCTCCCATCGATGAA
GAACGCAGCGAAATGCGATAAGTAATGTGAATTGCAGAATTCAGTGAATCATCGAATCTTTGAACGCACCTTGCGCCCTT
TGGTATTCCGAAGGGCATGCCTGTTTGAGTGTC-ATCAAATTA-TCAA---------CCTTGCTT-----GC-TTTT---
--ACTAGC-TCG--------CTCGGC---TTGGAT-GTGA-GG---G----CTGCTGGC-TTCC----TTCAGT--GGAT
GGTCTGCTCCCTTTAAATGCATTAGTGGGA-TCTC--TTGTGGA-----------CCG-TCACTTGG-TGTGATAA-TTA
TCTATGCCAG-TTGACTTTGAAGC-AAACTTATGGG-AA-CCTGCTTATAACCG------TCCTTTG-----GGACAAC-
----T------------TTAACATTTTGACCT--

FLF744
TTGG--TA-CTGTTGCTGGCT-CTT-----CGC-TGAGCA-TGTGCACG---TCC--ATCTATTT-A-TCT----TCTCT
-TGTGCACCTTTTG-TAGTCTTTTGAAAAAG---
-----------------------------------AGTTGGGT----GTGTCCTCGCAA---------GGGGG-------
---------------------GACGCTTTGCCCTTTT---
--------------------------TTCG---------GAG-----------------------GCTACG-----TTTT
CA----------------------------TATA-CACTT----ATAAAGTTAC-AGAATGTC---TTTTAAAGA--TTG
TGC--TAGTC-GCAGTCATT--------AAACC-T-ATACAACTTTCAGCAACGGATCTCTTGGCTCTCCCATCGATGAA
GAACGCAGCGAAATGCGATAAGTAATGTGAATTGCAGAATTCAGTGAATCATCGAATCTTTGAACGCACCTTGCGCCCTT
TGGTATTCCGAAGGGCATGCCTGTTTGAGTGTC-ATTAAATTA-TCAA---------CCTTAGAT-----GC-TTT----
------GC-TTGCGAAGCGTCTAGGC---TTGGAT-GTGG-GG---G-TGTTTGCTGGC-TTCC----TTCAGT--GGAT
GGTCTGCTCCCCTTAAATGCATTAGCGGGA-TCTC--TTGTGGA-----------CCG-TCACTTGG-TGTGATAA-TTA
TCTATACCAC-TTGGCTTTGAAGCAAATCTTATGAG-GA-CCCGCTCATAACCG------TCCCTTCCC--GGGACAAC-
---------TC------TTGACATTTTGACCT-GCCTTCACCCTCGGTGTGCGTCAGCTCATCGTGGCCGTCAACAAGAT
GGACACCACCAAGGTGAG----TCTTTTTTTC-CT-----------------AATTTGGATATTAATTTTCTTAATCCAA
CGGTCCGGGGGAAGGGAAGGGAGAACGGTTCCATGGAATTATTCAGGAAAACGCCCCCTTCCTTCAAAAAGGCCGGTACC
ACCCCCAAAATAATGGCTTCCGCCCCCTTTCCGGCTGGGACGG-GGAACAAAT--GGTGGAAGAAGCCAACAAAGATGAA
CTCCTCCTTT--CCCCGCTTTAA--------------TTAATTAATCTAAACGGTTGGCCAACATGGCCTGG-TTCCAGG
GCTGGAACCAGGAAAA---CCAAGGTTGG--GGCCGCCAGGGCAAAACCCTTCTT--GAAGGTAACC-------------
-ATTGCCTTGAGGCCCCATTCCGGCCAAGGCCA-
MSX744
TTGG--TA-CTGTTGCTGGCT-CTT-----CGC-TGAGCA-TGTGCACG---TCC--ATCTATTT-A-TCT----TCTCT
-TGTGCACCTTTTG-TAGTCTTTTGAAAAAG---
-----------------------------------AGTTGGGT----GTGTCCTCGCAA---------GGGGG-------
---------------------GACGCTTTGCCCTTTT---
--------------------------TTCG---------GAG-----------------------GCTACG-----TTTT
CA----------------------------TATA-CACTT----ATAAAGTTAC-AGAATGTC---TTTTAAAGA--TTG
TGC--TAGTC-GCAGTCATT--------AAACC-T-ATACAACTTTCAGCAACGGATCTCTTGGCTCTCCCATCGATGAA
GAACGCAGCGAAATGCGATAAGTAATGTGAATTGCAGAATTCAGTGAATCATCGAATCTTTGAACGCACCTTGCGCCCTT
TGGTATTCCGAAGGGCATGCCTGTTTGAGTGTC-ATTAAATTA-TCAA---------CCTTAGAT-----GC-TTT----
------GC-TTGCGAAGCGTCTAGGC---TTGGAT-GTGG-GG---G-TGTTTGCTGGC-TTCC----TTCAGT--GGAT
GGTCTGCTCCCCTTAAATGCATTAGCGGGA-TCTC--TTGTGGA-----------CCG-TCACTTGG-TGTGATAA-TTA
TCTATACCAC-TTGGCTTTGAAGCAAATCTTATGAG-GA-CCCGCTCATAACCG------TCCCTTCCC--GGGACAAC-
---------TC------TTGACATTTTGACCT-GCCTTCACCCTCGGTGTGCGTCAGCTCATCGTGGCCGTCAACAAGAT
GGACACCACCAAGGTGAG----TCTTTTTTTC-CT-----------------AATTTGGATATTAATTTTCTTAATCCAA
CGGTCCGGGGGAAGGGAAGGGAGAACGGTTCCATGGAATTATTCAGGAAAACGCCCCCTTCCTTCAAAAAGGCCGGTACC
ACCCCCAAAATAATGGCTTCCGCCCCCTTTCCGGCTGGGACGG-GGAACAAAT--GGTGGAAGAAGCCAACAAAGATGAA
CTCCTCCTTT--CCCCGCTTTAA--------------TTAATTAATCTAAACGGTTGGCCAACATGGCCTGG-TTCCAGG
GCTGGAACCAGGAAAA---CCAAGGTTGG--GGCCGCCAGGGCAAAACCCTTCTT--GAAGGTAACC-------------

-ATTGCCTTGAGGCCCCATTCCGGCCAAGGCCA-
Mycena_epipterygia_olrim523
--------------------------------C-TGAGCA-TGTGCACG---TCC--ATCTATTT-A-TCT----TCTCT
-TGTGCACCTTTTG-TAGTCTTTTGAAGAAG---
-----------------------------------AGTGGGGT---GGTATCCTC---A---------GGGAG-------
---------------------AACGCTTCGCCCTTTT---
--------------------------TTCA---------GAG-----------------------GCTATG-----TTTT
CA----------------------------TATA-CAC-T----ATAAAGTTAC-AGAATGTC---TTTTAAAGA--TTG
TGC--TAGCC-GCAGTCATT--------AAACC-T-ATACAACTTTCAGCAACGGATCTCTTGGCTCTCCCATCGATGAA
GAACGCAGCGAAATGCGATAAGTAATGTGAATTGCAGAATTCAGTGAATCATCGAATCTTTGAACGCACCTTGCGCCCTT
TGGTATTCCGAAGGGCATGCCTGTTTGAGTGTC-ATTAAATTA-TCAA---------CCTT-CTT-----GC-TTT----
------GC-TTGCAAAGCATCTAGGC---TTGGAT-GTGG-GG---G-TTTTTGCTGGC-TTCC----TTCAGT--GGAT
GGTCTGCTCCCCTTAAATGCATTAGCGGGA-TCTC--TTGTGGA-----------CCG-TCACTTGG-TGTGATAA-TTA
TCTATACCAC-TTGGCCTTGAAGCAAATCTTATGAG-GA-CCCGCTCATAACCG------TCCCTTATG---GGACAAC-
---------TC------TTGACA---

Mycena_epipterygia_JB13
TTGG--TA-CTGTAGCTGGCTACTT-----CAC-TGAGCA-TGTGCACG---TCC--ATCTATTT-A-TCT----TCTCT
-TGTGCACCTTTTG-TAGTCTTTTGAAGAAG---
-----------------------------------AGTGGGGT---GGTATCCTC---A---------GGGAG-------
---------------------AACGCTTCGCCCTTTT---
--------------------------TTCA---------GAG-----------------------GCTATG-----TTTT
CA----------------------------TATA-CAC-T----ATAAAGTTAC-AGAATGTC---TTTTAAAGA--TTG
TGC--TAGCC-GCAGTCATT--------AAACC-T-ATACAACTTTCAGCAACGGATCTCTTGGCTCTCCCATCGATGAA
GAACGCAGCGAAATGCGATAAGTAATGTGAATTGCAGAATTCAGTGAATCATCGAATCTTTGAACGCACCTTGCGCCCTT
TGGTATTCCGAAGGGCATGCCTGTTTGAGTGTC-ATTAAATTA-TCAA---------CCTT-CTT-----GC-TTT----
------GC-TTGCAAAGCATCTAGGC---TTGGAT-GTGG-GG---G-TTTTTGCTGGC-TTCC----TTCAGT--GGAT
GGTCTGCTCCCCTTAAATGCATTAGCGGGA-TCTC--TTGTGGA-----------CCG-TCACTTGG-TGTGATAA-TTA
TCTATACCAC-TTGGCCTTGAAGCAAATCTTATGAG-GA-CCCGCTCATAACCG------TCCCTTCTG---GGAC----
----

Mycena_laevigata_HMJAU43187
CTTGGGTG-CTGATGCTGGCC-------TTTAC-TGGCAC-TGTGCTCGCC-CCA--AACTATTT-A-TTA----TCTCT
ATGTGCACCTTTTG-TAGTCCGTTTTGTTAATGGAG------------------------GGAGCAATCCCTTCAAGGAG

CCTTTCCGCAGTC----------------------AATGCGGG-------TTTT--------------GTGGG---TTTG
AGCGCGCGAGCGCAC-----------TTTCCCTGTCTTCTCCACAAGCAATTGTG-GGTTTGGGGCATTGAGCGTGCAAG
CGCACTTGTCCTTGGCTTC------CTTCA-------GAAAG-----------------------GCTATG-----TTTT
TA----------------------------TATA-CCCTA----TTAAAGTTAC-AGAATGTCT--TTTTAACGA--TTG
TGC--TAGTC-GCAGTCATT--------AAATC-T-ATACAACTTTCAGCAACGGATCTCTTGGCTCTCCCATCGATGAA
GAACGCAGCGAAATGCGATAAGTAATGTGAATTGCAGAATTCAGTGAATCATCGAATCTTTGAACGCACCTTGCGCCCTT
TGGTATTCCGAAGGGCATGCCTGTTTGAGTGTC-ATTAAATTA-TCAA---------CCTTGCGC-----GC-TTTT---
--ACTAGTGTCG--------CTTGGC---TTGGAT-GTGA-GG---G--TTTTGCTGGC-TTCC----TTCAGTG-GATT
GGTCTGCTCCCTTTAAATGCATTAGTGGGA-TCTC--TTGTGGA-----------CCG-TCACTTGG-TGTGATAA-TTA
TCTATGCCGT-TTGACTTTGAAGC-AAATTTATGGG-AA-CCTGCTTATAACCG------TCCTTTACC---GGACAAC-
AATCT-T--AA------TTGACATTTTGACCT--

Mycena_laevigata_MJAU43604
CTTGGGTG-CTGATGCTGGCC-------TTTAC-TGGCAC-TGTGCTCGCC-CCA--AACTATTT-A-TTA----TCTCT
ATGTGCACCTTTTG-TAGTCCGTTTTGTTAATGGAG------------------------GGAGCAATCCCTTCAAGGAG
CCTTTCCGCAGTC----------------------AATGCGGG-------TTTT--------------GTGGG---TTTG
AGCGCGCGAGCGCAC-----------TTTCCCTGTCTTCTCCACAAGCAATTGTG-GGTTTGGGGCATTGAGCGTGCAAG
CGCACTTGTCCTTGGCTTC------CTTCA-------GAAAG-----------------------GCTATG-----TTTT
TA----------------------------TATA-CCCTA----TTAAAGTTAC-AGAATGTCT--TTTTAACGA--TTG
TGC--TAGTC-GCAGTCATT--------AAATC-T-ATACAACTTTCAGCAACGGATCTCTTGGCTCTCCCATCGATGAA
GAACGCAGCGAAATGCGATAAGTAATGTGAATTGCAGAATTCAGTGAATCATCGAATCTTTGAACGCACCTTGCGCCCTT
TGGTATTCCGAAGGGCATGCCTGTTTGAGTGTC-ATTAAATTA-TCAA---------CCTTGCGC-----GC-TTTT---
--ACTAGTGTCG--------CTTGGC---TTGGAT-GTGA-GG---G--TTTTGCTGGC-TTCC----TTCAGTG-GATT
GGTCTGCTCCCTTTAAATGCATTAGTGGGA-TCTC--TTGTGGA-----------CCG-TCACTTGG-TGTGATAA-TTA
TCTATGCCGT-TTGACTTTGAAGC-AAATTTATGGG-AA-CCTGCTTATAACCG------TCCTTTACC---GGACAAC-
AATCT-T--AA------TTGACATTTTGACCT--

Mycena_sanguinolenta_ASIS21424
ATTG-GTA-CTGATGCTGGCT-CTT-----CAC-TGAGCA-TGTGCTCGT--CCA--TCTTATTT-A-TCT----TCTCT
-TGTGCACATTTTG-TAGTCTTGAAGACCTCTGCGCAAATACATTTGCTGCAGGTTGGGAGATGCTAACCCT--------
-TCTCTCGTTGCCTT--------------------TGTGCAAG-------CTTT--------------GGGGA-------
---------------ATGTT-AACCCTTTCCTCGCGT---
-------------------------CTTCA----------AG-----------------------ACTATG-----TTTT
CA----------------------------TATA-CACT-----ATAAAGTTAC-AGAATGTC---TTTTAACGA-----

----CTGCTT-GCAGTCATT--------AAACC-T-ATACAACTTTCAGCAACGGATCTCTTGGCTCTCCTATCGATGAA
GAACGCAGCGAAATGCGATAAGTAATGTGAATTGCAGAATTCAGTGAATCATCGAATCTTTGAACGCACCTTGCGCCCTT
TGGTATTCCGAAGGGCATGCCTGTTTGAGTGTC-ATTAAATTA-TCAA---------CCTTCCTCT---TGC-TTTC---
--ATTAGT-TYGAG------TTAGGC---TTGGAT-GTGA-GG---G--TCTTGCCGGC-TTTT--------------GT
GGTCAGCTCCCTTTAAATGCATTAGTGGGA-TCTA--TTGTGGA-----------CCG-TCACTTGG-TGTGATAA-TTA
TCTATGCCTT-GAGACTTTGAAGC-AAATTTATTAG-AA-CCTGCTTATAACCG------TCCTTCACG---GGACAAC-
---------TT------ATGACAATT-GACCT--

Mycena_sanguinolenta_CBHHK098
-----GTA-CTGATGCTGGCT-CTT-----CAC-TGGGCA-TGTGCTCGT--CCA--TCTTATTT-A-TCT----TCTCT
-TGTGCACATTTTG-TAGTCTTGAAGAACTCCGCGCA--TTCATTTGTTGCGGGCTGGGAGATGCTAACCCT--------
-TCTCTCGTTGCCTT--------------------TGTGCAAG-------CCTT--------------GGGGG-------
---------------TTGTT-AACCCTTTCCCCGCTT---
-------------------------CTTCA----------AG-----------------------ACTATG-----TTTT
CA----------------------------TATA-CCCT-----ATAAAGTTAC-AGAATGTC---TTTTAACGA-----
----CTGCTT-GCAGTCATT--------AAACC-T-ATACAACTTTCAGCAACGGATCTCTTGGCTCTCCTATCGATGAA
GAACGCAGCGAAATGCGATAAGTAATGTGAATTGCAGAATTCAGTGAATCATCGAATCTTTGAACGCACCTTGCGCCCTT
TGGTATTCCGAAGGGCATGCCTGTTTGAGTGTC-ATTAAATTA-TCAA---------CCTTGCTC-----GC-TTTT---
--ACTAGC-TTGAG------TTAGGC---TTGGAT-GTGA-GG---G--TTTTGCTGGC-TTTC--------------GT
GGTCTGCTCCCTTTAAATGCATTAGTGGGA-TCTA--TTGTGGA-----------CCG-TCACTTGG-TGTGATAA-TTA
TCTATGCTTT-GGGACTTTGAAAC-AAACTTTTTAG-AA-TCCGCTTATAACCG------TCCTCACGG---ACA-----
----

FLF1861
ATTG-GTG-CTGTTGCTGGCTTCTTTA--ACTT-GAGGCA-TGTGCACGCCCCAT--ATCTATTT-A-TCT----TCTCT
-TGTGCACATTTTG-TGGTCTTGAATCGAAAAA---
-CCCCTCGCAGC-----------------------AATGCGGT------TTTTT--------------GGGAG---GCTG
GGT-------------TTTT-TGCCCTTCTCCTGCTT---
------------------------TCTTCA----------AG-----------------------GCTACG-----TTTT
CA----------------------------TATA-CACGT--TTTTAAAGTTAC-AGAATGTCTTTTTTTAACGA--TTG
TGC-TTGGTT-GCAGTCATT--------AAACC-T-ATACAACTTTCAGCAACGGATCTCTTGGCTCTCCCATCGATGAA
GAACGCAGCGAAATGCGATAAGTAATGTGAATTGCAGAATTCAGTGAATCATCGAATCTTTGAACGCACCTTGCGCCCTT
TGGTATTCCGAAGGGCATGCCTGTTTGAGTGTC-ATTAAATTA-TCAA---------CCTTGTTC-----GC-TTTT---
--CGGAGC-TTGAG------CTAGGC---TTGGAT-GTGA-GG---GCTTTTTGCTGGC-TTCC----TTCAGTG-GATC

GGTCTGCTCCCTTTAAATGCATTAGTGGGA-TCTCTTTTGTGGA-----------CCG-TCACTTGG-TGTGATAA-TTA
TCTACGCCAT--TGACT--------TGCTTAACGAG-AA-TCTGCTTATAACCG------TCTCGCCAAA-GGGACAAC-
AAC-T-T--TA------TTGACAATTTGACCT-GCGTTCACCCTCGGTGTGCGTCAGCTCATCGTTGCTGTCAACAAGAT
GGACACGACCAAGGTTTG----CCTTTTTTGG-TCTCT-------------TCTTTTGGCTATT----TTCTCAACTCGA
CAACTTTTTACAGTGGAGCGAGGATCGTTTCAATGAAATTATCAAAGAGACGTCCACCTTCATCAAGAAGGTCGGGTACA
ACCCCAAAAGCATTGCCTTCGTCCCCATCTCGGGCTGGCACGGTTGACAACATGGGTTGGAGGAGTCCGACCAAAGTATG
ATTCCCAAACTTTCCATTGCTAAGCATAGGC---ACTTTTGCTTGAT---ACGGCCTCTCAGCATGCCCTGGGTTCAAGG
GCTGGGACCAAGGGAGACCAAAAGCCGGTTGGTCGTCAAGGGCAAAGACCCTCCCTCGAAGGCTATCGGAATGCCCATCG
AAGCCCCCCGGGGTCCGGCCCCTTCTGACAAAA-
MSX1861
ATTG-GTG-CTGTTGCTGGCTTCTTTA--ACTT-GAGGCA-TGTGCACGCCCCAT--ATCTATTT-A-TCT----TCTCT
-TGTGCACATTTTG-TGGTCTTGAATCGAAAAA---
-CCCCTCGCAGC-----------------------AATGCGGT------TTTTT--------------GGGAG---GCTG
GGT-------------TTTT-TGCCCTTCTCCTGCTT--
------------------------TCTTCA----------AG-----------------------GCTACG-----TTTT
CA----------------------------TATA-CACGT--TTTTAAAGTTAC-AGAATGTCTTTTTTTAACGA--TTG
TGC-TTGGTT-GCAGTCATT--------AAACC-T-ATACAACTTTCAGCAACGGATCTCTTGGCTCTCCCATCGATGAA
GAACGCAGCGAAATGCGATAAGTAATGTGAATTGCAGAATTCAGTGAATCATCGAATCTTTGAACGCACCTTGCGCCCTT
TGGTATTCCGAAGGGCATGCCTGTTTGAGTGTC-ATTAAATTA-TCAA---------CCTTGTTC-----GC-TTTT---
--CGGAGC-TTGAG------CTAGGC---TTGGAT-GTGA-GG---GCTTTTTGCTGGC-TTCC----TTCAGTG-GATC
GGTCTGCTCCCTTTAAATGCATTAGTGGGA-TCTCTTTTGTGGA-----------CCG-TCACTTGG-TGTGATAA-TTA
TCTACGCCAT--TGACT--------TGCTTAACGAG-AA-TCTGCTTATAACCG------TCTCGCCAAA-GGGACAAC-
AAC-T-T--TA------TTGACAATTTGACCT-GCGTTCACCCTCGGTGTGCGTCAGCTCATCGTTGCTGTCAACAAGAT
GGACACGACCAAGGTTTG----CCTTTTTTGG-TCTCT-------------TCTTTTGGCTATT----TTCTCAACTCGA
CAACTTTTTACAGTGGAGCGAGGATCGTTTCAATGAAATTATCAAAGAGACGTCCACCTTCATCAAGAAGGTCGGGTACA
ACCCCAAAAGCATTGCCTTCGTCCCCATCTCGGGCTGGCACGGTTGACAACATGGGTTGGAGGAGTCCGACCAAAGTATG
ATTCCCAAACTTTCCATTGCTAAGCATAGGC---ACTTTTGCTTGAT---ACGGCCTCTCAGCATGCCCTGGGTTCAAGG
GCTGGGACCAAGGGAGACCAAAAGCCGGTTGGTCGTCAAGGGCAAAGACCCTCCCTCGAAGGCTATCGGAATGCCCATCG
AAGCCCCCCGGGGTCCGGCCCCTTCTGACAAAA-
Mycena_hyalinostipitata_NX0686
ATTGGGCG-CTGT-GCTGGCC-TT-------TC-GGGGCA-TGTGCACGCGTTCCAAGTCTTTTC-A-TCT---TTCTTT
-TGTGAACCTAGTG-TAGGCTTTGGATGAGTG--
-----------------------------------AGCGGGGTAAAACCTGTTT--------------GCGAG-------
----
--------------------------TTTG---------AAG-----------------------ACTA-------TTTT
TA----------------------------TAAA-CCAA-----GCAAAATTATTAGAATGTCT-ATCTTTGTTA-----
--C-TTGGCG-CAAGCCAGTA---ATGAAAACC-T-ATACAACTTTCAGCAACGGATCTCTTGGCTCTCCCATCGATGAA
GAACGCAGCGAAATGCGATAAGTAATGTGAATTGCAGAATTCAGTGAATCATCGAATCTTTGAACGCACCTTGCGCCCTT
TGGTATTCCGAAGGGCATGTCTGTTTGAGTGTC-ATTAAATTC-TCAA---------CCTTGTAA-----ACGCTTTTTT
T-GGGTGTTTAT--------TGAGGC---TTGGAT-GTGA-GG---G-T-TTTGCTGGC-TTCC----TTCAGT---GGT
GGTCTGCTCCCTTTAAAAGCATTAGTGGGA-TCCT--TTGTGGAA--------CTTGT-TCACTTGG-TGTGATAATTTA
TCTACGCCGC-TTGAATGTCTA-CAAAACCTATGGG-AA-CCTGCTTACAACCG------TCTGTAA----TGGACAAT-
---------TT------TTGAACC-TTGACCT--

Mycena_hyalinostipitata_NX0694
ATTGGGCG-CTGT-GCTGGCC-TT-------TC-GGGGCA-TGTGCACGCGTTCCAAGTCTTTTC-A-TCT---TTCTTT
-TGTGAACCTAGTG-TAGGCTTTGGATGAGTG--
-----------------------------------AGCGGGGTAAAACCTGTTT--------------GCGAG-------
----
--------------------------TTTG---------AAG-----------------------ACTA-------TTTT
TA----------------------------TAAA-CCAA-----GCAAAATTATTAGAATGTCT-ATCTTTGTTA-----
--C-TTGGCG-CAAGCCAGTA---ATGAAAACC-T-ATACAACTTTCAGCAACGGATCTCTTGGCTCTCCCATCGATGAA
GAACGCAGCGAAATGCGATAAGTAATGTGAATTGCAGAATTCAGTGAATCATCGAATCTTTGAACGCACCTTGCGCCCTT
TGGTATTCCGAAGGGCATGTCTGTTTGAGTGTC-ATTAAATTC-TCAA---------CCTTGTAA-----ACGCTTTTTT
T-GGGTGTTTAT--------TGAGGC---TTGGAT-GTGA-GG---G-T-TTTGCTGGC-TTCC----TTCAGT---GGT
GGTCTGCTCCCTTTAAAAGCATTAGTGGGA-TCCT--TTGTGGAA--------CTTGT-TCACTTGG-TGTGATAATTTA
TCTACGCCGC-TTGAATGTCTA-CAAAACCTATGGG-AA-CCTGCTTACAACCG------TCTGTAA----TGGACAAT-
---------TT------TTGAACCTTTGACCT--

Mycena_substylobates_NX0571
ATTGGGCG-CTGT-GCTGGCC-TT-------TC-GGGGCA-TGTGCACGCGTTCG--ACATTATT-A-TCT---ATCTTT
-TGTGAACCTAGTG-TGGGCTTTGAATGCGTA--
-----------------------------------GACGGGG-GCAACCTGAAT--------------ACAAG-------
----
--------------------------TTCG---------AAG-----------------------ACCA-------TTTT
TA----------------------------TAAA-CCTAT----AAAAAGTTAACAGAATG-----TCTTTGTTA-----
--C-TTGACT-TTCGTCAGTA---ATG-AAACC-T-ATACAACTTTCAGCAACGGATCTCTTGGCTCTCCCATCGATGAA
GAACGCAGCGAAATGCGATAAGTAATGTGAATTGCAGAATTCAGTGAATCATCGAATCTTTGAACGCACCTTGCGCCCTT
TGGCATTCCGAAGGGCATGCTTGTTTGAGTGTC-ATTAAATTC-TCAA---------CCTTGTAA-----ATGCTTT---
---TGTGTTTCA--------CAAGGC---TTGGAT-GTGA-GG---G-T-TTTGCTGGC-TTCC----TTCAGT---GGC
GGTCTGCTCCCTTTAAATGTATTAGTGGAA-TCCT--TTGTGGA-----------CGG-ACACTTGG-TGTGATAATTTA
TCTACGCCGC-TTGTACTGAAAGCAAAACCTGTGGG-AC-TCTGCTTATAACTG------TCTGTAA----TGGACAAC-
------T-TTT------TTTGAACTATGACCT--

Mycena_substylobates_NX0574

ATTGGGCG-CTGT-GCTGGCC-TT-------TC-GGGGCA-TGTGCACGCGTTCG-AACATTATT-A-TCT---ATCTTT
-TGTGAACCTAGTG-TGGGCTTTGAATGCGTA--
-----------------------------------GACGGGG-GCAACCTGAAT--------------ACAAG-------
----
--------------------------TT-G---------AAG-----------------------ACCA-------TTTT
TA----------------------------TAAA-CCTAT----AAAAAGTTAACAGAATG-----TCTTTGTTA-----
--C-TTGACT-TTCGTCAGTA---ATG-AAACC-T-ATACAACTTTCAGCAACGGATCTCTTGGCTCTCCCATCGATGAA
GAACGCAGCGAAATGCGATAAGTAATGTGAATTGCAGAATTCAGTGAATCATCGAATCTTTGAACGCACCTTGCGCCCTT
TGGTATTCCGAAGGGCATGCTTGTTTGAGTGTC-ATTAAATTC-TCAA---------CCTTGTAA-----ATGCTTT---
---TGTGTTTCA--------CAAGGC---TTGGAT-GTGA-GG---G-T-TTTGCTGGC-TTCC----TTCAGT---GGC
GGTCTGCTCCCTTTAAATGTATTAGTGGAA-TCCT--TTGTGGA-----------CGG-ACACTTGG-TGTGATAATTTA
TCTACGCCGC-TTGTACTGAAAGCAAAACCTGTGGG-AC-TCTGCTTATAACTG------TCTGTAA----TGGACAAC-
------T-TTT------TTTGAACTATGACCT--

M._stylobatesJS151130

ATCGAGCG-CTGT-GCTGGCC-TT-------CC-GGGGCA-TGTGCACGCGTTCT-----TTAAT-A-TATA--ATCTCT
-TGTGAACCTAGTG-TGGGCTTTGGAGGAACA--
-----------------------------------GTTGGTCTTTGTGCCTTCT--------------GGGAA-------
----
--------------------------TCTG---------AAG-----------------------ACCA-------TTTT
TA----------------------------TACA-CGTTT-TATAAAAAGTTAACAGAATG-----TCTTGTTTA-----
--C-TTGACG-CTAGTCAGTA---AAAAAAAAC-T-ATACAACTTTCAGCAACGGATCTCTTGGCTCTCCCATCGATGAA
GAACGCAGCGAAATGCGATAAGTAATGTGAATTGCAGAATTCAGTGAATCATCGAATCTTTGAACGCACCTTGCGCCCTT
TGGTATTCCGAAGGGCATGCCTGTTTGAGTGTC-ATTAAATTC-TCAA---------CCTTGAAAGGCTTGTCTTTT---
-------------------CAATGGT---TTGGAT-GTGA-GG---G-T-TTTGCTGGC-TTCC----TTCAGT--GGAT
GGTCTGCTCCCTTTAAATGCATTAGTGGGA-TCCT--TTGTGGA-----------CGG-TCACTTGG-TGTGATAA-TTA
TCTACGCCGCTTGTACTGTGAAGCAAAACTTGTAGG-AA-CCTGCTTATAACCG------TCTGTAA----TGGACAAT-
------T----------TTGAACATTTGACCT--

M._stylobatesJS1511302

ATCGAGCG-CTGT-GCTGGCC-TT-------CC-GGGGCA-TGTGCACGCGTTCT-----TTAAT-A-TATA--ATCTCT
-TGTGAACCTAGTG-TGGGCTTTGGAGGAACA--

-----------------------------------GTTGGTCTTTGTGCCTTCT--------------GGGAA-------
----
--------------------------TCTG---------AAG-----------------------ACCA-------TTTT
TA----------------------------TACA-CGTTT-TATAAAAAGTTAACAGAATG-----TCTTGTTTA-----
--C-TTGACG-CAAGTCAGTAAA-AAAAAAAAC-T-ATACAACTTTCAGCAACGGATCTCTTGGCTCTCCCATCGATGAA
GAACGCAGCGAAATGCGATAAGTAATGTGAATTGCAGAATTCAGTGAATCATCGAATCTTTGAACGCACCTTGCGCCCTT
TGGTATTCCGAAGGGCATGCCTGTTTGAGTGTC-ATTAAATTC-TCAA---------CCTTGAAAGGCTTGTCTTTT---
-------------------CAATGGT---TTGGAT-GTGA-GG---G-T-TTTGCTGGC-TTCC----TTCAGT--GGAT
GGTCTGCTCCCTTTAAATGCATTAGTGGGA-TCCT--TTGTGGA-----------CGG-TCACTTGG-TGTGATAA-TTA
TCTACGCCGCTTGTACTGTGAAGCAAAACTTGTAGG-AA-CCTGCTTATAACCG------TCTGTAA----TGGACAAT-
------T----------TTGAACATTTGACCT--

Mycena_tenerrima_HMJAU43646
TTGGGGCG-CTGT-GCTGGCC-TT-------TC-GGGGCA-TGTGCACGCGTTTC-CATCTATTT-A-CTT---ATCTCT
-TGTGAACCTAGTG-TGGGCTTTGAATGAGTG--
-----------------------------------GGTTCGAGTGGAACTGGTCTTTCATTAGATTGGGGAAG---CTTG
GGC---
------------TAGCTTG-------TTTG---------AAG-----------------------ACCAT------TTTT
TA----------------------------TAAA-CCTA--TTTGAAATTTATC-AGAATGT----ACTTCTTTA-----
--C-TTGACG-CGAGTCAGT-----AAGAACCT-T-ATACAACTTTCAGCAACGGATCTCTTGGCTCTCCCATCGATGAA
GAACGCAGCGAAATGCGATAAGTAATGTGAATTGCAGAATTCAGTGAATCATCGAATCTTTGAACGCACCTTGCGCCCTT
TGGTATTCCGAAGGGCATGCTTGTTTGAGTGTC-ATTAACTTC-TCAA---------CCTTGGTG----GAC-TTTG---
---------TTGTTCACT-TCAAGGC---TTGGAT-GTGA-GG---G-TCTTTGCTGGC-TTCC----TTCAGT---GGC
GGTCTGCTCCCTTTAAATGCATTAGTGGAA-TCCT--TTGTGGA-----------CGG-TCACTTGG-CTTGATAA-TTA
TCTAAGCTTTGTGCGCTGTGAAGCAAGCTTTATAGG-ACTTCTGCTTATAACCG------TCTTGTAAT--GAGACAAC-
------T--TT------TTGAACCTTTGACCT--

Mycena_tenerrima_HMJAU43816
TTGGGGCG-CTGT-GCTGGCC-TT-------TC-GGGGCA-TGTGCACGCGTTTC-CATCTATTT-A-CTT---ATCTCT
-TGTGAACCTAGTG-TGGGCTCTGAATGAGTG--
-----------------------------------GGTTCGAGTGGAACTGGTCTTTCATTAGATTGGGGAAG---CTTG
GGC---
------------TAGCTTG-------TTTG---------AAG-----------------------ACCAT------TTTT
TA----------------------------TAAA-CCTA--TTTGAAATTTATC-AGAATGT----ACTTCTTTA-----

--C-TTGACG-CGAGTCAGT-----AAGAACCT-T-ATACAACTTTCAGCAACGGATCTCTTGGCTCTCCCATCGATGAA
GAACGCAGCGAAATGCGATAAGTAATGTGAATTGCAGAATTCAGTGAATCATCGAATCTTTGAACGCACCTTGCGCCCTT
TGGTATTCCGAAGGGCATGCTTGTTTGAGTGTC-ATTAACTTC-TCAA---------CCTTGGTG----GAC-TTTG---
---------TTGTTCGCT-TCAAGGC---TTGGAT-GTGA-GG---G-TCTTTGCTGGC-TTCC----TTCAGT---GGC
GGTCTGCTCCCTTTAAATGCATTAGTGGGA-TCCT--TTGTGGA-----------CGG-TCACTTGG-CTTGATAA-TTA
TCTATGCTTTGTGCGCTGTGAAGCAAGATTTATGGG-ACTTCTGCTTATAACCG------TCTTGTAAC--GAGACAAC-
------T--TT------TTGAACCTTTGACCT--

Mycena_picta_TUR194167
ACCTGGTG-CTGATGCTGGCT-CTC------TCGGGGGCA-TGTGCTCGCATCAT-------CTT-A-TCTATTATCTCT
-TGTGAACCTTTTG-TAGGCTCTGAATCAAGTA---
-CCTTTCGCAGCGCTTT-----------------TAAAGTGCT---CGCGGTCT--------------GAGGG---GTTT
GACGATGCTGCAAA-------GCCGTCTCTCCTTACC---
------------TGCTTGC-------TTCA---------GAG-----------------------TCTATG------TTT
TA----------------------------TATA-CTCTG----TTAAAGTCTT-AGAATGTCT--TMTTATTGA-----
--------CG-AAAGTCAGTA-------AAATC-T-ATACAACTTTCAGCAACGGATCTCTTGGCTCTCCCATCGATGAA
GAACGCAGCGAAATGCGATAACTAATGTGAATTGCAGAATTCAGTGAATCATCGAGTCTTTGAACGCACCTTGCGCCCTT
TGGTATTCCGAAGGGCATGCCTGTTTGAGTGTC-ATTAAATTC-TCAA---------CCTTGAGC--------TTTT---
--GCGAGC-----------TCAAGGC---TTGGAT-GTGA-GG---G---CTTGCTGGC-TTCC----TTCAGTG-GATT
GGTCTGCTCCCTTTAAATGCATTAGTGGGA-TCCT--TTGTGGA-----------GCG-TCACTTGG-TGTGATAA-TTA
TCTAYGCCTC-TTGACC--GAAGCAAGCCTTATGGG-AA-CCTGCTTATAACTG------TCCTTCA----AGGACAAT-
------T--TA------TTGACTATTTGACCT--

Mycena_picta_iNat64022635
ACCTGGTG-CTGATGCTGGCT-CTC------TCGGGGGCA-TGTGCTCGCACCAT-------CTT-A-TCTATTATCTCT
-TGTGAACCTTTTG-TAGGCTCTGAATCAAGTA---
-CCTTTCGCAGCGCTTT-----------------TAAAGTGCT---CGCGGTCT--------------GAGGG---GTTT
GACGATGCTGCAAA-------GCCGTCTCTCCTTACC---
------------TGCTTGC-------TTCA---------GAG-----------------------TCTATG------TTT
TA----------------------------TATA-CTCTG----TTAAAGTCTT-AGAATGTCT--TATTATTGA-----
--------CG-AAAGTCAGTA-------AAATC-T-ATACAACTTTCAGCAACGGATCTCTTGGCTCTCCCATCGATGAA
GAACGCAGCGAAATGCGATAACTAATGTGAATTGCAGAATTCAGTGAATCATCGAGTCTTTGAACGCACCTTGCGCCCTT
TGGTATTCCGAAGGGCATGCCTGTTTGAGTGTC-ATTAAATTC-TCAA---------CCTTGAGC--------TTTT---
--GCGAGC-----------TCAAGGC---TTGGAT-GTGA-GG---G---CTTGCTGGC-TTCC----TTCAGTG-GATT

GGTCTGCTCCCTTTAAATGTATTAGTGGGA-TCCT--TTGTGGA-----------GCG-TCACTTGG-TGTGATAA-TTA
TCTATGCCTC-TTGACC--GAAGCAAGCCTTATGGG-AA-CCTGCTTATAACTG------TCCTTCA----AGGACAAT-
------T--TA------TTGACTATTTGACCT--

Mycena_interrupta_HMJAU43791
CTGTGGTG-CTGT-GCTGGCC-TTTC--------GGGGCA-TGTGCACGCATT----ACTTTTTTCA-TTA--TCTTTTT
-TGTGAACC-AGTG-TAGGCTTTAGAATG---
-TCTCTAGTAATGGG--------------------GAAGCAGG-------TTTGCGCC----------TGTGGT-ACTCG
TGAGCG--AGC---
-------------------------TTTTG---------AAG-----------------------ACTATG-----ATTT
TA----------------------------TATA-AACC---TTTGTATGTCCT-TGAATGT----CTTTATTGG-----
--C----CGC-AAGGCTAGT---------AATC-T-ATACAACTTTCAGCAACGGATCTCTTGGCTCTCCCATCGATGAA
GAACGCAGCGAAATGCGATAAGTAATGTGAATTGCAGAATTCAGTGAATCATCGAATCTTTGAACGCACCTTGCGCCCTT
TGGTATTCCGAAGGGCATGCTTGTTTGAGTGTC-ATTAAATTC-TCAAAAA-----CCTTTGGATT---TAT-TTTT---
--------------------GAAGGCTTTTTGGAT-GTGA-GG---G---TTTGCTGGC-TTCC----TTTAGTGGATTT
GGTCTGCTCCCTTTAAATGCATTAGTGGAT-TCCT--TTGTGAA-----------CGAATCACTTGG-TGTGATAATCTA
TCTACGCCGC-TTGAATCTGAAGCAAAACCTATGGG-AG-CCCGCTCATAACCG------TCTTTTCGG---AGACAAC-
----TAC--CT------TTGACCATTTGACCT--

Mycena_interrupta_HMJAU43849
CTGTGGTG-CTGT-GCTGGCC-TTTC--------GGGGCA-TGTGCACGCATT----ACTTTTTTCA-TTA--TCTTTTT
-TGTGAACC-AGTG-TAGGCTTTAGAATG---
-TCTCTAGTAATGGG--------------------GAAGCAGG-------TTTGCGCC----------TGTGGT-ACTCG
TGAGCG--AGC---
-------------------------TTTTG---------AAG-----------------------ACTATG-----ATTT
TA----------------------------TATA-AACC---TTTGTATGTCCT-TGAATGT----CTTTATTGG-----
--C----CGC-AAGGCTAGT---------AATC-T-ATACAACTTTCAGCAACGGATCTCTTGGCTCTCCCATCGATGAA
GAACGCAGCGAAATGCGATAAGTAATGTGAATTGCAGAATTCAGTGAATCATCGAATCTTTGAACGCACCTTGCGCCCTT
TGGTATTCCGAAGGGCATGCTTGTTTGAGTGTC-ATTAAATTC-TCAAAAA-----CCTTTGGATT---TAT-TTTT---
--------------------GAAGGCTTTTTGGAT-GTGA-GG---G---TTTGCTGGC-TTCC----TTTAGTGGATTT
GGTCTGCTCCCTTTAAATGCATTAGTGGAT-TCCT--TTGTGAA-----------CGAATCACTTGG-TGTGATAATCTA
TCTACGCCGC-TTGAATCTGAAGCAAAACCTATGGG-AG-CCCGCTCATAACCG------TCTTTTCGG---AGACAAC-
----TAC--CT------TTGACCATTTGACCT--

Mycena_pluteoides_HMJAU43765
TCGTGGTG-CTGT-GCTGGCC-TTCC--------GGGGCA-TGTGCACGCACTGC--AATTATTTCA-TTA---TCTTCT
-TGTGAACC-AGTG-TAGGCTTTG--
--TTTGTC----------AGTAGT-GCTAA
GGCGCGAAAGC---
-------------------------AAGGG---------AAG-----------------------TCTATG-----ATTT
TA----------------------------TATA-AACC---TTTGTATGTCTT-TGAATGTCT-ACTTTATTGG-----
--C----CGC-AAGGCTAGTA-------AAACC-T-ATACAACTTTCAGCAACGGATCTCTTGGCTCTCCCATCGATGAA
GAACGCAGCGAAATGCGATAAGTAATGTGAATTGCAGAATTCAGTGAATCATCGAATCTTTGAACGCACCTTGCGCCCTT
TGGTATTCCGAAGGGCATGCTTGTTTGAGTGTC-ATTAAATTC-TCAATGAAACAGGCTTTGTTG-----AC-TGTCTCA
-----------------------------TTGGAT-GTGA-GG---G---TTTGCTGGC-TT---------------TCG
GGTCTGCTCCCTTCAAATGCATTAGTGGAT-TCCT--TTGTGAA-----------CGA-TCACTTGG-TGTGATAATCTA
TCTACGCCGC-TTGACTCTGAAGCAAAACCTATGGG-AG-TCTGCTCACAACCG------TCTTTCGCT---AGACAAC-
------T--TC------TTGACCATTTGACCT--

Mycena_pluteoides_HMJAU43771
TCGTGGTG-CTGT-GCTGGCC-TTCC--------GGGGCA-TGTGCACGCACTGC--AATTATTTCA-TTA---TCTTCT
-TGTGAACC-AGTG-TAGGCTTTG--
--TTTGTC----------AGTAGT-GCTAA
GGCGCGAAAGC---
-------------------------AAGGG---------AAG-----------------------TCTATG-----ATTT
TA----------------------------TATA-AACC---TTTGTATGTCTT-TGAATGTCT-ACTTTATTGG-----
--C----CGC-AAGGCTAGTA-------AAACC-T-ATACAACTTTCAGCAACGGATCTCTTGGCTCTCCCATCGATGAA
GAACGCAGCGAAATGCGATAAGTAATGTGAATTGCAGAATTCAGTGAATCATCGAATCTTTGAACGCACCTTGCGCCCTT
TGGTATTCCGAAGGGCATGCTTGTTTGAGTGTC-ATTAAATTC-TCAATGAAACAGGCTTTGTTG-----AC-TGTCTCA
-----------------------------TTGGAT-GTGA-GG---G---TTTGCTGGC-TT---------------TCG
GGTCTGCTCCCTTCAAATGCATTAGTGGAT-TCCT--TTGTGAA-----------CGA-TCACTTGG-TGTGATAATCTA
TCTACGCCGC-TTGACTCTGAAGCAAAACCTATGGG-AG-TCTGCTCACAACCG------TCTTTCGCT---AGACAAC-
------T--TC------TTGACCATTTGACCT--

Mycena_leaiana_HKAS79900

---GGATGTCTGATGCTGGCCTCTTCG-------GGGGCA-TGTGCTCGTCTCC---GTCTATTT-A-TCT----TCTCT
-TGTGCACCTTTTG-TAGTCTTGAAGTCGAGTTGTA--
-CCCTCCGCAGC-----------------------AATGCGGG----------------------TATGGGGAT-CGCTG
GCCTAGTCAGC---------------TTTCCCTG--
-----------CTTATCGC-------TTCA---------AGG-----------------------TCTATG-----TTTT
CA----------------------------TACA-CACT-----ATAAAGTTAC-AGAATGTC---TATTAAAGA--TTG
TGC--TAGTC-GCAGTCATT--------AAACC-T-ATACAACTTTCAGCAACGGATCTCTTGGCTCTCCCATCGATGAA
GAACGCAGCGAAATGCGATAAGTAATGTGAATTGCAGAATTCAGTGAATCATCGAATCTTTGAACGCACCTTGCGCCCTT
TGGTATTCCGAAGGGCATGCCTGTTTGAGTGTC-ATTAAATTA-TCAA---------CCCTAAAA-----GC--------
---------TTGCGCTGT-TAGTGGC---TTGGAT-GTGG-GG---G--TTTTGCTGGC-TTCC----TTCAGT--GGAC
GGTCTGCTCCCCTTAAATACATTAGTGGGA-TCTTC-TTGTAGA-----------------ACTCAGGTGTGATAA-TTA
TCTACGCCTC------CGTGAAGC-AAGTCAATTGG-AC-CCTGCTCATAACTG------TCCTTTGCG---GGACAAC-
------T--CC------TTTAACTTT--

Mycena_leaiana_HKAS126400

ATCGGATGTCTGATGCTGGCCTCTTCG-------GGGGCA-TGTGCTCGTCTCC---GTCTATTT-A-TCT----TCTCT
-TGTGCACCTTTTG-TAGTCTTGAAGTTGAGTTGTA--
-CCCTCCGCAGC-----------------------AATGCGGG----------------------TATGGGGAT-CGCTG
GCCTAGTCAGC---------------TTTCCCTG--
-----------CTTATCGC-------TTCA---------AGG-----------------------TCTATG-----TTTT
CA----------------------------TACA-CACT-----ATAAAGTTAC-AGAATGTC---TATTAAAGA--TTG
TGC--TAGTT-GCAGTCATT--------AAACC-T-ATACAACTTTCAGCAACGGATCTCTTGGCTCTCCCATCGATGAA
GAACGCAGCGAAATGCGATAAGTAATGTGAATTGCAGAATTCAGTGAATCATCGAATCTTTGAACGCACCTTGCGCCCTT
TGGTATTCCGAAGGGCATGCCTGTTTGAGTGTC-ATTAAATTA-TCAA---------CCCTAATG-----GC--------
---------TTGCGCTATTCAGTGGC---TTGGAT-GTGG-GG---G-TTTTTGCTGGC-TTCC----TTCAGT--GGAC
GGTCTGCTCCCCTTAAATACATTAGTGGGA-TCTTC-TTGTAGA-----------------ACTCCGGTGTGATAA-TTA
TCTACGCCTC------TGTGAAGC-AAGTCTCTTGG-AC-CCTGCTCATAACTG------TCCTTTGCG---GGACAAC-
------T--CT------TTGACATTTTGAC--

Mycena_heteracantha_MK309785

CTTGAGTG-CTGTTGCTGGCG-TGAAA-------GGCGCACTGTGCACGC-------ATTCACTTTATTTTTCAACCCCT
TTGTGCACCTTTTTATAAGTCTTGAGTGA---A-------

-TCTCTCGCATTCAGTTGCG---------------GATGTAGA----GTGTTTT-------AAT---------T-CGTTG
AAT---
---------AAACAATTATC---ACTCTTA-----------------------------------CTCACAAGG--CTTT
TATA--------------------------CATA-CACCTTTTAAACAAGTCAT-AGAATGTTA-CTTTTGTTGA-----
--------CG-CAAGTCAATAG------AAACC-AAATACAACTTTCAGCAACGGATCTCTTGGCTCTCCCATCGATGAA
GAACGCAGCGAAATGCGATAAGTAATGTGAATTGCAGAATTCAGTGAATCATCGAATCTTTGAACGCACCTTGCGCCCTT
TGGTATTCCGAAGGGCATGCCTGTTTGAGTGTC-ATTGAATTC-TCAA---------CCTTATAC--------CTTT---
---ATTGTATTG--------TAAGGC---TTGGAT-GTGA-GG---G-TTATTGCTGGC-TTCC----TTCAATGTTGAT
GGTCAGCTCCCTTGAAATGCATTAGTGGAA-TCCT--TTGTGGA-----------TCG-TCACTGAG-TGTGATAATTTA
TCTACGCTCG-TTGACTCTGAAGC-AATCTTATACGGAA-TCTGCTTATAACAG------TCCTTCAGT--TGGACAAT-
------T--TA-----CTTGACAATTTGACCT--

Mycena_heteracantha_MK309786
CTTGAGTG-CTGTTGCTGGCG-TGAAA-------GGCGCACTGTGCACGC-------ATTCACTTTATTTTTCAACCCCT
TTGTGCACCTTTTTATAAGTCTTGAGTGA---A-------
-TCTCTCGCATTCAGTTGCG---------------GATGTAGA----GTGTTTT-------AAT---------T-CGTTG
AAT---
---------AAACAATTATC---ACTCTTA-----------------------------------CTCACAAGG--CTTT
TATA--------------------------CATA-CACCTTTTAAACAAGTCAT-AGAATGTTA-CTTTTGTTGA-----
--------CG-CAAGTCAATAG------AAACC-AAATACAACTTTCAGCAACGGATCTCTTGGCTCTCCCATCGATGAA
GAACGCAGCGAAATGCGATAAGTAATGTGAATTGCAGAATTCAGTGAATCATCGAATCTTTGAACGCACCTTGCGCCCTT
TGGTATTCCGAAGGGCATGCCTGTTTGAGTGTC-ATTGAATTC-TCAA---------CCTTATAC--------CTTT---
---ATTGTATTG--------TAAGGC---TTGGAT-GTGA-GG---G-TTATTGCTGGC-TTCC----TTCAATGTTGAT
GGTCAGCTCCCTTGAAATGCATTAGTGGAA-TCCT--TTGTGGA-----------TCG-TCACTGAG-TGTGATAATTTA
TCTACGCTCG-TTGACTCTGAAGC-AATCTTATACGGAA-TCTGCTTATAACAG------TCCTTCAGT--TGGACAAT-
------T--TA-----CTTGACAATTTGACCT--

Mycena_alphitophora_NX0491
CCTGGATG-TTGATGCTGGCG-TGAAA--------GCGCA-TGTGCTCGC-------ATTCATATTA-TTTCAAATCCCT
TTGTGCACCTTTC--TAGTCTCGGATTCGAAAAGGA------------------------------------A-------
-CCTCTCACACTCGATCACCTTATTATCTTTGGGTAAACCGAA-------------------GTATTGGATTGT-GCTTG
AGCGCT--------TTCATT-AGCGTCCTCACTAGTCCT---
---------ATGTTCTTGCCTGTGGGTTTG---------AAGCATTTGAGCC----AGAAATGGTCCTACG-----CTTC
TATGTCAGCAGTATGGTTCGGACTAGCTTTTATATCTCTAT---TGTATGTTAA-AGAATGTCATCTTTTGTTGA-----

--------CG-CAAGTCAATG----AAAAAACC-T-ATACAACTTTCAGCAACGGATCTCTTGGCTCTCCCATCGATGAA
GAACGCAGCGAAATGCGATAAGTAATGTGAATTGCAGAATTCAGTGAATCATCGAATCTTTGAACGCACCTTGCACCCTT
TGGTATTCCGAAGGGTATGCCTGTTTGAGTGTC-ATTAAATTC-TCAA---------CCTTACAA--------CTTT---
--TTGTGAGTGG--------TAAGGC---TTGGAT-GTGA-GG---G-TTATTGCTGGC-TTCC----TTCAGTG-CGAT
GGTCAGCTCCCTTTAAATGTATTAGTGGAA-TTC---TTGTGAA-----------TCGTTCACTGAG-TGTGATAA-TTA
TCTACGCTCT---------------AGATTTGCAAC-GG-TCTGCTTATAACCG------TCCTTCAGT--TGGACAAT-
------T--TA------TTGACAATTTGACCT--

Mycena_alphitophora_NX0679
CCTGGATG-TTGATGCTGGCG-TGAAA--------GCGCA-TGTGCTCGC-------ATTCATATTA-TTTCAAATCCCT
TTGTGCACCTTTC--TAGTCTCGGATTCGAAAAGGA------------------------------------A-------
-CCTCTCACACTCGATCACCTTATTATCTTTGGGTAAACCGAA-------------------GTATTGGATTGT-GCTTG
AGCGCT--------TTCATT-AGCGTCCTCACTAGTCCT---
---------ATGTTCTTGCCTGTGGGTTTG---------AAGCATTTGAGCC----AGAAATGGTCCTACG-----CTTC
TATGTCAGCAGTATGGTTCGGACTAGCTTTTATATCTCTAT---TGTATGTTAA-AGAATGTCATCTTTTGTTGA-----
--------CG-CAAGTCAATG----AAAAAACC-T-ATACAACTTTCAGCAACGGATCTCTTGGCTCTCCCATCGATGAA
GAACGCAGCGAAATGCGATAAGTAATGTGAATTGCAGAATTCAGTGAATCATCGAATCTTTGAACGCACCTTGCACCCTT
TGGTATTCCGAAGGGTATGCCTGTTTGAGTGTC-ATTAAATTC-TCAA---------CCTTACAA--------CTTT---
--TTGTGAGTGG--------TAAGGC---TTGGAT-GTGA-GG---G-TTATTGCTGGC-TTCC----TTCAGTG-CGAT
GGTCAGCTCCCTTTAAATGTATTAGTGGAA-TTC---TTGTGAA-----------TCGTTCACTGAG-TGTGATAA-TTA
TCTACGCTCT---------------AGATTTGCAAC-GG-TCTGCTTATAACCG------TCCTTCAGT--TGGACAAT-
------T--TA------TTGACAATTTGACCT--

Mycena_hygrophoroides_HMJAU43417
CTTGGGTG-TTGATGCTGGCG-TGAAA--------GCGCA-TGTGCTCGC-------ATTCATATTA-TTTCAAATCCCT
TTGTGCACCTTTC--TAGTCTCGAATTCGAAAAGGA------------------------------------A-------
-CCTCTCACACTCGATCACC-TATTATCTCTGGGTAAACCGGA-------------------GTATTGGATTGT-GCTTG
TGCGCT--------TTTATT-AGCGTCCTCACTCGTCCT---
---------ATGTTCTTGCCTGTGGGTTTG---------AAGCATTTGAGCC----AGTAATGGTCCTACG-----CTTC
TATGTCAGCAGTATGGTTCGGACTAGCTTTTATATCTCTAT---TGTATGTTAA-AGAATGTCAT-CTTTGTTGA-----
--------CG-AAAGTCAACG-----AAAAACC-T-ATACAACTTTCAGCAACGGATCTCTTGGCTCTCCCATCGATGAA
GAACGCAGCGAAATGCGATAAGTAATGTGAATTGCAGAATTCAGTGAATCATCGAATCTTTGAACGCACCTTGCACCCTT
TGGTATTCCGAAGGGTATGCCTGTTTGAGTGTC-ATTAAATTC-TCAA---------CCTTACAA--------CTTT---
--TTGTGAGTCG--------TAAGGC---TTGGAT-GTGA-GG---G-TTATTGCTGGC-TTCC----TTCAGTG-TGAT

GGTCAGCTCCCTTTAAATGTATTAGTGGAA-TTC---TTGTGAA-----------TCGTTCACTGAG-TGTGATAA-TTA
TCTACGCTCT---------------AGATTTGCAAC-GG-TCTGCTTATAACCG------TCCTTAAGT--TGGACAAT-
------T--TA------TTGACAATTTGACCT--

Mycena_hygrophoroides_HMJAU43421
CTTGGGTG-TTGATGCTGGCG-TGAAA--------GCGCA-TGTGCTCGC-------ATTCATATTA-TTTCAAATCCCT
TTGTGCACCTTTC--TAGTCTCGAATTCGAAAAGGA------------------------------------A-------
-CCTCTCACACTCGATCACC-TATTATCTCTGGGTAAACCGGA-------------------GTATTGGATTGT-GCTTG
TGCGCT--------TTTATT-AGCGTCCTCACTCGTCCT---
---------ATGTTCTTGCCTGTGGGTTTG---------AAGCATTTGAGCC----AGTAATGGTCCTACG-----CTTC
TATGTCAGCAGTATGGTTCGGACTAGCTTTTATATCTCTAT---TGTATGTTAA-AGAATGTCAT-CTTTGTTGA-----
--------CG-AAAGTCAACG-----AAAAACC-T-ATACAACTTTCAGCAACGGATCTCTTGGCTCTCCCATCGATGAA
GAACGCAGCGAAATGCGATAAGTAATGTGAATTGCAGAATTCAGTGAATCATCGAATCTTTGAACGCACCTTGCACCCTT
TGGTATTCCGAAGGGTATGCCTGTTTGAGTGTC-ATTAAATTC-TCAA---------CCTTACAA--------CTTT---
--TTGTGAGTCG--------TAAGGC---TTGGAT-GTGA-GG---G-TTATTGCTGGC-TTCC----TTCAGTG-TGAT
GGTCAGCTCCCTTTAAATGTATTAGTGGAA-TTC---TTGTGAA-----------TCGTTCACTGAG-TGTGATAA-TTA
TCTACGCTCT---------------AGATTTGCAAC-GG-TCTGCTTATAACCG------TCCTTAAGT--TGGACAAT-
------T--TA------TTGACAATTTGACCT--

Mycena_miscanthi_HMJAU43573
CTGGGTGT-TTGATGCTGGCG-CGAAA--------GTGCA-TGTGCTCAC-------ATTCATCTTA-TTTCAAATCCCT
TTGTGCACCTTTC--TAGTCTCGAATTCGAAAAGGA------------------------------------A-------
-CCTCTCACACTCGATCACC-TA-CATCTCTGGGTAAACCGGA-------------------GCGTTGGATGT--GCTTG
TGCGCT--------TTCATCAAGCGTCCTCACTAGTCCT---
---------ATGTTCTTGCCTGTGGGTTTG---------AAGTATTTGAGCCGTGTAACAACGGTCCTACT-----CTTC
TATGTCAGCAGTATGGTTCGGACTAGCTTTTATATCTCTGT---TGTATGTTAA-AGAATGTCATCTTTTGTTGA-----
--------CG-AAAGTCAATGA--AAAAAAACC-T-ATACAACTTTCAGCAACGGATCTCTTGGCTCTCCCATCGATGAA
GAACGCAGCGAAATGCGATAAGTAATGTGAATTGCAGAATTCAGTGAATCATCGAATCTTTGAACGCACCTTGCACCCTT
TGGTATTCCGAAGGGTATGCCTGTTTGAGTGTC-ATTAAATTC-TCAA---------CCTTACAA--------CTTT---
--TTGTGAGCGG--------TAAGGC---TTGGAT-GTGAGGG---G-TTATTGCTGGC-TTCC----TTCAGTG-TGAT
GGTCAGCTCCCTTTAAATGCATTAGTGGAA-TTC---TTGTGAA-----------TCGTTCACTGAG-TGTGATAA-TTA
TCTACGCTCT---------------AGATTTGCAA--AG-TCTGCTTATAACTG------TCCTTGAGT--TGGACAAT-
------T--TA------TTGACAATTTGACCT--

Mycena_miscanthi_HMJAU43582
CTGGGTGT-TTGATGCTGGCG-CGAAA--------GTGCA-TGTGCTCAC-------ATTCATCTTA-TTTCAAATCCCT
TTGTGCACCTTTC--TAGTCTCGAATTCGAAAAGGA------------------------------------A-------
-CCTCTCACACTCGATCACC-TA-CATCTCTGGGTAAACCGGA-------------------GCGTTGGATGT--GCTTG
TGCGCT--------TTCATCAAGCGTCCTCACTAGTCCT---
---------ATGTTCTTGCCTGTGGGTTTG---------AAGTATTTGAGCCGTGTAACAACGGTCCTACT-----CTTC
TATGTCAGCAGTATGGTTCGGACTAGCTTTTATATCTCTGT---TGTATGTTAA-AGAATGTCATCTTTTGTTGA-----
--------CG-AAAGTCAATGA--AAAAAAACC-T-ATACAACTTTCAGCAACGGATCTCTTGGCTCTCCCATCGATGAA
GAACGCAGCGAAATGCGATAAGTAATGTGAATTGCAGAATTCAGTGAATCATCGAATCTTTGAACGCACCTTGCACCCTT
TGGTATTCCGAAGGGTATGCCTGTTTGAGTGTC-ATTAAATTC-TCAA---------CCTTACAA--------CTTT---
--TTGTGAGCGG--------TAAGGC---TTGGAT-GTGAGGG---G-TTATTGCTGGC-TTCC----TTCAGTG-TGAT
GGTCAGCTCCCTTTAAATGCATTAGTGGAA-TTC---TTGTGAA-----------TCGTTCACTGAG-TGTGATAA-TTA
TCTACGCTCT---------------AGATTTGCAA--AG-TCTGCTTATAACTG------TCCTTGAGT--TGGACAAT-
------T--TA------TTGACAATTTGACCT--

Mycena_griseotincta_HMJAU43805
CTGGGTGT-TTGATGCTGGCG-TGAAA--------GCGCA-TGTGCTCGC-------ATTCATCTTA-TTTCAAATCCCT
TTGTGCACCTTTC--TAGTCTCGAATTCGAAAAGGA------------------------------------A-------
-CCTCTCACACTTGATCACC-TA-CATCTCTGGGTAAGCCGGA-------------------GCGTTGGATGT--GCTTG
TGCGCT--------TTCATT-AGCGTCCTCACTAGTCCT---
---------AAGTTCTTGCCTGTGGGTTTG---------AAGTATTTGAGCCGTGTAACAACGGTCCTACT-----CTTC
TATGTCAGCAGTATGGTTCGGACTAGCTTTTATATCTCTAT---TGTATGTTAA-AGAATGTCATCTTTTGTTGA-----
--------CG-AAAGTCAATG---AAAAAAACC-T-ATACAACTTTCAGCAACGGATCTCTTGGCTCTCCCATCGATGAA
GAACGCAGCGAAATGCGATAAGTAATGTGAATTGCAGAATTCAGTGAATCATCGAATCTTTGAACGCACCTTGCACCCTT
TGGTATTCCGAAGGGTATGCCTGTTTGAGTGTC-ATTAAATTC-TCAA---------CCTTACAA--------CTTT---
--TTGTGAGTGG--------TAAGGC---TTGGAT-GTGA-GG---G-TTATTGCTGGC-TTCC----TTCAGTG-CGAT
GGTCAGCTCCCTTTAAATGTATTAGTGGAA-TTC---TTGTGAA-----------TCGTTCACTGAG-TGTGATAA-TTA
TCTACGCTCT---------------AGATTTGCAA--AG-TCTGCTTATAACCG------TCCTTCAGT--TGGACAAT-
------T--TC------TTGACAATTTGACCT--

Mycena_griseotincta_HMJAU43800
CTGGGTGT-TTGATGCTGGCG-TGAAA--------GCGCA-TGTGCTCGC-------ATTCATCTTA-TTTCAAATCCCT
TTGTGCACCTTTC--TAGTCTCGAATTCGAAAAGGA------------------------------------A-------
-CCTCTCACACTTGATCACC-TA-CATCTCTGGGTAAGCCGGA-------------------GCGTTGGATGT--GCTTG
TGCGCT--------TTCATT-AGCGTCCTCACTAGTCCT---
---------AAGTTCTTGCCTGTGGGTTTG---------AAGTATTTGAGCCGTGTAACAACGGTCCTACT-----CTTC
TATGTCAGCAGTATGGTTCGGACTAGCTTTTATATCTCTAT---TGTATGTTAA-AGAATGTCATCTTTTGTTGA-----
--------CG-AAAGTCAATG---AAAAAAACC-T-ATACAACTTTCAGCAACGGATCTCTTGGCTCTCCCATCGATGAA
GAACGCAGCGAAATGCGATAAGTAATGTGAATTGCAGAATTCAGTGAATCATCGAATCTTTGAACGCACCTTGCACCCTT
TGGTATTCCGAAGGGTATGCCTGTTTGAGTGTC-ATTAAATTC-TCAA---------CCTTACAA--------CTTT---
--TTGTGAGTGG--------TAAGGC---TTGGAT-GTGA-GG---G-TTATTGCTGGC-TTCC----TTCAGTG-CGAT
GGTCAGCTCCCTTTAAATGTATTAGTGGAA-TTC---TTGTGAA-----------TCGTTCACTGAG-TGTGATAA-TTA
TCTACGCTCT---------------AGATTTGCAA--AG-TCTGCTTATAACCG------TCCTTCAGT--TGGACAAT-
------T--TC------TTGACAATTTGACCT--

Mycena_amygdalina_MT497545
ACCAGGTT-TTGATGCTGGCC-CTT-----CAC-CGGGCA-TGTGCTCGCTCCGA--ATCTATTT-A-TCT----TCTCT
-TGTGCACCTCTTG-TAGTCTTTGAAA---------------------------------AACGCGAACC----------
---TCTCCCATC-----------------------AATGCGGA----------------------CTGGGGGG---CTGG
GGCCCTAAAAC-------------CCCTTCCCCGGCT---
------------TGCTTTC-------TTTC---------ACG-----------------------GCTATG-----TTTT
CC----------------------------TAAA-CACT-----TTTTAGTTAC-AGAATGTC---TTTTAAAGA--TTG
----TCGCTC-TCGCCCACTC----ATAAAACC-T-ATACAACTTTCACTCCCAGATCTCTTGACTCTCCCATCAATGAA
TAACGCATGTAAATGGGATAATTCATGGGAATAGCAAAACTCAGAGAATCCTCTAATCCCTTAGCTCTTCCTGAGGCCTT
TGGTATTCCAAAGGGCATGCCTGCTTGAGTGTG-ATTAATTTC-TCAG---------CCTTGCTC-----GC-TTTGCAA
G-------------------TGTTGC---TTGGAT-GTGT-GG---G---CTTGCTGGC-TTCC----TTCCTT--GGAT
GGTCTGCTTCCGTTTTATGGATTACCGGGA-TCTC--TTGTGGAACT------------TCATCTGT-CGCGATAA-TTA
TCTATGCCACGTCTACCGTGAAGC--GCATTATGGG-AC-CCTGAGTACA-----CA---TCCCCGCTG--GGGACAATA
------T--CT------CTGACAATTTGACAA--

Mycena_amygdalina_HMJAU43700
ACCAGGTT-TTGATGCTGGCC-CTT-----CAC-CGGGCA-TGTGCTCGCTCCGA--ATCTATTT-A-TCT----TCTCT
-TGTGCACCTCTTG-TAGTCTTTGAAA---------------------------------AACGCGAACC----------

---TCTCCCATC-----------------------AATGCGGA----------------------CTGGGGGG---CTGG
GGCCCTAAAC--------------CCCTTCCCCGGCT---
------------TGCTTTC-------TTTC---------ACG-----------------------GCTATG-----TTTT
CC----------------------------TAAA-CACT-----TTTTAGTTAC-AGAATGTC---TTTTAAAGA--TTG
----TCGCTC-TCGCCCACTC----ATAAAACC-T-ATACAACTTTCACTCCCAGATCTCTTGACTCTCCCATCAATGAA
TAACGCATGTAAATGGGATAATTCATGGGAATAGCAAAACTCAGAGAATCCTCTAATCCCTTAGCTCTTCCTGAGGCCTT
TGGTATTCCAAAGGGCATGCCTGCTTGAGTGTG-ATTAATTTC-TCAG---------CCTTGCTC-----GC-TTTGCAA
G-------------------TGTTGC---TTGGAT-GTGT-GG---G---CTTGCTGGC-TTCC----TTCCTT--GGAT
GGTCTGCTTCCGTTTTATGGATTACCGGGA-TCTC--TTGTGGAACT------------TCATCTGT-CGCGATAA-TTA
TCTATGCCACGTCTACCGTGAAGC--GCATTATGGG-AC-CCTGAGTACA-----CA---TCCCCGCTG--GGGACAATA
------T--CT------CTGACAATTTGACAA--

Xeromphalina_campanella_KM024575
TCGGGGAA-CTGTTGCTGGCC-CGT-----CAC-AGGGCA-TGTGCACGCTCCCC-AATTTTCCT---TCT----CCACC
-TGTGCACCCTTTG-TAGACTTTGGAGGA---
-CCTCTCGAGGAAACTC-----------------GGATGTGGG----------------------GTCGCTGA-------
--------------TTCACT-TCGGCTTCCCTTG--
-----------C------------GCTTCC---------AGG-----------------------TCTATGCA---TTTT
TA----------------------------CACA-CACTT----TAAATGTCTC-AGAATGTC---TTTCATGGC-----
----TTGCCG-TAAAAAAGCAAAGCTTTAAACT-TAATACAACTTTCAACAACGGATCTCTTGGCTCTCGCATCGATGAA
GAACGCAGCGAAATGCGATAAGTAATGTGAATTGCAGAATTCAGTGAATCATCGAATCTTTGAACGCACCTTGCGCTCCT
TGGTATTCCGAGGAGCATGCCTGTTTGAGTGTC-ATTAAATTC-TCAA---------CCTCTCCA-----GC-TTTT---
---------TTGGCTGG--ACGAGGA---TTGGAT-GTGGGGG---G---TGTGCTGGC-TTCGTC----------AAGA
GGTCGGCTCCCCTTAAATGCATTAGCGGGA-ACGTC-TTGCGGA-----------CCG-TCACTTGG-TGTGATAA-CTA
TCTACGCCTC-TTGACC--------GATGCTCTTGT----TCTGCTTCTAACCG------TCCATTGACT-TGGACAGC-
------T--CT-----TTTGACAATTTGACCT--

Xeromphalina_campanella_KP835678
TCGGGGAA-CTGTTGCTGGCC-CGT-----CAC-AGGGCA-TGTGCACGCTCCCC-AATTTTCCT---TCT----CCACC
-TGTGCACCCTTTG-TAGACTTTGGAGGA---
-CCTCTCGAGGAAACTC-----------------GGATGTGGG----------------------GTCGCTGA-------
--------------TTCACT-TCAGCTTCCCTTG--
-----------C------------GCTTCC---------AGG-----------------------TCTATGCA---TTTT
TA----------------------------CACA-CACTT----TAAATGTCTT-AGAATGTC---TTTCATGGC-----

----TTGCCG-TAAAAAAGCAAAGCTTTAAACT-TAATACAACTTTCAACAACGGATCTCTTGGCTCTCGCATCGATGAA
GAACGCAGCGAAATGCGATAAGTAATGTGAATTGCAGAATTCAGTGAATCATCGAATCTTTGAACGCACCTTGCGCTCCT
TGGTATTCCGAGGAGCATGCCTGTTTGAGTGTC-ATTAAATTC-TCAA---------CCTCTCCA-----GC--TTT---
---------TTGGCTGG--ACGAGGA---TTGGAT-GTGG-GG---G---TGTGCTGGC-TTCGTC----------AAGA
GGTGGGCTCCCCTTAAATGCATTAGCGGGA-ACGTC-TTGCGGA-----------CCG-TCACTTGG-TGTGATAA-CTA
TCTACGCCTC-ATGACC--------GATGCTCTTGT----TCTGCTTCTAACCG------TCCATTGACT-TGGACAGC-
------T--CT-----CTTGACAATTTGACCT--

Mycena_speirea_LT671445
ATGGAGTG-TTGTAGCTGGCT-CTTC--------GGAGCA-TGTGCACGCACTTCAAATTCATCT---TTA----CCACC
-TGTGCACTTTTG--TAGACTGTGATA---
---ACTCTCAATGGTTTACTCCGT---------------TGGA----------------------TTGAAGGA-------
-------------------CATGCGTCTTAGCTGTCTTTC--
-----------------------GACTTCA---------CAG-----------------------TCTATGTCT--CATC
TA---------------------------CTATA------------AAAAGTCT-AGAATGTA---ACTTGTGGGTCTT-
-----------TTGACCTATA-------AAACT-TAATACAACTTTCAACAACGGATCTCTTGGCTCTCGCATCGATGAA
GAACGCAGCGAAATGCGATAAGTAATGTGAATTGCAGAATTCAGTGAATCATCGAATCTTTGAACGCACCTTGCACTCCT
TGGTATTCCGAGGAGTATGCCTGTTTGAGTGTC-ATTAAATTC-TCAAA--------CCTCTTTG-----AC-TTTA---
---------TTGACAA---AGAGGCT---TTGGAT-GTGG-GG---G----TTGCTGGC-TTTC------------ACG-
AGTCAGCTCTCCTTAAATACATTAGTGGTT-ACTG--TTCTCCA---------------CTTCCTGG-TGTGATAA--TA
TCTACGCCTA----------GTGGAGATGTTAAGTGTAG-CTGCTCTCTAACCG------TCCATGCAATGTGGACAAA-
----------------TTGAACCCTTTGACCT--

FLF1269
AATGGAGG-CTGTCGCTGACT-CTT----TCACGAGAGTA-TGTGCACGC-CTTCAAATTCATCT---TTC----ACCAC
-TGTGAACTGTAG--TAGACTATGATA---
---TCTCTCAATGGCTCGCCAT-----------------TGGA----------------------TTGAAGGA-------
-------------------C-ATGCGCTTAGCTGTCTTTC--
---------AA--------------TTTCG---------TAG-----------------------TCTCCCCCTTATCTT
TA----------------------------AAAA----------CTAAAGTCCT-AGAATGTC---TTTTGTGGGTGTA-
-----------AAAACCTAT--------AAACT-T-ATACAACTTTCAACAACGGATCTCTTGGCTCTCGCATCGATGAA
GAACGCAGCGAAATGCGATAAGTAATGTGAATTGCAGAATTCAGTGAATCATCGAATCTTTGAACGCACCTTGCACTCCT
TGGTATTCCGAGGAGTATGCCTGTTTGAGTGTC-ATTAAATTC-TCAAA--------CTTCATAG-----GC-TTTA---
---------TTGACTA---TGAGGCT---TTGGAT-GTGG-GG---G----TTGCCGGCTTTTC------------ACGA

AGTCAGCTCTCCTTAAATACATTAGTGGTT-ACTG--TTCTCCA---------------CTTCTTGG-TGTGATAA-TTA
TCTACGCCTA----------GTGAAGAAGTAAAGTGTAG-CTGCTTTCTAACCG------TCCATTTACT-TGGACAAC-
----------------TTGAACCATTTGACCT-GCCTTCACCCTTGGCGTCCGTCAGTTGATCGTCGCCGTCAACAAGAT
GGATACCACCAAGGTTAGCAAATTTACGTCAA-ATT---------------TTCCTC---ATGT------CCTAAT--CA
CCGCGTTTCACAGTGGAGTGAAGATCGCTTCAACGAAATTATCAAGGAAACGTCGCAGTTCATCAAAAAGGTCGGATACA
ACCCCAAGGCCGTCGCTTTTGTGCCAATATCTGGATGGCACGG-TGACAATAT--GTTGGAAGAATCC------------
---GAACACATGACCTGG--TCAAGG
GCTGGACCAAGGAGAC---CAAGGGTGGT--GTCGTCAAGGG-AAAACTCTCCTC---GATGCCATCG------------
-ATGCCATCGAACCTCCCGTCCGCCC-TCTGAC-
FLF1582
AATGGAGG-CTGTCGCTGACT-CTT----TCACGAGAGTA-TGTGCACGC-CTTCAAATTCATCT---TTC----ACCAC
-TGTGAACTGTAG--TAGACTATGATA---
---TCTCTCAATGGCTCGCCAT-----------------TGGA----------------------TTGAAGGA-------
-------------------C-ATGCGCTTAGCTGTCTTTC--
---------AA--------------TTTCG---------TAG-----------------------TCTCCCCCTTATCTT
TA----------------------------AAAA----------CTAAAGTCCT-AGAATGTC---TTTTGTGGGTGTA-
-----------AAAACCTAT--------AAACT-T-ATACAACTTTCAACAACGGATCTCTTGGCTCTCGCATCGATGAA
GAACGCAGCGAAATGCGATAAGTAATGTGAATTGCAGAATTCAGTGAATCATCGAATCTTTGAACGCACCTTGCACTCCT
TGGTATTCCGAGGAGTATGCCTGTTTGAGTGTC-ATTAAATTC-TCAAA--------CCTCATAG-----GC-TTTA---
---------TTGACTA---TGAGGCT---TTGGAT-GTGG-GG---G----TTGCCGGCTTTTC------------ACGA
AGTCAGCTCTCCTTAAATACATTAGTGGTT-ACTG--TTCTCCA---------------CTTCTTGG-TGTGATAA-TTA
TCTACGCCTA----------GTGAAGAAGTAAAGTGTAG-CTGCTTTCTAACCG------TCCATTTACT-TGGACAAC-
----------------TTGAACCATTTGACCT--

Mycena_auricoma_AFTOL_ID_1341
TGTTAGAG-TTGA-GCTGACTTCTC---------GGAGTA-TGTGCTCGCTCTAT-CACTTTCCT-C-TCT---TTCCCC
-TGTGCACATTTG--TAGGCTACGATAACTATCAGA-----------------------T------------GG------
TTCCTTCGGACCAACTGCTT--G-AGGTGCTTGCCATTGCGGC-------------------------------------
-------------------------ATGCCCTTG--
-----------C-------------CATCG---------TAG-----------------------TCTATG-----TTTA
CAAGC------------------------TCATAACAGTCA--------------AGAATGTA---TTTTGTTGGTCT--
-----------CAGACCGATA------CAAACC-TTATACAACTTTCAGCAACGGATCTCTTGGCTCTCGCATCGATGAA
GAACGCAGCGAAATGCGATAAGTAATGTGAATTGCAGAATTCAGTGAATCATCGAATCTTTGAACGCACCTTGCGCCCTT
TGGTATTCCGAAGGGCACGCCTGTTCGAGTGTC-ATTAAATTC-TCAAA--------CCTTCAAG--------TTAGTTA
--ATCTGACTTT--------CAGGCT---TTGGAT-TTGG-GA---G----TTGCTGGTCTTTC-------------GATA
GTTCAGCTCTTCTTGAATGCATCAGCGGAT-TCTA------------CTCGGTCCCCTTTCATCTGG-TGTGATAG-TTA
TCTACGCCTT----------TGAAATGACCTCCTGT-AG-CTGCTTTCTAATAG------TCTCTTCTA--GAGACAGC-
---------------TCTTCAATGT---

Mycena_auricoma_HKAS126433

TGTTAGAG-TTGA-GCTGACTTCTC---------GGAGTA-TGTGCTCGCTCTAT-CACTTTCCT-C-TCT---TTCCCC
-TGTGCACATTTG--TAGGCTACGATAACTATCAGA-----------------------T------------GG------
TTCCTTCGGACCAACTGCTT--G-AGGTGCTTGCCATTGCGCA-------------------------------------
--------------------------TGCCCTTG--
-----------C-------------CATCG---------TAG-----------------------TCTATG-----TTTA
CAAAC------------------------TCATAACAGTCA--------------AGAATGTA---TTTTGTTGGTCT--
-----------CAGACCGATA------CAAAAC-TTATACAACTTTCAGCAACGGATCTCTTGGCTCTCGCATCGATGAA
GAACGCAGCGAAATGCGATAAGTAATGTGAATTGCAGAATTCAGTGAATCATCGAATCTTTGAACGCACCTTGCGCCCTT
TGGTATTCCGAAGGGCACGCCTGTTCGAGTGTC-ATTAAATTC-TCAAA--------CCTTTAAG--------TTAGTTA
--ATCTGACTTT--------CAGGCT---TTGGAT-TTGG-GA---G----TTGCTGGTCTTTC------------GATA
GTTCAGCTCTTCTTGAATGCATCAGCGGAT-TCTA------------CTCGGTCCCCTTTCATCTGG-TGTGATAG--TA
TCTACGCCTT----------TGAAATGACCTCCTGT-AG-CTGCTTTCTAATAG------TCTCTTCTA--GAGACAGC-
---------------TCTTCAATGTTTGACCT--

Mycena_floridula_MK309770

ATTGG-AG-TTGTAGCTGACTGCTTCG-------GCAGTA-TGTGCTCGCTTC----ATTCTTCT---TTT----CCACC
-TGTGCACCTTTTG-TAGGCTATGTTAAC---
-TCTCGTGCGTTGGGCTTTT----------------------------AGTCCTTCGTCGG--ATTTCAAGAGG---CTTG
GTTGTCCTTTCAT-TA-GGT-TTCCATCCGCTTGTTC---
-------------------------TTTCA---------TAGT----------------------TCTATG------TCT
TA----------------------------TACA-CTCT-----TTAATAACCT-AGAATGTC---TTTTGTGGGTCTTG
TAC---------------------CTATAAACT-T-ATACAACTTTTAACAACGGATCTCTTGGCTCTCGCATCGATGAA
GAACGCAGCGAAATGCGATAAGTAATGTGAATTGCAGAATTCAGTGAATCATCGAATCTTTGAACGCACCTTGCGCCCTT
TGGTATTCCGAAGGGCATGCCTGTTTGAGTGTC-ATTAAATTC-TCAAACC------TCTTGCTA-----GT-TTAT---
--TCTAGTTTCG---------AGGCT---TTGGAT-ATGG-GA---G-TCTTTGCTGG--AACCTA--TTCATT--TAGG
GTTCAGCTCTCCTTAAATGCATTAGCGGTTAACTT----------------GATCCCCTTTCATCGG-TGTGATAA-TTA
TCTACGCCTTTCGAAATGATCAGCAGAATGT------TA-ATAGCTTCTAACCAG-----TCCCTAGTC---GGACAAC-
------T--TT-----TATGACCATTTGACCT--

Mycena_floridula_MK309771
ATTGG-AG-TTGTAGCTGACTGCTTCG-------GCAGTA-TGTGCTCGCTTC----ATTCTTCT---TTT----CCACC
-TGTGCACCTTTTG-TAGGCTATGTTAAC---
-TCTCGTGCGTTGGGCTTTT---------------------------AGTCCTTCGTCGG--ATTTCAAGAGG---CTTG
GTTGTCCTTTCAT-TA-GGT-TTCCATCCGCTTGTTC---
-------------------------TTTCA---------TAGT---------------------TCTATG-------TCT
TA----------------------------TACA-CTCT-----TCAATAACCT-AGAATGTC---TTTTGTGGGTCTTG
TAC---------------------CTATAAACT-T-ATACAACTTTTAACAACGGATCTCTTGGCTCTCGCATCGATGAA
GAACGCAGCGAAATGCGATAAGTAATGTGAATTGCAGAATTCAGTGAATCATCGAATCTTTGAACGCACCTTGCGCCCTT
TGGTATTCCGAAGGGCATGCCTGTTTGAGTGTC-ATTAAATTC-TCAAACC------TCTTGCTA-----GT-TTAT---
--TCTAGTTTCG---------AGGCT---TTGGAT-ATGG-GA---G-TCTTTGCTGG--AACCTA--TTCATT--TAGG
GTTCAGCTCTCCTTAAATGCATTAGCGGTTAACTT----------------GATCCCCTTTCATTGG-TGTGATAA-TTA
TCTACGCCTTTCGAAATGATCAGCAGAATGT------TA-ATAGCTTCTAACCAG-----TCCCTAGTC---GGACAAC-
------T--TT-----TATGACCATTTGACCT--

Mycena_flavoalba_CBS_59_50
ATTGGAAG-TTGTAGCTGACTGCTTCG-------GCAGTA-TGTGCTCGCTTC----ATTCTTCT---TTT----CCACC
-TGTGCACCTTTTG-TAGGCTATGTTAAC---
-TCTCGTGCGTTGGGCTTTT-------------------------CAAGTCCTTCGCCGG--ATTTCAAGAGG---CTTG
GCTTCCCTTTCAT-CAGGGT-TTCCATCCGCTTGTTC---
--------------------------TTCA---------TAGT---------------------TCTATG-------TCT
TA----------------------------TACA-CTCT-----TTAA-AACCT-AGAATGTC---TTTTGTGGGTCTTG
TAC---------------------CTATAAACT-TAATACAACTTTTAACAACGGATCTCTTGGCTCTCGCATCGATGAA
GAACGCAGCGAAATGCGATAAGTAATGTGAATTGCAGAATTCAGTGAATCATCGAATCTTTGAACGCACCTTGCGCCCTT
TGGTATTCCGAAGGGCATGCCTGTTTGAGTGTC-ATTAAATTC-TCAAACC------TCTTGCTA-----GT-TTAT---
--TCTAGTCTCG---------AGGCT---TTGGAT-ATGG-GA---G-TCTTTGCTGG--AACCTA--TTCATT--TAGG
ATTCAGCTCTCCTTAAATGTATTAGCGGTTAACTT----------------GATCCCCTTTCATTGG-TGTGATAA-TTA
TCTACGCCTT-GAAAATGATCAGCAGAATGT------TA-ATGGCTTCTAATCAG-----TCCCTAGTC---GGACAAC-
------T--TT-----CATTGACA-TTGACCT--

Mycena_flavoalba_CBS_258_53
ATTGG-AG-TTGTAGCTGACTGCTTCG-------GCAGTA-TGTGCTCGCTTC----ATTCTTCT---TTT----CCACC
-TGTGCACCTTTTG-TAGGCTATGTTAAC---

-TCTCGTGCGTTGGGCTTTT-------------------------TAAGTCCTTCGCCGG--ATTTCAAGAGG---CTTG
GCTTCCCTTTTAT-TAGGGT-TTCCATCTGCTTGTTC---
--------------------------TTCA---------TAGT----------------------TCTATG------TCT
TA----------------------------TACA-CTCT-----TTAA-AACCT-AGAATGTC---TTTTGTGGGTCTTG
TAC---------------------CTATAAACT-TAATACAACTTTTAACAACGGATCTCTTGGCTCTCGCATCGATGAA
GAACGCAGCGAAATGCGATAAGTAATGTGAATTGCAGAATTCAGTGAATCATCGAATCTTTGAACGCACCTTGCGCCCTT
TGGTATTCCGAAGGGCATGCCTGTTTGAGTGTC-ATTAAATTC-TCAAACC------TCTTGCTA-----GT-TTAT---
--TCTAGTCTCG---------AGGCT---TTGGAT-ATGG-GA---G-TCTTTGCTGG--AACCTA--TTCATT--TAGG
GTTCAGCTCTCCTTAAATGTATTAGCGGTTAACTT----------------GATCCCCTTTCATTGG-TGTGATAA-TTA
TCTACGCCTT-GAAAATGATCAGCAGAATGT------TA-ATAGCTTCTAATCAG-----TCCCTAGTC---GGACAAC-
------T--TT-----CATTGACA-TTGACCT--

FLF1713

ATTGG-AG-TTGTAGCTGACTGCTTCG-------GCAGTA-TGTGCTCGCTTC----ATTCTTCT---TTT----CCACC
-TGTGCACCTTTTG-TAGGCTATGTTAAC---
-TCTCGTGCGTTGGGCTTTT-----------------------TTTAAGTCCTTCGCCGG--ATTTCAAGAGG---CTTG
GATTTCCTTTCAT-TAGGTT-TTCCATCCGCTTGTTC---
--------------------------TTCA---------TAGT----------------------TCTATG------TCT
TA----------------------------TACA-CTCT-----TTAA--ACCT-AGAATGTC---TTTTGTGGGTCTTG
TAC---------------------CTATAAACT-T-ATACAACTTTTAACAACGGATCTCTTGGCTCTCGCATCGATGAA
GAACGCAGCGAAATGCGATAAGTAATGTGAATTGCAGAATTCAGTGAATCATCGAATCTTTGAACGCACCTTGCGCCCTT
TGGTATTCCGAAGGGCATGCCTGTTTGAGTGTC-ATTAAATTC-TCAAACC------TCTTGCTA-----GT-TTAT---
--TCTAGTCTCG---------AGGCT---TTGGATAATGG-GA---G-TCTTTGCTGG--AACC--------TT--TAGG
GTTCAGCTCTCCTTAAATGCATTAGCGGTTAACTT----------------GATCCCCTTTCATTGG-TGTGATAA-TTA
TCTACGCCTTTCGAAATGATCAGCAGAATGT------TA-ATAGCTTCTAATCAG-----TCCCTAGTC---GGACAAC-
------T--TT-----ATTGACCATTTGACCT-GCCTTCACCCTCGGTGTCCGCCAGCTCATCGTCGCTGTCAACAAGAT
GGACACTACCAAGGTTCG----TTTTCTTTAT-TTC---------------TTTTCCGAAAAAG------CTCAAG--CT
GATTCCCCAACAGTGGAGCGAAGATCGTTTCAACGAAATTATCAAGGAGACCTCGACTTTCATCAAAAAGGTTGGCTACA
ACCCCAAAGCTGTTGCCTTTGTGCCCATTCTGGATGGCACGG-TGACAACAT--GTTGGAGGAGTCCCCCAAGTG----
---------CGTCTTTTTTTCAATCGTGACACTAACATTCGCTAATT------ACTTTACAGCATGCCATGG-TTCAAGG
GCTGGACCAAGGAGAC---CAAGGCTGGT--GTCTCCAAGGGAAAGACCCTTCTC---GAGGCCATCG------------
-ATGCTATCGAGCCCCCTGTTCGTCCATCAGAC-

MSX1713

ATTGG-AG-TTGTAGCTGACTGCTTCG-------GCAGTA-TGTGCTCGCTTC----ATTCTTCT---TTT----CCACC
-TGTGCACCTTTTG-TAGGCTATGTTAAC---
-TCTCGTGCGTTGGGCTTTT-----------------------TTTAAGTCCTTCGCCGG--ATTTCAAGAGG---CTTG
GATTTCCTTTCAT-TAGGTT-TTCCATCCGCTTGTTC---
--------------------------TTCA---------TAGT----------------------TCTATG------TCT
TA----------------------------TACA-CTCT-----TTAA--ACCT-AGAATGTC---TTTTGTGGGTCTTG

TAC---------------------CTATAAACT-T-ATACAACTTTTAACAACGGATCTCTTGGCTCTCGCATCGATGAA
GAACGCAGCGAAATGCGATAAGTAATGTGAATTGCAGAATTCAGTGAATCATCGAATCTTTGAACGCACCTTGCGCCCTT
TGGTATTCCGAAGGGCATGCCTGTTTGAGTGTC-ATTAAATTC-TCAAACC------TCTTGCTA-----GT-TTAT---
--TCTAGTCTCG---------AGGCT---TTGGATAATGG-GA---G-TCTTTGCTGG--AACC--------TT--TAGG
GTTCAGCTCTCCTTAAATGCATTAGCGGTTAACTT----------------GATCCCCTTTCATTGG-TGTGATAA-TTA
TCTACGCCTTTCGAAATGATCAGCAGAATGT------TA-ATAGCTTCTAATCAG-----TCCCTAGTC---GGACAAC-
------T--TT-----ATTGACCATTTGACCT-GCCTTCACCCTCGGTGTCCGCCAGCTCATCGTCGCTGTCAACAAGAT
GGACACTACCAAGGTTCG----TTTTCTTTAT-TTC---------------TTTTCCGAAAAAG------CTCAAG--CT
GATTCCCCAACAGTGGAGCGAAGATCGTTTCAACGAAATTATCAAGGAGACCTCGACTTTCATCAAAAAGGTTGGCTACA
ACCCCAAAGCTGTTGCCTTTGTGCCCATTCTGGATGGCACGG-TGACAACAT--GTTGGAGGAGTCCCCCAAGTG----
---------CGTCTTTTTTTCAATCGTGACACTAACATTCGCTAATT------ACTTTACAGCATGCCATGG-TTCAAGG
GCTGGACCAAGGAGAC---CAAGGCTGGT--GTCTCCAAGGGAAAGACCCTTCTC---GAGGCCATCG------------
-ATGCTATCGAGCCCCCTGTTCGTCCATCAGAC-
Mycena_aurantiidisca_AFTOL_ID_1685
TTTGG-AG-TTG-AGCTGGCTGTTTCG-------GCAGCA-TGGGCTTGCTTC----ATTCTTCT---TTT----CCACC
-TGTGCACCTTTTG-TAGGGCTATGTACC---
-TCTCGTGCGTTKGGGYTTT-----------------------ATTGTYTATTTCGTCGG--ATATCAGAGGG---GCT-
------------------TT-CTCGGCCCCTTTGTAC---
--------------------------TTCA---------TAGT---------------------CCCTATG------TTT
TA----------------------------TACA-CTCG-----TTAAAAACCT-AGAATGTC----TTTGTGGGTCTTG
TAC---------------------CTATAAACT-T-ATACAACTTTTAACAACGGATCTCTTGGCTCTCGCATCGATGAA
GAACGCAGCGAAATGCGATAAGTAATGTGAATTGCAGAATTCAGTGAATCATCGAATCTTTGAACGCACCTTGCGCCCTT
TGGTATTCCGAAGGGCATGCCTGTTTGAGTGTC-ATTAAATTC-TCAAACC-----TTCTTGCTA-----GT-TTAT---
--TCTGGTTTTG--------GAGGCT---TTGGAT-ATGG-GA---G-TCTTTGCTGGA-AACCTA--TTCATTT-AGGG
GTTCAGCTCTCCTYAAATGCATTAGCGGTTAACTT----------------GATCCCCTTTCATTGG-TGTGATAA-TTA
TCTACGCCTGTTGAAATGATCATCAGAATGT------TA-ATAGCTTCTAATCCG-----TCCCTAGTT---GGACAAC-
------T--TT-----ATTGACCA--

Mycena_acicula_H6036823
AACACAAG-TTGTTGCTGGCT-CCCTA-------GGGGCA-TGTGCACACTTGTA--AGTCTTCA-ACTCT-CTTCCCCT
TTGTGCACTTTTGGCTAGTACTATATACT---
----------------------------------TGAAGCGAGAAATGTTTCTCTAACC---------GGAAA-------
------------------------CGTTTTGTTGATC---
-------------------------TTTTA-------GTGTGT----------------------GCTATGCACC-TTTT
TA----------------------------CAAA----------CGTATGTTTT-TGAATGTCA--TTGCAAGGTCTTTG
TAC---------------------CTGTAAATCTATATACAACTTTCAGCAACGGATCTCTTGGCTCTCGCATCGATGAA
GAACGCAGCGAAATGCGATAATTAATGTGAATTGCAGAATTCAGTGAATCATCGAATCTTTGAACGCACCTTGCACCCTT
TGGTATTCCGAAGGGTATGCCTGTTTGAGTGTC-ATTAAATTC-TCAA---------CCATACAA-----GT-TTTTTC-
---------TTG--------TAAGGC---TTGGAT-GTGG-AG---G--CTTTGCAGGT-TCCTT-----------AGTG

AATCTGCTCCTCTTAAATGCATTAGTGGGAACCGT--TTGTAGGGGTTCATTTATGTATTCACTTGA-TGTGATAG-TTG
TATCATCATCGATATGTGAATTACGGGAACTCTGAG-GCTCTTGCTTCTAACAG------TCTTGAACA--AAGACAAT-
------T--TA-----AATCTCAATTTGACCT--

Mycena_acicula_AH56040
AACACAAG-TTGTTGCTGGCT-CCCTA-------GGGGCA-TGTGCACACTTGTA--AGTCTTCA-ACTCT---TCCCCT
TTGTGCACTTTTGGCTAGTACTATATACT---
----------------------------------TGAAGCGAGAAATGTTTCTCTAACC---------GGAAA-------
------------------------CGTTTTGTTGATC---
-------------------------TTTTA-------GTGTGT----------------------GCTATGCACC-TTTT
TA----------------------------CAAA----------CGTATGTTTT-TGAATGTCA--TTGCAAGGTCTTTG
TAC---------------------CTGTAAATCTATATACAACTTTCAGCAACGGATCTCTTGGCTCTCGCATCGATGAA
GAACGCAGCGAAATGCGATAATTAATGTGAATTGCAGAATTCAGTGAATCATCGAATCTTTGAACGCACCTTGCACCCTT
TGGTATTCCGAAGGGTATGCCTGTTTGAGTGTC-ATTAAATTC-TCAA---------CCATACAA-----GT-TTTTTC-
---------TTG--------TAAGGC---TTGGAT-GTGG-AG---G--CTTTGCAGGT-TCCTT-----------AGTG
AATCTGCTCCTCTTAAATGCATTAGTGGGAACCGT--TTGTAGGGGTTCATTTATGTATTCACTTGA-TGTGATAG-TTG
TATCATCATCGATATGTGAATTACGGGAACTCTGAG-GCTCTTGCTTCTAACAG------TCTTGAACA--AAGACAAT-
------T--TA-----AATCTCAATTTGACC---

Mycena_aurantiidisca_PBM1282

---------------------------------GCCTTCACTCTCGGTGTTCGTCAGCTCATCGTCGCCGTCAACAAGAT
GGACACTACCAAGGCTCG----TCTTCATTTC-TTT---------------GTTTTT---AGAG------TTCAGG--CT

AAGATAGCAACAGTGGAGCGAGGACCGTTTCAACGAAATTATCAAGGAAACCTCTACCTTCATCAAAAAGGTTGGCTACA
ACCCCAAAGCTGTTGCCTTTGTTCCCATCTCTGGGTGGCACGG-TGACAACAT--GATGGAGGAGTCCCCCAAGTA----
----------CGCTTTTCTTTCGACTATGAAG--AAGATTTACCAATC------GTTTAACAGCATGCCATGG-TTCAAGG
GCTGGACCAAGGAGAC---CAAGGCTGGT--GTCACCAAGGGCAAGACCCTTCTC---GAGGCCATCG------------
-ATGCCATCGAGCCCCCTGTTCGTCCTTCAGAC-
