# Supplementary material for: Unveiling the diversity of the families Cyphellaceae and Mycenaceae from Southeast Asia
Source: IMA Fungus. 2026 Jul 10;17:e182174. doi: 10.3897/imafungus.17.182174 (PMC13379713; doi:10.3897/imafungus.17.182174)
Supplement: Supplementary material 2 — Model Test Output 1 [file imafungus-17-e182174-s002.pdf]

```
P A U P *
Version 4.0b10 for 32-bit Microsoft Windows
Thu Nov 13 14:08:34 2025


-----------------------------NOTICE-----------------------------
      This is a beta-test version.  Please report any crashes,
      apparent calculation errors, or other anomalous results.
      There are no restrictions on publication of results obtained
      with this version, but you should check the WWW site
      frequently for bug announcements and/or updated versions.
      See the README file on the distribution media for details.

----------------------------------------------------------------


Neighbor-joining search settings:
  Ties (if encountered) will be broken randomly; initial seed =
1778476860
  Distance measure = Jukes-Cantor
  (Tree is unrooted)

   Note: Ties were encountered; neighbor-joining tree may not be unique

   Tree found by neighbor-joining method stored in tree buffer
   Time used = 0.00 sec

***** BEGIN TESTING 24 MODELS OF EVOLUTION *****

    ** Model 1 of 24 * Calculating JC **

Likelihood scores of tree(s) in memory:
  Likelihood settings:
    Number of substitution types  = 1
    Assumed nucleotide frequencies (set by user):
      A=0.25000  C=0.25000  G=0.25000  T=0.25000
    Among-site rate variation:
      Assumed proportion of invariable sites  = none
      Distribution of rates at variable sites = equal
    These settings correspond to the JC69 model
    Number of distinct data patterns under this model = 916
    Molecular clock not enforced
    Starting branch lengths obtained using Rogers-Swofford
approximation
```

```
     method
   Branch-length optimization = one-dimensional Newton-Raphson with
pass
                        limit=20, delta=1e-006
   -ln L (unconstrained) = unavailable due to missing-data and/or
ambiguities

Writing likelihood scores (and parameter estimates) to file
"L:\Ñ¡ÔñÄ£ÐÍ\MrModeltest2.3\mrmodel.scores"

Tree number 1:
  -Ln likelihood = 20998.61904

Time used to compute likelihoods = 0.25 sec

   ** Model 2 of 24 * Calculating JC+I **

Likelihood scores of tree(s) in memory:
  Likelihood settings:
    Number of substitution types  = 1
    Assumed nucleotide frequencies (set by user):
      A=0.25000  C=0.25000  G=0.25000  T=0.25000
    Among-site rate variation:
      Assumed proportion of invariable sites  = estimated
      Distribution of rates at variable sites = equal
    These settings correspond to the JC69+I model
    Number of distinct data patterns under this model = 916
    Molecular clock not enforced
    Starting   branch   lengths   obtained   using   Rogers-Swofford
approximation
     method
   Branch-length optimization = one-dimensional Newton-Raphson with
pass
                        limit=20, delta=1e-006
   -ln L (unconstrained) = unavailable due to missing-data and/or
ambiguities

Writing likelihood scores (and parameter estimates) to file
"L:\Ñ¡ÔñÄ£ÐÍ\MrModeltest2.3\mrmodel.scores"

Tree number 1:
  -Ln likelihood = 20186.82358
  Estimated value of proportion of invariable sites = 0.248263
```

Time used to compute likelihoods = 1.67 sec

** Model 3 of 24 * Calculating JC+G **

Likelihood scores of tree(s) in memory:
Likelihood settings:
Number of substitution types = 1
Assumed nucleotide frequencies (set by user):
A=0.25000 C=0.25000 G=0.25000 T=0.25000
Among-site rate variation:
Assumed proportion of invariable sites = none
Distribution of rates at variable sites = gamma (discrete approximation)
Shape parameter (alpha) = estimated
Number of rate categories = 4
Representation of average rate for each category = mean
These settings correspond to the JC69+G model
Number of distinct data patterns under this model = 916
Molecular clock not enforced
Starting branch lengths obtained using Rogers-Swofford approximation
method
Branch-length optimization = one-dimensional Newton-Raphson with pass
limit=20, delta=1e-006
-ln L (unconstrained) = unavailable due to missing-data and/or ambiguities

Writing likelihood scores (and parameter estimates) to file "L:\Ñ¡ÔñÄ£ÐÍ\MrModeltest2.3\mrmodel.scores"

Tree number 1:
-Ln likelihood = 19516.70981
Estimated value of gamma shape parameter = 0.567450

Time used to compute likelihoods = 2.79 sec

** Model 4 of 24 * Calculating JC+I+G **

Likelihood scores of tree(s) in memory:
Likelihood settings:
Number of substitution types = 1
Assumed nucleotide frequencies (set by user):
A=0.25000 C=0.25000 G=0.25000 T=0.25000

```
    Among-site rate variation:
      Assumed proportion of invariable sites  = estimated
      Distribution  of  rates  at  variable  sites  =  gamma  (discrete
approximation)
        Shape parameter (alpha)   = estimated
        Number of rate categories = 4
        Representation of average rate for each category = mean
    These settings correspond to the JC69+G+I model
    Number of distinct data patterns under this model = 916
    Molecular clock not enforced
    Starting   branch   lengths   obtained   using   Rogers-Swofford
approximation
      method
    Branch-length optimization = one-dimensional Newton-Raphson with
pass
                                 limit=20, delta=1e-006
    -ln L (unconstrained) = unavailable due to missing-data and/or
ambiguities

Writing likelihood scores (and parameter estimates) to file
"L:\Ñ¡ÔñÄ£ÐÍ\MrModeltest2.3\mrmodel.scores"

Tree number 1:
  -Ln likelihood = 19500.30614
  Estimated value of proportion of invariable sites = 0.157918
  Estimated value of gamma shape parameter = 0.878463

Time used to compute likelihoods = 21.65 sec

    ** Model 5 of 24 * Calculating F81 **

Likelihood scores of tree(s) in memory:
  Likelihood settings:
    Number of substitution types  = 1
    Nucleotide frequencies estimated via ML
    Among-site rate variation:
      Assumed proportion of invariable sites  = none
      Distribution of rates at variable sites = equal
    These settings correspond to the F81 model
    Number of distinct data patterns under this model = 1057
    Molecular clock not enforced
    Starting   branch   lengths   obtained   using   Rogers-Swofford
approximation
      method
```

```
    Branch-length optimization = one-dimensional Newton-Raphson with
pass
                             limit=20, delta=1e-006
    -ln L (unconstrained) = unavailable due to missing-data and/or
ambiguities

Writing likelihood scores (and parameter estimates) to file
"L:\Ñ¡ÔñÄ£ĐÍ\MrModeltest2.3\mrmodel.scores"

Tree number 1:
  -Ln likelihood = 20919.46502
  Estimated base frequencies = A:0.220450 C:0.263678 G:0.199722
T:0.316151

Time used to compute likelihoods = 3.01 sec

    ** Model 6 of 24 * Calculating F81+I **

Likelihood scores of tree(s) in memory:
  Likelihood settings:
    Number of substitution types  = 1
    Nucleotide frequencies estimated via ML
    Among-site rate variation:
      Assumed proportion of invariable sites  = estimated
      Distribution of rates at variable sites = equal
    These settings correspond to the F81+I model
    Number of distinct data patterns under this model = 1057
    Molecular clock not enforced
    Starting branch lengths obtained using Rogers-Swofford
approximation
      method
    Branch-length optimization = one-dimensional Newton-Raphson with
pass
                             limit=20, delta=1e-006
    -ln L (unconstrained) = unavailable due to missing-data and/or
ambiguities

Writing likelihood scores (and parameter estimates) to file
"L:\Ñ¡ÔñÄ£ĐÍ\MrModeltest2.3\mrmodel.scores"

Tree number 1:
  -Ln likelihood = 20091.24622
  Estimated base frequencies = A:0.214185 C:0.268895 G:0.196446
T:0.320473
```

  Estimated value of proportion of invariable sites = 0.249401

Time used to compute likelihoods = 11.06 sec

    ** Model 7 of 24 * Calculating F81+G **

Likelihood scores of tree(s) in memory:
  Likelihood settings:
    Number of substitution types = 1
    Nucleotide frequencies estimated via ML
    Among-site rate variation:
      Assumed proportion of invariable sites = none
      Distribution of rates at variable sites = gamma (discrete approximation)
        Shape parameter (alpha)   = estimated
        Number of rate categories = 4
        Representation of average rate for each category = mean
    These settings correspond to the F81+G model
    Number of distinct data patterns under this model = 1057
    Molecular clock not enforced
    Starting branch lengths obtained using Rogers-Swofford approximation
      method
    Branch-length optimization = one-dimensional Newton-Raphson with pass
                              limit=20, delta=1e-006
    -ln L (unconstrained) = unavailable due to missing-data and/or ambiguities

Writing likelihood scores (and parameter estimates) to file
"L:\Ñ¡ÔñÄ£ÐÍ\MrModeltest2.3\mrmodel.scores"

Tree number 1:
  -Ln likelihood = 19396.07125
  Estimated base frequencies = A:0.210423 C:0.273838 G:0.187075 T:0.328664
  Estimated value of gamma shape parameter = 0.546673

Time used to compute likelihoods = 19.80 sec

    ** Model 8 of 24 * Calculating F81+I+G **

Likelihood scores of tree(s) in memory:
  Likelihood settings:

```
    Number of substitution types  = 1
    Nucleotide frequencies estimated via ML
    Among-site rate variation:
      Assumed proportion of invariable sites  = estimated
      Distribution  of  rates  at  variable  sites  =  gamma  (discrete
approximation)
        Shape parameter (alpha)   = estimated
        Number of rate categories = 4
        Representation of average rate for each category = mean
    These settings correspond to the F81+G+I model
    Number of distinct data patterns under this model = 1057
    Molecular clock not enforced
    Starting   branch   lengths   obtained   using   Rogers-Swofford
approximation
      method
    Branch-length optimization = one-dimensional Newton-Raphson with
pass
                                 limit=20, delta=1e-006
    -ln L (unconstrained) = unavailable due to missing-data and/or
ambiguities

Writing likelihood scores (and parameter estimates) to file
"L:\Ñ¡ÔñÄ£ÐÍ\MrModeltest2.3\mrmodel.scores"

Tree number 1:
  -Ln likelihood = 19378.74694
  Estimated  base  frequencies  =  A:0.210396  C:0.273988  G:0.187251
T:0.328366
  Estimated value of proportion of invariable sites = 0.154868
  Estimated value of gamma shape parameter = 0.829974

Time used to compute likelihoods = 25.42 sec

    ** Model 9 of 24 * Calculating K80 **

Likelihood scores of tree(s) in memory:
  Likelihood settings:
    Number of substitution types  = 2
    Transition/transversion ratio estimated via ML
    Assumed nucleotide frequencies (set by user):
      A=0.25000  C=0.25000  G=0.25000  T=0.25000
    Among-site rate variation:
      Assumed proportion of invariable sites  = none
      Distribution of rates at variable sites = equal
```

```
    These settings correspond to the K80(K2P) model
    Number of distinct data patterns under this model = 935
    Molecular clock not enforced
    Starting   branch   lengths   obtained   using   Rogers-Swofford
approximation
      method
    Branch-length optimization = one-dimensional Newton-Raphson with
pass
                              limit=20, delta=1e-006
    -ln L (unconstrained) = unavailable due to missing-data and/or
ambiguities

Writing likelihood scores (and parameter estimates) to file
"L:\Ñ¡ÔñÄ£ÐÍ\MrModeltest2.3\mrmodel.scores"

Tree number 1:
  -Ln likelihood = 20562.19358
  Estimated ti/tv ratio = 1.386718 (kappa = 2.773436)

Time used to compute likelihoods = 0.58 sec

    ** Model 10 of 24 * Calculating K80+I **

Likelihood scores of tree(s) in memory:
  Likelihood settings:
    Number of substitution types  = 2
    Transition/transversion ratio estimated via ML
    Assumed nucleotide frequencies (set by user):
      A=0.25000  C=0.25000  G=0.25000  T=0.25000
    Among-site rate variation:
      Assumed proportion of invariable sites  = estimated
      Distribution of rates at variable sites = equal
    These settings correspond to the K80(K2P)+I model
    Number of distinct data patterns under this model = 935
    Molecular clock not enforced
    Starting   branch   lengths   obtained   using   Rogers-Swofford
approximation
      method
    Branch-length optimization = one-dimensional Newton-Raphson with
pass
                              limit=20, delta=1e-006
    -ln L (unconstrained) = unavailable due to missing-data and/or
ambiguities
```

Writing likelihood scores (and parameter estimates) to file
"L:\Ñ¡ÔñÄ£ÐÍ\MrModeltest2.3\mrmodel.scores"

Tree number 1:
  -Ln likelihood = 19736.98152
  Estimated ti/tv ratio = 1.445573 (kappa = 2.891146)
  Estimated value of proportion of invariable sites = 0.248489

Time used to compute likelihoods = 11.80 sec

    ** Model 11 of 24 * Calculating K80+G **

Likelihood scores of tree(s) in memory:
  Likelihood settings:
    Number of substitution types = 2
    Transition/transversion ratio estimated via ML
    Assumed nucleotide frequencies (set by user):
      A=0.25000 C=0.25000 G=0.25000 T=0.25000
    Among-site rate variation:
      Assumed proportion of invariable sites = none
      Distribution of rates at variable sites = gamma (discrete approximation)
        Shape parameter (alpha)   = estimated
        Number of rate categories = 4
        Representation of average rate for each category = mean
    These settings correspond to the K80(K2P)+G model
    Number of distinct data patterns under this model = 935
    Molecular clock not enforced
    Starting branch lengths obtained using Rogers-Swofford approximation
      method
    Branch-length optimization = one-dimensional Newton-Raphson with pass
                              limit=20, delta=1e-006
    -ln L (unconstrained) = unavailable due to missing-data and/or ambiguities

Writing likelihood scores (and parameter estimates) to file
"L:\Ñ¡ÔñÄ£ÐÍ\MrModeltest2.3\mrmodel.scores"

Tree number 1:
  -Ln likelihood = 19036.22984
  Estimated ti/tv ratio = 1.629996 (kappa = 3.259993)
  Estimated value of gamma shape parameter = 0.547256

Time used to compute likelihoods = 14.25 sec

** Model 12 of 24 * Calculating K80+I+G **

Likelihood scores of tree(s) in memory:
  Likelihood settings:
    Number of substitution types = 2
    Transition/transversion ratio estimated via ML
    Assumed nucleotide frequencies (set by user):
      A=0.25000 C=0.25000 G=0.25000 T=0.25000
    Among-site rate variation:
      Assumed proportion of invariable sites = estimated
      Distribution of rates at variable sites = gamma (discrete approximation)
        Shape parameter (alpha)   = estimated
        Number of rate categories = 4
        Representation of average rate for each category = mean
    These settings correspond to the K80(K2P)+G+I model
    Number of distinct data patterns under this model = 935
    Molecular clock not enforced
    Starting branch lengths obtained using Rogers-Swofford approximation
      method
    Branch-length optimization = one-dimensional Newton-Raphson with pass
                              limit=20, delta=1e-006
    -ln L (unconstrained) = unavailable due to missing-data and/or ambiguities

Writing likelihood scores (and parameter estimates) to file
"L:\Ñ¡ÔñÄ£ÐÍ\MrModeltest2.3\mrmodel.scores"

Tree number 1:
  -Ln likelihood = 19018.90884
  Estimated ti/tv ratio = 1.633015 (kappa = 3.266030)
  Estimated value of proportion of invariable sites = 0.156833
  Estimated value of gamma shape parameter = 0.831558

Time used to compute likelihoods = 23.83 sec

** Model 13 of 24 * Calculating HKY **

Likelihood scores of tree(s) in memory:

```
  Likelihood settings:
    Number of substitution types  = 2 (HKY85 variant)
    Transition/transversion ratio estimated via ML
    Nucleotide frequencies estimated via ML
    Among-site rate variation:
      Assumed proportion of invariable sites  = none
      Distribution of rates at variable sites = equal
    These settings correspond to the HKY85 model
    Number of distinct data patterns under this model = 1057
    Molecular clock not enforced
    Starting   branch   lengths   obtained   using   Rogers-Swofford
approximation
      method
    Branch-length optimization = one-dimensional Newton-Raphson with
pass
                            limit=20, delta=1e-006
    -ln L (unconstrained) = unavailable due to missing-data and/or
ambiguities

Writing likelihood scores (and parameter estimates) to file
"L:\Ñ¡ÔñÄ£ÐÍ\MrModeltest2.3\mrmodel.scores"

Tree number 1:
  -Ln likelihood = 20493.47618
  Estimated  base  frequencies  =  A:0.227443  C:0.248258  G:0.204776
T:0.319523
  Estimated ti/tv ratio = 1.410600 (kappa = 2.749580)

Time used to compute likelihoods = 3.82 sec

    ** Model 14 of 24 * Calculating HKY+I **

Likelihood scores of tree(s) in memory:
  Likelihood settings:
    Number of substitution types  = 2 (HKY85 variant)
    Transition/transversion ratio estimated via ML
    Nucleotide frequencies estimated via ML
    Among-site rate variation:
      Assumed proportion of invariable sites  = estimated
      Distribution of rates at variable sites = equal
    These settings correspond to the HKY85+I model
    Number of distinct data patterns under this model = 1057
    Molecular clock not enforced
    Starting   branch   lengths   obtained   using   Rogers-Swofford
```

approximation
method
Branch-length optimization = one-dimensional Newton-Raphson with pass
limit=20, delta=1e-006
-ln L (unconstrained) = unavailable due to missing-data and/or ambiguities

Writing likelihood scores (and parameter estimates) to file "L:\Ñ¡ÔñÄ£ÐÍ\MrModeltest2.3\mrmodel.scores"

Tree number 1:
-Ln likelihood = 19674.02289
Estimated base frequencies = A:0.225451 C:0.250191 G:0.207972 T:0.316387
Estimated ti/tv ratio = 1.440685 (kappa = 2.806831)
Estimated value of proportion of invariable sites = 0.248367

Time used to compute likelihoods = 16.24 sec

** Model 15 of 24 * Calculating HKY+G **

Likelihood scores of tree(s) in memory:
Likelihood settings:
Number of substitution types = 2 (HKY85 variant)
Transition/transversion ratio estimated via ML
Nucleotide frequencies estimated via ML
Among-site rate variation:
Assumed proportion of invariable sites = none
Distribution of rates at variable sites = gamma (discrete approximation)
Shape parameter (alpha) = estimated
Number of rate categories = 4
Representation of average rate for each category = mean
These settings correspond to the HKY85+G model
Number of distinct data patterns under this model = 1057
Molecular clock not enforced
Starting branch lengths obtained using Rogers-Swofford approximation
method
Branch-length optimization = one-dimensional Newton-Raphson with pass
limit=20, delta=1e-006
-ln L (unconstrained) = unavailable due to missing-data and/or

ambiguities

Writing likelihood scores (and parameter estimates) to file "L:\Ñ¡ÔñÄ£ÐÍ\MrModeltest2.3\mrmodel.scores"

Tree number 1:
  -Ln likelihood = 18962.66526
  Estimated base frequencies = A:0.229010 C:0.249742 G:0.199273 T:0.321975
  Estimated ti/tv ratio = 1.629504 (kappa = 3.165468)
  Estimated value of gamma shape parameter = 0.540993

Time used to compute likelihoods = 23.28 sec

** Model 16 of 24 * Calculating HKY+I+G **

Likelihood scores of tree(s) in memory:
  Likelihood settings:
    Number of substitution types = 2 (HKY85 variant)
    Transition/transversion ratio estimated via ML
    Nucleotide frequencies estimated via ML
    Among-site rate variation:
      Assumed proportion of invariable sites = estimated
      Distribution of rates at variable sites = gamma (discrete approximation)
        Shape parameter (alpha) = estimated
        Number of rate categories = 4
        Representation of average rate for each category = mean
    These settings correspond to the HKY85+G+I model
    Number of distinct data patterns under this model = 1057
    Molecular clock not enforced
    Starting branch lengths obtained using Rogers-Swofford approximation
      method
    Branch-length optimization = one-dimensional Newton-Raphson with pass
                                 limit=20, delta=1e-006
    -ln L (unconstrained) = unavailable due to missing-data and/or ambiguities

Writing likelihood scores (and parameter estimates) to file "L:\Ñ¡ÔñÄ£ÐÍ\MrModeltest2.3\mrmodel.scores"

Tree number 1:

```
  -Ln likelihood = 18945.81995
  Estimated base frequencies = A:0.228375 C:0.250107 G:0.199812 
T:0.321706
  Estimated ti/tv ratio = 1.628123 (kappa = 3.161430)
  Estimated value of proportion of invariable sites = 0.155250
  Estimated value of gamma shape parameter = 0.817453

Time used to compute likelihoods = 34.54 sec

    ** Model 17 of 24 * Calculating SYM **

Likelihood scores of tree(s) in memory:
  Likelihood settings:
    Number of substitution types  = 6
    Substitution rate-matrix parameters estimated via ML
    Assumed nucleotide frequencies (set by user):
      A=0.25000  C=0.25000  G=0.25000  T=0.25000
    Among-site rate variation:
      Assumed proportion of invariable sites  = none
      Distribution of rates at variable sites = equal
    These settings correspond to a submodel of the GTR model
    Number of distinct data patterns under this model = 1057
    Molecular clock not enforced
    Starting branch lengths obtained using Rogers-Swofford 
approximation
      method
    Branch-length optimization = one-dimensional Newton-Raphson with 
pass
                              limit=20, delta=1e-006
    -ln L (unconstrained) = unavailable due to missing-data and/or 
ambiguities

Writing likelihood scores (and parameter estimates) to file
"L:\Ñ¡ÔñÄ£ÐÍ\MrModeltest2.3\mrmodel.scores"

Tree number 1:
  -Ln likelihood = 20489.39963
  Estimated R-matrix:
              -    1.1127332    2.2293077    1.3084689
      1.1127332            -   0.94941372    3.7320672
      2.2293077   0.94941372            -            1
      1.3084689    3.7320672            1            -

Time used to compute likelihoods = 4.15 sec
```

```
   ** Model 18 of 24 * Calculating SYM+I **

Likelihood scores of tree(s) in memory:
  Likelihood settings:
    Number of substitution types  = 6
    Substitution rate-matrix parameters estimated via ML
    Assumed nucleotide frequencies (set by user):
      A=0.25000  C=0.25000  G=0.25000  T=0.25000
    Among-site rate variation:
      Assumed proportion of invariable sites  = estimated
      Distribution of rates at variable sites = equal
    These settings correspond to a submodel of the GTR+I model
    Number of distinct data patterns under this model = 1057
    Molecular clock not enforced
    Starting   branch   lengths   obtained   using   Rogers-Swofford
approximation
      method
    Branch-length optimization = one-dimensional Newton-Raphson with
pass
                              limit=20, delta=1e-006
    -ln L (unconstrained) = unavailable due to missing-data and/or
ambiguities

Writing likelihood scores (and parameter estimates) to file
"L:\Ñ¡ÔñÄ£ÐÍ\MrModeltest2.3\mrmodel.scores"

Tree number 1:
  -Ln likelihood = 19689.57933
  Estimated R-matrix:
              -     1.1448845    2.5310409    1.3758238
       1.1448845           -    0.93562401    3.7510585
       2.5310409   0.93562401           -           1
       1.3758238    3.7510585           1           -
  Estimated value of proportion of invariable sites = 0.245312

Time used to compute likelihoods = 16.65 sec

   ** Model 19 of 24 * Calculating SYM+G **

Likelihood scores of tree(s) in memory:
  Likelihood settings:
    Number of substitution types  = 6
    Substitution rate-matrix parameters estimated via ML
```

```
    Assumed nucleotide frequencies (set by user):
      A=0.25000  C=0.25000  G=0.25000  T=0.25000
    Among-site rate variation:
      Assumed proportion of invariable sites  = none
      Distribution  of  rates  at  variable  sites  =  gamma  (discrete
approximation)
        Shape parameter (alpha)   = estimated
        Number of rate categories = 4
        Representation of average rate for each category = mean
    These settings correspond to a submodel of the GTR+G model
    Number of distinct data patterns under this model = 1057
    Molecular clock not enforced
    Starting   branch   lengths   obtained   using   Rogers-Swofford
approximation
      method
    Branch-length optimization = one-dimensional Newton-Raphson with
pass
                             limit=20, delta=1e-006
    -ln L  (unconstrained) = unavailable due to missing-data and/or
ambiguities

Writing likelihood scores (and parameter estimates) to file
"L:\Ñ¡ÔñÄ£ÐÍ\MrModeltest2.3\mrmodel.scores"

Tree number 1:
  -Ln likelihood = 18984.16000
  Estimated R-matrix:
              -     1.0799814      2.44113      1.33805
        1.0799814           -    0.87965578    4.4670675
          2.44113    0.87965578           -            1
          1.33805     4.4670675           1            -
  Estimated value of gamma shape parameter = 0.554951

Time used to compute likelihoods = 29.62 sec

    ** Model 20 of 24 * Calculating SYM+I+G **

Likelihood scores of tree(s) in memory:
  Likelihood settings:
    Number of substitution types  = 6
    Substitution rate-matrix parameters estimated via ML
    Assumed nucleotide frequencies (set by user):
      A=0.25000  C=0.25000  G=0.25000  T=0.25000
    Among-site rate variation:
```

Assumed proportion of invariable sites = estimated
Distribution of rates at variable sites = gamma (discrete approximation)
Shape parameter (alpha) = estimated
Number of rate categories = 4
Representation of average rate for each category = mean
These settings correspond to a submodel of the GTR+G+I model
Number of distinct data patterns under this model = 1057
Molecular clock not enforced
Starting branch lengths obtained using Rogers-Swofford approximation method
Branch-length optimization = one-dimensional Newton-Raphson with pass limit=20, delta=1e-006
-ln L (unconstrained) = unavailable due to missing-data and/or ambiguities

Writing likelihood scores (and parameter estimates) to file "L:\Ñ¡ÔñÄ£ÐÍ\MrModeltest2.3\mrmodel.scores"

Tree number 1:
-Ln likelihood = 18967.74676
Estimated R-matrix:

-	1.0831461	2.4821372	1.3550781
1.0831461	-	0.86557891	4.4435084
2.4821372	0.86557891	-	1
1.3550781	4.4435084	1	-

Estimated value of proportion of invariable sites = 0.152312
Estimated value of gamma shape parameter = 0.832913

Time used to compute likelihoods = 55.83 sec

** Model 21 of 24 * Calculating GTR **

Likelihood scores of tree(s) in memory:
Likelihood settings:
Number of substitution types = 6
Substitution rate-matrix parameters estimated via ML
Nucleotide frequencies estimated via ML
Among-site rate variation:
Assumed proportion of invariable sites = none
Distribution of rates at variable sites = equal
These settings correspond to the GTR model

```
    Number of distinct data patterns under this model = 1057
    Molecular clock not enforced
    Starting branch lengths obtained using Rogers-Swofford
approximation
      method
    Branch-length optimization = one-dimensional Newton-Raphson with
pass
                            limit=20, delta=1e-006
    -ln L (unconstrained) = unavailable due to missing-data and/or
ambiguities

Writing likelihood scores (and parameter estimates) to file
"L:\Ñ¡ÔñÄ£ÐÍ\MrModeltest2.3\mrmodel.scores"

Tree number 1:
  -Ln likelihood = 20461.79636
  Estimated base frequencies = A:0.239695 C:0.227214 G:0.233797
T:0.299294
  Estimated R-matrix:
                -     1.2665043    2.4831557    1.2858237
        1.2665043          -       1.0942562    3.8264294
        2.4831557    1.0942562          -            1
        1.2858237    3.8264294          1            -

Time used to compute likelihoods = 6.57 sec

    ** Model 22 of 24 * Calculating GTR+I **

Likelihood scores of tree(s) in memory:
  Likelihood settings:
    Number of substitution types  = 6
    Substitution rate-matrix parameters estimated via ML
    Nucleotide frequencies estimated via ML
    Among-site rate variation:
      Assumed proportion of invariable sites  = estimated
      Distribution of rates at variable sites = equal
    These settings correspond to the GTR+I model
    Number of distinct data patterns under this model = 1057
    Molecular clock not enforced
    Starting branch lengths obtained using Rogers-Swofford
approximation
      method
    Branch-length optimization = one-dimensional Newton-Raphson with
pass
```

```
                        limit=20, delta=1e-006
   -ln L (unconstrained) = unavailable due to missing-data and/or
ambiguities

Writing likelihood scores (and parameter estimates) to file
"L:\Ñ¡ÔñÄ£ÐÍ\MrModeltest2.3\mrmodel.scores"

Tree number 1:
  -Ln likelihood = 19657.69778
  Estimated base frequencies = A:0.223896 C:0.241241 G:0.229256
T:0.305607
  Estimated R-matrix:
             -    1.3028971    2.9408918    1.4054608
      1.3028971           -    1.0491572    3.6516331
      2.9408918    1.0491572           -            1
      1.4054608    3.6516331           1            -
  Estimated value of proportion of invariable sites = 0.246549

Time used to compute likelihoods = 17.34 sec

   ** Model 23 of 24 * Calculating GTR+G **

Likelihood scores of tree(s) in memory:
  Likelihood settings:
    Number of substitution types  = 6
    Substitution rate-matrix parameters estimated via ML
    Nucleotide frequencies estimated via ML
    Among-site rate variation:
      Assumed proportion of invariable sites  = none
      Distribution of rates at variable sites = gamma (discrete
approximation)
        Shape parameter (alpha)   = estimated
        Number of rate categories = 4
        Representation of average rate for each category = mean
    These settings correspond to the GTR+G model
    Number of distinct data patterns under this model = 1057
    Molecular clock not enforced
    Starting branch lengths obtained using Rogers-Swofford
approximation
      method
    Branch-length optimization = one-dimensional Newton-Raphson with
pass
                        limit=20, delta=1e-006
   -ln L (unconstrained) = unavailable due to missing-data and/or
```

ambiguities

Writing likelihood scores (and parameter estimates) to file "L:\Ñ¡ÔñÄ£ÐÍ\MrModeltest2.3\mrmodel.scores"

Tree number 1:

-Ln likelihood = 18953.71379

Estimated base frequencies = A:0.232483 C:0.234724 G:0.221387 T:0.311406

Estimated R-matrix:

-	1.3349211	3.1106278	1.2849336
1.3349211	-	1.1293043	4.3680543
3.1106278	1.1293043	-	1
1.2849336	4.3680543	1	-

Estimated value of gamma shape parameter = 0.548387

Time used to compute likelihoods = 57.27 sec

** Model 24 of 24 * Calculating GTR+I+G **

Likelihood scores of tree(s) in memory:

Likelihood settings:

Number of substitution types = 6

Substitution rate-matrix parameters estimated via ML

Nucleotide frequencies estimated via ML

Among-site rate variation:

Assumed proportion of invariable sites = estimated

Distribution of rates at variable sites = gamma (discrete approximation)

Shape parameter (alpha) = estimated

Number of rate categories = 4

Representation of average rate for each category = mean

These settings correspond to the GTR+G+I model

Number of distinct data patterns under this model = 1057

Molecular clock not enforced

Starting branch lengths obtained using Rogers-Swofford approximation method

Branch-length optimization = one-dimensional Newton-Raphson with pass limit=20, delta=1e-006

-ln L (unconstrained) = unavailable due to missing-data and/or ambiguities

```
Writing likelihood scores (and parameter estimates) to file
"L:\Ñ¡ÔñÄ£ÐÍ\MrModeltest2.3\mrmodel.scores"

Tree number 1:
  -Ln likelihood = 18936.85671
  Estimated  base  frequencies  =  A:0.228805  C:0.237232  G:0.221576
T:0.312388
  Estimated R-matrix:
                 -     1.351191    3.2070042    1.3259589
          1.351191           -    1.1066194    4.3122697
         3.2070042    1.1066194           -            1
         1.3259589    4.3122697           1            -
  Estimated value of proportion of invariable sites = 0.155651
  Estimated value of gamma shape parameter = 0.830136

Time used to compute likelihoods = 00:01:23.3
```
